# The unique composition of Indian gut microbiome, gene catalogue, and associated fecal metabolome deciphered using multi-omics approaches

**DOI:** 10.1093/gigascience/giz004

**Published:** 2019-01-30

**Authors:** D B Dhakan, A Maji, A K Sharma, R Saxena, J Pulikkan, T Grace, A Gomez, J Scaria, K R Amato, V K Sharma

**Affiliations:** 1 Metagenomics and Systems Biology Laboratory, Department of Biological Sciences, Indian Institute of Science Education and Research Bhopal, Bhauri, Madhya Pradesh, 462066, India; 2 Department of Genomic Science, Central University of Kerala, Periye Post, Kasargod, Kerala, 671316, India; 3 Division of Biology, Kansas State University, 116 Ackert Hall, Manhattan, Kansas, KS 66506, USA; 4 Microbiomics Laboratory, Department of Animal Science, University of Minnesota, 1988 Fitch Avenue, Minnesota, MN 55108, USA; 5 Animal Disease Research & Diagnostic Laboratory, Veterinary and Biomedical Sciences Department, South Dakota State University, Brookings, South Dakota, SD 57007, USA; 6 Department of Anthropology, Northwestern University, 1810 Hinman Avenue, Evanston, Illinois, IL 60208, USA

**Keywords:** Indian gut microbiome, whole-genome shotgun, metagenomics, metabolomics, integrated gene catalog, metagenome-wide association study, core gut microbiome, short chain fatty acids, branched chain amino acids

## Abstract

**Background:**

Metagenomic studies carried out in the past decade have led to an enhanced understanding of the gut microbiome in human health; however, the Indian gut microbiome has not been well explored. We analyzed the gut microbiome of 110 healthy individuals from two distinct locations (North-Central and Southern) in India using multi-omics approaches, including 16S rRNA gene amplicon sequencing, whole-genome shotgun metagenomic sequencing, and metabolomic profiling of fecal and serum samples.

**Results:**

The gene catalogue established in this study emphasizes the uniqueness of the Indian gut microbiome in comparison to other populations. The gut microbiome of the cohort from North-Central India, which was primarily consuming a plant-based diet, was found to be associated with *Prevotella* and also showed an enrichment of branched chain amino acid (BCAA) and lipopolysaccharide biosynthesis pathways. In contrast, the gut microbiome of the cohort from Southern India, which was consuming an omnivorous diet, showed associations with *Bacteroides, Ruminococcus*, and *Faecalibacterium* and had an enrichment of short chain fatty acid biosynthesis pathway and BCAA transporters. This corroborated well with the metabolomics results, which showed higher concentration of BCAAs in the serum metabolome of the North-Central cohort and an association with *Prevotella*. In contrast, the concentration of BCAAs was found to be higher in the fecal metabolome of the Southern-India cohort and showed a positive correlation with the higher abundance of BCAA transporters.

**Conclusions:**

The study reveals the unique composition of the Indian gut microbiome, establishes the Indian gut microbial gene catalogue, and compares it with the gut microbiome of other populations. The functional associations revealed using metagenomic and metabolomic approaches provide novel insights on the gut-microbe-metabolic axis, which will be useful for future epidemiological and translational researches.

## Background

Determining the taxonomic and functional composition of a healthy gut microbiome across different populations is essential for understanding its role in maintaining human health. Several large-scale, world-wide microbiome projects have revealed variability in the gut microbial composition of healthy individuals due to factors such as mode of delivery, age, geographical location, diet, and lifestyle [1–5]. The majority of the gut microbiome studies have determined microbial taxonomy and functional diversity using 16S rRNA marker gene-based and/or whole-genome shotgun (WGS) approaches to understand the functional role of the gut microbiome. However, novel insights on the complex interplay among diet, gut microbes, and human health, along with the role of key microbial metabolites such as short chain fatty acids (SCFAs) and branched chain amino acids (BCAAs), derived from the microbial fermentation of dietary fibers, are beginning to emerge from recent gut metabolomics studies [6, 7]. Moreover, the direct impact of microbial metabolome on human health is also becoming apparent from the recent studies focusing on the “gut microbiome-host metabolism axis” [8]. Therefore, an integrative approach using both metagenome and metabolome-based characterizations of the gut microbiome appears pragmatic for gaining deeper functional and mechanistic insights into the role of gut microbes on human health.

The large-scale studies carried out so far mainly represent the gut microbiome of urban populations primarily from Europe, the United States, and other “WEIRD” countries (i.e., the Western, Educated, Industrialized, Rich, and Democratic countries) [9, 10]. Only recently, some studies have characterized the human microbiome from diverse ethnic populations and found significant compositional variations compared to the microbiomes from other previously studied populations [11–14]. India is the seventh largest country in the world and harbors the second largest population spread across multiple geographical locations with enormous diversity in ethnicity, lifestyles, and dietary habits. India is home to the majority of the world's vegetarian population but also has an almost equal representation of its population consuming animal-based diets. The Indian population also has the highest prevalence of diabetes in the world [15]. According to the World Health Organization estimates (WHO, 2011), 53% of deaths in India in the year 2008 were attributed to metabolic conditions such as diabetes and cardiovascular diseases, which are predicted to reach ∼75% by 2030 [16].

A few studies have investigated the gut microbiome of the Indian population. A recent study by Maji et al. has shown the functional association of human gut microbiome dysbiosis with tuberculosis through a time-course study of six tuberculosis patients in India [17]. However, other gut microbiome studies were mainly limited by small cohort sizes and amplicon-based (16S rRNA gene) sequencing and analysis [17–21]. Thus, several large-scale efforts are needed to identify the Indian population-specific microbiome biomarkers and to understand the impact of the gut microbiome on health and disease in the Indian population along with global comparisons.

However, to uncover the enormous gut microbiome diversity inherent in the different sub-populations of India, extensive sampling and analyses are required. Therefore, as the first large-scale study from India, we selected two prominent locations in North-Central India, i.e., LOC1: Bhopal city, Madhya Pradesh, and Southern India, i.e., LOC2: Kerala. The two locations also had different dietary habits. The Southern-India (LOC2) diet consisted of rice, meat, and fish, whereas the North-Central (LOC1) diet consisted of carbohydrate-rich food including plant-derived products, wheat, and trans-fat food (high-fat dairy, sweets, and fried snacks). The Human Development Index Report (UNDP; United Nations Development Programme), India and SRS-based life-table (Sample Registration Survey, 2010–14) has revealed that the citizens from Kerala had the highest life-expectancy rates (>74 years) in India, whereas those in Madhya Pradesh (capital city Bhopal) exhibited the lowest (<65 years) [22]. Further, a higher predisposition of the North-Indian population towards diabetes, cardiovascular diseases, and hypertension is also known, which in contrast is much lower in Southern India, perhaps due to the lifestyle differences in the two regions [15, 23]. Thus, to gain deeper functional insights into the microbiome from these two distinct sub-populations of India, a multi-omics approach was carried out using amplicon-based profiling of taxonomic composition (16S rRNA gene sequencing), WGS-based profiling of metagenome, and gas chromatography/mass spectrometry (GC-MS)-based profiling of fecal and serum metabolomic signatures.

## Data Description

The two selected locations, Bhopal (LOC1) and Kerala (LOC2) from North-Central and Southern parts of India, are about 2,000 kilometers apart and provided a distinct representation of the Indian population with respect to diet and lifestyle (Additional File 1). The 110 (62 females, 58 males) individuals recruited in this study were not suffering from any disease, as reported by personal medical history and physical examination, and confirmed no exposure to antibiotics for at least one month prior to sampling. Recruited individuals had an average body mass index (BMI) of 21.16 (±5.23 standard deviation), an average age of 29.72 years (±17.41 standard deviation), and were not diagnosed with any disease at the time of sample collection, and thus were considered as “healthy” (Additional File 1). Moreover, they did not have a second-degree relative history of T2D. The recruitment of volunteers, sample collection, and other study-related procedures were carried out by following the guidelines and protocols approved by the Institute Ethics Committee of the Indian Institute of Science Education and Research (IISER), Bhopal, India. The fecal samples were frozen within 30 minutes of collection and were then used for 16S rRNA gene V3 hypervariable region amplicon sequencing, WGS-based metagenomic sequencing, and metabolomic analysis. Further, the serum samples collected from a subset of volunteers were used for GC-MS-based metabolomics analysis. The sequencing of V3 hypervariable region of 16S rRNA gene and shotgun metagenome sequencing from the 110 fecal samples resulted in 54.87 million paired-end reads (503,460 ± 175,547 [mean ± standard deviation] reads/sample) and 499.98 million paired-end reads (4,545,280 ± 1,498,663 [mean ± standard deviation] reads/sample), respectively (Methods, Additional File 2 and Additional File 3). The metabolomic analysis was also performed on all fecal and subset of serum samples collected from the same healthy participants using GC-MS, and the resultant raw files were used for further analysis. The data description of participants and the data generated from each sample are provided in Additional File 1 under the Metadata information section.

## Analyses

### Construction of an Indian gut microbial gene catalogue and updated integrated gene catalogue

The first step for functional analysis was the construction of an extensive catalogue of gut microbial genes from the Indian population, which was not available previously. A De Bruijn graph-based assembly of reads resulted in 2,165,507 contigs of length ≥500 bp with a total contig size of 3.086 Gbp representing 68.25% of total reads and a mean N50 value of 2,288 bp. To obtain assemblies of low coverage genomic regions or genomes present in the Indian gut microbiome, the reads from each sample were mapped on assembled contigs obtained from their respective sample, and the remaining singletons (unassembled reads) were pooled and re-assembled together into an additional 45,839 contigs with length ≥500 bp and a total assembled length of 34.68 Mbp. A total of 1,551,581 non-redundant genes were predicted from contigs, which represent the gut microbial gene catalogue of the Indian cohorts.

**Figure 1:**
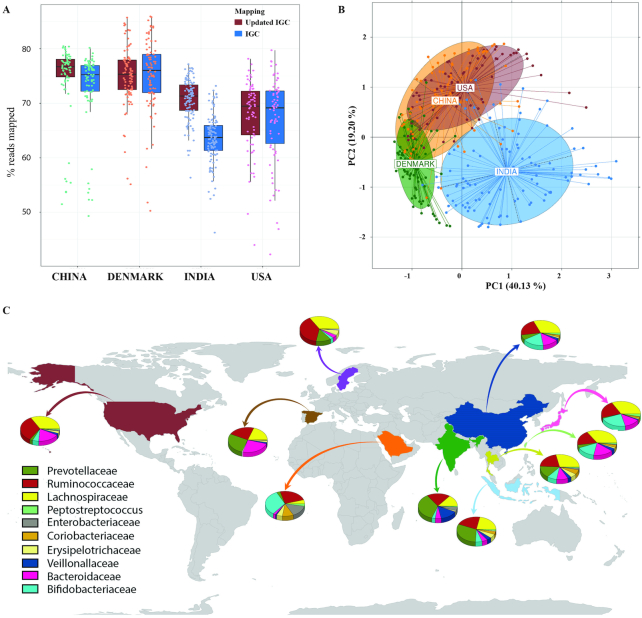
Comparison of the Indian gut microbiome with other major populations using 16S rRNA gene and metagenomic datasets. **(A)** Percentage of total reads that could be mapped to IGC and updated IGC containing the Indian gene catalogue. Plotted are interquartile ranges (IQR; in boxes), median (as dark lines in the boxes), lowest and highest values within 1.5 times the IQR (shown as whiskers extending from boxes), and outliers as points beyond these whiskers. The blue and red boxes show percentage of reads mapped to IGC and updated IGC (containing the Indian microbial genes). **(B)** Principal component analysis using metagenomic species (MGS)/co-abundance gene group (CAG) proportion derived from a metagenome-wide association study. Variations across populations are shown using PC1 and PC2. **(C)** Illustration of proportions of bacterial families in different populations and their composition as determined from 16S rRNA gene datasets (adult population only). The mean family compositions of abundant families (≥1%) are represented in separate pie plots from 10 different country-wise datasets, showing their overall microbial composition compared to Indian population.

The integrated gene catalogue (IGC) established by Li et al. in a previous multicohort study consisted of 9,879,896 genes identified from 1,267 gut metagenomes representing multiple populations [24]. A total of 943,395 genes (sharing < 90% identity with IGC) out of 1,551,581 from the Indian gut microbial gene catalogue were identified as unique to the Indian microbial gene catalogue. The IGC was updated to construct an “Updated-IGC” by adding these 943,395 non-redundant genes from the Indian gene catalogue. The updated-IGC consisting of 10,823,291 non-redundant genes (an 8.8% increase from IGC) was used as the reference gene catalogue for the subsequent analysis performed in this study. A total of 70.74% (±3.77% standard deviation) mapping coverage of reads (∼7.5% increase in the mapping of reads) was observed from the 110 Indian samples on the updated-IGC as compared to 63% (±4.61% standard deviation) on IGC, showing a significant (False Discovery Rate (FDR) adj. *P* value = 10^−16^; Student *t*test) increase in mapping of Indian microbial dataset (Fig. 1A and Additional File 4). The datasets from populations in the United States (HMP), Denmark (MetaHIT), and China (a study from Qin et al.) mentioned in Table 1 were used for a comparative analysis of the microbiome of the Indian population with other populations [7, 10, 25]. The mapping of reads from these three datasets (HMP, MetaHIT, and China) on updated-IGC (mean mapping coverage: HMP = 67.74%, MetaHIT = 75.21%, and China = 77.44%) did not show a significant (*P* values: HMP = 0.5, MetaHIT = 0.85, and China = 0.17; Student *t*test) increment from their mapping coverage on IGC (mean mapping coverage: HMP = 66.93%, MetaHIT = 75.02%, and China = 77.37%) as observed in Fig. 1A. This shows that the addition of a subset of non-redundant genes (sharing <90% identity with IGC) from the Indian gut microbiome to the IGC significantly increased (FDR adj. *P* value = 10^−16^; Student *t*test) the mapping percentage of reads from the Indian microbiome dataset as compared to the other datasets.

**Table 1:** Metagenomic datasets used for comparative analysis (meta-analysis) of the microbiome and a metagenome-wide association study

Dataset	No. of samples	Sequence data (GB)	No. of genes
INDIA	110	110	4,809,378
USA	74	441	6,521,885
DENMARK	85	103.87	7,141,214
CHINA	71	180.78	5,464,702

### Identification of taxonomic signatures of Indian gut microbiome

To determine the taxonomic and functional composition of the Indian gut microbiome and to identify Indian-specific gut-microbial signatures, a cross-population comparison was carried out using the 16S rRNA gene hypervariable region and shotgun metagenomic data from the other populations. A reference-independent metagenome-wide association study (MGWAS) was carried out to identify the Indian-specific gut metagenomic markers through a comparison with similar large-scale studies from other populations [26]. The genes from the metagenomic samples of four countries (India, China, the United States, and Denmark) were clustered (see Methods section) into 924 clusters based on their co-occurrence and Pearson correlations (ρ ≥ 0.9) across samples, resulting in 335 metagenomic species (MGS) having ≥700 genes in each cluster, and 589 co-abundance gene groups (CAGs) consisting of ≥100 genes in each cluster. Out of the 924 metagenomic clusters, 195 could be assigned up to the species level using the taxonomic assignment strategy described in the Methods section. Canberra distances were calculated from MGS/CAG abundance profiles and their principal component analysis (PCA) was carried out using “countries” as factors for explaining the variance between samples, which showed that the Indian population formed a distinct cluster from the other populations in PCA (Fig. 1B). It is interesting to note that the samples from the Indian cohort were more widely spread owing to the higher inter-sample Canberra distances between Indian samples (mean = 0.689) as compared to other datasets having average inter-sample distances of 0.61, 0.59, and 0.54 for US, China, and Denmark populations, respectively (Additional File 5: Supplementary Fig. S1). This could be attributed to the significant (FDR adj. *P* value = 0.00013) differences in MGS abundance profiles between LOC1 and LOC2 populations as revealed on comparison of their principal coordinates (Additional File 5: Supplementary Fig. S2).

Further, the identification of enriched MGS from *P* values calculated using negative binomial (NB) model-based Wald test (implemented in DESeq2) and log odds ratio showed that the species belonging to the genera *Bacteroides, Alistipes, Clostridium*, and *Ruminococcus* were depleted in the Indian population (China, Denmark, and United States; log odds ratio < −2 and adj. *P* value < 0.01), whereas the MGS/CAGs annotated as *Prevotella, Mitsuokella, Dialister, Megasphaera*, and *Lactobacillus* were found to be associated with the Indian population (adj. *P* value < 0.01; log odds ratio > 2) and were the major drivers for separation of Indian samples from other populations (Additional File 5: Supplementary Fig. S3; Additional File 6). Furthermore, the distribution of microbial families across 10 different populations was also calculated using 16S rRNA gene markers, which revealed the Indian gut microbiome to have the highest abundance of Prevotellaceae (Fig. 1C). The feature selection method applied using random forest along with pairwise Wilcoxon rank-sum test also identified Prevotellaceae to be significantly higher (FDR adj. *P* < 0.05) in gut microbiome of Indian cohort compared to the other population datasets except Indonesia (*P* value = 0.506) (Additional File 5; Supplementary Figs. S4, S5, and S6) where a comparable abundance of Prevotellaceae was present. The high abundance of Prevotellaceae in the Indian population underscores its importance as the marker taxa for the Indian cohort.

### Microbial functions enriched in the Indian population

Functional comparison of Indian microbiome with other populations was carried out by mapping the genes derived from assembled contigs to the EggNOG database. In total 69,386 EggNOG functions were identified from the Indian gut microbiome, including 2,328 novel functions obtained from clustering the unmapped genes (see Methods section). The core microbial functions that are essential for microbial survival and present in almost 80% individuals were used for the functional comparison. The core microbiome was derived using a similar strategy as employed in MetaHIT (see Methods section) [25]. A set of 1,890 essential genes from six bacterial species, namely, *Escherichia coli* MG1655I and MG165II, *Bacteroides thetaiotaomicron* VPI-5482, *Pseudomonas* PA01, *Salmonella enteric* serovar *Typhi*, and *Staphylococcus aureus* NCTC 8325, were obtained and were assigned with eggNOG annotations. The eggNOG abundance profile generated from relative abundances of genes observed in Indian and other population datasets were ranked based on their mean abundance in descending order. The range of eggNOGs that included 85% of the 1,890 essential genes were considered as a part of the core microbial eggNOG set for each population dataset and was used for the analysis. Most of the essential genes were included in the top-ranking clusters, suggesting that the essential genes are present in higher abundance than the accessory function genes (Additional File 5: Supplementary Fig. S7).

The core microbiome of Indian samples was compared with the core microbiome of US, China, and Denmark populations. The proportion of essential genes covered by top-ranking eggNOG clusters showed that 85% of the essential genes could be covered in the least number (15,300) of eggNOGs in the case of Indian population, whereas it was covered by a higher number of eggNOGs in the case of US (20,400), China (19,900), and Denmark (30,900) populations (Additional File 5: Supplementary Fig. S8). These observations suggest that the core functional microbiome of Indian population is less diverse than the other populations. This corroborates well with the alpha diversity (mean Shannon index) calculated using gene abundance tables rarefied at 1,000,000 seqs/sample (for n = 30 random iterations), which also showed that the Indian microbiome is significantly (*P* value < 10^−16^) less diverse than the microbiome of the other populations analyzed in this study (Additional File 5: Supplementary Fig. S9).

**Figure 2:**
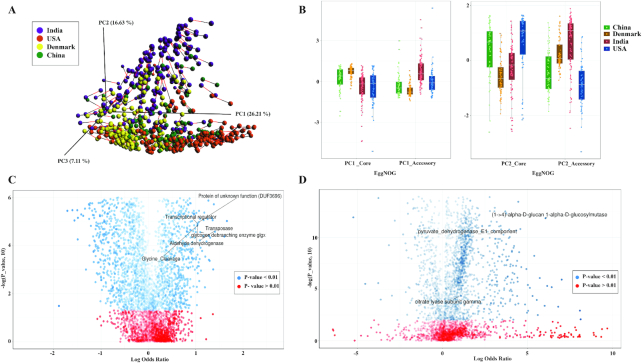
Functional variations and differences between Indian populations and other populations determined from core and accessory microbial functions. **(A)** Procrustes analysis was performed on Bray Curtis distances calculated from core EggNOG and accessory EggNOG abundance tables in all populations. PCA analysis shows the concordance of core and accessory functions in India, Denmark, US, and China populations. The red and black lines are associated with core and accessory datasets, respectively. **(B)** Eigenvalues calculated from PCA of samples using core EggNOGs and accessory EggNOGs are plotted. The box plots showing for core and accessory eigenvalues for all samples in different populations are shown. Each box plot represents the median shown as white line between the boxes, the upper and lower ends of the boxes representing upper quartile (75th percentile) and lower quartile (25th percentile). The whiskers extending on both ends represent 2.5* interquartile range. The different colored dots overlaid for each sample are plotted over the box. The enrichment or depletion of **(C)** Eggnog and **(D)** Kegg functions in India compared to other populations are shown as volcano plots. The log-transformed FDR adj. *P* values calculated from negative binomial-based Wald test from DESeq2 are plotted on the *x*-axis. The log odds ratio calculated for India vs Other datasets are plotted on the *y*-axis. The EggNOGs/KOs with *P* value < 0.05 are shown in blue whereas those having *P* values>0.05 are shown in red. The EggNOGs/KOs extending on the right and left side and with *P* value>0.05 are labeled as highly enriched in India and other datasets, respectively.

In total, 5,588 eggNOGs were characterized as core functions commonly present in the core microbiome of all the four population datasets. The co-inertia (Procrustes) analysis and the eigenvalues calculated from PCA using both core and accessory functions also showed that the Indian gut microbiome was significantly (FDR adj. *P* value = 6.4 × 10^−10^, 2 × 10^−16^ and 0.05 with China, Denmark, and United States, respectively, for PC1) different from the other datasets (Fig. 2A and 2B). These results also show the uniqueness of Indian gut microbial functions in composition and diversity at both core and accessory levels. The Indian gut microbiome was found to be enriched (FDR adj. *P* < 0.05, log odds ratio >1.5) in functions for carbohydrate and energy metabolism, including degradation of complex polysaccharides and glycogen, and was also enriched for enzymes from the TCA cycle, which corroborates well with the carbohydrate-rich diet of the Indian population (Fig. 2C and 2D and Additional File 7: enriched KO and EggNOG functions).

### Unsupervised clustering of Indian samples and their association with previously identified enterotypes

**Figure 3:**
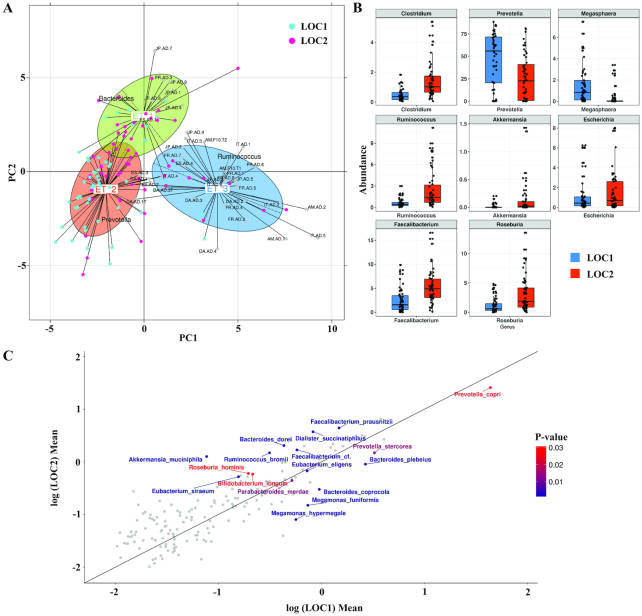
Variations in gut microbiome at the two locations. **(A)** Between-class analysis, which visualizes results from PCA and clustering, using genus-level abundance from 37 cross-national datasets and genus abundance of 110 Indian samples obtained from mapping of reads to reference genomes. The samples from LOC1 (cyan), LOC2 (pink), and 37 cross-national samples from Arumugam et al. (gray and labeled) are placed into three distinct enterotypes based on clustering. **(B)** Significantly different genera (FDR adj. *P* value < 0.05; NB model-based Wald test) between the two locations are shown as box plots with boxes representing interquartile range, dark lines between the boxes representing median values, and whiskers representing the 1.5 x IQR on each side. **(C)** Scatterplot of log-transformed mean values of species abundance in LOC1 (n = 53) and LOC2 (n = 57) individuals. Red color gradient points represent differentially abundant (FDR adj. *P* < 0.05; NB model-based Wald test) species with lower *P* values from red to blue.

Arumugam et al. classified the samples from multiple populations into clusters based on genus-level profiles and identified three prominent clusters called enterotypes [2]. In order to identify the enterotypes from Indian gut microbiome, a meta-analysis was performed using genus-level abundances of samples from the four nations as used by Arumugam et al. along with the Indian cohort. There were three prominent clusters observed with the majority (63.6%) of Indian population falling into enterotype-2, which was primarily driven by *Prevotella*. The analysis revealed differences in the distribution of samples from LOC1 and LOC2, where a higher number of samples from LOC1 (73.5%) was associated with enterotype-2 compared to LOC2 (54%). In contrast, LOC2 samples were associated with enterotype-1 (30.3%) and enterotype-3 (16.07%), which were driven by *Bacteroides* and *Ruminococcus*, respectively (Fig. 3A; Additional File 8).

An independent microbial abundance-based clustering of Indian samples using Jensen Shannon distances revealed two prominent clusters. The clustering was validated using the Calinski Harabasz index (CHI) and prediction strength, which uses a cross-validation approach to validate the robustness of clustering (Additional File 9). Cluster 1 was primarily enriched in species from genus *Prevotella* (*P* < 10^−10^), and Cluster 2 was quite widely spread and was enriched in species belonging to *Bifidobacterium* (*P* = 10^−13^), *Ruminococcus* (*P* = 0.031), *Clostridium* (*P* = 0.04), and *Faecalibacterium* (*P* = 0.046) (Additional File 5: Supplementary Fig. S10, Additional File 10). The higher abundances of *Prevotella* in LOC1 and *Bacteroides* in LOC2 in India are perhaps due to the different dietary habits at the two locations. The LOC1 population was mainly consuming a carbohydrate-rich diet comprising vegetable-based foods and grains, whereas the LOC2 population was consuming a diet consisting of rice, meat, and fish. Similar variations in microbiome diversity due to differences in dietary habits have also been observed in earlier studies [27, 28]. However, to confirm the above observations and to assess the quantitative effect of dietary habits on microbial variations, further longitudinal studies are necessary where detailed dietary information needs to be collected through a food-frequency questionnaire.

A similar cluster analysis performed using functional information derived from the abundance of Kyoto Encyclopedia of Genes and Genomes (KEGG) Orthologs (KO) also showed the clustering of samples into two distinct clusters, namely, C1 and C2 (Additional File 5: Supplementary Fig. S11). In comparison to clusters derived from taxonomic information, only 14 out of 110 samples were placed in different clusters using the functional information showing a similarity (*P* value = 0.6841; Fisher exact test; Additional File 11) in cluster allocation using both taxonomic and functional information. C1 was found enriched in genes coding for enzymes such as β- glucosidase (Log Odds Ratio (LOR) = 3.364; *P* value = 10^−20^), and α-fucosidase (LOR = 0.73; *P* = 10^−8^), which are involved in the breakdown of plant-polysaccharides, whereas the genes coding for enzymes such as lipase (LOR = −1.34; *P* = 10^−12^), carnitine-coA dehydratase (LOR = −1.81; *P* value = 0.029), and amino peptidase (LOR = −2.72; *P* = 10^−10^), which are involved in the metabolism of an animal-based diet, were enriched in C2 (FDR adj. *P* < 0.05) (Additional File 12).

To identify the covariates explaining the maximum variations in microbial profiles across samples, unweighted UniFrac distances were calculated using phylogenetic distances between operational taxonomic unit (OTU) reference sequences and an OTU table rarefied at 100,000 seqs/sample. The principal component analysis of UniFrac distances and the correlation of loadings for each sample with the covariates using polyserial/biserial correlation identified distinct locations (LOC1 and LOC2) and diet (vegetarian and omnivorous) to be the major covariates explaining the variation in taxonomic diversity between the samples (Additional File 5: Supplementary Fig. S12, Additional File 13). An ordination of 110 Indian samples using gene abundance profiles from metagenomic data showed location and diet to be significantly (FDR adj. *P* value < 0.01; polyserial correlation) associated with PC1, explaining the maximum variation between samples (Additional File 5: Supplementary Fig. S13, Additional File 13). A significant correlation (ρ = 0.708; *P* value = 2 × 10^−16^ Spearman rank correlation) was also observed between location and diet covariates. A comparison of functional diversity using gene abundance curves with increasing number of samples performed between the two locations showed that the microbiome profiles of LOC2 populations were more diverse in their composition compared to LOC1 populations (Additional File 5: Supplementary Fig. S14). The inter-individual Bray-Curtis distances calculated on normalized gene abundance profiles between LOC1 and LOC2 populations also showed significant differences (FDR adj. *P* < 0.05), where LOC2 population displayed higher inter-individual heterogeneity in their microbial community structure as compared to LOC1 population (Additional File5: Supplementary Fig. S15).

Major differences in the microbiome profiles were apparent at the Phylum level (using 16S rRNA gene amplicon sequencing) from the higher Bacteroidetes to Firmicutes ratio (*P* = 0.002) in LOC1 (1.93) compared to LOC2 (0.86), which have been previously reported as a result of differences in dietary habits, i.e., vegetarian or plant-based (carbohydrate-rich) vs omnivore or animal-based (protein-rich) diets (Additional File 5: Supplementary Fig. S16) [29, 30]. Notably, these variations were not attributable to BMI (Spearman rank correlation, FDR adj. *P* = 0.78). Taxonomic profiles generated from metagenomic datasets through reads mapped to reference genomes were compared between the two locations at genus and species levels using NB model-based Wald test implemented in DESeq2. *Prevotella*and*Megasphaera* were observed to be higher in LOC1, whereas *Ruminococcus* and *Faecalibacterium* were higher in LOC2 (FDR adj. *P* < 0.05, Wilcoxon rank-sum test) (Fig. 3B). Within these genera, *P. copri and P. stercorea* species were significantly higher in LOC1, whereas *F. prausnitzii* and *R. bromii* belonging to genus *Faecalibacterium* and *Ruminococcus*, respectively, were higher in LOC2. In addition, *Akkermansia muciniphila, Eubacterium siraeum*, and *Roseburia hominis* were observed to be higher in LOC2, and *M. funiformis* and *M. hypermegale* from genus *Megamonas* were higher in LOC1 (Fig. 3C). Moreover, the metagenomic species derived from clustering of gene profiles showed that MGS/CAGs were enriched in LOC1 (log odds ratio >2; adj. *P* < 0.05), of which seven MGS/CAGs were annotated to *Prevotella copri*. Similarly, 67 MGS/CAGs were found enriched in LOC2 (adj. *P* < 0.05; log odds ratio < −2) and included 8 and 3 MGS/CAGs annotated to SCFA-producing species *Faecalibacterium prausnitzii* and *Roseburia inulinivorans*, respectively (Additional File 14). Interestingly, both, *F. prausnitzii* and *R. inulinivorans* species enriched in LOC2 are known SCFA producers and are regarded as commensals with anti-inflammatory properties [31]. In contrast, *Prevotella*, which was abundant in the LOC1, is known to be associated with a fiber-rich diet [32].

### Defining the Indian gut metabolome

**Figure 4:**
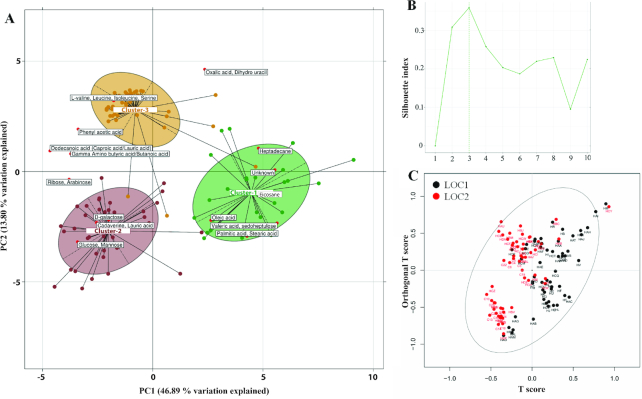
Between-class analysis to identify metabotypes and their associated metabolites. **(A)** Metabolite clusters (MES) abundance profiles of samples were generated, and their clustering was performed using PAM (partitioning around medoids) clustering. The between-class and PCA of JSD distances and PAM clustering identified three clusters to be optimum for their segregation using **(B)** Silhouette index. The metabolites valeric acids and saturated fatty acids such as palmitic acid and stearic acid were found to be higher in Cluster1. The carbohydrates such as glucose and galactose were found to be higher in Cluster 2. The BCAAs, lauric acid, and butyric acid were found to be higher in Cluster 3. **(C)** OPLS-DA analysis using locations as classes shows locations as differentiating factors in separating the samples based on their metabolomic profiles.

The analysis of microbial community structure and functions from the two locations having different lifestyle and diet revealed significant insights. Previous studies have shown a direct role of diet in shaping the different gut microbiomes [33]. Thus, to gain deeper insights into the metabolic activity of microbiomes from LOC1 and LOC2 as driven by different diets, fecal metabolites were analyzed using a GC-MS-based metabolomics approach. An unsupervised between-class analysis of metabolomic profiles separated the samples into three separate clusters, and the robustness was confirmed using prediction strength and Silhouette index (Fig. 4A and 4B). Polyserial correlation of covariates showed location to be the major factor explaining the variation at PC1 (FDR adj. *P* < 0.01) separating Cluster 1 from Clusters 2 and 3. In contrast, vegetarian and omnivorous diet groups emerged as other factors explaining the variation at PC2 (FDR adj. *P* < 0.01) and separating Cluster 2 from Cluster 3 (Additional File 15). The covariates' location and diet were also observed to be highly correlated variables showing their strong influence on gut microbiome. Cluster 1 was associated with LOC1 and showed higher concentration of saturated fatty acids including palmitic acid, stearic acid, and valeric acid. Cluster 3 was associated with LOC2 and showed higher abundances of BCAAs, valine, leucine, and isoleucine, and SCFAs, propionate, and butyrate concentrations. Cluster 2 was enriched in D-glucose, galactose, mannose, lauric acid, and cadaverine (a polyamine associated with meat consumption) and was also observed to be associated with LOC2 [34]. To assess the effect of different covariates on the separation of samples, PERMANOVA was performed (Table 2). The location was found to explain maximum variation for separation of samples, whereas diet was the second most important variable in explaining the variance. The Orthogonal Projections to Latent Structures Discriminant Analysis (OPLS-DA) model was used to expose the class separation for each of the covariates using Q^2^ values that assessed the quality measurement (Table 3). The OPLS-DA models validated by random permutation (n = 200) of class labels showed Q^2^ values for location and diet to be higher than Q^2^ values produced from random permutations with location showing highest Q^2^ values (Additional File 5: Supplementary Fig. S17). The OPLS-DA model also showed clear separation of samples between locations as class of separation (Fig. 4C).

**Table 2:** PERMANOVA to assess the effect of covariates on metabolomics profiles of samples

Variable	Sum of Sq	Mean Sq	F-Model	R^2^	*P* value
Location	0.05841	0.058406	4.9423	0.04455	0.0009
Diet	0.04701	0.04701	4.2132	0.03586	0.0009
Age	0.01618	0.01618	1.4505	0.0123	0.161
Gender	0.00488	0.00488	0.4370	0.00373	0.927

**Table 3:** OPLS-DA model and its validation for different covariates as class of separation

Variable	R^2^X	Q^2^ (cumulative)	pR^2^	pQ^2^
Location	**0.165**	**0.205**	0.005***	0.005***
Diet	0.168	0.123	0.005***	0.005***
Age	0.155	−0.00067	0.075	0.065
Gender	0.106	−0.247	0.145	0.96
Cluster (genus based)	0.16	0.15	0.005***	0.005***

pR^2^ and pQ^2^ show *P* values for validation of OPLS-DA model with *P* value <0.01 shown as significant (*).

### Positive correlation of BCAA transporters with BCAA levels in fecal metabolome

**Figure 5:**
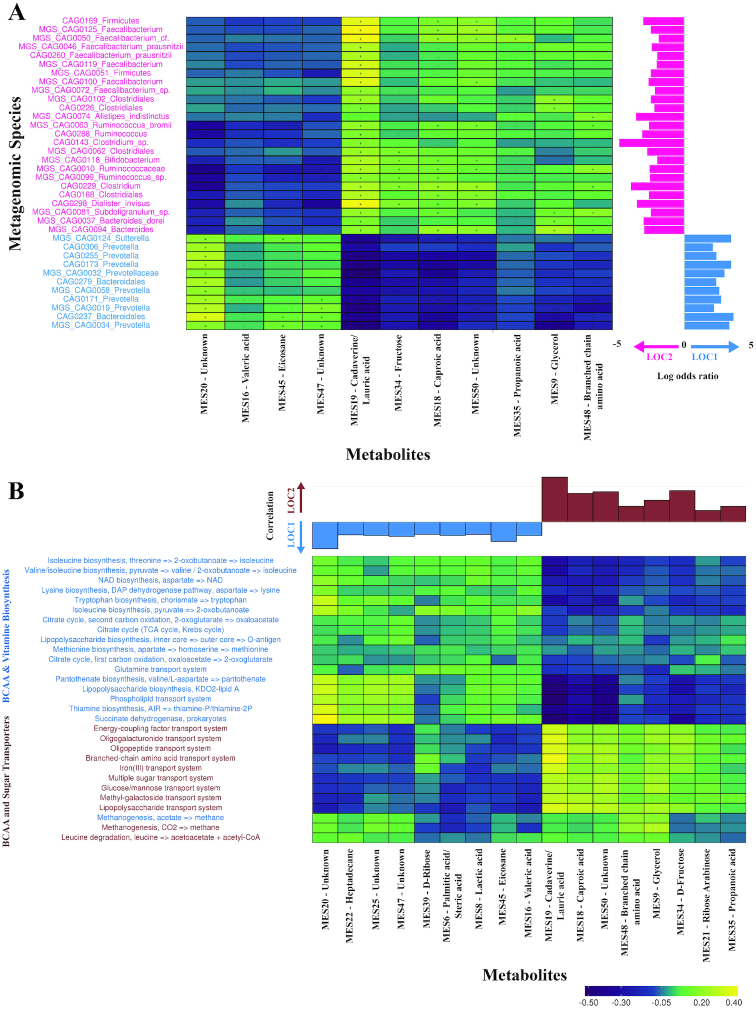
Spearman rank correlations of metabolites with species and metabolic modules. **(A)** Spearman rank correlation coefficients were calculated between significantly different metagenomic species and significantly different metabolites between LOC1 and LOC2 populations. The correlations showing significant FDR adj. *P* < 0.05 are plotted. The bars on the right show the log odds ratio of the abundance of MGS, with positive values indicating enrichment in LOC1 and the negative values indicating enrichment in LOC2. **(B)** Spearman rank correlations between significantly different (FDR adj. *P* < 0.05, NB model-based Wald test) pathway modules and significantly different metabolite abundances in all samples. The significant (*P* < 0.05) correlations are plotted, and the color intensities depict the correlation coefficients. The correlation of metabolites with locations is shown with labels in dark red colors showing association with LOC, and the labels in green colors showing correlation with LOC1.

We also identified the marker metabolites, which showed significant (Spearman correlation, FDR adj. *P* < 0.05) associations with LOC1 or LOC2. In total, 17 metabolite clusters were identified, of which 9 were associated with LOC1 and 8 were associated with LOC2 (Additional File 16). These marker metabolites showed a positive association with MGS/CAGs. For instance, *Prevotella* annotated clusters correlated significantly with valeric acid and sedoheptulose metabolite markers, which showed a higher relative abundance in LOC1. In contrast, MGS/CAGs belonging to *Faecalibacterium, Clostridium, Ruminococcus*, and *Alistipes* were positively associated with BCAAs, cadaverine, propanoate, and lauric acid in LOC2 (Fig. 5A). In addition to the positive association of BCAAs with species enriched in LOC2, a correlation analysis of significantly different (FDR adj. *P* < 0.05, DESeq2-based Wald test; Additional File 17) functional modules revealed that fecal BCAA abundances were positively correlated with BCAA transporter abundance in LOC2. In contrast, BCAA abundance in the fecal metabolome showed a negative correlation (*P* < 0.05) with BCAA biosynthesis pathways (Fig. 5B).

**Figure 6:**
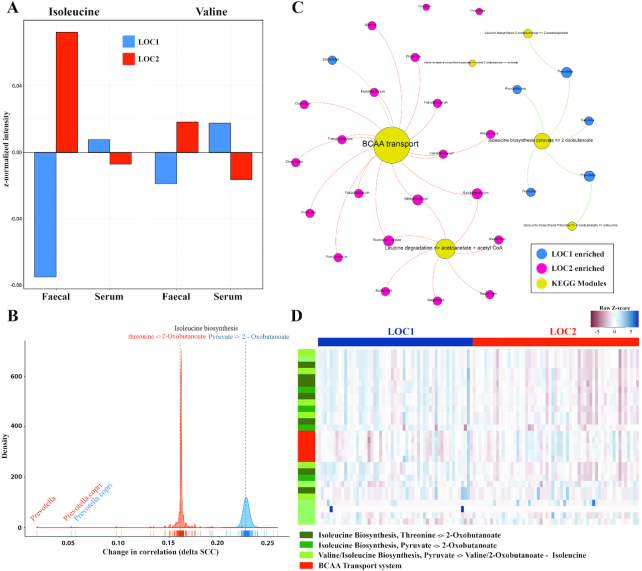
BCAA abundance and their differential correlation with LOC1 and LOC2. **(A)** Bar plot showing z-normalized values of serum and fecal BCAA (valine and isoleucine) relative concentration in LOC1 and LOC2. **(B)** The effect of specific microbial species on associations between BCAA biosynthesis pathways and BCAA levels in fecal metabolome, illustrated by change in background adjusted Spearman correlation coefficient when a given species has been excluded from analysis, is shown (see Methods). The density plot shows the distribution of correlation for species and the changes caused by specific species as marked by lines below. **(C)** Network analysis of Spearman correlations between the branched chain amino acids biosynthesis, degradation, and transport KEGG modules with MGS abundance in both LOC1 and LOC2 populations. The node size is proportional to the degree of interactions, and the links between module and MGS show interactions or significant correlations (FDR adj. *P* < 0.05) with negative (in red) and positive (in blue) correlation coefficients.  **(D)** Plot showing relative abundance of KOs associated with different modules of BCAA biosynthesis and transporters in LOC1 and LOC2.

The above observations are significant given that BCAAs are important metabolites involved in glucose homeostasis by stimulating insulin secretion [35]. Higher BCAA levels in the fecal samples could be a result of its uptake by microbes via BCAA transporters, leading to their accumulation in the microbial cells detected in fecal metabolome. This is concordant with higher relative abundance of *Bacteroides vulgatus* and *Eubacterium sireaeum* in LOC2 compared to LOC1, which are known to harbor higher abundance of BCAA transporters (Fig. 3C) [8]. Further support for this hypothesis emerged from the correlation of circulating BCAA levels (valine and isoleucine) in serum with the corresponding concentrations in feces. Interestingly, serum BCAA concentrations were significantly higher in LOC1 individuals as compared to LOC2 individuals, which is in contrast with their BCAA levels in the fecal metabolome (Fig. 6A). Thus, one possibility is that the accumulation of BCAA in the feces of individuals of LOC2 was mediated by the inward transport of BCAA by the gut bacteria. In contrast, the lower BCAA accumulation in gut microbes and a higher BCAA biosynthesis by microbial species and its eventual absorption in serum appears to be a plausible reason for the higher BCAA concentrations in serum of LOC1 populations.

### Role of *Prevotella copri* in the regulation of BCAA levels

To explore the differences in association of functional pathway modules between the two locations, KOs within each module were correlated with KOs from other modules using Spearman correlation coefficient. The KOs showing significant differences in correlations between LOC1 and LOC2 were identified. This differential correlation analysis of BCAA biosynthetic modules with other pathways in LOC1 and LOC2 revealed that BCAA modules were independently driven in LOC1 and LOC2 (Spearman rank correlation, FDR adj. *P* < 0.01) (Additional File 5: Supplementary Fig. S18A and S18B). To identify the species and the metabolic pathways that contributed most to the BCAA abundance in fecal and serum metabolome profiles, a correlation analysis with iterations leaving each species out was performed for each metabolic module (Fig. 6B). The species whose removal leads to a maximum change in the correlation of metabolic pathway with metabolite was identified and was considered as an important contributor of that metabolite.

Notably, the BCAA biosynthesis-dependent changes in BCAA levels were largely driven by *Prevotella* species through threonine-dependent and -independent biosynthesis pathways as observed from Delta SCC_bg_ values when genes from this species were removed (see Methods section). The correlation network analysis with differential MGS/CAGs revealed threonine-independent isoleucine biosynthesis pathway to be highly correlated with *Prevotella copri* in LOC1 (Fig. 6C). The first enzyme, D-citramalate synthase, catalyzing the first step of threonine-independent isoleucine biosynthesis pathway was also observed as highly enriched (LOR = 1.7) in LOC1 [36]. Further, BCAA biosynthesis pathways were found to be higher in LOC1, whereas BCAA transporters were higher in LOC2 (Fig. 6D), leading to the dynamic changes in BCAA concentrations in fecal and serum metabolome in LOC1 and LOC2 as observed in Fig. 6A.

## Discussion

Compositional and functional human gut microbiome studies in different populations have been instrumental in establishing the role of gut microbiome in human health [2, 28, 37, 38]. However, such population-specific signatures and their functional roles are yet unknown for the Indian gut microbiome. This study provides the first insights into the Indian gut microbiome represented through a cohort of 110 individuals from two prominent locations to reveal the taxonomic and functional diversity using 16S rRNA gene, metagenomic analysis, and metabolomic profiling. Although the sequencing depth was modest (1.36 ± 0.5 Gbp per sample, mean ± standard deviation), the inclusion of 110 individuals from two distinct geographic locations as well as the identification of Indian gut microbiome-specific genes provide a first insight into the Indian gut microbiome and are thus considered important additions to the field. The selection of two distinct sub-populations (Bhopal-LOC1 and Kerala-LOC2) was an important consideration to capture the microbiome variations resulting from different diets and lifestyles of these two cohorts. LOC1 provided a representation of the population from North-Central India mainly consuming a carbohydrate and fat-rich diet, whereas LOC2 represented a population from Southern India consuming an omnivorous diet with rice and animal-based products as the primary components.

This study established the gene catalogue of the Indian gut microbiome, which provides the first insights into the yet unknown functional gut microbiome of the Indian population. The genes encoding several transposons, peptidase, glucosidase, and plant polysaccharide degradation enzymes were unique to the Indian population and not represented in other microbiome datasets. The Updated-IGC (IGC+India) constructed by the addition of unique non-redundant genes from the Indian population to the Integrated gene catalogue is likely to act as a reference dataset for gut microbiome studies for global comparative studies and particularly for studies involving South-Asian populations that have similar dietary habits and lifestyle.

In addition to the basic housekeeping functions of the gut microbiome, which were also found abundant in other datasets, the Indian gut microbiome was enriched in functions for carbohydrate and energy metabolism including degradation of complex polysaccharides, which corroborates well with the typical carbohydrate-rich diet of the Indian population [39]. The distant clustering of Indian samples from other populations revealed the unique composition of the Indian gut microbiota (Fig. 1B). *Prevotella* emerged as the most discriminatory genus associated with the Indian population as revealed by both amplicon and MGWAS. The abundance of *Prevotella* was also indicated in the previous 16S rRNA gene-based microbiome studies of the Indian population carried out in small to medium-sized cohorts [18, 19]. Recently, *Prevotella* has been commonly observed in different non-Western communities that consume a plant-rich diet, such as in the Papua New Guineans, native Africans, rural Malawians, and BaAka pygmies [11, 40] and has also been associated with vegetarianism in the Western populations [41, 42]. However, it has not been observed at such high abundance in the Western countries so far. The MGWAS approach in this study showed the presence of *Megasphaera, Lactobacillus*, and *Mitsuokella* as the other major genera associated with the Indian gut microbiome.

Several recent studies have shown a relationship between the abundance of specific strains of *Prevotella* with inflammatory diseases, since it has a higher intrinsic capacity to stimulate Th17-mediated inflammation, which is generally not expected in a strict commensal bacterium [41, 43, 44]. However, the high abundance of *Prevotella* in the healthy gut microbiome of the Indian population does not corroborate with its potential inflammatory role reported so far. Since this study was only focused on the gut microbiome of healthy individuals, it is difficult to draw conclusions on the potential inflammatory role of this species. One potential explanation could be the complex set of interactions between host genetic risk factors and environment in which the presence of *Prevotella* may be only one of the factors [45]. Further, strain-level variations are known in the inflammatory responses and not all species of *Prevotella* could be potentially inflammatory, as also evident from the known high genetic diversity within and between the species of *Prevotella* [43]. Thus, the high abundance of *Prevotella* in the healthy microbiota emphasizes the requirement for larger cohort studies in different populations to gain deeper insights into the potential inflammatory roles of gut microbes.

The abundance of *Prevotella* has been associated with plant-based diets, and the typical carbohydrate-rich diet of the Indian population could be one of the reasons for the over-representation of this genus in the Indian gut microbiome [46]. Likewise, the predominance of other microbial species from genus *Lactobacillus, Megasphaera*, and *Mitsuokella* could be due to the higher intake of fermented food and dairy products along with the carbohydrate-rich diet in LOC1 [46, 47]. Similarly, *Bacteroides* and *Clostridium*, which were abundant in LOC2, are associated with diets rich in animal-based products, consistent with the omnivorous diet of LOC2 [42]. Interestingly, taxonomy-based clusters 1 and 2 showed associations with the two locations LOC1 and LOC2 and also with the two KO-based clusters (C1 and C2) (Additional File 5: Supplementary Figs. S10 and S11). It is to be noted that C1 was enriched in enzymes involved in the degradation of carbohydrate and plant polysaccharides, which correlates well with the carbohydrate-rich diet in LOC1. In contrast, C2 was enriched in enzymes involved in lipid and protein degradation, which relate to the constituents of an omnivorous diet in LOC2. These observations further support the correlations between location, diet and enterotype. Although, the concept of enterotype classification is sometimes criticized due to statistical weakness in some studies, a meta-analysis of Indian samples with samples from Arumugam et al. revealed three robust clusters, with Indian samples mostly associated with enterotype-2 driven by *Prevotella* [2]. This classification of samples from multiple population/studies into enterotypes has the potential to be clinically relevant in various aspects such as disease diagnosis, early detection of disease, biomarker development, personalized treatments, and xenobiotic metabolism [48]. It is a representation of the major microbial species in the gut microbiome and thus appears useful for microbiome-based population stratification. A robust statistical analysis with increased sample sizes, direct clinical associations, and detailed molecular interventions are essential for further strengthening its potential.

The study also established the previously unknown fecal metabolome of the Indian population, which showed strong clustering into three metabolomic clusters differentiated by location and diet. The metabolomic clusters also correlated well with the respective dietary habits of the two locations, where metabolomic Cluster 1 showed an association with LOC1 and was enriched in saturated fatty acids such as palmitic acid and stearic acid, whereas metabolomic Cluster 3 showed an association with LOC2 and was enriched in BCAAs such as isoleucine, valine, and leucine and in SCFAs such as propionic acid and butyric acid. A medium chain fatty acid “lauric acid” was also found abundant in LOC2 perhaps due to the high dietary consumption of coconut oil in this location [49, 50]. Lauric acid has been reported to be beneficial by preventing fat deposition in blood vessels and acting as an anti-inflammatory and anti-oxidative agent [51].

**Figure 7:**
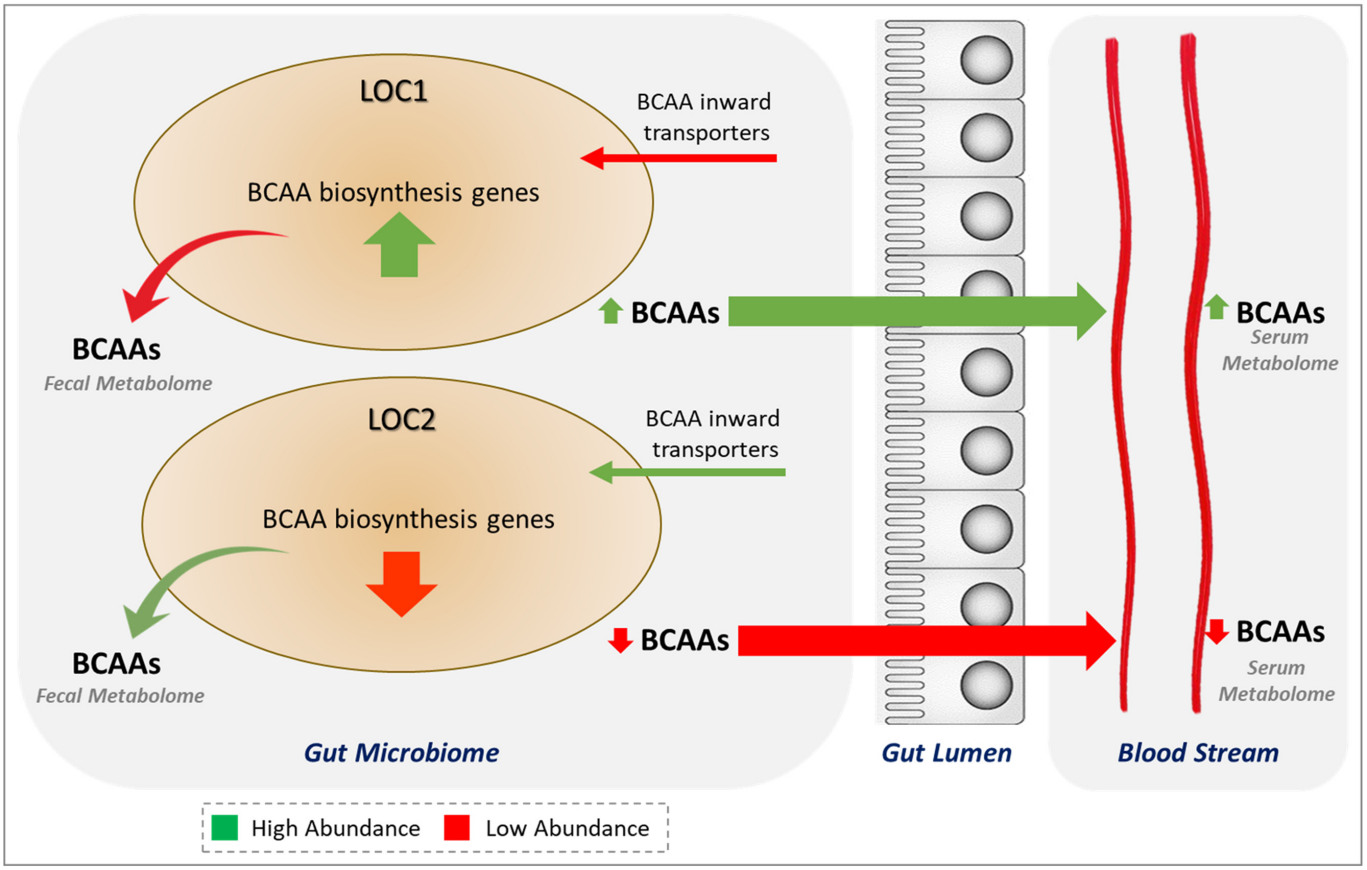
BCAA transporters playing a key role in maintaining the levels of BCAAs in feces and serum. The dynamics of BCAA concentration levels in fecal and serum metabolome influenced by microbial BCAA biosynthesis and transport pathways and their differential abundance in LOC1 and LOC2 is shown.

The major BCAA “isoleucine” being produced through a less common threonine-independent pathway for isoleucine biosynthesis, and the higher enrichment of the key enzyme, D-citramalate synthase of the above pathway confirmed its higher abundance in LOC1 as compared to LOC2. Further, this pathway was found to be associated with a single species, *Prevotella copri*, as reported earlier [8]. Taken together, it appears that the higher abundance of BCAA biosynthesis genes and a lower abundance of BCAA inward transporters in gut microbiome resulted in the lower BCAA accumulation in the fecal metabolome and higher BCAA concentration in serum as observed in LOC1 (Fig. 7) [8]. However, a contrasting pattern was observed in the case of LOC2, where the lower abundance of BCAA biosynthesis genes and the higher abundance of BCAA inward transporters correlated well with the higher and lower BCAA concentrations in feces and serum, respectively.

The higher levels of SCFAs in LOC2 could be a consequence of the consumption of an omnivorous diet, which is associated with a Firmicutes-rich gut microbiome [52]. SCFAs have well-established roles in human health as an energy source, an anti-inflammatory agent, and for improving intestinal homeostasis by increasing IL-18 production [53]. In contrast, higher serum BCAA levels have well-known roles in promoting insulin resistance and type-2 diabetes (T2D) and were found to be higher in the serum in LOC1. Several reports on the role of a high-fat diet in the modulation of microbiota and alteration in intestinal barrier are emerging, with results showing increased absorption and circulating levels of BCAA and reduction of SCFAs such as butyrate, acetate, propionate, and secondary bile acids, as also noted in the case of LOC1 [54, 55]. High-fat and carbohydrate-rich diets have also been associated with an increase in abundance of Bacteroidetes (gram-negative bacteria) leading to a skewed Bacteroidetes:Firmicutes ratio towards the former phylum [32]. Such a ratio was also apparent in this study in LOC1 dominated by *Prevotella* from the phylum Bacteroidetes. Further, higher serum concentrations of circulating BCAA were also observed in LOC1. These results provide hints on the role of dietary habits in shaping the gut microbiome and its plausible impact on the BCAA and SCFA dynamics observed in these populations.

To conclude, this multi-omics-based gut microbiome study of a healthy cohort from two different parts of India provides novel insights into the Indian gut microbiome and metabolome and reveals the unique gene catalogue from the poorly characterized Indian population. Further studies using higher sequencing depths and including both healthy and diseased individuals will help in obtaining more comprehensive functional and taxonomic information of gut microbiome from Indian population and its impact on human health.

## Methods

### Study design and subject enrollment

The study cohort consisted of 110 healthy individuals belonging to different age groups from infants (<1 year) to aged (>50 years), with an average subject age of 29.72 ± 17.4 years (mean ± standard deviation) from two different locations across India, i.e., Bhopal (LOC1, n = 53) and Kerala (LOC2, n = 57), which are separated by ∼1000 miles. LOC1 is located in North-Central India with the majority of population being vegetarian, whereas LOC2 is located in Southern India where the population's dietary habits mostly consist of rice, seafood, and red meat (see Diet description section in Additional File 1). According to the Indian Food Composition Table, the primary Indian diet is rich in carbohydrates such as rice, wheat, and potato and in fat and proteins from milk and dairy products [56]. In addition, several accompaniments to the primary diet also exist, including a variety of grains, vegetables, fruits, and usage of oil, spices, and animal products.

The fecal samples for metagenomics and blood samples for serum metabolomics were collected from healthy participants, and their metadata is provided in Additional File 1 under the Metadata information section. The recruitment of volunteers, sample collection, and other study-related procedures were carried out by following the guidelines and protocols approved by the Institute Ethics Committee of the IISER, Bhopal, India. Each fecal sample was frozen within 30 minutes of collection. Written informed consent was obtained from all subjects prior to any study-related procedures, along with information on gender, age, and diet for a period of one month prior to the collection of fecal samples. The recruited individuals did not receive any medication at least one month prior to the sample collection. All the recruited individuals had an average BMI of 21.16 (±5.23), were not diagnosed with T2D at the time of sample collection, and did not have a second-degree relative history of T2D. The above samples were then used for 16S rRNA gene V3 hypervariable region amplicon sequencing, shotgun metagenomic sequencing, and metabolomic analysis.

### Fecal metagenomic DNA extraction

Metagenomic DNA was isolated from all the fecal samples using QIAamp Stool Mini Kit (Qiagen, CA) according to the manufacturer's instructions. DNA concentration was estimated by Qubit HS dsDNA assay kit (Invitrogen, CA), and quality was estimated by agarose gel electrophoresis. All the DNA samples were stored at −80°C until sequencing.

### 16S rRNA gene amplicon and shotgun metagenome sequencing

The extracted DNA (5 ng) was PCR amplified with seven different custom modified 5ʹ-end adaptor-ligated 341F and 534R primers (see the Primer Details section in Additional File 1) targeting the V3 hypervariable region of 16S rRNA gene. After evaluating the amplified products on 2% w/v agarose gel, the products were purified using Ampure XP kit (Beckman Coulter, Brea, CA). Amplicon libraries were prepared by following the Illumina 16S rRNA gene metagenomic library preparation guide. Metagenomic libraries were prepared using Illumina Nextera XT sample preparation kit (Illumina Inc.) by following the manufacturer's protocol. Library size of all the libraries was assessed using Agilent 2100 Bioanalyzer (Agilent Technologies, Santa Clara, CA) and quantified on a Qubit 2.0 fluorometer using Qubit dsDNA HS kit (Life technologies) and by qPCR using KAPA SYBR FAST qPCR Master mix and Illumina standards and primer premix (KAPA Biosystems, Wilmington, MA) following the Illumina suggested protocol. Both the amplicon and metagenomic libraries were loaded on Illumina NextSeq 500 platform using NextSeq 500/550 v2 sequencing reagent kit (Illumina Inc.), and 150 bp paired-end sequencing was performed at the Next-Generation Sequencing (NGS) Facility, IISER Bhopal, India.

### Amplicon-based taxonomic analysis

A total of 24 Gbp of data were retrieved on de-multiplexing of paired-end reads with an average of 210 Mbp per sample. The paired-end reads were assembled using FLASH and were quality filtered at Q20 (80% bases) Phred quality score using NGSQC Toolkit v 2.3.3 [57, 58]. The primer sequences were trimmed from the high-quality reads. The reads were further clustered into OTUs using closed-reference OTU picking protocol of QIIME at ≥97% identity against ARB SILVA database release 132 (13 December 2017) [59, 60]. The most abundant read was selected as the representative sequence for each OTU and was assigned with taxonomy using the SILVA database. An OTU table containing the abundance of each OTU for each sample was generated and used for further analysis. For phylogenetic analysis, representative 16S rRNA genes of phylotypes were aligned against a core set of 16S rRNA gene sequences using align_seqs.py with the PyNAST v.1.2.2 algorithm [61]. The unweighted UniFrac distances between samples were calculated using rarefied OTU abundance (100,000 seqs/sample) table and phylogenetic distances between representative sequences from each OTU [62].

### Pre-processing of the metagenomic reads

A total of 150 Gbp of metagenomic sequence data (mean = 1.36 Gb) was generated from 110 fecal samples. The metagenomic reads were filtered using NGSQC toolkit v2.3.3 with a cutoff ≥Q20 [57]. The high-quality reads were further filtered to remove the host-origin reads (human contamination) from metagenomic reads using 18mer matches parameter in Best Match Tagger BMTagger v3.101 (BMTagger, RRID:SCR_014619; [63]), which resulted in the removal of an average of 1% reads. The reads from each sample were assembled separately into contigs using IDBA ud version 1.1.0 [64] with parameters “-mink 31 –maxk 87 –step 5.” The reads from each sample were mapped to contigs to estimate read recruitment using FR-HIT version 0.7 [65]. The unmapped reads resulting from each sample were pooled together and *de novo* assembly was performed on the combined set of singleton (unmapped) reads from all samples. The open reading frames (ORFs) from each contig (length ≥ 500 bp) were predicted using MetaGeneMark v.3.38 [66]. Pair-wise alignment of genes was performed using BLAT version 2.7.6 [67], and the genes that had an identity ≥95% and alignment coverage ≥90% were clustered into a single set of non-redundant genes, from which the longest gene was selected as the representative ORF to construct the non-redundant gene catalog.

The IGC, which represents 1,297 human gut metagenomic samples comprising of HMP, MetaHIT, and Chinese datasets, was retrieved [24]. The gene catalogue constructed from Indian samples was combined with the IGC to construct a non-redundant gene catalog (using identity ≥95% and alignment coverage ≥90%) and is referred to as “Updated-IGC” in the subsequent analysis.

### Quantification of gene content

The quantification of gene content was carried out using the strategy performed by Qin et al., [7] where the high-quality reads were aligned against the updated IGC using SOAP2 in SOAP aligner version 2.21 with an identity cutoff ≥90% [68]. Two types of alignments were considered for sequence-based profiling:

1. The entire paired-end read mapped to the gene.
2. One end of paired-end read mapped to a gene and other end outside genic region.

In both cases, the mapped read was counted as one copy.

The relative abundance of a gene within the sample was calculated as: ${{\rm{a}}_i} = \frac{{{{\rm{b}}_i}}}{{\sum j{{\rm{b}}_j}}}$

a_i_: relative abundance of gene in sample S; x_i_: the times in which gene i was detected in sample S (the number of mapped reads); b_i_: copy number of gene i in sequenced data from sample S.

### Phylogenetic assignment of reads

A total of 4,097 reference microbial genomes were obtained from the Human Microbiome Project (HMP) and National Center for Biotechnology Information (NCBI) on 5 December 2015 (Additional File 18). The databases were independently indexed into two Bowtie indexes using Bowtie-2 version 2.2.9 (Bowtie 2, RRID:SCR_016368) [69]. The metagenomic reads were aligned to the reference microbial genomes using Bowtie-2. The mapped reads from both indexes were merged by selecting the alignment having the higher identity (≥90% identity). The percent identity was calculated using the formula: %identity = 100*(matches/total aligned length). The normalized abundance of a microbial genome was calculated by summing the total number of reads aligned to its reference genome. For reads showing hits to both indexed databases with equal identity, each genome was assigned 0.5 read count. The relative abundance of each genome was calculated by adding the normalized abundance of each genome divided by the total abundance. The CHI was used to calculate the variance between the clusters compared to the variance within clusters [2].

### Construction of common core microbial functions

To identify the core microbial functions in the gut microbiome of Indian populations and to understand their abundance compared to the other populations, the core microbiome was constructed using a similar strategy as mentioned in MetaHIT [25]. However, to construct a comprehensive core functional microbiome, the information of essential functions from six different microbes including two strains of *Escherichia coli, Bacteroides thetaiotaomicron, Pseudomonas aeruginosa, Salmonella enteric*, and *Staphylococcus aureus* was used instead of considering a single microorganism. The list of essential genes was collected from DEG database v5.0 [70]. A total of 1,890 genes were identified as essential genes in all the six microorganisms. These genes were aligned against eggnog v4.1 database using diamond and were annotated with eggNOG ID [71, 72]. The core gut microbiome functions were also calculated using the above strategy for the US, Denmark, and Chinese population gut microbial samples to remove the variations arising due to differences in data analysis procedures. Apart from identifying the clusters that represented ≥85% genes within the range of essential gene functions, the low prevalent eggNOG functions, which were present in ≥0.0001% abundance in ≥80% of samples in that population, were further filtered out. This added filtration step helped in removing all the low abundant functions. To represent the core, the variance of these functions was also calculated between the two Indian locations. The eggNOGs showing significant deviations in variations (*P* value≤ 0.05; Levene's test) [73] were further filtered out from the analysis.

### Construction of metagenomic species for MGWAS

To identify metagenomic markers using a reference-independent approach on metagenomic samples, a metagenome-wide association study was performed for 340 samples (age and gender matched) including India (both locations), US, China, and Denmark populations. The genes present in at least ≥10% of samples were considered and clustered using the canopy-mgs algorithm as described [74]. The genes having Pearson correlation coefficient (≥0.9) were clustered into CAGs. Furthermore, the genes for which ≥90% abundance was obtained from a single sample were discarded.

To determine the taxonomic origin of each MGS/CAG (metagenomic cluster), all the genes were aligned against reference microbial genomes of 4,097 genomes from HMP and NCBI at nucleotide level using BLASTN [75]. The alignment hits were filtered using an E-value ≤10^−6^ and alignment coverage ≥80% of the gene length, and 2,773,591 (25.6%) genes showed alignments against the reference genomes. The remaining 8,049,540 unassigned genes were aligned against UNIREF database (UniRef 50) at protein sequences [76], of which 4,553,299 genes (56.56%) could be assigned with taxonomic annotations. The sequences that found multiple top hits with equal % sequence identity and scores were further assigned taxonomy based on the lowest common ancestor method. The genes were finally assigned to taxa based on comprehensive parameters of sequence similarity across phylogenetic ranks as described earlier [77]. The identity threshold of ≥95% was used for assignment up to species level, ≥85% identity threshold for assignment up to genus level, and ≥65% identity was used for phylum level assignment using BLASTN. The taxonomic assignments of MGS/CAGs were performed with the criteria that ≥50% genes in each MGS should map to the same lowest phylogenetic group. Thus, if a particular species is assigned ≥50% genes out of the total genes, the assignment will be carried out at species level rather than at genus or higher orders. The relative abundance of MGS/CAGs in each sample was estimated by using relative abundance values of all genes from that MGS/CAG. A Poisson distribution was fitted to the relative abundance values of the data. The mean estimated from Poisson distribution was assigned as the relative abundance of that MGS. The profile of MGS/CAGs were generated and used for further analysis.

### Fecal and serum metabolomic sample preparation and derivatization

Lyophilized fecal samples were used to achieve better metabolite coverage as described previously [78]. Metabolites were extracted with 1 mL of ice-cold methanol: water (8:2) from 80 mg of lyophilized samples in a bath ultrasonicator (Bioruptor UCD-200, Diagenode) at 4°C for 30 minutes followed by 2 minutes of vortexing. The supernatant was extracted by centrifugation at 18,000 g for 15 minutes at 4°C and dried at 50°C under a gentle stream of nitrogen gas. To remove the residual water molecules from the samples, 100 uL of toluene was added to the dry residue and evaporated completely at 50°C under nitrogen gas. Dry extracted metabolites were first derivatized with 50 uL of methoxyamine hydrochloride in pyridine (20 mg/mL) at 60°C for 2 hours, and the second derivatization was performed with 100 uL of N-Methyl-N-trimethylsilyltrifluoroacetamide (MSTFA) in 1% trimethylchlorosilane (TMCS) at 60°C for 45 minutes to form trimethylsilyl (TMS) derivatives. Finally, 150 uL of the TMS derivatives was transferred into GC glass vial inserts and subjected to gas chromatography/time-of-flight mass spectrometry analysis. Serum samples were prepared (polar metabolites only) and derivatized as described by Psychogium et al. [79].

### Method development and validation

Matrix dilution approach was used for validating the linearity and range of dilution [78]. Pooled fecal samples were used to create the reference peaks to validate the peaks coming from individual samples, which were needed due to the presence of a relatively high abundance of fecal metabolites in the pooled samples. The supernatant of feces after extraction was serially diluted 2, 5, 10, 50, 100, 200, and 500 times with methanol: water (8:2). At dilution 2, the maximum numbers of peaks were seen and were processed with the same dilution factor for all the samples. A total of 30 chemical standard mixtures and the pooled fecal samples were used to validate the method. Each stock solution of test standard was carefully prepared in deionized water or with pure ethanol (50 150 350, 500 um) for the determination of linear range, regression coefficient (R2), limit of detection, and repeatability. L-norvaline (1, 2.5, 5, 10, 20 mg/mL in ethanol) was used as a spiked external standard for the optimized derivatization of the method.

### GC-MS analysis

GC-MS analysis was performed on an in-house Agilent 7890A gas chromatograph with 5975C MS system. An HP-5 (25 m × 320 um × 0.25 um i.d.) fused silica capillary column (Agilent J&W Scientific, Folsom, CA) was used with the open split interface. The injector, transfer line, and ion source temperatures were maintained at 220°C, 220°C, and 250°C, respectively. Oven temperature was programmed at 70°C for 0.2 minutes, and increased at 10°C/min to 270°C where it was sustained for 5 minutes, and further increased at 40°C/min to 310°C where it was held for 11 minutes. The MS was operated in the electron impact ionization mode at 70eV. Mass data were acquired in full scan mode from m/z 40 to 600 with an acquisition rate of 20 spectra per second. To detect retention time shifts and enable Kovats retention index (RI) calculation, a standard alkane series mixture (C10–C40) was injected periodically during the sample analysis. RIs are relative retention times normalized to n-alkanes eluted adjacently. For serum samples, we used 2 uL aliquot with a split ratio of 4:1 on the same column as described above. The injector port temperature was held at 250°C, and the helium gas flow rate was set to 1 mL/min at an initial oven temperature of 50°C. The oven temperature was increased at 10°C/min to 310°C for 11 minutes and mass data were acquired in full scan mode from m/z 40 to 600 with an acquisition rate of 20 spectra per second.

### Metabolomic analysis and metabolite profile generation

Raw CDF files were used for peak identification and filtering, and the XCMS package in R was used for pre-processing of the peaks. First, the parameters used for pre-processing of the reads were optimized by calculating the reliability index using the formula given below:

\begin{eqnarray*}
{\rm{Reliability\ index}} &=& {( {{\rm{number\ of\ reliable\ peaks}}} )^2}\nonumber\\
&& /{\rm{number\ of\ unreliable\ peaks}}{\rm{.}}
\end{eqnarray*}

The reliable peaks were identified for each of the settings such as fwhm, S/N, and bw, with a predefined range of values, and regression coefficient was calculated for dilutions of QC samples. The number of peaks with a high coefficient of determination (R^2^ ≥ 0.9) was considered reliable, whereas the peaks with very low R^2^ (≤ 0.05) were considered unreliable peaks [80]. The finally optimized parameters were: profmethod = bin, method = matched Filter, fwhm = 8 and 5 for fecal and serum samples, respectively, and S/N = 12 and 3 for fecal and serum samples, respectively, bw = 5 (for first grouping), smooth = linear, family = gaussian, extra = 1, plot type = mdevden, missing = 8, bw = 3 (for second grouping). Further, to compare across multiple samples, the peak intensities were normalized (root transformed) and scaled using z-transformation. These normalized and scaled peak intensities were used for further statistical analysis.

A multivariate statistical method, OPLS-DA [81], was used to identify differences between LOC1 samples (n = 53) and LOC2 (n = 55) samples. Metabolites driving the differences were identified in metabolic profiles of LOC1 and LOC2 samples using correlations coefficients. The clusters of co-abundant metabolite profiles were identified using R package “WGCNA” [82]. Signed weighted metabolite co-abundance correlation after scaling and centering was calculated across all samples. The soft threshold of β = 15 was chosen for scale-free topology. The dynamic hybrid tree cutting algorithm was used to identify the clusters with a deepsplit = 4 and minimum cluster size = 4. The profile of each fecal metabolite cluster was summarized using eigenvector. The abundance profile of each cluster of metabolites (MES) was calculated using the same methodology as used for MGS cluster abundance profiles.

### Retention index calculation

GC-MS data obtained from the alkene series run was used to calculate the RI for each peak in the samples, and the obtained RI values were further used at the time of library search for the identification of individual metabolite.

\begin{equation*}
I\ = \ 100\ X\ [n + (logtx - \log tn)/(\log tn + 1 - \log tn)
\end{equation*}

Where, tx = retention time of the peak, tn = retention time of preceding alkane, and tn+1 = retention time of the following alkane.

### Clustering and enterotype analysis

Cluster of samples in the dataset were identified from the relative abundance profiles of Genus or Orthologous groups (OG) in the samples. The Jensen-Shannon distances (which estimates the probability distributions between the samples) were calculated, and the abundance profiles were clustered using partitioning around medoids (PAM) clustering algorithm as mentioned previously [83]. The optimal number of clusters was assessed using CHI that has shown good performance in recovering the optimal number of clusters [84]. Similarly, the prediction strength from “fpc” package in R, which used the cross-validation approach, was also employed as another metric for cluster validation. Both the CHI and prediction strength showed quite significantly correlated results. For clustering, CHI and prediction strength gave non-identical values; silhouette index was calculated to estimate the robustness of clusters.

### Between-class analysis

The between-class analysis was performed to identify the drivers and support the clustering of the genus/species/OG abundance profiles into clusters. The between-class analysis is a type of principal component analysis with instrumental variables that maximize the separation between classes of this variable. The instrumental variable here is the cluster classification using PAM clustering and the top species, which contributed the maximum to the principal components obtained from between-class analysis, were identified as driver species/genus/OG based on their eigenvalues. The analysis was performed using ade4 package in R.

### Diversity analysis

The inter-sample Canberra distances were also calculated using MGS Abundance between populations. The richness of microbiome samples across populations was obtained from Shannon index calculated using raw gene abundance table rarefied at equal depth (1,000,000 seqs/sample) over n = 30 random samplings. The beta diversity for 16S rRNA genes (between the samples) was calculated as unweighted UniFrac distances using OTU tables rarefied at 100,000 seqs/sample and phylogenetic distance between representative sequences from each OTU [85]. The effects of covariates such as age, diet, location (LOC1 and LOC2), and gender were compared for correlation, with principal components identified from principal component analysis using UniFrac distances. The polyserial correlations with *P* values were calculated for categorical variables, and the significance of the covariates for explaining the variation was estimated at each principal component.

### Network analysis

Spearman rank correlations were computed between each of the species/MGS and between the MGS and functional modules/metabolites. The correlations with significant *P* values were selected and were used for the network analysis. The undirected links were generated between correlated nodes (species/KOs/modules), and the strength of the links was given weight based on their correlation coefficients. The network structure was generated using “igraph” package in R. The modularity of the network for KOs association was generated with each module representing the functional modules defined in KEGG database. The negative correlation was not considered in generating the network modules. Moreover, the positive correlations were filtered (ρ ≥ 0.6) for most of the network analysis.

### Supervised learning

Predictive models were built using supervised machine learning algorithm Random Forest [86]. The models were optimized using 10,000 trees and default settings of mtry (number for variables used to build the model). The mean three-fold cross-validation error rates were calculated for each binary tree and the ensemble of trees. The mean decrease in accuracy, which is the increase in error rates on leaving the variable out, was calculated for each prediction and tree and was used to estimate the importance score. The variables showing a higher mean decrease in accuracy of prediction were considered important for the segregation of the datasets into groups based on the categorical variable.

## Statistical Analysis

All the statistical comparisons between groups were performed using negative binomial model-based Wald test implemented in DESeq2 and non-parametric Wilcoxon rank-sum test with FDR adjusted *P* values to control for multiple comparisons [87–89]. The correlations between two variables and the correlations within were calculated using Spearman correlation coefficient with adjusted *P* values [90]. The correlations between categorical and numeric variables were performed using Polyserial correlation/biserial correlations [91]. To identify the enrichment of enzymes/species associated with a host, odds ratio was used as a measure of the enrichment of a feature in a group. The odds ratio was calculated as OR (k) = [∑_s = LOC1_ A_sk_/∑_s = LOC1_(∑_i≠k_ A_si_)]/[∑_s = LOC2_ A_sk_/∑_s = LOC2_ (∑_i≠k_ A_si_)] for enrichment of genes/species between two locations, where A_sk_ denotes abundance of species/gene k in sample S. Also the enrichment of species/genes between Indian microbiome compared to other datasets consisting of US, Denmark and China referred as “OTHERS” were computed as OR(k) ([∑_s = INDIA_ A_sk/_∑_s = INDIA_(∑_i≠k_ A_si_)]/[∑_s = OTHERS_ A_sk_/∑_s = OTHERS_ (∑_i≠k_ A_si_)]). All the graphs and plots were generated using the ggplot2 package in R.

### Correlation analysis between functional modules and metabolite clusters

To calculate the association of microbial functional modules with fecal metabolite clusters, the Spearman correlation coefficients were calculated to rank KOs for association with metabolite clusters and metabotypes. To quantify the shift in Spearman correlation between given KEGG module and the metabolite cluster compared to the background distribution, the background adjusted median Spearman correlation was calculated for a given KEGG module m as:

\begin{equation*}
{\rm SCC}_{\rm bg.adj} = {\rm median}\,\left({\rm SCC}_{\rm KOs\epsilon\,KEGG\,Module\,m}\right) \nonumber\\
\qquad\qquad\qquad -{\rm median}\,\left( {\rm SCC}_{\rm KOs\,KEGG\,Module\,m} \right)
\end{equation*}

Where SCC_KO_ is the partial Spearman correlation coefficient between KO and the metabolite cluster.

Identification of microbial species driving the association between KEGG module and metabolite abundance was done by iterating the correlation between KO belonging to the KEGG module and the metabolite after excluding the genes annotated to that KO from each species. The change in median Spearman correlation coefficient between the KOs and the metabolite, when genes from that species are excluded from the analysis, was calculated as described previously [8]. The species showing the maximum change in the overall correlation of module with metabotype was plotted.

## Availability of supporting data

The datasets generated and/or analyzed during the current study have been deposited in the NCBI BioProject database under project number PRJNA397112. Metabolomics data are available via the MetaboLights database (accession number MTBLS803). Supporting data are also available via the *GigaScience* repository, GigaDB [92].

## Additional Files

**Additional File 1:** Supplementary data containing the metadata and sample information

**Additional File 2:** Summary of sequencing statistics showing the number of reads per sample for 16S rRNA gene amplicon dataset

**Additional File 3:** Summary of sequencing statistics showing the number of reads per sample for Whole Genome Shotgun metagenomic dataset

**Additional File 4:** Summary of the reads mapped to Integrated Gene Catalogue and Indian catalogue combined with IGC.

**Additional File 5:** Figures S1 to S18

**Additional File 6:** Differentially abundant MGS between India and other populations

**Additional File 7:** Differentially abundant functions (Kegg Orthologues (KOs) and EggNOGs) between India and other populations.

**Additional File 8:** Sample-wise representation of Indian samples into Enterotypes identified from Meta-analysis with 37 samples from four nations used in Arumugam et al.

**Additional File 9:** Calinski Harabasz index and prediction strength calculated for clusters derived from 16S rRNA gene based genus abundance, metagenome based species abundance and metagenome based KO abundance profiles.

**Additional File 10:** Mean relative abundance of genus in Cluster-1 and Cluster-2 and their associated *P*-values of difference calculated using NB model based Wald test.

**Additional File 11:** The sample-wise association into clusters using Genus based and KO based clustering and their differences.

**Additional File 12:** Differentially abundant KEGG orthologue functions between Cluster-1 and Cluster-2.

**Additional File 13:** Polyserial correlation of covariates with principal components explaining variations across samples using unweighted UniFrac distances.

**Additional File 14:** Differentially abundant MGS observed between two locations and their enrichment calculated using Log Odds ratio and NB model based *P*-values.

**Additional File 15:** Polyserial correlation of covariates with principal components explaining variations across samples using metabolomics data.

**Additional File 16:** Table shows the Spearman's rank correlation coefficient values of metabolites with Metabotypes.

**Additional File 17:** Table shows the differential abundance of KEGG Modules between LOC1 and LOC2

**Additional File 18:** List of reference genomes from NCBI and HMP databases for reference mapping

giga-d-18-00212_original_submission.pdf

giga-d-18-00212_revision_1.pdf

giga-d-18-00212_revision_2.pdf

response_to_reviewer_comments_original_submission.pdf

response_to_reviewer_comments_revision_1.pdf

reviewer_1_report_original_submission -- Sean P Kenedy, Ph.D 7/31/2018 Reviewed

reviewer_1_report_revision_1 -- Sean P Kenedy, Ph.D 11/20/2018 Reviewed

reviewer_2_report_original_submission -- CÃ©dric Laczny 8/14/2018 Reviewed

reviewer_2_report_revision_1 -- CÃ©dric Laczny 11/21/2018 Reviewed

Supplemental Files

## Abbreviations

BCAA: branched chain amino acid; BMI: body mass index; CAG: co-abundance gene group; CHI: Calinski Harabasz index; FDR: False Discovery Rate; GC-MS: gas chromatography/mass spectrometry; GO: Genus or Orthologous groups; HMP: Human Microbiome Project; IGC: integrated gene catalogue; IISER: Indian Institute of Science Education and Research; KO: Kyoto Encyclopedia of Genes and Genomes Ortholog; LOR: Log Odds Ratio; MGS: metagenomic species; MGWAS: metagenome-wide association study; NB: negative binomial; NCBI: National Center for Biotechnology Information; PAM: partitioning around medoids; OPLS-DA: Orthogonal Projections to Latent Structures Discriminant Analysis; ORF: open reading frame; OTU: operational taxonomic unit; PCA: principal component analysis; RI: retention index; SCFA: short chain fatty acid; T2D: type-2 diabetes; WGS: whole-genome shotgun.

## Collection of datasets for comparative analysis

The 74 HMP metagenomes were collected from http://hmpdacc.org/HMASM or NCBI SRA (accession SRR059347). The 85 Danish fecal metagenomes from METAHIT were obtained from European Nucleotide Archive (http://www.ebi.ac.uk/ena, study accession number ERP000108). The 71 Chinese metagenome samples were obtained from NCBI SRA (accession number—SRR341581).

## Ethics approval and consent to participate

The recruitment of volunteers, sample collection, and other study-related procedures were carried out by following the guidelines and protocols approved by the Institute Ethics Committee of IISER, Bhopal, India. Written informed consent was obtained from all subjects prior to any study-related procedures.

## Competing interests

The authors declare that they have no competing interests.

## Funding

This work was supported by the intramural funding received from IISER Bhopal, Madhya Pradesh, India.

## Author contributions

V.K.S. and A.M. conceived the work and participated in the design of the study. A.M. and J.P. collected all the samples in collaboration with T.G. A.M. designed the study protocols and performed sample processing, DNA extraction, metabolite extraction, and profiling from fecal and blood samples. R.S. and A.M. carried out the library preparation and sequencing work. D.B.D. carried out all metagenomic data and statistical analysis. A.K.S. and D.B.D. analyzed the metabolomics data. A.M. and D.B.D. did the primary data interpretation of analytical outcomes under the supervision of V.K.S. A.M., D.B.D., A.K.S., R.S., A.G., J.S., K.R.A., and V.K.S. drafted the manuscript. All authors read and approved the final manuscript.
