# Supplementary material for: The unique composition of Indian gut microbiome, gene catalogue, and associated fecal metabolome deciphered using multi-omics approaches
Source: Gigascience. 2019 Jan 30;8(3):giz004. doi: 10.1093/gigascience/giz004 (PMC6394208; doi:10.1093/gigascience/giz004)
Supplement: giga-d-18-00212_original_submission.pdf [file giz004_giga-d-18-00212_original_submission.pdf]

GigaScience

Multi-omics analysis reveals Indian gut microbiome variations due to diet and location and its implications on human health

--Manuscript Draft--

**Manuscript Number:**	GIGA-D-18-00212
**Full Title:**	Multi-omics analysis reveals Indian gut microbiome variations due to diet and location and its implications on human health
**Article Type:**	Research
**Funding Information:**	
**Abstract:**	Background<br><br>Metagenomic studies carried out in the past decade have led to an enhanced understanding of the gut microbiome in human health, however, the Indian gut microbiome is not well-explored yet. We comprehensively analysed the gut microbiota of 110 healthy individuals from two distinct locations (North-Central and Southern India) using multi-omics approaches, including 16S rRNA marker gene and shotgun metagenomics, and faecal and serum metabolomics.<br><br>Results<br><br>The gene catalogue established in this study highlighted the uniqueness of the Indian gut microbiome in comparison to other populations. The North-Central population, which was primarily consuming a plant-based diet, was found to be associated with Prevotella, and thus showed an enrichment of BCAA and lipopolysaccharide biosynthesis pathways. In contrast, the South-Indian population, which was consuming an omnivorous diet, showed associations with Bacteroides, Ruminococcus and Faecalibacterium, and had an enrichment of SCFA biosynthesis pathway and BCAA transporters. This corroborated with the metabolomic results, where the BCAA levels were observed to be higher in the serum metabolome of the North-Central population, apparently regulated by Prevotella. In contrast, BCAAs were found higher in the faecal metabolome of South-Indian population, which was correlated with the enrichment of BCAA transporters.<br><br>Conclusions<br><br>The study demonstrates the influence of location and diet on the gut microbiome and its functional consequences on human health, and supplements the current knowledge on the poorly characterized Indian gut microbiome. The integrated approach used provides novel insights on the gut-microbe-metabolic axis, which will be useful for future epidemiological and translational researches.
**Corresponding Author:**	Vineet Kumar Sharma, Ph.D.<br>Indian Institute of Science Education and Research Bhopal<br>Bhopal, Madhya Pradesh INDIA
**Corresponding Author Secondary Information:**	
**Corresponding Author's Institution:**	Indian Institute of Science Education and Research Bhopal
**Corresponding Author's Secondary Institution:**	
**First Author:**	Darshan B Dhakan
**First Author Secondary Information:**	
**Order of Authors:**	Darshan B Dhakan
	Abhijit Maji
	Ashok K Sharma

Powered by Editorial Manager® and ProduXion Manager® from Aries Systems Corporation

	Rituja Saxena
	Joby Pulikkan
	Tony Grace
	Andres Gomez
	Joy Scaria
	Katherine R Amato
	Vineet Kumar Sharma, Ph.D.
**Order of Authors Secondary Information:**	
**Additional Information:**	
**Question**	**Response**
Are you submitting this manuscript to a special series or article collection?	No
**Experimental design and statistics**<br><br>Full details of the experimental design and statistical methods used should be given in the Methods section, as detailed in our Minimum Standards Reporting Checklist. Information essential to interpreting the data presented should be made available in the figure legends.<br><br>Have you included all the information requested in your manuscript?	Yes
**Resources**<br><br>A description of all resources used, including antibodies, cell lines, animals and software tools, with enough information to allow them to be uniquely identified, should be included in the Methods section. Authors are strongly encouraged to cite Research Resource Identifiers (RRIDs) for antibodies, model organisms and tools, where possible.<br><br>Have you included the information requested as detailed in our Minimum Standards Reporting Checklist?	Yes
**Availability of data and materials**<br><br>All datasets and code on which the conclusions of the paper rely must be either included in your submission or deposited in publicly available repositories (where available and ethically appropriate), referencing such data using	Yes

a unique identifier in the references and in the “Availability of Data and Materials” section of your manuscript.<br><br>Have you have met the above requirement as detailed in our Minimum Standards Reporting Checklist?	

Powered by Editorial Manager® and ProduXion Manager® from Aries Systems Corporation

Manuscript
Click here to download Manuscript Manuscript_IGM_GigaScience.docx

Title: Multi-omics analysis reveals Indian gut microbiome variations due to diet and location and its implications on human health

Authors: D.B. Dhakan[1†], A. Maji[1†], A.K. Sharma[1], R. Saxena[1], J. Pulikkan[2], T. Grace[2,3], A. Gomez[4], J. Scaria[5], K.R. Amato[6], V.K. Sharma[1]*

Affiliations: [1]Metagenomics and Systems Biology Laboratory, Department of Biological Sciences, Indian Institute of Science Education and Research Bhopal, India, [2]Department of Genomic Science, Central University of Kerala, India, [3]Division of Biology, Kansas State University USA, [4]Microbiomics Laboratory, Department of Animal Science, University of Minnesota, USA, [5]Animal Disease Research & Diagnostic Laboratory, Veterinary and Biomedical Sciences Department, South Dakota State University, USA, [6]Department of Anthropology, Northwestern University, USA

Email IDs: darshan@iiserb.ac.in, abhi71084@gmail.com, ashoks773@gmail.com, ritus@iiserb.ac.in, puljobcmi@gmail.com, tonygrace99@gmail.com, gomeza@umn.edu, joy.scaria@sdstate.edu, katherine.amato@northwestern.edu, vineetks@iiserb.ac.in

[†] These authors contributed equally to this work

*Corresponding author

V.K. Sharma: vineetks@iiserb.ac.in

Abstract

Background

Metagenomic studies carried out in the past decade have led to an enhanced understanding of the gut microbiome in human health, however, the Indian gut microbiome is not well-explored yet. We comprehensively analysed the gut microbiota of 110 healthy individuals from two distinct locations (North-Central and Southern India) using multi-omics approaches, including 16S rRNA marker gene and shotgun metagenomics, and faecal and serum metabolomics.

Results

The gene catalogue established in this study highlighted the uniqueness of the Indian gut microbiome in comparison to other populations. The North-Central population, which was primarily consuming a plant-based diet, was found to be associated with *Prevotella*, and thus showed an enrichment of BCAA and lipopolysaccharide biosynthesis pathways. In contrast, the South-Indian population, which was consuming an omnivorous diet, showed associations with *Bacteroides*, *Ruminococcus* and *Faecalibacterium*, and had an enrichment of SCFA biosynthesis pathway and BCAA transporters. This corroborated with the metabolomic results, where the BCAA levels were observed to be higher in the serum metabolome of the North-Central population, apparently regulated by *Prevotella*. In contrast, BCAAs were found higher in the faecal metabolome of South-Indian population, which was correlated with the enrichment of BCAA transporters.

Conclusions

The study demonstrates the influence of location and diet on the gut microbiome and its functional consequences on human health, and supplements the current knowledge on the poorly

characterized Indian gut microbiome. The integrated approach used provides novel insights on the gut-microbe-metabolic axis, which will be useful for future epidemiological and translational researches.

Keywords: Indian Gut Microbiome, Metagenomics, Metabolomics, Enterotypes, Integrated Gene Catalog, Metagenome-Wide Association Study, Core gut microbiota, Short Chain Fatty Acids, Branched Chain Amino Acids, Lipopolyschharides.

Background

Determining the constitution of a healthy gut microbiota and understanding its variability across populations is essential for assessing the impact of microbial dysbiosis on human health. Several large-scale, world-wide microbiome projects have revealed variability in the gut microbial composition of healthy individuals due to factors such as mode of delivery, age, geographical location, diet and lifestyle, and have helped in the better understanding of gut microbiome in human health and disease [1-5]. Most gut microbiome studies have determined microbial taxonomy and functional diversity using marker gene-based and/or WGS approaches to understand the functional role of the gut microbiome. However, novel insights on the complex interplay between diet, gut microbes and human health in the context of key microbial metabolites, such as short-chain fatty acids (SCFAs) and Branch Chain Amino Acids (BCAAs), derived from the microbial fermentation of dietary fibres are beginning to emerge from recent gut metabolomics studies [6, 7]. Moreover, the direct impact of the microbial metabolome on human health is also becoming apparent from the recent studies focusing on the 'gut microbiome- host metabolism axis' [8]. Therefore, an integrative approach using both metagenome and metabolome- based characterizations of the gut microbiome appears pragmatic for gaining deeper functional and mechanistic insights into the role of gut microbes on human health.

The significant large-scale studies carried out so far represent the gut microbiome of urban populations majorly from Europe, US and other allegedly named WEIRD countries (i.e., the Western, Educated, Industrialized, Rich, and Democratic countries) [9]. Only recently, some initiatives have been taken for the characterization of the human microbiome from diverse ethnic populations, which have shown significant variations from the major world populations [9-14]. India is the seventh largest country in the world and harbours the second largest population with

enormous diversity in populations, lifestyles and dietary habits across multiple geographical locations. India is also a home to the majority of the world's vegetarian population, but is equally dominated by people consuming both vegetarian and animal-based diets. Moreover, the Indian population has the highest prevalence of diabetes worldwide [15] and according to the World Health Organization estimates (WHO, 2011), 53% of deaths in India in the year 2008 were attributed to non-communicable conditions such as diabetes and cardiovascular diseases, which are predicted to reach ~75% by 2030 [16]. The gut microbiome has been implicated in many such diseases in India and in other populations [14, 17, 18]. Describing population-specific variations in the microbial profile of healthy individuals is critical for identifying population-specific as well as universal microbiome-based biomarkers for specific diseases [19]. A few studies have investigated the gut microbiome of the Indian population, but all were focused on small cohort sizes and have relied only on 16S rRNA gene-based sequencing analysis [10, 20, 21]. Therefore, investigating the impact of diet and location on the gut microbiome of the Indian population is crucial for improving our understanding on the role of the gut microbiome in health and disease in a global context.

To cover the enormous gut microbiome diversity inherent in the different sub-populations of India, extensive sampling and analyses are required. Therefore, as the first large-scale study from India, we selected two prominent locations in North-Central India, i.e. LOC1: Bhopal city, Madhya Pradesh, and Southern India, i.e. LOC2: Kerala. The dietary habits between the two locations are very different, as the South-Indian population (LOC2) diet consists of rice, meat and fish, whereas the North-Central population (LOC1) consumes a carbohydrate-rich diet including plant-derived products, wheat and trans-fat food (high-fat dairy, sweets and fried snacks). In addition, the 'Human Development Index Report, UNDP' (United Nations Development Programme), India

and SRS-based life-table (Sample Registration Survey, 2010-14) has revealed that the citizens from Kerala had the highest life-expectancy rates (>74 years) in India, while those in Madhya Pradesh (capital city 'Bhopal') exhibited the lowest (<65 years) [22]. Further, it is known that there is a higher predisposition of the North-Indian population towards diabetes, cardiovascular diseases and hypertension, which in contrast is much lower in Southern India, perhaps due to the lifestyle differences in the two regions [3]. Thus, to gain deeper functional insights into the microbiome from these two distinct and representative sub-populations of India, a comprehensive multi-omics approach was carried out using amplicon-based profiling of taxonomic composition (16S rRNA sequencing), WGS-based profiling of metagenomic content and GC-MS-based profiling of faecal and serum metabolomic signatures.

Data Description

The two selected locations, Bhopal (LOC1) and Kerala (LOC2) provided a distinct representation of the Indian population in the context of diets and lifestyle from North-Central and Southern parts of India, which are almost 2000 km apart **(Additional File 1)**. The 110 (62 females, 58 males) individuals recruited in this study were not suffering from any disease as reported by personal medical history and physical examination, and confirmed no exposure to antibiotics for at least one month prior to sampling, and thus, were considered as 'healthy' (**Additional File 1**). The sequencing of V3 hypervariable region of 16S rRNA gene and shotgun metagenome sequencing from the 110 faecal samples resulted into 54.87 million paired-end reads (503,460 ± 175,547 (mean ± sd) reads/sample) and 499.98 million paired-end reads (4,545,280 ± 1,498,663 (mean ± sd) reads/sample), respectively (Methods, **Additional file 2** and **Additional file 3)**.

Analyses

Construction of an Indian gut microbial gene catalogue and updated integrated gene catalogue (IGC)

The first step for functional analysis was the construction of an extensive catalogue of gut microbial genes from the Indian population, which was not yet available. A De Bruijn graph-based assembly of reads resulted in 1,337,547 contigs of length ≥300 bp with a total contig length of 1.78 Gbp representing 43% of the total reads. To obtain assemblies of low coverage genomic regions or genomes present in the Indian gut microbiome, the singletons from all the samples were combined and assembled into additional 0.33 million contigs with length ≥300 bp and a total assembled length of 232 Mbp. The ORFs predicted from contigs resulted in 1,479,998 non-redundant genes, which represent the gene catalogue of the Indian gut microbiome. In addition, the integrated gene catalogue (IGC) represents a cohort of 9,879,896 genes identified from 1,267 gut metagenomes from three populations of the world (HMP, MetaHIT and Chinese dataset), and was also updated with the Indian gene catalogue to construct an updated IGC [1, 23, 24]. A total of 718,360 non-redundant genes were added from Indian samples, which increased the size of IGC to 10,598,256 non-redundant genes (6.7% increase), and was referred to as 'updated IGC'. A total of 69.2% (± 4.01%) mapping coverage of reads (~6% increase in the mapping of reads) was observed on the updated IGC as compared to 63% (± 4.61%) mapping on the previous non-updated IGC (**Additional File 4**). However, a similar increment in mapping coverage of reads for other population datasets was not observed, and the mapping coverage of HMP (67.74%), China (73.38%) and MetaHIT (75.21%) on the updated IGC were comparable to their mapping coverage to IGC (**Fig. 1A**). This analysis indicates that the genes contributed by the Indian gut microbiome are unique and not represented in other gut microbiome datasets.

Identification of taxonomic signatures of Indian gut microbiome

To determine the taxonomic and functional composition of the Indian gut microbiome and to identify Indian specific gut-microbial signatures, a cross-population comparison was carried out using the 16S rRNA and metagenomic data from other populations. In order to derive metagenomic markers for comparison with similar large-scale studies from other populations, a non-reference based metagenome-wide association study (MGWAS) was carried out [25]. The genes from metagenomic samples of four countries (India, China, USA and Denmark) were clustered (see Methods) into 866 clusters based on their co-occurrence and higher Pearson correlations across samples ($\rho = 0.9$) resulting into 224 MGS (metagenomic species) having ≥700 genes in each cluster, and 642 CAGs (co-abundance gene groups) consisting of ≥50 genes in each cluster. Out of the 866 metagenomic clusters, 197 could be assigned up to species level using the taxonomic assignment strategy described in Methods. Jaccard distances were calculated from MGS/CAG abundance profiles and their PCA analysis was carried out using 'countries' as factors for explaining the variance between samples, which showed that the Indian population formed a distinct cluster separated from the other populations at PC1 (**Fig. 1B**). The MGS/CAGs annotated as *Prevotella*, *Mitsuokella*, *Dialister*, *Megasphaera*, and *Lactobacillus* were found to be the drivers of this separation as observed from their factor loading scores, and were associated with and enriched in the Indian population. Further, the identification of enriched MGS showed that the species belonging to the genus *Clostridium*, and phylum Firmicutes and Bacteroides were depleted in the Indian population and were enriched in the other populations (China, Denmark and USA; Log Odds Ratio <-7 and P-value <0.001) (**Additional File 5: Figure S1**). Furthermore, the distribution of microbial families from different populations was also calculated across the globe using 16S rRNA markers. A cross-population comparison revealed Indian gut microbiome to have

a higher abundance of Prevotellaceae and Veillonellaceae, suggesting them as the marker microbial families associated with the Indian population (**Fig. 1C**).

Microbial functions enriched in the Indian population

Functional comparison of Indian microbiome with other populations was carried out by mapping the genes derived from assembled contigs to the EggNOG database. In total 68,693 EggNOG functions were identified from the Indian gut microbiome, including 1,726 novel functions obtained from clustering the unmapped genes (see Methods). The core microbial functions which are essential for microbial survival and are present in almost 80% individuals were used for the functional comparison. The core microbiome was derived using a similar strategy as employed in MetaHIT (see Methods) [26]. A core microbial EggNOG profile was generated using a gene cohort comprising of 1,890 essential genes from six bacterial species namely, *Escherichia coli* MG1655I and MG165II, *Bacteroides thetaiotaomicron* VPI-5482, *Pseudomonas* PA01, *Salmonella enteric* serovar Typhi and *Staphylococcus aureus* NCTC 8325. The eggNOGs were ranked based on their mean abundance in descending order, and the range that included 85% of essential genes were considered for building the core microbial eggNOG set and were used for the analysis. Most of the essential genes were included in the top-ranking clusters suggesting that the essential genes are present in higher abundance than the accessory function genes (**Additional File 5: Figure S2**). The core microbiome of Indian samples was compared with the core microbiome of USA, China and Denmark populations. The proportion of essential genes covered by top-ranking nine eggNOG clusters showed that 85% of the essential genes could be covered in the least number (15,000) of eggNOGs in the case of Indian population, while in the case of Denmark it was covered by twice the number (30,000) of eggNOGs (**Additional File 5: Figure S3**). These observations suggest that the core functional microbiome of Indian population is less diverse than other populations. This

also corroborates with the alpha diversity (Shannon) calculations using gene abundances, which showed that the Indian microbiome is less diverse than the microbiome of other world populations (**Additional File 5: Figure S4**). In total, 5,296 eggNOGs were characterized as core functions commonly present in the core microbiome of all the four population datasets. The co-inertia (Procrustes) analysis and the Eigen values, and their scores calculated from PCA, using both core and accessory functions also showed that the Indian gut microbiome was significantly different from other datasets (**Fig. 2A and 2B**). This data also shows the uniqueness of Indian microbial functions in composition and diversity at both core and accessory levels. The Indian microbiome was found to be enriched (FDR Adj. $P<0.05$, Log Odds Ratio >1.5) in functions for carbohydrate and energy metabolism including degradation of complex polysaccharides, which corroborates well with the carbohydrate-rich diet of the Indian population (**Fig. 2C** and **Additional File 6**).

Detection of enterotypes and variations in Indian gut microbiome between locations

To determine the diversity of gut microbial communities present in the Indian population, detection of enterotypes (groups of samples having similar profiles and lesser variance) was performed using an unsupervised clustering approach [2]. The Jensen Shannon distance matrices were used and principal component analysis identified two prominent enterotypes. ET-1 was primarily driven by *Prevotella* ($P<0.001$), and ET-2 was driven by other microbes belonging to Bacteroides ($P<0.02$), *Ruminococcus* ($P<0.001$) and *Faecalibacterium* ($P< 0.02$) (**Additional File 5: Figure S5**, **Additional File 7**). The abundances of *Prevotella* in LOC1 and Bacteroides in LOC2 in India are perhaps due to the dietary habits of the two locations. The LOC1 population was mainly consuming a carbohydrate-rich diet comprising of vegetable-based foods and grains, whereas the LOC2 population was consuming a diet consisting of rice, meat and fish. These patterns seem to align with the patterns reported in other populations [27, 28].

The robustness of clusters was demonstrated using Calinski Harabasz index (CHI) and prediction strength, which uses a cross validation approach (**Additional File 8**). A similar cluster analysis performed using the functional information derived from the abundance of KEGG Orthologs (KO) also showed the clustering of samples into two enterotypes, named as C1 and C2 (**Additional File 5: Figure S6)**. In comparison to enterotypes derived from taxonomic information, only 16 out of 110 samples were placed in different clusters using the functional information revealing significant concordance (FDR Adj. $P<0.0001$, Spearman's correlation Coefficient = 0.69). C1 was found enriched in genes coding for enzymes such as Phytase (Log Odds Ratio (LOR) = 2.96), β-glucosidase (LOR = 1.75), and α-fucosidase (LOR = 1.32), which are involved in the breakdown of plant-polysaccharides, whereas the genes coding for enzymes such as lipase (LOR = -5.34), carnitine-coA dehydratase (LOR = -2.59) and amino peptidase (LOR = -2.66), which are involved in the metabolism of animal-based diet, were enriched in C2 (FDR Adj. $P<0.05$) (**Additional File 9**).

To identify the components explaining the variations in microbial profiles across samples, unweighted UniFrac distances were calculated using 16S rRNA sequences rarefied at 100,000 sequences per sample. The principal component analysis (PCA) of Unifrac distances and the scores for each sample correlated with the covariates using polyserial correlation, and distinct locations (LOC1 and LOC2) and diets (vegetarian and omnivorous) were identified to be the major variables explaining the variation between samples at PC2 (**Fig. 3A, Additional File 10**). A comparison of taxonomic and functional diversity performed between the two locations using Shannon diversity index and rarefactions of genes from each sample, also showed that the microbiome profiles of LOC2 populations were more diverse in their composition compared to LOC1 populations (**Fig. 3B** and **Additional File 5: Figure S7**). The inter-individual Bray Curtis distances of gene profiles

between LOC1 and LOC2 populations also showed significant differences (FDR Adj. P<0.05), where LOC2 population displayed higher inter-individual heterogeneity in their microbial community structure as compared to LOC1 population **(Fig. 3C)**.

Major differences in the microbiome profiles (using the 16S rRNA dataset) at the phylum level were apparent from the higher Bacteroidetes to Firmicutes ratios (P<0.002) in LOC1 (1.93) compared to LOC2 (0.86), which have been previously reported as a result of differences in dietary habits, i.e. vegetarian or plant-based (carbohydrate-rich) vs. omnivore or animal-based (protein-rich) diets (**Additional File 5: Figure S8**) [29, 30]. Notably, these variations were not attributable to BMI (Spearman's Rank correlation, FDR Adj. P=0.78). At the genus level also *Prevotella*, *Megasphaera*, *Mitsuokella*, and *Lactobacillus* were observed to be higher in LOC1, whereas *Ruminococcus*, *Clostridium*, *Faecalibacterium* and *Roseburia* were higher in LOC2 (FDR Adj. P<0.05, Wilcoxon rank sum test); (**Fig. 3D & E**). Similarly, out of 107 marker MGS/CAG obtained from MGWAS, those annotated to *Prevotella copri* were found enriched in LOC1 (Log Odds Ratio > 2; FDR Adj. P<0.05; 41 MGS/CAG), whereas MGS/CAGs annotated to SCFA producing species such as *Faecalibacterium prausnitzii* and *Roseburia inulinivorans*, were enriched in LOC2 (FDR Adj. P<0.05; Log Odds Ratio < -2; 66 MGS/CAG) (**Additional File 11**). Interestingly, the two species found higher in LOC2 are known SCFA producers and have also been regarded as commensals with anti-inflammatory properties [31]. In contrast, *Prevotella*, which was abundant in the LOC1, is known to be associated with high fibre-rich diet [32].

Defining the Indian gut metabolome

The analysis of microbial community structure and functions from the two locations having different lifestyle and diet revealed significant insights. Previous studies have shown a direct role of diet in the selection of differential gut microbiomes [33]. Thus, to gain deeper insights into the

metabolic activity of microbiomes from LOC1 and LOC2 as driven by different diets, faecal metabolites were analysed using a GC-MS-based metabolomics approach. An unsupervised between class analysis of metabolomic profiles separated the samples into three separate clusters, and the robustness was confirmed using prediction strength and Silhouette index (**Fig. 4A and 4B**). Polyserial correlation of covariates showed location to be the major factor explaining the variation at PC1 (FDR Adj. $P<0.01$) separating Metabotype-1 from Metabotype-2 and 3. In contrast, vegetarian and omnivorous diet groups emerged as other factors explaining the variation at PC2 (FDR Adj. $P<0.01$), and separating Metabotype-2 from 3 (**Additional File 12**). The OPLS-DA model derived from normalized peak intensities also showed differential clustering of samples from the two locations (**Fig. 4C, Table 2**). Metabotype-1 was associated with LOC1 and showed higher abundances of saturated fatty acids including palmitic acid, stearic acid, and valeric acid. Metabotype-3 was associated with LOC2 and showed higher abundances of BCAAs valine, leucine and isoleucine, and SCFAs propionate and butyrate. Metabotype-2 was enriched in D-glucose, galactose, mannose, lauric acid and cadaverine (a polyamine that denotes meat consumption) [34].

Positive correlation of BCAA transporters with BCAA levels in faecal metabolome

We also identified the marker metabolites, which showed significant (Spearman's correlation, FDR Adj. $P<0.05$) associations with LOC1 or LOC2. In total, 17 metabolite clusters were identified, of which nine were associated with LOC1, and eight were associated with LOC2 (**Additional File 13**). These marker metabolites showed a positive association with MGS/CAGs. For instance, *Prevotella* annotated clusters correlated significantly with valeric acid and sedoheptulose metabolite markers, which showed a higher relative abundance in LOC1. In contrast, MGS/CAGs belonging to *Faecalibacterium*, *Clostridium*, *Ruminococcus*, and *Alistipes*

were positively associated with BCAAs, cadaverine, propanoate and lauric acid in LOC2 (**Fig. 5A**). In addition to the positive association of BCAAs with species enriched in LOC2, a correlation analysis of significantly different (FDR Adj. P<0.05, Wilcoxon rank sum test; **Additional File 14**) functional modules revealed that faecal BCAA abundances were positively correlated with BCAA transporter abundance in LOC2. In contrast, BCAA abundance in the faecal metabolome showed a negative correlation (P<0.05) with BCAA biosynthesis pathways (**Fig. 5B**).

The above observations are significant given that BCAAs are important metabolites involved in glucose homeostasis, by stimulating insulin secretion [35]. Higher BCAA levels in the faecal matter could be a result of its inward transport in microbial cells by the BCAA transporters, thus leading to their accumulation in the colon lumen. This is concordant with higher relative abundance of *Bacteroides vulgatus* and *Eubacterium sireaeum* in LOC2 compared to LOC1, which are known to harbour higher abundance of BCAA transporters [36]. Further support for this hypothesis emerged from the correlation of circulating BCAA levels (valine and isoleucine) in serum with the corresponding levels in feces. Interestingly, serum BCAA levels were significantly higher in LOC1 individuals as compared to LOC2 individuals, which contrasted with the BCAA levels in the faecal metabolome (**Fig. 6A**). Thus, it is likely that the accumulation of BCAA in the feces of individuals of LOC2 was mediated by their gut microbiome. In contrast, due to the lower BCAA accumulation in feces and a higher BCAA biosynthesis by microbial species in LOC1, BCAA levels were observed to be in higher concentration in serum of LOC1 population, and hence higher BCAA absorption.

***Prevotella copri* regulates BCAA levels through threonine-independent isoleucine biosynthesis pathway**

To explore the differences in association of functional pathway modules between the two locations, KOs within each module were correlated with KOs from other modules using Spearman's correlation coefficient. The KOs showing significant differences in correlations between LOC1 and LOC2 were identified. This differential correlation analysis of BCAA biosynthetic modules with other pathways in LOC1 and LOC2 revealed that BCAA modules were independently driven in LOC1 and LOC2 (Spearman's rank correlation, FDR Adj. $P<0.01$) (**Fig. 6B and 6C**). To identify the species and the metabolic pathways that contributed most to the BCAA abundance in faecal and serum metabolome profiles, a correlation analysis with iterations leaving each species out was performed for each metabolic module (**Additional File 5: Figure S9**). The species whose removal leads to a maximum change in the correlation of metabolic pathway with metabolite was identified, and was considered as an important contributor of that metabolite [8]. Notably, a single species *Prevotella copri* was found driving the 'threonine-independent isoleucine biosynthesis' functional module. Among the other BCAA biosynthesis pathways, valine biosynthesis was also driven by species from *Prevotella*.

The correlation network analysis with different MGS/CAGs also revealed threonine-independent isoleucine biosynthesis pathway to be highly correlated with *Prevotella copri* in LOC1, and was the major pathway utilized by this species for BCAA biosynthesis (**Fig. 6D**). The first enzyme, D-citramalate synthase, catalysing the threonine-independent isoleucine biosynthesis pathway was also observed as highly enriched (LOR = 1.7) in LOC1. Further, BCAA biosynthesis was observed to be higher in LOC1 as compared to LOC2, and BCAA transporters were found higher in LOC2 as compared to LOC1 (**Fig. 6E**).

Discussion

Compositional and functional human gut microbiome studies in different populations have been instrumental in establishing the role of gut microbiome in human health [28, 37-39]. However, such population-specific signatures and functional insights for the Indian gut microbiome are yet unknown. Thus, the present work provides the first comprehensive survey of the Indian gut microbiome represented through a cohort of 110 individuals from two prominent locations. Several insights into the taxonomic and functional diversity emerged from the 16S rRNA and metagenomic analysis and were validated through metabolomic profiling, which is a prominent highlight of this study. Given the high diversity of diet and lifestyle in India, the selection of two distinct locations (Bhopal – LOC1, and Kerala – LOC2) as the representative sub-populations was an important consideration. The inclusion of LOC1 provided a representation of the population from North-Central India mainly consuming a carbohydrate and fat rich diet, whereas LOC2 represented a population from Southern India consuming an omnivorous diet with rice and animal-based products as the primary components.

This study established the gene catalogue of the Indian gut microbiome, which also exemplified its uniqueness. The genes encoding several transposons, peptidase, glucosidase, and plant polysaccharide degradation enzymes were unique to the Indian population and not represented in other microbiome datasets. This catalogue is likely to act as a reference dataset for gut microbiome studies in South-Asian populations, which have similar dietary habits and lifestyle, and for global comparative studies. Apart from the basic housekeeping functions of the microbiome, which were also found abundant in other datasets, the Indian gut microbiome was enriched in functions for carbohydrate and energy metabolism including degradation of complex polysaccharides, which corroborates well with the typical carbohydrate-rich diet of the Indian population [24]. The distant clustering of Indian samples from other populations revealed the unique composition of the Indian

gut microbiota (**Fig. 1B**). *Prevotella* emerged as the most discriminatory genus associated with the Indian population, as revealed by both amplicon and MGWAS. Its abundance was also indicated in the previous 16S rRNA-based microbiome studies of the Indian population, from small to medium-sized cohorts [21, 40]. Recently, *Prevotella* has been commonly observed in different non-Western communities who consume a plant-rich diet, such as in the Papua New Guineans, native Africans, rural Malawians, BaAka pygmies, etc. and has also been associated with vegetarianism in the Western populations [41-43]. However, it has not been observed at such high abundance in the western countries so far. The MGWAS approach in this study showed the presence of *Megasphaera*, *Lactobacillus* and *Mitsuokella* as the other major driver genera associated with the Indian microbiome.

Interestingly, the most abundant genus *Prevotella* in the Indian gut microbiome is a gram-negative bacterium from the phylum Bacteroidetes that typically releases lipopolysaccharides (LPS), a constituent of the bacterial outer membrane, from the dead bacterial cells, which can enter the circulation to elicit an inflammatory response through endotoxemia [44]. Several recent studies have shown a relationship between the abundance of specific strains of *Prevotella* with inflammatory diseases, since it has a higher intrinsic capacity to stimulate Th17-mediated inflammation, which is generally not expected in the strict commensal bacteria [41, 45]. However, the high abundance of *Prevotella* in the healthy gut microbiome of the Indian population does not corroborate with its potential inflammatory role reported so far. Further, the species *P. copri*, which is observed to be the most abundant in this study has been constantly reported to promote rheumatoid arthritis in different populations, which yet again is inconsistent with its high abundance in the healthy Indian population [46]. A probable explanation for this emerges from the understanding that the elicitation of an inflammatory response is mediated by a complex set of

interactions between host genetic risk factors and environment in which the presence of *Prevotella* may only be one of the factors [47]. Further, strain-level variations are known in the inflammatory responses and not all species of *Prevotella* could be potentially inflammatory, as also evident from the known high genetic diversity within and between the species of *Prevotella* [45]. Taken together, this description seemingly explains the high abundance of *Prevotella* in the healthy microbiota despite of its potential inflammatory properties, and emphasizes the requirement for larger cohort studies in different populations to gain deeper insights into the potential inflammatory roles of gut microbiome.

The abundance of *Prevotella* has been associated with plant-based diets, and the typical carbohydrate-rich diet of the Indian population could be one of the reasons for the over-representation of this genus in the Indian gut microbiome [48]. Likewise, the predominance of other microbial species from genus *Lactobacillus*, *Megasphaera* and *Mitsuokella* could be due to the higher intake of fermented food and dairy products along with the carbohydrate-rich diet in LOC1 [33, 48]. Similarly, *Bacteroides* and *Clostridium*, which were abundant in LOC2, are associated with diets rich in animal-based products, consistent with the omnivorous diet of LOC2 [37]. Interestingly, ET-1 and ET-2 enterotypes showed associations with the two locations LOC1 and LOC2, and also with the two KO-based clusters (C1 and C2) (**Additional File 5: Figure S5 and S6**). It is to be noted that C1 was enriched in enzymes involved in the degradation of carbohydrate and plant polysaccharides, which correlates well with the carbohydrate-rich diet in LOC1. In contrast, C2 was enriched in enzymes involved in lipid and protein degradation, which relate to the constituents of an omnivorous diet in LOC2. These observations further support the correlation between location, diet, and enterotype. Although, the concept of enterotype classification is sometimes criticised due to statistical weakness in some studies, however, a

statistically sound classification has the potential to be clinically relevant in various aspects such as disease diagnosis, early-detection of disease, biomarker development, personalised treatments and xenobiotic metabolism [19]. It is a representation of the major microbial species in the gut microbiome, and thus appears useful for microbiome-based population stratification. A robust statistical analysis with increased sample sizes, direct clinical associations, and detailed molecular interventions are essential for further strengthening its potential [38].

The study also established the previously unknown faecal metabolome of the Indian population, which showed strong clustering into three metabotypes differentiated by location and diet. The metabotypes also correlated well with the respective dietary habits of the two locations, where Metabotype-1 showed an association with LOC1 and was enriched in saturated fatty acids such as palmitic acid and stearic acid, whereas Metabotype-3 showed an association with LOC2, and was enriched in BCAAs such as isoleucine, valine and leucine, and SCFAs such as propionic acid, and butyric acid. A medium chain fatty acid (MCFA) ‘lauric acid’ was also found abundant in LOC2 perhaps due to the high dietary consumption of coconut oil in this location [49, 50]. Lauric acid has known health benefits such as preventing fat deposition in blood vessels and acting as an anti-inflammatory and anti-oxidative agent [51].

The major BCAA ‘isoluecine’ being produced through a less common threonine-independent pathway for isoleucine biosynthesis, and the higher enrichment of the key enzyme, D-citramalate synthase of the above pathway confirmed its higher abundance in LOC1 as compared to LOC2. Further, this pathway was found to be associated with a single species, *Prevotella copri* as reported earlier [36]. Taken together, it appears that at LOC1, the higher abundance of BCAA biosynthesis genes and a lower abundance of BCAA inward transporters in gut microbiome resulted in the lower BCAA accumulation in the gut microbiome, leading to a higher absorption and a higher

BCAA levels in serum, which was also supported by lower abundance of BCAA in faeces **(Fig. 7).** However, a contrasting pattern was observed in the case of LOC2, where the lower abundance of BCAA biosynthesis genes and the higher abundance of BCAA inward transporters correlated well with the higher and lower BCAA abundances in feces and serum, respectively.

The higher levels of SCFAs in LOC2 could be a consequence of the consumption of omnivorous diet, which is associated with a Firmicute-rich gut microbiome [31]. SCFAs now have well-established roles in human health as an energy source, an anti-inflammatory agent, and for improving intestinal homeostasis by increasing IL-18 production [52]. In contrast, higher serum BCAA levels have well-known roles in promoting insulin resistance and Type-2 Diabetes (T2D), and were found higher in the serum in LOC1. Several reports on the role of a high-fat diet in the modulation of microbiota and alteration in intestinal barrier are emerging, which results in the increased absorption and circulating levels of LPS and branched-chain amino acid (BCAA) and in the reduction of SCFAs such as butyrate, acetate, propionate, and secondary bile acids, as also noted in the case of LOC1 [44]. A high-fat and carbohydrate-rich diet have also been associated with an increase in abundance of Bacteroidetes (gram-negative bacteria), which reduces the abundance of Firmicutes leading to a skewed Bacteroidetes: Firmicutes ratio towards the former phylum [33]. Such a ratio was also apparent in this study in LOC1 dominated by *Prevotella* from the phylum Bacteroidetes [53].

Further, a several-fold increased risk of developing T2D has been found with the increase in circulating BCAA, which were also observed to be higher in LOC1 [36]. In contrast, secondary bile acids, which can activate glucagon-like peptide-1 (GLP1) secretion and help in protection against insulin resistance, were high in LOC2 [53]. These results correlate well with the known higher predisposition of the North-Indian population towards diabetes, cardiovascular diseases and

hypertension, as compared to Southern India. These observations also provide clues for the differential metabolic risks in the two populations due to the differences in dietary habits, which drive their characteristic microbiome. Many of the high-risk components such as trans-fat food (high-fat dairy, sweets and fried snacks) in North-Indian diets appear to be a reason for the higher prevalence of cardio-metabolic risk factors such as abdominal adiposity and hypertension, which are linked to the higher incidences of diabetes and cardiovascular diseases, and could be among one of the reasons for the shorter life-expectancy as compared to the South-Indian population [54, 55]. These metabolic diseases impose a drastic social, economic and health burden making India the World's diabetes capital and needs imperative measures for its control. In this scenario, the data and results from this study provides significant insights on the impact of diet on gut microbiome, which appears promising in reducing the metabolic risk factors originating through the interactions between diet and gut microbes to maintain a healthy gut flora, and necessitates the need for further studies to provide confirmatory evidences for the diet-gut microbiome mediated metabolic risks between the two populations.

This multi-omics based gut microbiome study of a healthy Indian population provides novel insights into the ecology and biogeography of the human gut microbiome from the poorly characterized Indian population, and their functional potential as determined by metagenomics and metabolomics. The comparison of the Indian gut microbiome with other available large-scale gut microbiome studies reveals the unique microbial community structures in the Indian population and demonstrates variations in the gut microbiome of Indians due to variation in location and dietary habits. The study also provides further evidence on the 'diet-gut microbiome-host metabolism axis' and confirms the notion that the gut microbiome is not just a passive substrate-degrading system but is actively involved in the host-microbiome crosstalk [56]. Further, the study

shows that an integrated approach using metabolomics and metagenomics is crucial for the identification of the repertoire of signals between microbiome and host, and in establishing the confounding factors for the gut-microbe-metabolic axis. The results from this study are also prospective to serve as a reference point for future epidemiological studies and translational applications.

Methods

Study design and subject enrolment

The study cohort consisted of 110 healthy individuals belonging to different age groups from infants (<1 year) to aged (>50 years), with an average subject age of 29.72 ± 17.4 years (mean ± sd) from two different locations across India i.e., Bhopal (LOC1, n=53) and Kerala (LOC2, n=57), which are separated by ~1000 miles. LOC1 was located in North-Central India with the majority of population being vegetarian, whereas LOC2 was located in Southern India where the population with dietary habits mostly consisting of rice, seafood and red meat (Diet description section in **Supplementary Table 1**). According to the 'Indian Food Composition Table', the primary Indian diet is rich in carbohydrates such as rice, wheat and potato, and in fat and proteins from milk and dairy products [55]. In addition, several accompaniments to the primary diet also exist including a variety of grains, vegetables, fruits, and usage of oil, spices and animal products.

The faecal samples for metagenomics and blood samples for serum metabolomics were collected from healthy participants and their metadata is provided in **Supplementary Data** under the Metadata information section. The recruitment of volunteers, sample collection, and other study-related procedures were carried out by following the guidelines and protocols approved by the Institute Ethics Committee of Indian Institute of Science Education and Research (IISER), Bhopal,

India. Each faecal sample was frozen within 30 mins of the collection. A written informed consent was obtained from all subjects prior to any study-related procedures, along with information on gender, age, and diet for a period of one month prior to the collection of faecal samples. The recruited individuals did not undergo any medication at least one month prior to the sample collection. All the recruited individuals had an average BMI of 21.16 (±5.23), and were not diagnosed with T2D at the time of sample collection, and did not have a second-degree relative history of T2D. The above samples were then used for 16S rRNA V3 hypervariable region amplicon sequencing, shotgun metagenomic sequencing, and metabolomic analysis.

Faecal metagenomic DNA extraction

Metagenomic DNA was isolated from all the faecal samples using QIAamp Stool Mini Kit (Qiagen, CA, USA) according to the manufacturer's instructions. DNA concentration was estimated by Qubit HS dsDNA assay kit (Invitrogen, CA, USA), and quality was estimated by agarose gel electrophoresis. All the DNA samples were stored at −80 °C until sequencing.

16S rRNA amplicon and shotgun metagenome sequencing

The extracted DNA (5ng) was PCR amplified with seven different custom modified 5′-end adaptor-ligated 341F and 534R primers (See the primer details section in **Supplementary Data**) targeting the V3 hypervariable region of 16S rRNA gene. After evaluating the amplified products on 2% w/v agarose gel, the products were purified using Ampure XP kit (Beckman Coulter, Brea, CA USA). Amplicon libraries were prepared by following the Illumina 16S metagenomic library preparation guide. Metagenomic libraries were prepared using Illumina Nextera XT sample preparation kit (Illumina Inc., USA) by following the manufacturer's protocol. Library size of all the libraries was assessed using Agilent 2100 Bioanalyzer (Agilent Technologies, Santa Clara, USA.), and quantified on a Qubit 2.0 fluorometer using Qubit dsDNA HS kit (Life technologies,

USA) and by qPCR using KAPA SYBR FAST qPCR Master mix and Illumina standards and primer premix (KAPA Biosystems, Wilmington, MA, USA) following the Illumina suggested protocol. Both the amplicon and metagenomic libraries were loaded on Illumina NextSeq 500 platform using NextSeq 500/550 v2 sequencing reagent kit (Illumina Inc., USA), and 150 bp paired-end sequencing was performed at the Next-Generation Sequencing (NGS) Facility, IISER Bhopal, India.

Amplicon-based taxonomic analysis

A total of 24 Gbps of data were retrieved on de-multiplexing of paired-end reads with an average of 210 Mbp per sample. The paired-end reads were assembled using FLASH and were quality filtered at Q20 (80% bases) Phred quality score, and the primer sequences were trimmed from the High Quality (HQ) reads [57]. The reads were further clustered into OTUs using closed-reference OTU picking protocol of QIIME at ≥97% identity against Greengenes Database v 13_5 [58, 59]. The most abundant read was selected as the representative sequence for each OTU and was assigned with taxonomy using the Greengenes database. OTU table containing the abundance of each OTU for each sample was generated and used for further analysis. For phylogenetic analysis, representative 16S rRNA of phylotypes were aligned against a core set of 16S rRNA gene sequences in Greengenes database using align_seqs.py with the PyNAST algorithm [60]. The phylogenetic distances between reads were calculated using aligned dataset and were used for the calculation of unweighted UniFrac distances.

Pre-processing of the Metagenomic reads

A total of 150 Gbp of metagenomic sequence data (mean = 1.36 Gb) was generated from 110 faecal samples. The metagenomic reads were filtered using NGSQC toolkit with a cutoff ≥Q20 [61]. The high-quality reads were further filtered to remove the host-origin reads (human

contamination) from bacterial metagenomic reads, which resulted in the removal of an average of 1% reads. The reads from each sample were assembled into contigs at a k-mer size of 63 bp using SOAPdenovo [62]. The singletons resulting from each sample were pooled together and denovo assembly was repeated on the combined set of singleton reads from all samples. The ORFs from each contig (length ≥ 300bp) were predicted using MetaGeneMark [63]. Pair-wise alignment of genes was performed using BLAT, and the genes which had an identity ≥ 95% and alignment coverage ≥ 90% were clustered into a single set of non-redundant genes, from which the longest gene was selected as the representative ORF to construct the non-redundant gene catalog.

Integrated Gene Catalog (IGC), which represents 1,297 human gut metagenomic samples comprising of HMP, MetaHIT and Chinese datasets, was retrieved [23]. The gene catalog constructed from Indian samples was combined with the IGC to construct a non-redundant gene catalog (using identity ≥ 95% and alignment coverage ≥ 90%) and is referred to as 'updated IGC' in the subsequent analysis.

Quantification of gene content

The quantification of gene content was carried out using the strategy performed by Qin et al., [7] where the high-quality reads were aligned against the updated IGC using SOAP2 in SOAP aligner with an identity cut off ≥ 90% [64]. Two types of alignments were considered for sequence-based profiling:

(1) The entire paired-end read mapped to the gene.
(2) One end of paired-end read mapped to a gene and other end remained unmapped.

In both cases, the mapped read was counted as one copy. Further, the read count was normalized based on length of the gene as: $bi = \frac{xi}{Li}$

The relative abundance of a gene within the sample was calculated as: $ai = \frac{bi}{\sum jbj} = \frac{\frac{xi}{Li}}{\sum j\frac{xj}{Lj}}$

a_i: relative abundance of gene in sample S; x_i: The times in which gene i was detected in sample S (the number of mapped reads); L_i: length of gene i; b_i: copy number of gene i in sequenced data from sample S.

Phylogenetic assignment of reads

A total of 4,097 reference microbial genomes were obtained from Human Microbiome Project (HMP) and National Centre for Biotechnology Information (NCBI) on 5th December 2015. The databases were independently indexed into two Bowtie indexes using Bowtie-2 [65]. The metagenomic reads were aligned to the reference microbial genomes using Bowtie-2. The mapped reads from both indexes were merged by selecting the alignment having the higher identity (≥ 90% identity). The percent identity was calculated using the formula: %identity = 100*(matches/total aligned length). The normalized abundance of a microbial genome was calculated by summing the total number of reads aligned to its reference genome, normalized by the genome length and the total number of reads in the dataset. For reads showing hits to both indexed databases with equal identity, each genome was assigned 0.5 read count. The relative abundance of each genome was calculated by adding the normalized abundance of each genome divided by the total abundance. The Calinski Harabasz index (CHI) was used to calculate the variance between the clusters compared to the variance within clusters [2].

Construction of common core microbial functions

To identify the core microbial functions in the gut microbiome of Indian populations and to understand their abundance compared to the other populations, the core microbiome was constructed using a similar strategy as mentioned in MetaHIT [2]. However, to construct a

comprehensive core functional microbiome, the information of essential functions from six different microbes including two strains of *Escherichia coli*, *Bacteroides thetaiotaomicron*, *Pseudomonas aeruginosa*, *Salmonella enteric* and *Staphylococcus aureus*, was used instead of considering a single microorganism. The list of essential genes was collected from DEG database v [66]. 1,890 genes were identified as essential genes in all the six microorganisms. The core gut microbiome functions were also calculated using the above strategy for the USA, Denmark and Chinese population gut microbial samples to remove the variations arising due to differences in data analysis procedures. Apart from identifying the clusters that represented ≥85% genes within the range of essential gene functions, the low prevalent eggNOG functions, which were present in ≥ 0.0001% abundance in ≥ 80% of samples in that population, were further filtered out. This added filtration step helped in removing all the low abundant functions. To represent the core, the variance of these functions was also calculated between the two Indian locations. The eggNOGs which showed significant deviations in variations (P-value≤ 0.05; Levene's test) were further filtered out from the analysis.

Construction of Metagenomic Species for MGWAS

To identify metagenomic markers using a non-reference based approach on metagenomic samples, a metagenome-wide association study was performed for 340 samples (age and gender matched) including India (both locations), USA, China and Denmark populations. The genes present in at least ≥10% of samples were considered and clustered using the canopy-mgs algorithm as described [7]. The genes having Pearson's correlation coefficient (≥0.9) were clustered into CAGs. Furthermore, the genes for which ≥ 90% abundance was obtained from a single sample were discarded.

To determine the taxonomic origin of each MGS/CAG (metagenomic cluster), all the genes were aligned against reference microbial genomes of 4,097 genomes from HMP and NCBI at nucleotide level using BLASTN. The alignment hits were filtered using an E-value $\leq 10^{-6}$ and alignment coverage ≥ 80% of the gene length, and 2,687,688 genes showed alignments against the reference genomes. The remaining genes were aligned against UNIREF database (UniRef 50) at protein sequences [67]. The multiple best hits with equal identity and scores were further assigned taxonomy based on LCA (Lowest Common Ancestor) method. The genes were finally assigned to taxa based on comprehensive parameters of sequence similarity across phylogenetic ranks as described earlier [68]. The identity threshold of ≥95% was used for assignment up to species level, ≥85% identity threshold for assignment up to genus level, and ≥65% identity was used for phylum level assignment using BLASTN. The taxonomic assignments of MGS/CAGs were performed with the criteria that ≥50% genes in each MGS should map to the same lowest phylogenetic group. So if a particular species is assigned ≥50% genes out of total the assignment will be carried out at species level rather than at genus or higher orders. The relative abundance of MGS/CAGs in each sample was estimated by using relative abundance values of all genes from that MGS/CAG. A Poisson distribution was fitted to the relative abundance values of the data. The mean estimated from Poisson distribution was assigned as the relative abundance of that MGS. The profile of MGS/CAGs were generated and used for further analysis.

Faecal and Serum metabolomic sample preparation and derivatization

Lyophilized faecal samples were used to achieve better metabolite coverage, as described previously [69]. Metabolites were extracted with 1 mL of ice-cold methanol: water (8:2) from 80 mg of lyophilized samples in a bath ultrasonicator (Bioruptor ™ UCD-200, Diagenode, USA) at 4°C for 30 min followed by 2 min of vortexing. The supernatant was extracted by centrifugation

at 18,000 g for 15 min at 4°C and dried at 50°C under a gentle stream of nitrogen gas. To remove the residual water molecules from the samples, 100uL of toluene was added to the dry residue and evaporated completely at 50°C under nitrogen gas. Dry extracted metabolites were first derivatized with 50 uL of methoxyamine hydrochloride (MOX) in pyridine (20 mg/mL) at 60°C for 2 hours, and the second derivatization was performed with 100 uL of MSTFA in 1% TMCS at 60°C for 45 min to form trimethylsilyl (TMS) derivatives. Finally, 150 uL of the TMS derivatives was transferred into a GC glass vial inserts and subjected to GC/TOFMS analysis. Serum samples were prepared (polar metabolites only) and derivatized as described by Psychogium et al., 2011 [70].

Method development and validation

Matrix dilution approach was used for validating the linearity and range of dilution [69]. Pooled faecal samples were used to create the reference peaks to validate the peaks coming from individual samples, which were needed due to the presence of a relatively high abundance of faecal metabolites in the pooled samples. The supernatant of feces after extraction was serially diluted 2, 5, 10, 50, 100, 200 and 500 times with methanol: water (8:2). At dilution 2, the maximum numbers of peaks were seen and were processed with the same dilution factor for all the samples. A total of 30 chemical standards mixture and the pooled faecal samples were used to validate the method. Each stock solution of test standard was carefully prepared in deionized water or with pure ethanol (50,150 350, 500 um) for the determination of linear range, regression coefficient (R2), limit of detection (LOD), and repeatability. L-norvaline (1, 2.5, 5, 10, 20 mg/ml in ethanol) was used as a spiked external standard for the optimized derivatization of the method.

GC-MS analysis

GC-MS was performed on an in-house Agilent 7890A gas chromatograph with 5975C MS system. An HP-5 (25 m × 320 um × 0.25 um i.d.) fused silica capillary column (Agilent J&W Scientific,

Folsom, CA), was used with the open split interface. The injector, transfer line and ion source temperatures were maintained at 220, 220 and 250 °C, respectively. Oven temperature was programmed at 70°C for 0.2 min, and increased at 10°C/min to 270°C where it was sustained for 5 min, and further increased at 40°C/min to 310°C where it was held for 11 minutes. The MS was operated in the electron impact ionization mode at 70eV. Mass data were acquired in full scan mode from m/z 40 to 600 with an acquisition rate of 20 spectra per second. To detect retention time shifts and enable Kovats retention index (RI) calculation, a standard Alkane series mixture (C10–C40) was injected periodically during the sample analysis. RIs are relative retention times normalized to n-alkanes eluted adjacently. For serum samples, we used 2uL aliquot with a split ratio of 4:1 on the same column as described above. The injector port temperature was held at 250°C, and the helium gas flow rate was set to 1mL/min at an initial oven temperature of 50°C. The oven temperature was increased at 10°C/min to 310°C for 11min and mass data were acquired in full scan mode from m/z 40 to 600 with an acquisition rate of 20 spectra per second.

Metabolomic analysis and metabolite profile generation

Raw CDF files were used for peak identification and filtering and the XCMS package in R were used for pre-processing of the peaks. First, the parameters used for pre-processing of the reads were optimized by calculating the reliability index using the formula given below:

Reliability index = (number of reliable peaks)^2 /number of unreliable peaks.

The reliable peaks were identified for each of the settings such as fwhm, S/N and bw, with a predefined range of values and regression coefficient was calculated for dilutions of QC samples. The number of peaks with a high coefficient of determination ($R^2 \geq 0.9$) were considered reliable, whereas the peaks with very low R^2 (≤ 0.05) were considered unreliable peaks[71]. The finally optimized parameters were: profmethod = bin, method = matched Filter, fwhm =8 and 5 for

faecal and serum samples, respectively, and S/N = 12 and 3 for faecal and serum samples, respectively, bw =5 (for first grouping), smooth = linear, family = gaussian, extra = 1, plot type = mdevden, missing =8, bw = 3 (for second grouping). Further, in order to compare across multiple samples, the peak intensities were normalized (root transformed) and scaled using z-transformation. These normalized and scaled peak intensities were used for further statistical analysis.

A multivariate statistical method, Orthogonal Projections to Latent Structures Discriminant Analysis (OPLS-DA), was used to identify differences between LOC-1 samples (n=53) and LOC-2 (n=55) samples. Metabolites driving the differences were identified in metabolic profiles of LOC-1 and LOC-2 samples using correlations coefficients. The clusters of co-abundant metabolite profiles were identified using R package "WGCNA". Signed weighted metabolite co-abundance correlation after scaling and centering was calculated across all samples. The soft threshold of $\beta = 15$ was chosen for scale-free topology. The dynamic hybrid tree cutting algorithm was used to identify the clusters with a deepsplit = 4 and minimum cluster size = 4. The profile of each faecal metabolite cluster was summarized using eigenvector. The abundance profile of each cluster of metabolites (MES) was calculated using the same methodology as used for MGS cluster abundance profiles.

Retention index (RI) calculation

GC-MS data obtained from the alkene series run was used to calculate the RI for each peak in the samples, and the obtained RI values were further used at the time of library search for the identification of individual metabolite.

$$I = 100\ X\ [n + (logtx - \log tn)/\ (\log tn + 1 - \log tn)$$

Where, tx = retention time of the peak, tn = retention time of preceding alkane, and tn+1 = retention time of the following alkane.

Enterotype Analysis

Enterotypes in the dataset were identified from the relative abundance profiles of Genus or Orthologous groups (OG) in the samples. The Jensen-Shannon distances (which estimates the probability distributions) between the samples were calculated and the abundance profiles were clustered using PAM (partitioning around medoids) clustering algorithm as mentioned previously [72]. The optimal number of clusters was assessed using CHI that has shown good performance in recovering the optimal number of clusters. Similarly, the prediction strength was also employed as another metric for cluster validation. Both the CHI and prediction strength showed quite significantly correlated results. For clustering, CHI and prediction strength gave non-identical values, silhouette index was calculated to estimate the robustness of clusters.

Between class analysis

Between class analysis was performed to identify the drivers and support the clustering of the genus/species/OG abundance profiles into enterotypes. The instrumental variables were the enterotype classification and the top species, which contributed the maximum to the principal components obtained from between class analysis, and were identified as driver species/genus/OG based on their factor scores.

Diversity Analysis

The within-sample diversity metrics such as a number of observed species, Shannon index, and Phylogenetic distance were calculated for each rarefied sample (at fixed or varying depths) and were compared to different types of samples. The beta diversity (between the samples) was

calculated using unweighted UniFrac distances between the samples for rarefied OTU tables. The effect of covariates such as age, enterotypes, diet, geography and gender were compared for correlation with principal components identified from principal component analysis using UniFrac distances. The polyserial correlations with P-values were calculated for categorical variables and the significance of the covariates for explaining the variation was estimated at each principal component.

Network Analysis

Spearman's rank correlations were computed between each of the species/MGS and the between MGS and functional modules/metabolites. The correlations with significant P-values were selected and were used for the network analysis. The undirected links were generated between correlated nodes (species/KOs/modules) and the strength of the links were given weights based on their correlation coefficients. The network structure was generated using "igraph" package in R. The modularity of the network for KOs association was generated with each module representing the functional modules defined in KEGG database. The negative correlation was not considered in generating the network modules. Moreover, the positive correlations were filtered ($\rho \geq 0.6$) for most of the network analysis.

Supervised learning

Predictive models were built using supervised machine learning algorithm Random Forest (RF). The models were optimized using 10,000 trees and default settings of mtry (number for variables used to build the model). The mean three-fold cross-validation error rates were calculated for each of the binary tree and the ensemble of trees. The mean decrease in accuracy, which is the increase in error rates on leaving the variable out, was calculated for each prediction and tree and was used to estimate the importance score. The variables showing a higher mean decrease in accuracy of

prediction were considered important for the segregation of the datasets into groups based on the categorical variable.

Statistical Analysis

All the statistical comparisons between groups were performed using non-parametric Wilcoxon Rank Sum Test with FDR Adjusted P-Values to control for multiple comparisons. The correlations between two variables and the correlations within were calculated using Spearman's Correlation Coefficient with Adjusted P-Values. The correlations between categorical and numeric variables were performed using Polyserial correlation/biserial correlations. To identify the enrichment of enzymes/species associated with a host, Odds Ratio was used as a measure of the enrichment of an enzyme in a host. The Odds Ratio was calculated as $OR(k) = [\sum_{s=LOC1} A_{sk} / \sum_{s=LOC1}(\sum_{i \neq k} A_{si})] / [\sum_{s=LOC2} A_{sk} / \sum_{s=LOC2} (\sum_{i \neq k} A_{si})]$, where A_{sk} denotes abundance of enzyme k in sample S. Apart from that, Reporter features algorithm was used for gene-set analysis of significant pathways associated with different groups of samples. The algorithm takes the adjusted P-values and folds changes (log odds ratio) as input for each KO. The gene statistic is calculated based on the significant association of KO and its direction of change through which the pathway is scored by calculating the global P-value. All the graphs and plots were generated using the ggplot2 package in R.

Correlation analysis between functional modules and metabolite clusters

To calculate the association of microbial functional modules with faecal metabolite clusters, the Spearman's correlation coefficients were calculated to rank KOs for association with metabolite clusters and Metabotypes. To quantify the shift in Spearman correlation between given KEGG module and the metabolite cluster compared to the background distribution, the background adjusted median Spearman's correlation was calculated for a given KEGG module m as:

$$SCC_{bg.adj} = \text{median}\,(SCC_{KOs \in \text{KEGG Module m}}) - \text{median}\,(SCC_{KOs\ \text{KEGG Module m}})$$

Where SCC_{KO} is the partial Spearman's correlation coefficient between KO and the metabolite cluster.

Identification of microbial species driving the association between KEGG Module and metabolite abundance was done by iterating the correlation between KO belonging to the KEGG module and the metabolite after excluding the genes annotated to that KO from each species. The change in median Spearman’s correlation coefficient between the KOs and the metabolite, when genes from that species are excluded from the analysis, was calculated as described previously [36]. The species showing the maximum change in the overall correlation of module with metabotype was plotted.

List of abbreviations

Indian Gut Microbiome (IGM), Enterotypes (ET), Integrated Gene Catalog (IGC), Metagenome-Wide Association Study (MGWAS), Short Chain Fatty Acids (SCFAs), Branched Chain Amino Acids (BCAAs).

Declarations

Collection of Datasets for Comparative analysis

The 74 HMP metagenomes were collected from http://hmpdacc.org/HMASM or NCBI SRA (accession SRR059347). The 85 Danish fecal metagenomes from METAHIT were obtained from European Nucleotide Archive (http://www.ebi.ac.uk/ena, study accession number ERP000108). The 71 Chinese metagenome samples were obtained from NCBI SRA (accession number – SRR341581).

Ethics approval and consent to participate

The recruitment of volunteers, sample collection, and other study-related procedures were carried out by following the guidelines and protocols approved by the Institute Ethics Committee of Indian Institute of Science Education and Research (IISER), Bhopal, India. A written informed consent was obtained from all the subjects prior to any study-related procedures.

Consent for publication

Not applicable

Availability of data and materials

The datasets generated and/or analysed during the current study have been deposited in the National Centre for Biotechnology Information (NCBI) BioProject database under the project number PRJNA397112 and will be made publicly available on publication or on request at the time of peer review.

Competing interests

The authors declare that they have no competing interests.

Funding

This work was supported by the intramural funding received from IISER Bhopal, Madhya Pradesh, India.

Author’s contributions

VKS and AM conceived the work and participated in the design of the study. AM designed the study protocol. AM and JP collected all the samples in collaboration with TG. AM performed the all sample processing, DNA extraction, metabolite extraction and profiling from faecal and blood samples. RS and AM carried out the library preparation and sequencing work. DBD carried out all metagenomic data and statistical analysis. AKS and DBD analyzed the metabolomics data. AM

and DBD did the primary data interpretation of analytical outcomes under the supervision of VKS. AM, DBD, RS, AG, JS, KRA and VKS drafted the manuscript. All authors read and approved the final manuscript.

Acknowledgments

We thank MHRD, Govt of India and Centre for Research on Environment and Sustainable Technologies (CREST) at IISER Bhopal for providing financial support. However, the views expressed in this manuscript are that of the authors alone and no approval of the same, explicit or implicit, by MHRD should be assumed. The sequencing and computational analysis were performed at the NGS Facility and HPC and computing facility, respectively, at IISER Bhopal. DBD, AM, RS and JP received fellowships from the UGC (University Grants Commission), Centre for Research on Environment and Sustainable Technologies (CREST, IISER Bhopal), DST-INSPIRE and Central University of Kerala, respectively.

References:

1. Turnbaugh PJ, Ley RE, Hamady M, Fraser-Liggett CM, Knight R and Gordon JI. The human microbiome project. Nature. 2007;449 7164:804.
2. Arumugam M, Raes J, Pelletier E, Le Paslier D, Yamada T, Mende DR, et al. Enterotypes of the human gut microbiome. nature. 2011;473 7346:174.
3. Yatsunenko T, Rey FE, Manary MJ, Trehan I, Dominguez-Bello MG, Contreras M, et al. Human gut microbiome viewed across age and geography. nature. 2012;486 7402:222.
4. Dominguez-Bello MG, Costello EK, Contreras M, Magris M, Hidalgo G, Fierer N, et al. Delivery mode shapes the acquisition and structure of the initial microbiota across multiple body habitats in newborns. Proceedings of the National Academy of Sciences. 2010;107 26:11971-5.
5. Saxena R and Sharma VK. A Metagenomic Insight Into the Human Microbiome: Its Implications in Health and Disease. In: Kumar D and Antonarakis S, editors. Medical and Health Genomics. Mica Haley, Academic Press, Elsevier; 2016. p. 107-19.
6. Schwiertz A, Taras D, Schäfer K, Beijer S, Bos NA, Donus C, et al. Microbiota and SCFA in lean and overweight healthy subjects. Obesity. 2010;18 1:190-5.
7. Qin J, Li Y, Cai Z, Li S, Zhu J, Zhang F, et al. A metagenome-wide association study of gut microbiota in type 2 diabetes. Nature. 2012;490 7418:55.
8. Pedersen HK, Gudmundsdottir V, Nielsen HB, Hyotylainen T, Nielsen T, Jensen BA, et al. Human gut microbes impact host serum metabolome and insulin sensitivity. Nature. 2016;535 7612:376-81.
9. Gupta VK, Paul S and Dutta C. Geography, ethnicity or subsistence-specific variations in human microbiome composition and diversity. Frontiers in microbiology. 2017;8:1162.
10. Bhute S, Pande P, Shetty SA, Shelar R, Mane S, Kumbhare SV, et al. Molecular characterization and meta-analysis of gut microbial communities illustrate enrichment of Prevotella and Megasphaera in Indian subjects. Frontiers in microbiology. 2016;7:660.
11. Gomez A, Petrzelkova KJ, Burns MB, Yeoman CJ, Amato KR, Vlckova K, et al. Gut Microbiome of Coexisting BaAka Pygmies and Bantu Reflects Gradients of Traditional Subsistence Patterns. Cell reports. 2016;14 9:2142-53. doi:10.1016/j.celrep.2016.02.013.
12. Obregon-Tito AJ, Tito RY, Metcalf J, Sankaranarayanan K, Clemente JC, Ursell LK, et al. Subsistence strategies in traditional societies distinguish gut microbiomes. Nature communications. 2015;6:6505. doi:10.1038/ncomms7505.
13. Häsler R, Kautz C, Rehman A, Podschun R, Gassling V, Brzoska P, et al. The antibiotic resistome and microbiota landscape of refugees from Syria, Iraq and Afghanistan in Germany. Microbiome. 2018;6 1:37.
14. Pulikkan J, Maji A, Dhakan DB, Saxena R, Mohan B, Anto MM, et al. Gut Microbial Dysbiosis in Indian Children with Autism Spectrum Disorders. Microbial ecology.1-13.
15. Mohan V, Sandeep S, Deepa R, Shah B and Varghese C. Epidemiology of type 2 diabetes: Indian scenario. Indian journal of medical research. 2007;125 3:217.
16. Organization WH. Waist circumference and waist-hip ratio: report of a WHO expert consultation, Geneva, 8-11 December 2008. 2011.
17. Schmidt TS, Raes J and Bork P. The human gut microbiome: from association to modulation. Cell. 2018;172 6:1198-215.
18. Maji A, Misra R, Dhakan DB, Gupta V, Mahato NK, Saxena R, et al. Gut microbiome contributes to impairment of immunity in pulmonary tuberculosis patients by alteration of butyrate and propionate producers. Environmental Microbiology. 2017.

19. Costea PI, Hildebrand F, Arumugam M, Backhed F, Blaser MJ, Bushman FD, et al. Enterotypes in the landscape of gut microbial community composition. Nature microbiology. 2018;3 1:8-16. doi:10.1038/s41564-017-0072-8.

20. Shetty SA, Marathe NP and Shouche YS. Opportunities and challenges for gut microbiome studies in the Indian population. Microbiome. 2013;1 1:24.

21. Tandon D, Haque MM, R S, Shaikh S, P S, Dubey AK, et al. A snapshot of gut microbiota of an adult urban population from Western region of India. PLoS One. 2018;13 4:e0195643. doi:10.1371/journal.pone.0195643.

22. Suryanarayana M, Agrawal A and Prabhu KS. Inequality-adjusted human development index for India's states. New Delhi, India: United Nations Development Programme. 2011.

23. Li J, Jia H, Cai X, Zhong H, Feng Q, Sunagawa S, et al. An integrated catalog of reference genes in the human gut microbiome. Nature biotechnology. 2014;32 8:834.

24. Qin J, Li R, Raes J, Arumugam M, Burgdorf KS, Manichanh C, et al. A human gut microbial gene catalogue established by metagenomic sequencing. Nature. 2010;464 7285:59-65. doi:10.1038/nature08821.

25. Wang J and Jia H. Metagenome-wide association studies: fine-mining the microbiome. Nature Reviews Microbiology. 2016;14 8:508.

26. Qin J, Li R, Raes J, Arumugam M, Burgdorf KS, Manichanh C, et al. A human gut microbial gene catalogue established by metagenomic sequencing. nature. 2010;464 7285:59.

27. Tyakht AV, Kostryukova ES, Popenko AS, Belenikin MS, Pavlenko AV, Larin AK, et al. Human gut microbiota community structures in urban and rural populations in Russia. Nature communications. 2013;4:2469.

28. Liang C, Tseng H-C, Chen H-M, Wang W-C, Chiu C-M, Chang J-Y, et al. Diversity and enterotype in gut bacterial community of adults in Taiwan. BMC genomics. 2017;18 1:932.

29. Aleksandrowicz L, Tak M, Green R, Kinra S and Haines A. Comparison of food consumption in Indian adults between national and sub-national dietary data sources. British Journal of Nutrition. 2017;117 7:1013-9.

30. Joy EJ, Green R, Agrawal S, Aleksandrowicz L, Bowen L, Kinra S, et al. Dietary patterns and non-communicable disease risk in Indian adults: secondary analysis of Indian Migration Study data. Public health nutrition. 2017;20 11:1963-72.

31. Ríos-Covián D, Ruas-Madiedo P, Margolles A, Gueimonde M, de los Reyes-Gavilán CG and Salazar N. Intestinal short chain fatty acids and their link with diet and human health. Frontiers in microbiology. 2016;7:185.

32. Kovatcheva-Datchary P, Nilsson A, Akrami R, Lee YS, De Vadder F, Arora T, et al. Dietary fiber-induced improvement in glucose metabolism is associated with increased abundance of Prevotella. Cell metabolism. 2015;22 6:971-82.

33. De Filippo C, Cavalieri D, Di Paola M, Ramazzotti M, Poullet JB, Massart S, et al. Impact of diet in shaping gut microbiota revealed by a comparative study in children from Europe and rural Africa. Proceedings of the National Academy of Sciences. 2010;107 33:14691-6.

34. Ruiz-Capillas C and Jimenez-Colmenero F. Biogenic amines in meat and meat products. Critical reviews in food science and nutrition. 2004;44 7-8:489-99.

35. Layman DK. The role of leucine in weight loss diets and glucose homeostasis. The Journal of nutrition. 2003;133 1:261S-7S.

36. Pedersen HK, Gudmundsdottir V, Nielsen HB, Hyotylainen T, Nielsen T, Jensen BA, et al. Human gut microbes impact host serum metabolome and insulin sensitivity. Nature. 2016;535 7612:376.

37. Wu GD, Chen J, Hoffmann C, Bittinger K, Chen Y-Y, Keilbaugh SA, et al. Linking long-term dietary patterns with gut microbial enterotypes. Science. 2011;334 6052:105-8.

38. Arumugam M, Raes J, Pelletier E, Le Paslier D, Yamada T, Mende DR, et al. Enterotypes of the human gut microbiome. Nature. 2011;473 7346:174-80. doi:10.1038/nature09944.

39. Cho I and Blaser MJ. The human microbiome: at the interface of health and disease. Nat Rev Genet. 2012;13 4:260-70. doi:10.1038/nrg3182.

40. Bhute S, Pande P, Shetty SA, Shelar R, Mane S, Kumbhare SV, et al. Molecular characterization and meta-analysis of gut microbial communities illustrate enrichment of Prevotella and Megasphaera in Indian Subjects. Frontiers in microbiology. 2016;7.

41. Ley RE. Gut microbiota in 2015: Prevotella in the gut: choose carefully. Nature reviews Gastroenterology & hepatology. 2016;13 2:69-70. doi:10.1038/nrgastro.2016.4.

42. Kovatcheva-Datchary P, Nilsson A, Akrami R, Lee YS, De Vadder F, Arora T, et al. Dietary Fiber-Induced Improvement in Glucose Metabolism Is Associated with Increased Abundance of Prevotella. Cell Metab. 2015;22 6:971-82. doi:10.1016/j.cmet.2015.10.001.

43. Martinez I, Stegen JC, Maldonado-Gomez MX, Eren AM, Siba PM, Greenhill AR, et al. The gut microbiota of rural papua new guineans: composition, diversity patterns, and ecological processes. Cell Rep. 2015;11 4:527-38. doi:10.1016/j.celrep.2015.03.049.

44. Hawkesworth S, Moore S, Fulford A, Barclay G, Darboe A, Mark H, et al. Evidence for metabolic endotoxemia in obese and diabetic Gambian women. Nutrition & diabetes. 2013;3 8:e83.

45. Larsen JM. The immune response to Prevotella bacteria in chronic inflammatory disease. Immunology. 2017;151 4:363-74. doi:10.1111/imm.12760.

46. Scher JU, Sczesnak A, Longman RS, Segata N, Ubeda C, Bielski C, et al. Expansion of intestinal Prevotella copri correlates with enhanced susceptibility to arthritis. eLife. 2013;2:e01202. doi:10.7554/eLife.01202.

47. Renz H, von Mutius E, Brandtzaeg P, Cookson WO, Autenrieth IB and Haller D. Gene-environment interactions in chronic inflammatory disease. Nat Immunol. 2011;12 4:273-7. doi:10.1038/ni0411-273.

48. Tremaroli V and Bäckhed F. Functional interactions between the gut microbiota and host metabolism. Nature. 2012;489 7415:242.

49. Jaarin K, Norliana M, Kamisah Y, Nursyafiza M and Qodriyah HMS. Potential role of virgin coconut oil in reducing cardiovascular risk factors. Exp Clin Cardiol. 2014;20 8:3399-410.

50. Boemeke L, Marcadenti A, Busnello FM and Gottschall CBA. Effects of coconut oil on human health. Open Journal of Endocrine and Metabolic Diseases. 2015;5 07:84.

51. Intahphuak S, Khonsung P and Panthong A. Anti-inflammatory, analgesic, and antipyretic activities of virgin coconut oil. Pharmaceutical biology. 2010;48 2:151-7.

52. Nicholson JK, Holmes E, Kinross J, Burcelin R, Gibson G, Jia W, et al. Host-gut microbiota metabolic interactions. Science. 2012:1223813.

53. Boulangé CL, Neves AL, Chilloux J, Nicholson JK and Dumas M-E. Impact of the gut microbiota on inflammation, obesity, and metabolic disease. Genome medicine. 2016;8 1:42.

54. Joshi SR and Parikh RM. India; the diabetes capital of the world: Now heading towards hypertension. Journal-Association of Physicians of India. 2007;55 Y:323.

55. Longvah T, Ananthan R, Bhaskarachary K and Venkaiah K. Indian food composition tables. National Institute of Nutrition, Indian Council of Medical Research, Department of Health Research, Ministry of Health and Family Welfare, Government of India (505 pp). 2017.

56. Li X, Shimizu Y and Kimura I. Gut microbial metabolite short-chain fatty acids and obesity. Bioscience of microbiota, food and health. 2017;36 4:135-40. doi:10.12938/bmfh.17-010.

57. Magoč T and Salzberg SL. FLASH: fast length adjustment of short reads to improve genome assemblies. Bioinformatics. 2011;27 21:2957-63.

58. Ritari J, Salojärvi J, Lahti L and de Vos WM. Improved taxonomic assignment of human intestinal 16S rRNA sequences by a dedicated reference database. BMC genomics. 2015;16 1:1056.

59. Caporaso JG, Kuczynski J, Stombaugh J, Bittinger K, Bushman FD, Costello EK, et al. QIIME allows analysis of high-throughput community sequencing data. Nature methods. 2010;7 5:335.
60. Caporaso JG, Bittinger K, Bushman FD, DeSantis TZ, Andersen GL and Knight R. PyNAST: a flexible tool for aligning sequences to a template alignment. Bioinformatics. 2009;26 2:266-7.
61. Patel RK and Jain M. NGS QC Toolkit: a toolkit for quality control of next generation sequencing data. PloS one. 2012;7 2:e30619.
62. Li R, Li Y, Kristiansen K and Wang J. SOAP: short oligonucleotide alignment program. Bioinformatics. 2008;24 5:713-4.
63. Zhu W, Lomsadze A and Borodovsky M. Ab initio gene identification in metagenomic sequences. Nucleic acids research. 2010;38 12:e132-e.
64. Li R, Yu C, Li Y, Lam T-W, Yiu S-M, Kristiansen K, et al. SOAP2: an improved ultrafast tool for short read alignment. Bioinformatics. 2009;25 15:1966-7.
65. Langmead B and Salzberg SL. Fast gapped-read alignment with Bowtie 2. Nature methods. 2012;9 4:357.
66. Zhang R and Lin Y. DEG 5.0, a database of essential genes in both prokaryotes and eukaryotes. Nucleic acids research. 2008;37 suppl_1:D455-D8.
67. Suzek BE, Huang H, McGarvey P, Mazumder R and Wu CH. UniRef: comprehensive and non-redundant UniProt reference clusters. Bioinformatics. 2007;23 10:1282-8.
68. Huson DH, Auch AF, Qi J and Schuster SC. MEGAN analysis of metagenomic data. Genome research. 2007;17 3:377-86.
69. Phua LC, Koh PK, Cheah PY, Ho HK and Chan ECY. Global gas chromatography/time-of-flight mass spectrometry (GC/TOFMS)-based metabonomic profiling of lyophilized human feces. Journal of Chromatography B. 2013;937:103-13.
70. Psychogios N, Hau DD, Peng J, Guo AC, Mandal R, Bouatra S, et al. The human serum metabolome. PloS one. 2011;6 2:e16957.
71. Gao X, Pujos-Guillot E, Martin J-F, Galan P, Juste C, Jia W, et al. Metabolite analysis of human fecal water by gas chromatography/mass spectrometry with ethyl chloroformate derivatization. Analytical biochemistry. 2009;393 2:163-75.
72. Kaufman L and Rousseeuw PJ. Partitioning around medoids (program pam). Finding groups in data: an introduction to cluster analysis. 1990:68-125.

Table 1. Metagenomic datasets used for comparative analysis (Meta-analysis) of the microbiome and MGWAS.

Dataset	No. of samples	Amount of data	No. of genes
**INDIA**	110	110Gb	4.565,784
**USA**	74	441 Gb	5,813,403
**DENMARK**	85	103.87 Gb	5,502,045
**CHINA**	71	180.78Gb	7,198,512

Table 2. OPLS-DA analysis of Metabolomic datasets with fraction of variation explained by X and Y axis with their P-values.

R^2X	R^2Y	Q^2	RMSE	Pre	Ort	pR^2	pQ^2
**0.174**	0.597	0.391	0.322	2	0	0.05	0.05

Figure title and legends

Fig. 1. Comparison of Indian gut microbiome with other major populations using 16S rRNA and metagenomic datasets. (A) Percentage of total reads that could be mapped to IGC and updated IGC containing Indian gene catalogue. Plotted are interquartile ranges (IQR in boxes), median (as dark lines in the boxes), lowest and highest values within 1.5 times the IQR (shown as whiskers extending from boxes) and outliers as points beyond these whiskers. The blue and red boxes showed percentage of reads mapped to IGC and updated IGC (containing the Indian microbial genes). **(B)** Principal Component Analysis using MGS/CAG proportion derived from MWAS. The samples are plotted along with the MGS/CAGs having taxonomic annotations. The MGS/CAGs are coloured according to their phylum. Variations across populations is shown using PC1 and PC2 along with factor loadings of major MGS/CAGs as biplots. **(C)** Illustration of proportions of bacterial families in different populations and their composition as determined from 16S rRNA datasets (adult population only). The mean family compositions of abundant families (≥1%) are represented in separate pie plots from 10 different country-wise datasets, showing their overall microbial composition compared to Indian population.

Fig. 2. Functional variations and differences between Indian populations and other populations determined from core & accessory microbial functions. (A) Procrustes analysis was performed on Bray Curtis distances calculated from core EggNOG and accessory EggNOG abundance tables in all populations. PCA analysis showing the concordance of core and accessory functions in India, Denmark, USA and China populations. The red and black lines are associated with core and accessory datasets, respectively. **(B)** Eigen values and their scores calculated from PCA of samples using core EggNOGs and accessory EggNOGs are plotted. The boxplots showing for core and accessory factor scores for all samples in different populations are shown. Each box

plot represents the median shown as white line between the boxes, the upper and lower ends of the boxes representing upper quartile (75th percentile) and lower quartile (25th percentile). The whiskers extending on both the ends represent 2.5* IQR (Inter Quartile Range). The different coloured dots overlaid for each sample are plotted over the box. **(C)** The enrichment or depletion of functions in India compared to other populations are shown as volcano plots. The log-transformed FDR adj P-values calculated from Wilcoxon rank sum test are plotted on the x-axis. The log odds ratio calculated for India vs Other datasets are plotted on the y-axis. The EggNOGs with P-value<0.05 are shown in blue while those were having P-values>0.05 are shown in red. The EggNOGs extending on right and left side and with P-value>0.05 are labelled as highly enriched in India and other datasets, respectively.

Fig. 3. Variations in gut microbiome at the two locations. (A) PCA analysis of unweighted UniFrac distances of OTUs from Indian population and their differentiation due to locations and diet. Here, the samples are grouped based on their locations (LOC1 and LOC2). The top six principal components tested for correlations with known factors showed location and diet to represent the most significant correlations. **(B)** The within-sample Shannon diversity calculated for LOC1 and LOC2 are plotted as box plots showing the difference in within-samples diversity between the two locations (*: P<0.05). **(C)** Inter sample Bray Curtis distances calculated for samples in LOC1 and LOC2 are shown as boxplots (*: P<0.05). **(D)** Heatmap showing the abundance of OTUs as z-transformed scores. The x-axis represents the OTUs and the genera assigned to the three prominent OTU clusters. **(E)** Significantly different genera between the two locations are shown as boxplots with boxes representing *interquartile range* (IQR), dark lines between the boxes representing median values and whiskers representing the 1.5 x IQR on each side.

Fig. 4. Between class analysis to identify metabotypes and their associated metabolites. (A) Metabolite clusters (MES) abundance profiles of samples were generated and their clustering was performed using PAM (partition around medoids) clustering. The between class and PCA of JSD distances and PAM clustering identified 3 metabotypes to be optimum for their segregation using **(B)** Silhouette index. The metabolites valeric acids, and saturated fatty acids such as palmitic acid and stearic acid, were found higher in Metabotype1. The carbohydrates such as glucose ad galactose, were found higher in Metabotype2. The branched chain amino acids, lauric acid and butyric acid were found higher in Metabotype3. **(C)** OPLS-DA analysis using locations as classes shows locations as differentiating factors in separating the samples based on their metabolomic profiles.

Fig. 5. Spearman's Rank correlations of metabolites with species and metabolic modules. (A) Spearman's Rank Correlation coefficients were calculated between significantly different metagenomic species and significantly different metabolites between LOC1 and LOC2 populations. The correlations showing significant FDR Adj. P <0.05 are plotted. The bars on the right show the Log Odds Ratio of the abundance of MGS with positive values indicating enrichment in LOC1, and the negative values indicating enrichment in LOC2. **(B)** Spearman's Rank correlations between significantly different (FDR Adj. P<0.05, Wilcoxon test) pathway modules and significantly different metabolite abundances in all samples. The significant (P<0.05) correlations are plotted and the colour intensities depict the correlation coefficients. The correlation of metabolites with locations is shown with labels in dark red colours showing association with LOC2, and the labels in green colours showing correlation with LOC1.

Fig. 6. BCAA abundance and their differential correlation with LOC1 and LOC2. (A) Bar plot showing z-normalized values of serum BCAA levels in LOC1 and LOC2. Differential

correlations between KO modules in **(B)** LOC1 and **(C)** LOC2 which showed significant differences (FDR Adj. P-value <0.05) in Spearman's correlations are plotted. The KOs within each module are associated with KOs from other modules. The KOs belonging to BCAA metabolism and their correlations with other KOs showed significant (FDR Adj. P-value <0.05) difference between LOC1 and LOC2. The network shows each KO as node and their associations with other KOs as edges. Only significant correlations (Correlation P-value < 0.05) are plotted. The KOs which had positive correlations with other KOs are connected by edges and the network analysis identifies important associations between modules from KO correlations. **(D)** Network analysis of Spearman's correlations between the branched chain amino acids biosynthesis, degradation and transport KEGG modules with MGS abundance in both LOC1 and LOC2 populations. The node size is proportional to the degree of interactions and the links between module and MGS show interactions or significant correlations (FDR Adj. P < 0.05) with negative (in Red) and positive (in Blue) correlation coefficients. **(E)** Plot showing z-normalized abundance of KOs associated with different modules of BCAA biosynthesis and transporters between LOC1 and LOC2.

Fig. 7. BCAA transporters playing a key role in maintaining the levels of BCAAs in feces and serum. The BCAA levels were observed to be significantly high in the serum samples of LOC1 and in the faecal samples of LOC2. The higher abundance of BCAA biosynthesis genes and the lower abundance of BCAA inward transporters in gut bacteria of LOC1 results in a higher availability of BCAAs for absorption in the blood stream through the gut lumen, and thus were observed in high abundance in the serum samples. In contrast, the high abundance of BCAA inward transporters in the gut bacteria of LOC2, results in a lower availability of BCAAs for absorption in the gut lumen, and thus were observed in lower abundance in the serum samples.

Additional Files

Additional File 1: Supplementary data

Additional File 2: Summary of sequencing statistics showing the number of reads per sample for 16S rRNA amplicon dataset

Additional File 3: Summary of sequencing statistics showing the number of reads per sample for Whole Genome Shotgun metagenomic dataset

Additional File 4: Summary of the reads mapped to Integrated Gene Catalogue and Indian catalogue combined with IGC.

Additional File 5: Figures S1 to S9

Additional File 6: Enriched core microbial functions in Indian gut microbiome compared to other populations

Additional File 7: Genus level differences between Enterotype-1 and Enterotype-2 with FDR Adjusted P-values determined by Wilcoxon rank sum test

Additional File 8: Tables showing Calinski Harabasz index and prediction strength determined for each cluster

Additional File 9: Enriched KOs identified using Wilcoxon rank sum test and Log Odds Ratios between ET-1 and ET-2

Additional File 10: Table showing Polyserial correlation of covariates with the principal components with FDR Adj. P-values

Additional File 11: Table showing enrichment of MGS/CAGs obtained from MWAS with their taxonomic annotations in LOC-1 and LOC-2

Additional File 12: Polyserial correlation of covariates with principal components explaining variations across samples using metabolomic dataset.

Additional File 13: Table showing Spearman's rank correlation coefficient values of metabolites with Metabotypes

Additional File 14: Table showing differential abundance of KEGG Modules between LOC-1 and LOC-2

Additional File 15: List of reference Genomes from NCBI and HMP databases for reference mapping

Figure 1

Click here to download Figure FIGURE_1.tif

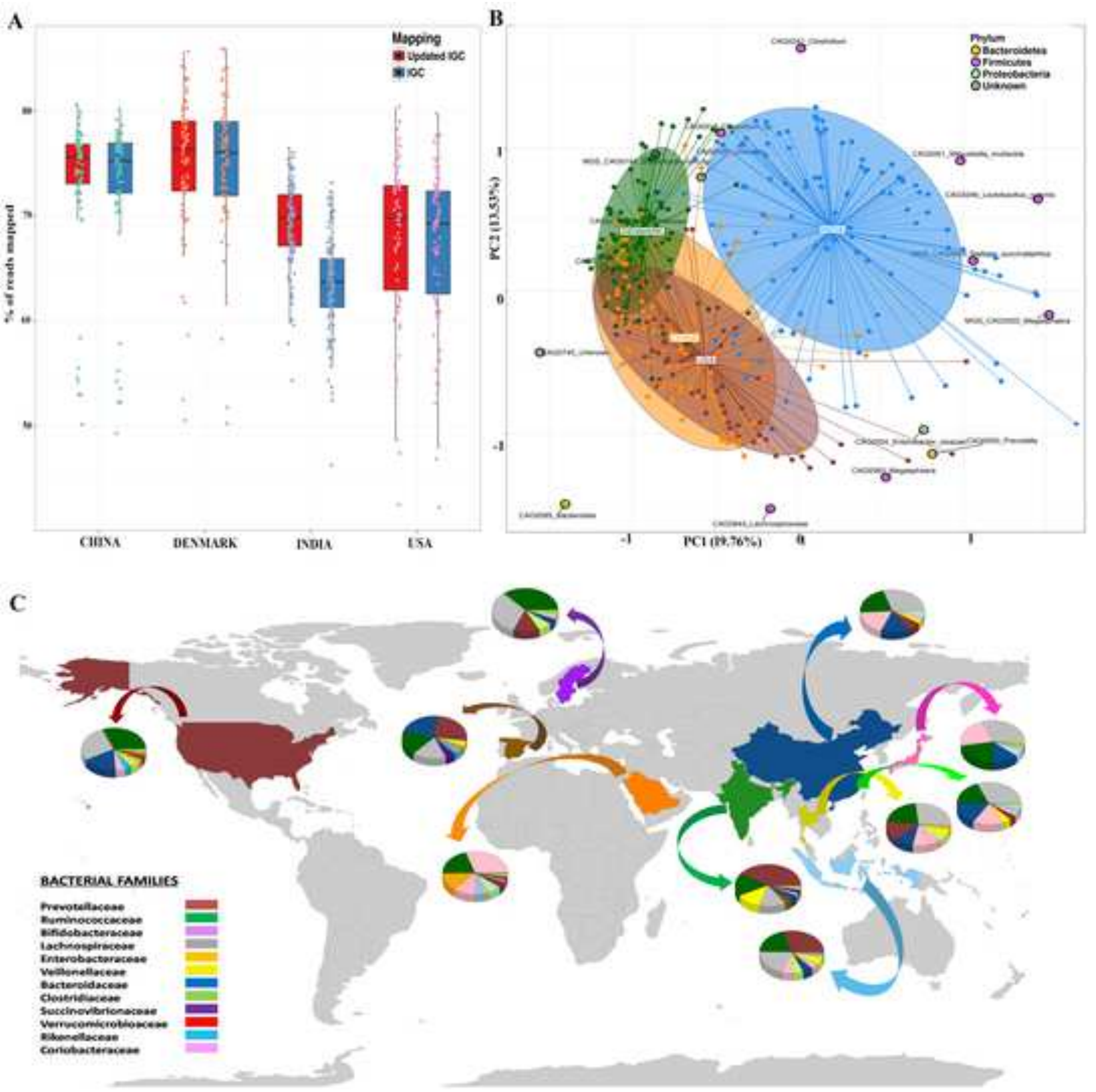

Figure 2

Click here to download Figure FIGURE_2.tif

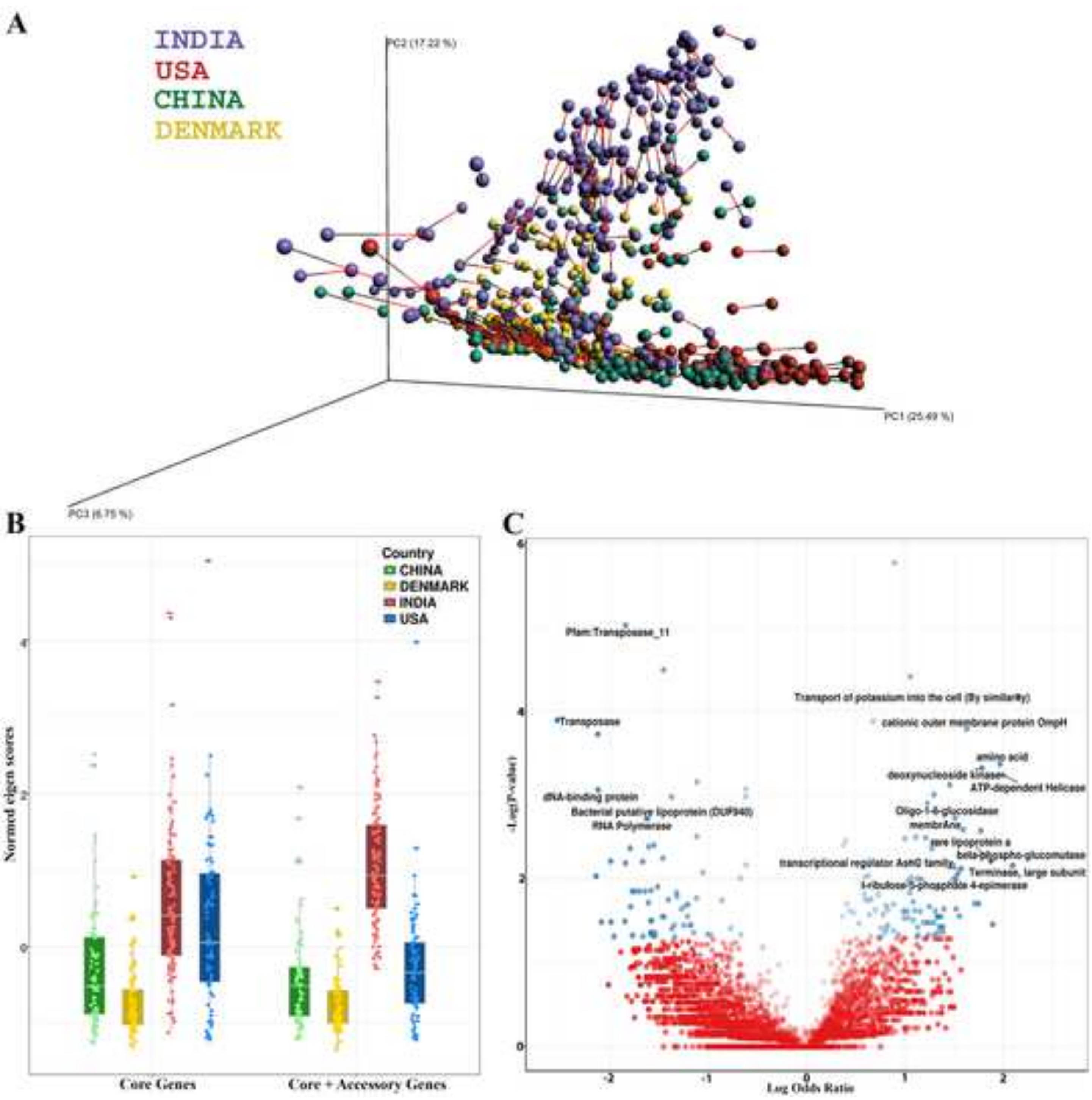

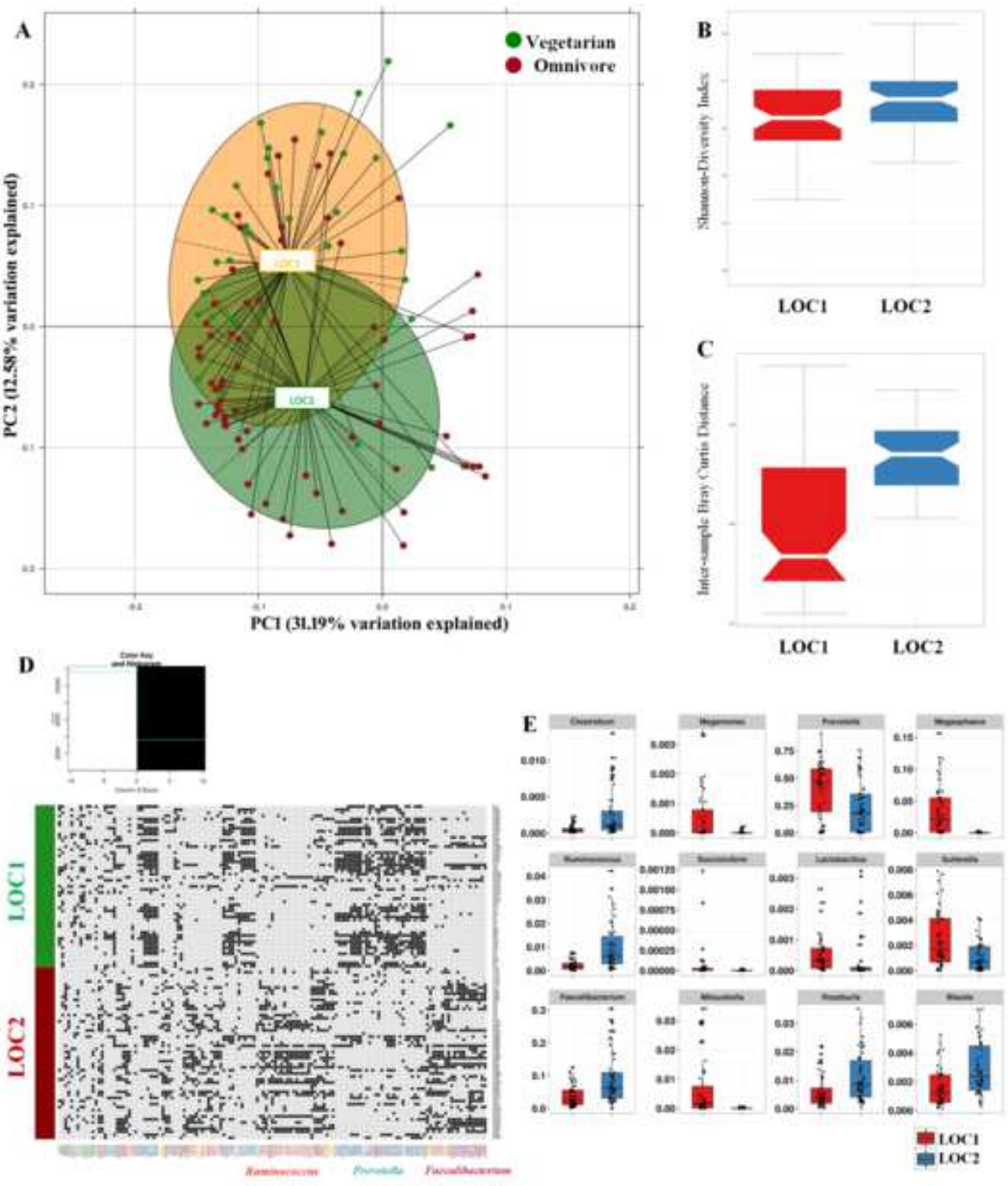
A
Vegetarian
Omnivore
PC2 (12.58% variation explained)
PC1 (31.19% variation explained)
B
Shannon-Diversity Index
LOC1
LOC2
C
Inter-sample Bray Curtis Distance
LOC1
LOC2
D
LOC1
LOC2
E
LOC1
LOC2

Figure 4

Click here to download Figure FIGURE_4.TIF

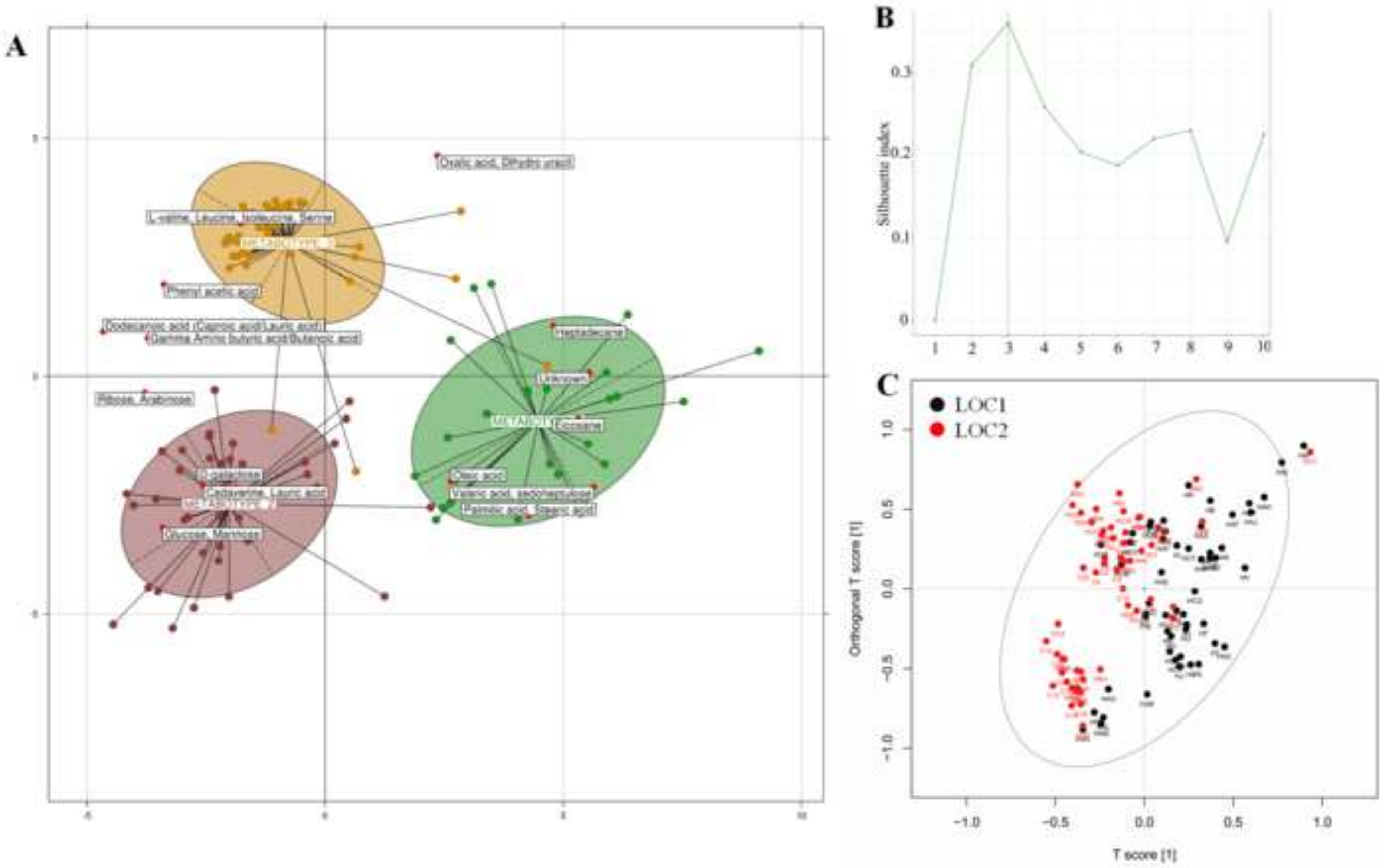

Figure 5

Click here to download Figure FIGURE_5.tif

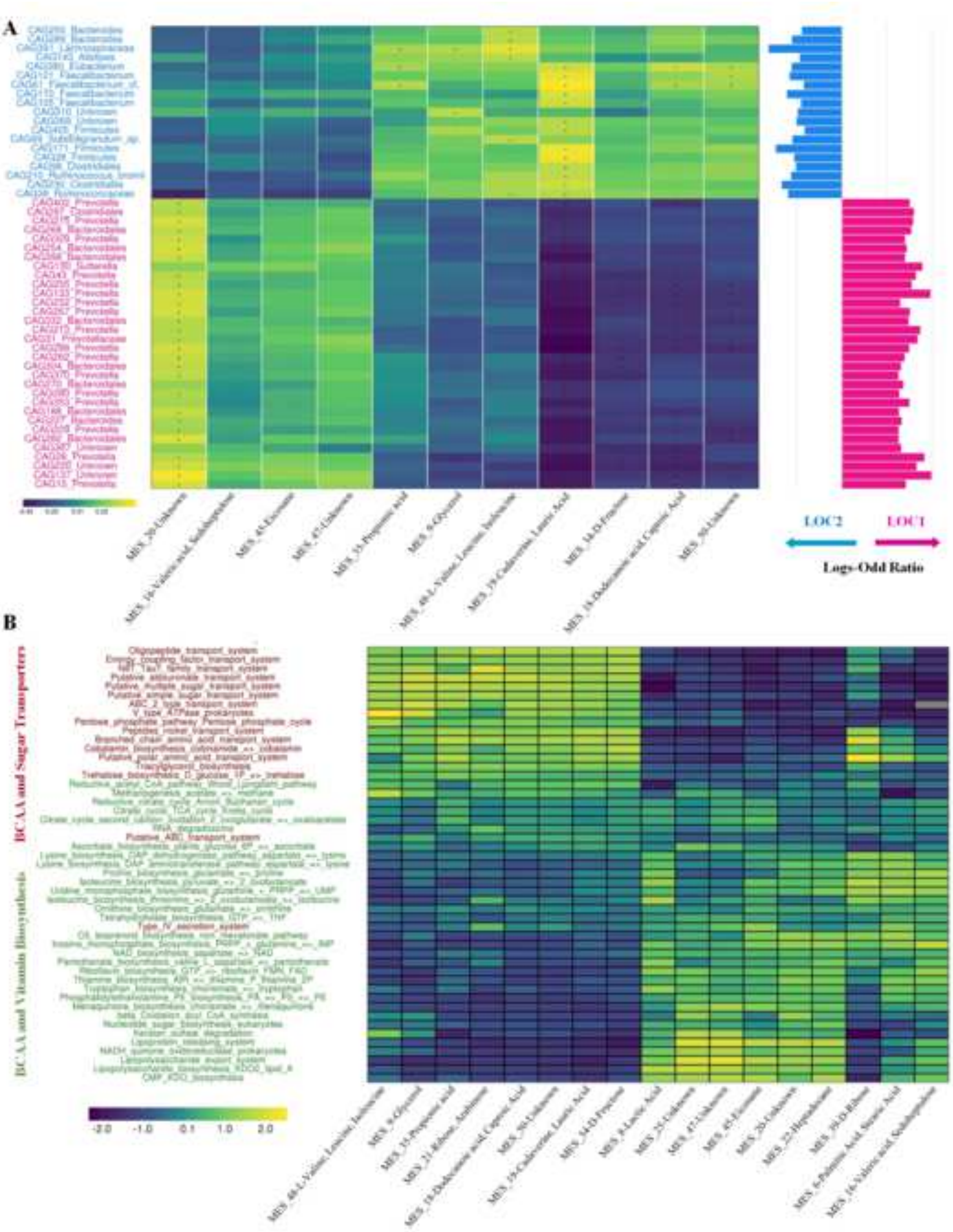

Figure 6

Click here to download Figure FIGURE_6.tif

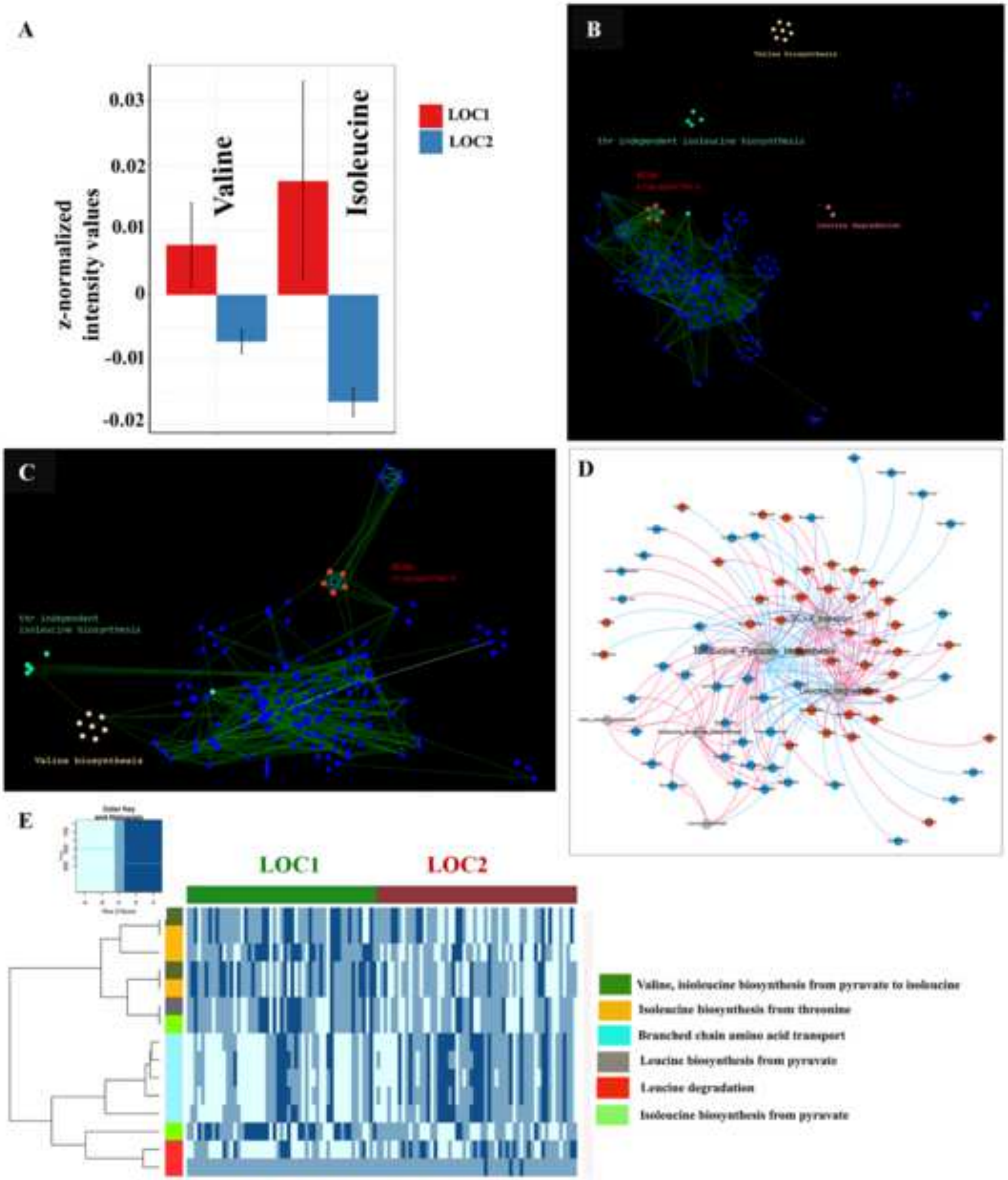

Figure 7 Click here to download Figure FIGURE_7.TIF

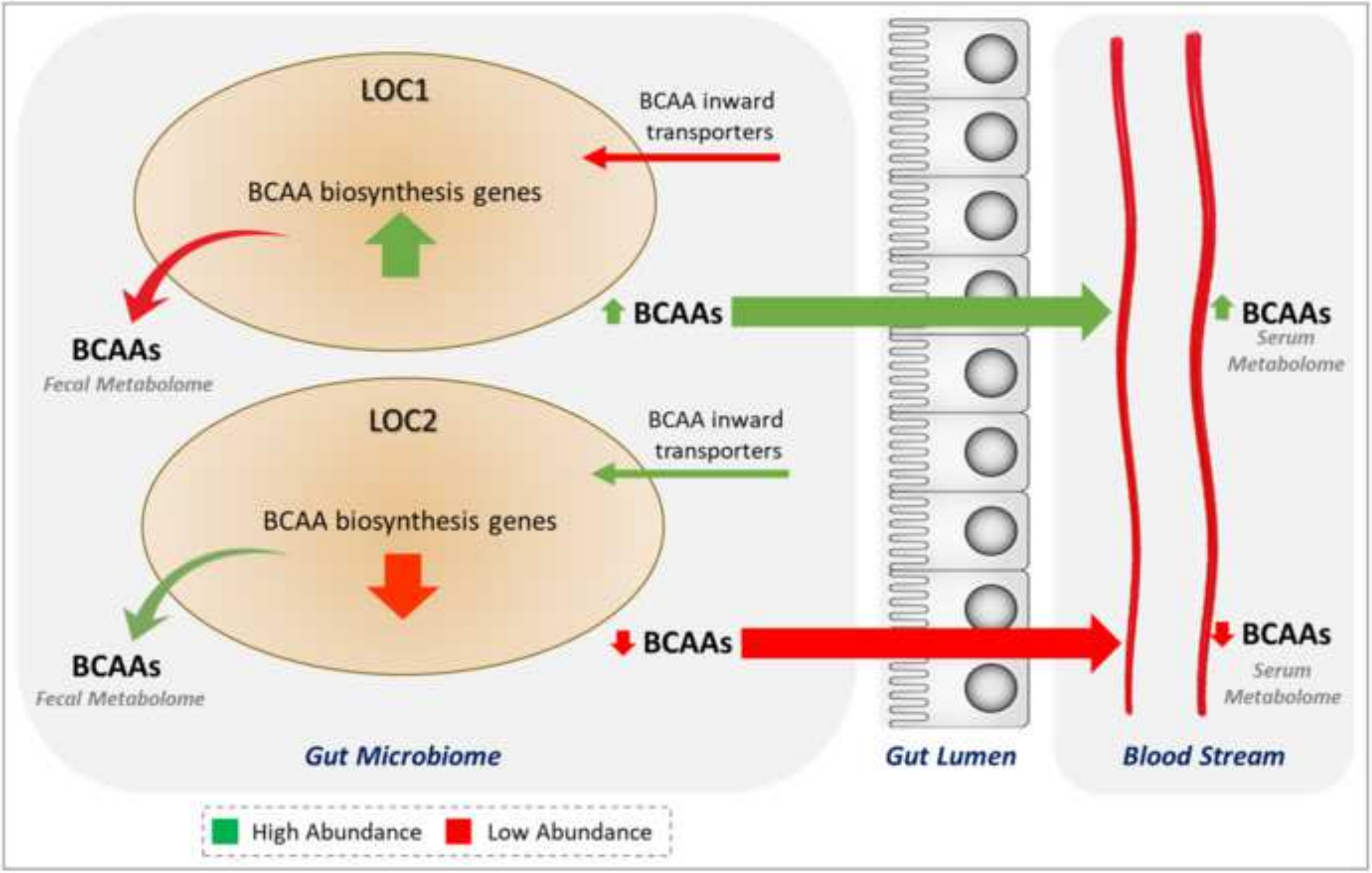

Additional File 1

Click here to access/download
Supplementary Material
Additional_file_1.doc

Additional File 2

Click here to access/download
Supplementary Material
Additional_file_2.xlsx

Additional File 3

Click here to access/download
Supplementary Material
Additional_file_3.xlsx

Additional File 4

Click here to access/download
Supplementary Material
Additional_file4.xlsx

Additional File 5

Click here to access/download
Supplementary Material
Additional_file5.docx

Additional File 6

Click here to access/download
Supplementary Material
Additional_file6.xlsx

Additional File 7

Click here to access/download
Supplementary Material
Additional_file7.xlsx

Additional File 8

Click here to access/download
Supplementary Material
Additional_file8.xlsx

Additional File 9

Click here to access/download
Supplementary Material
Additional_file9.xlsx

Click here to access/download
Supplementary Material
Additional_file10.xlsx

Additional File 11

Click here to access/download
Supplementary Material
Additional_file11.xlsx

Additional File 12

Click here to access/download
Supplementary Material
Additional_file12.xlsx

Additional File 13

Click here to access/download
Supplementary Material
Additional_file13.xlsx

Additional File 14

Click here to access/download
Supplementary Material
Additional_file14.xlsx

Additional File 15

Click here to access/download
Supplementary Material
Additional_file15.xlsx
