# Supplementary material for: The unique composition of Indian gut microbiome, gene catalogue, and associated fecal metabolome deciphered using multi-omics approaches
Source: Gigascience. 2019 Jan 30;8(3):giz004. doi: 10.1093/gigascience/giz004 (PMC6394208; doi:10.1093/gigascience/giz004)
Supplement: giga-d-18-00212_revision_1.pdf [file giz004_giga-d-18-00212_revision_1.pdf]

GigaScience

The unique composition of Indian gut microbiome, gene catalogue and associated faecal metabolome deciphered using multi-omics approaches

--Manuscript Draft--

**Manuscript Number:**	GIGA-D-18-00212R1
**Full Title:**	The unique composition of Indian gut microbiome, gene catalogue and associated faecal metabolome deciphered using multi-omics approaches
**Article Type:**	Research
**Funding Information:**	
**Abstract:**	Background<br><br>Metagenomic studies carried out in the past decade have led to an enhanced understanding of the gut microbiome in human health, however, the Indian gut microbiome is still not well explored. We analysed the gut microbiome of 110 healthy individuals from two distinct locations (North-Central and South) in India using multi-omics approaches, including 16S rRNA gene amplicon sequencing, whole genome shotgun metagenomic sequencing, and metabolomic profiling of faecal and serum samples.<br><br>Results<br><br>The gene catalogue established in this study emphasizes the uniqueness of the Indian gut microbiome in comparison to other populations. The gut microbiome of the cohort from North Central India, which was primarily consuming a plant-based diet, was found to be associated with Prevotella, and also showed an enrichment of Branched Chain Amino Acid (BCAA) and lipopolysaccharide (LPS) biosynthesis pathways. In contrast, the gut microbiome of the cohort from Southern India, which was consuming an omnivorous diet, showed associations with Bacteroides, Ruminococcus and Faecalibacterium, and had an enrichment of Short Chain Fatty Acid (SCFA) biosynthesis pathway and BCAA transporters. This corroborated well with the metabolomics results, which showed higher concentration of BCAAs in the serum metabolome of the North-Central cohort and an association with Prevotella. In contrast, the concentration of BCAAs were found higher in the faecal metabolome of the South Indian cohort, and showed a positive correlation with higher abundance of BCAA transporters.<br><br>Conclusions<br><br>The study revealed the unique composition of Indian gut microbiome, established the Indian gut microbial gene catalogue, and also compared it with the gut microbiomes from other populations. The functional associations revealed using metagenomic and metabolomic approaches provide novel insights on the gut-microbe-metabolic axis, which will be useful for future epidemiological and translational researches
**Corresponding Author:**	Vineet Kumar Sharma, Ph.D.<br>Indian Institute of Science Education and Research Bhopal<br>Bhopal, Madhya Pradesh INDIA
**Corresponding Author Secondary Information:**	
**Corresponding Author's Institution:**	Indian Institute of Science Education and Research Bhopal
**Corresponding Author's Secondary Institution:**	
**First Author:**	Darshan B Dhakan
**First Author Secondary Information:**	
**Order of Authors:**	Darshan B Dhakan

Powered by Editorial Manager® and ProduXion Manager® from Aries Systems Corporation

	Abhijit Maji
	Ashok K Sharma
	Rituja Saxena
	Joby Pulikkan
	Tony Grace
	Andres Gomez
	Joy Scaria
	Katherine R Amato
	Vineet Kumar Sharma, Ph.D.
**Order of Authors Secondary Information:**	
**Response to Reviewers:**	Replies to Comments -Reviewer 1<br><br>The revised manuscript text has been marked in Pink and Orange colours to indicate the changes made as per the suggestions of reviewer 1 and 2, respectively.<br><br>--Reviewer #1: The study entitled "Multi-omics analysis reveals Indian gut microbiome variations due to diet and location and its implications on human health" describes an in-depth sequencing and metabolomic analysis of a unique set of samples from two distinct locations in India. The authors correlate bacterial species composition and fecal metabolites in order to draw conclusions about health in the two geographic locations and the link with diet and disease risk. Specifically, the North Central, primarily vegetarian population, consumes a high proportion of high-fat and sugary foods and ranks among the lowest for life-expectancy. This is compared to a Southern location with an omnivorous population with a much higher life expectancy and lower risks of T2D and cardiovascular disease.<br>The correlation and discussion of specific metabolites and risk factors in the North Indian population versus the Southern population, and the conclusions appears to be supported by the data. The authors concentrate on a limited number of major metabolites, BCAAs and SCFAs, and link these to pathways identified in the bacterial species that are present in the populations. This focused approach is quite effective and the subsequent detailed discussion of P. Copri is very relevant (previous association with rheumatoid arthritis). The importance of bacteria-driven metabolism and its association with vegetarian diets are all interesting points where this study of the Indian population brings news perspectives.<br>Indeed the uniqueness of the Indian population, an under-sampled population, is a major contribution to the available databases. It is for this reason that I consider the work appropriate for publication with a certain number of minor revisions prior to publication:<br><br>Reply: We thank the reviewer for appreciating our work and providing suggestions which really helped in improving the manuscript. We have tried our best to satisfactorily address the comments and have performed all the suggested analysis. Additionally, we have improved the metagenomic assembly of Indian gut microbiome using IDBA-UD assembler (Kuang et al.; GigaScience; 2017: Please see Methods section). The mean N50 values across all samples showed an increase from 946 bp to 2,288 bp, and the total contig size increased from 1.78 Gbp to 3.086 Gbp (Please see Supplementary Figure 1) in the revised assembly. The updated non-redundant gene catalogue for Indian gut microbiome now consists of 1,551,581 genes. The genes from Indian gene catalogue were added to the Integrated Gene Catalogue (IGC) to construct the ‘Updated Integrated Gene Catalogue’ (India+IGC), which now consists of 10,823,291 non-redundant genes. We have updated all the corresponding results as per the revised Updated IGC and the suggestions provided by reviewer.<br>Reference<br>Kuang et al.; Connections between the human gut microbiome and gestational diabetes mellitus; GigaScience; 2017; doi 10.1093/gigascience/gix058

General comments:
--Subjects were excluded if there was reported use of antibiotics during the previous month. How was this cutoff determined and was any analysis performed on the cohort to determine if there was any residual effect of antibiotic use (a known issue in India)? This could be as simple as a PCoA plot, using time since last antibiotics exposure as a variable in the 16s diversity analysis.

Reply: We agree with the Reviewer that antibiotic treatment can have residual effects on the gut microbiome and is an important consideration while collecting the samples. A few recent studies have specifically examined these effects, such as the study carried out by Suez et al. demonstrated that a period of 28 days was sufficient for spontaneous recovery of microbiome composition after antibiotic treatment (Please refer Figure 2 of the article [1]). A recent study by Ruixin Liu et al. [2] has also used the same criteria, where the subjects who did not receive any antibiotic treatment for at least one month prior to sample collection were selected (Please refer to Online Methods: 'Faecal sample collection and DNA extraction' section of the cited manuscript). Dethlefsen and Relman [3] show that microbiome communities return to their initial state within one week after the end of antibiotic course. However, we agree that the return of microbiome composition to initial state do vary depending on the type of antibiotic used and can be incomplete. We also agree with the Reviewer's suggestion that a PCoA using time as variable since last antibiotic exposure and estimating its effect would help to identify the effect of treatment on microbiome composition. However, we did not collect this data during the sample collection, and thus could not perform this analysis. Nevertheless, as per the above mentioned studies including the recent ones, we were very careful in recruiting only those volunteers who were not exposed to any antibiotic treatment for over a month.
References
1.Jotham Suez et al; Post-Antibiotic Gut Mucosal Microbiome Reconstitution is Impaired by Probiotics and Improved by Autologous FMT; Cell; 2018; doi:10.1016/j.cell.2018.08.047
2.Ruixin Liu et al; Gut microbiome and serum metabolome alterations in obesity and after weight-loss intervention; Nature Medicine; 2017; doi:10.1038/nm.4358
3.Les Dethlefsen and David Relman; Incomplete recovery and individualised responses of the human distal gut microbiota to repeated antibiotic perturbation; PNAS; 2011; doi:10.1073/pnas.1000087107

--Could the authors please explain their use of Greengenes 13_5? This release dates to 2013. Was SILVA tested?

Reply: We used the Greengenes database because of its wide use in large number of microbiome studies (Yatsunenko et al; Nature; 2011 & Nakayama et al; Sci Rep; 2016) and also in some of our early publications (Maji et al; Environ Microbiol; 2018, Pullikan J et al; Microb Ecol; 2018). We agree with the Reviewer's suggestion of using ARB SILVA database for taxonomic classification of 16S rRNA gene sequences since the Greengenes database has not been updated after May 2013, which justifies the use of more recently updated SILVA database.
As per Reviewer's suggestion, we have now repeated the 16S rRNA gene analysis using ARB SILVA database release 132 (13th December 2017) as reference database for taxonomic annotation. In order to visualize the differences in the results generated from analysis using the two databases, we compared the taxonomies and OTUs generated from the two databases. The Supplementary Table 1 provides details on the percentage of reads assigned at different hierarchical levels using Greengenes and ARB Silva database as reference. There was a marked increase in assignment of OTUs at genus level using ARB SILVA database (95.2%) compared to Greengenes database (54.56%). The increase in the taxonomic annotation was also observed for other population datasets used in the comparison (Supplementary Table 1).
After the reanalysis of 16S rRNA gene data using the annotations from ARB SILVA database, the results have been updated in the revised manuscript in the Results and Figures (please see Figure 1C, Additional File 5: Figure S3, Figure S5 and Figure S10). We observed similar trends with significant improvements in the annotations of OTUs at the genus level.
References
Tanya Yatsunenko et al; Human gut microbiome viewed across age and geography; Nature; 2012; doi:10.1038/nature11053

Jiro Nakayama; Diversity in the gut bacterial community of school-age children in Asia; Nature Scientific Reports; 2015; doi:10.1038/srep08397
Maji A. et al; Gut microbiome contributes to impairment of immunity in pulmonary tuberculosis patients by alteration of butyrate and propionate producers; Environmental Microbiology; 2018; doi:10.1111/1462-2920.14015
Pullikan J. et al; Gut microbial dysbiosis in Indian children with Autism Spectrum Disorders; Microbial Ecology; 2018; doi:10.1007/s00248-018-1176-2

--I am convinced of the utility of the study, despite some of the additional comments below. Therefore, I would request that the raw shotgun metagenomics data also be made available, and not just the assembled contigs as is currently the case. This is extremely important so that future groups can improve on assemblies and annotations as more data is generated from future studies.

Reply: As per the reviewer's suggestion, we have now released the raw reads data which can be found at NCBI SRA (https://www.ncbi.nlm.nih.gov/sra) with Project ID: PRJNA397112. The assembled contigs, genes and gene catalogue will also be uploaded on the Giga Science ftp server, which can be accessed by any researcher for the future studies.

Specific comments:
--Line 209: "Detection of Enterotypes" The authors use the term 'analysis of enterotypes', referring to Arumugam et al., for the analysis performed in this section and relate the results to those found in the previous study. However the resulting two enterotypes are more accurately, and simply, called clusters, as they are based on two distinct populations in the current study only. This is in contrast to four-country, 22-metagenome analysis performed in Arumugam et al. I would suggest that the terminology be revised. This same type of nomenclature is repeated in line 272: 'metabotype.' I thank that referring to these as clusters is more accurate and more consistent.
It is also present in the discussion (lines 400-401) and methods (699). I would just stress again that two distinct geographical locations which can be statistically separated into two groups, within a single study, does not constitute an enterotype as defined in Arumugam et al. As LOC1 and LOC2 are distinct in this study, factoring this information into clinically relevant models (lines 403-408) does not require a further variable. The analysis and conclusions about the two groups, nevertheless, appear valid.
My suggestion, if the authors wish to use the "enterotype" comparison, would be to explore how this new dataset of 110 individuals fits when combined with that from Arumugam et al. Do the samples still classify into three enterotypes, and what is the distribution across LOC1 and LOC2?

Reply: We agree with the Reviewer's suggestion that the term 'enterotype' should be used when referring to cross national clusters resulting from similarities in microbiome profiles of different populations and their clustering into groups.
We thank the reviewer for the valuable suggestion to compare the Indian samples with that of Arumugam et al., and see if the Indian samples could still be classified into the three enterotypes. Thus, we performed the meta-analysis of 37 samples from the four nations used in Arumugam et al. with our Indian cohort consisting of 110 samples (Please see Figure 3A and Additional File 8). We were able to classify the Indian samples into three enterotypes using genus-level abundance of 110 Indian + 37 samples from four countries (Arumugam et al.). We also identified the distribution of samples from LOC1 and LOC2 in these three enterotypes. We could observe clear differences in representation of samples from India and the other four populations. We could also identify the differences in representation of samples from LOC1 and LOC2 among these enterotypes. We thank the Reviewer for suggesting this analysis, which helped in confirming the previous analysis and results. We have revised the results section 'Line: 246-255' to include the above analysis and have highlighted in pink. We have also revised the terminology from 'enterotypes' to 'clusters' when referring to the clusters using only Indian datasets in all the sections.

--Line 235: 16S Data Analysis
The authors use rarefied reads for downstream analysis. This type of normalization, while useful for calculating UniFrac distances, is no longer accepted as the gold

Powered by Editorial Manager® and ProduXion Manager® from Aries Systems Corporation

standard for statistical analysis of 16s data. See (McMurdie PJ, Holmes S. Waste not, want not: why rarefying microbiome data is inadmissible. PLoS computational biology. 2014). The authors should explain why they decided to use sub-sampling normalization. How the threshold of 100K was determined?

Reply: We thank the reviewer for this important suggestion on normalizing the 16S rRNA gene counts. Regarding the threshold of 100K, it was a cut-off based on the lowest sequencing depth among all the samples. We agree with the reviewer that the rarefactions method is useful for calculating UniFrac distances, however for comparative analysis it is not the gold standard now, and should be replaced with the methods used in study by McMurdie et al; PLOS Computational biology, as highlighted by the Reviewer. We would like to mention that we did not use rarefaction in any of our statistical analysis or comparisons except for diversity estimations (Alpha and Beta Diversity). For statistical analysis, we used relative abundance of taxa. As per the reviewer's suggestions, we have now revised all the statistical analysis performed using DESeq2 package in R as mentioned in the study (McMurdie et al.) suggested by the Reviewer. The Unifrac analysis has been revised based on OTUs picked using SILVA database (Please see Additional File 5: Figure S11 and Additional File 13).

--The differential analysis performed in relation to clinical data and location (lines 247-255) should be reanalyzed using current normalization methods (e.g. DeSeq2 or edgeR packages exist for R).

Reply: We appreciate and agree with the reviewer's suggestions on normalization. Earlier, we had calculated relative abundance by normalizing the raw count of each taxon with total number of reads in each sample. However, as per the reviewer's suggestion we have now re-run all the differential analysis on raw counts at taxonomic level using negative Binomial model based-Wald test in DESeq2. The genera that showed significant difference between Location 1 and Location 2 were plotted (Please see Figure 3B). We also reanalysed the differential species between LOC1 and LOC2 using DESeq2 based normalization on raw abundances of species obtained from mapping of metagenomic reads to the reference genomes (Please see Figure 3C). Further, differential analysis between clusters was also performed using DeSeq2 based normalization on raw counts (Please see Additional File 10). The results and figures have now been updated according to the latest analysis carried out using DESeq2.

--Lines 347-352: The addition of 110 individuals is a major contribution. Yet, I think that the authors would agree, any future metagenomics analysis of the intestinal microbiota, even those focusing on South-Asia populations, would best be accomplished using the IGC + this study's additional database. Analysis would not be performed using this study's catalog alone. Please consider rewording here to accurately present the impact of the study.

Reply: We agree with the reviewer's suggestion that IGC+ Indian gene catalogue (constructed in this study), referred to as 'Updated-IGC', would be more useful as a reference database than the Indian gene catalog alone even when studying the South-Asian populations. Thus, we have now also uploaded the 'Updated IGC' at the GigaScience web server. We have also revised the line 421-424 to include these changes.

--Line 561: The authors appear to perform normalization in relation to gene length, probably RPKM. Like 16s analysis, it has been demonstrated that this type of normalization is not the most appropriate for whole genome metagenomics analysis (https://doi.org/10.1186/s12864-016-2386-y). The authors should rerun the analysis to validate that the bacterial species cited in the manuscript remain significant after applying a modern normalization method such as DESeq2 or edgeR. Perhaps other significant species will also be identified.

Reply: We do agree with the Reviewer that the method of normalization can have an impact on the results. As per the reviewer's suggestion, we have now recalculated gene abundance for all the datasets as raw counts instead of normalizing them by gene length, or as proportions. The raw read counts of genes were used for MGWAS analysis and the construction of MGS was performed. The MGS abundance was

Powered by Editorial Manager® and ProduXion Manager® from Aries Systems Corporation

recalculated, and reanalysed using DESeq2. The P-values obtained were used for further analysis. The differential abundance of MGS between India and other datasets were determined using negative binomial model-based Wald test implemented in DESeq2 for calculating the P-values (Please see Additional File 5: Figure S2, Additional File 6). Moreover, the differential abundance (P-value calculation) of MGS between LOC1 and LOC2 was also determined using DESeq2 based normalization (Please see Additional File 14). Using the raw abundance, we also re-calculated abundance of EggNOG, KEGG Orthologues (KO) and KEGG Modules and performed differential analysis using NB model based Wald test in DESeq2 (Please see Figure 2C, 2D and Additional File 7, Additional File 12, Additional File 17). We have now revised the manuscript at the above mentioned places to include the revised results.

--Line 603: The reference cited does not describe the canopy-mgs algorithm. The correct reference is Nature Biotechnology volume 32, pages 822-828 (2014); 'Identification and assembly of genomes and genetic elements in complex metagenomic samples without using reference genomes.' This reference also describes MGS (metagenomic species) that the authors refer to (Line 726, and elsewhere in text).

Reply: We thank the Reviewer for pointing out this error. We have now corrected this reference in the manuscript (Line: 650).

Reply to Comments- Reviewer 2

The revised manuscript text has been marked in Pink and Orange colours to indicate the changes made as per the suggestions of reviewer 1 and 2, respectively.

Reviewer #2: # SUMMARY
--In this manuscript Dhakan & Maji et al. report on their multi-omic analyses of 110 healthy individuals from two distinct regions in India. The authors obtained 16S rRNA gene (V3 region) amplicon sequencing data, metagenomic sequencing data, and metabolomic data from volunteers' faecal samples. In addition, metabolomic data from serum samples were obtained. Using the metagenomic sequencing data, the existing Integrated Gene Catalog (IGC) was expanded by adding novel, non-redundant genes derived from the India cohort. This represents an important addition to the IGC, thereby further complementing the global, human gut-derived microbial gene catalog. The authors compared the taxonomic composition (amplicon and metagenomic data) and the functional potential (metagenomic data) of Indian-derived gut samples to samples from earlier studies (China, Denmark, USA) and found the Indian microbiome to be largely distinct. The authors conclude that diet is likely to be a strong factor in this, especially since the eating habits are often strongly conserved according to region. Using the metabolomic data, Dhakan & Maji et al. identified differences in the faecal and serum concentrations according to region.
GENERAL COMMENTS
--Overall, I think that this study nicely complements existing microbiome studies by further expanding gut microbiome characterization to include samples derived from an Indian population and from different diets (plant-based and omnivorous). Moreover, it highlights the importance of complementary omics, here, metabolomics, in the study of host-microbe interactions.

Reply: We thank the reviewer for appreciating our work and providing suggestions which really helped in improving the manuscript. We have tried our best to satisfactorily address the comments and have performed all the suggested analysis. Additionally, we have improved the metagenomic assembly of Indian gut microbiome using IDBA-UD assembler (Kuang et al.; GigaScience; 2017: Please see Methods section). The mean N50 values across all samples showed an increase from 946 bp to 2,288 bp, and the total contig size increased from 1.78 Gbp to 3.086 Gbp (Please see Supplementary Figure 1) in the revised assembly. The updated non-redundant gene catalogue for Indian gut microbiome now consists of 1,551,581 genes. The genes from Indian gene catalogue were added to the Integrated Gene Catalogue (IGC), to construct the ‘Updated Integrated Gene Catalogue’ (India+IGC) and now consists of 10,823,291 non-redundant genes. We have updated all the corresponding results as

Powered by Editorial Manager® and ProduXion Manager® from Aries Systems Corporation

per the revised Updated IGC and the suggestions provided by reviewer.
Reference
Kuang et al.; Connections between the human gut microbiome and gestational diabetes mellitus; GigaScience; 2017; doi 10.1093/gigascience/gix058

--While many of the authors' conclusions are supported by the reported results, I found that some conclusions need to be toned down as there is not sufficient supporting evidence for these conclusions. Please also see my detailed comments.

Reply: We have made our best efforts to address all the comments and have provided below a point-wise reply to the comments and suggestions. We have also revised the Discussion section at several places to tone down the conclusions correlating the impact of microbiome composition on health as suggested by the reviewer.

--The metagenomic sequencing depth in this study is unfortunately not particularly deep, but neither is it shallow. While sequencing depth is always a limiting factor, it is an important factor if the objective is the recovery of novel genetic/genomic information. This needs to be considered when concluding.

Reply: We agree with the reviewer that sequencing depth is a limiting factor in metagenomic studies. In this study, the sequencing depth was not too high (1.5 ± 0.5 Gbp per sample, mean ± standard deviation), compared to the datasets from other microbiome studies (METAHIT: 4.5 Gbp, 100bp reads; Human Microbiome Project: 2.9 Gb, 100bp reads; Qin et al; 2012: 2.61Gbp, 100 bp reads) that were used for comparison with Indian microbiome. However, through a read length of 150bp and a decent paired-end sequencing depth (1.5Gbp) of 110 individuals in this study, we have been able to provide the first insights on the Indian gut microbiome and reveal its unique composition. The increase in sequencing depth certainly would recover more novel genetic information from low abundant microbes which is an important point to consider while making the conclusions. We have now mentioned it in the discussion section and have also considered it while interpreting the results and deriving conclusions (Line: 408-411, 518-520).
References
Qin et a; A human gut microbial gene catalogue established by metagenomic sequencing; Nature; 2010; doi 10.1038/nature08821.
The Human Microbiome Project Consortium; Structure, function and diversity of the healthy human microbiome; Nature; 2012; doi 10.1038/nature11234.
Qin et al; A metagenome-wide association study of gut microbiota in type-2 diabetes; Nature; 2012; doi 10.1038/nature11450.

--Moreover, I found the variation/spread of the samples from the Indian cohort exceptionally large (Fig. 1 B). This might be something the authors could elaborate on.

Reply: We agree with the reviewer that the spread of the samples from the Indian cohort needs to be discussed in the manuscript. The reason for this variation/spread is the higher inter-sample distances between samples from Indian population compared to other populations (Additional File 5: Figure S1). We have now analysed the principal coordinates from PCA in Figure 1B (Please see Additional File 5; Figure S2). The Wilcoxon rank sum test of coordinates at PC1 revealed significant difference between LOC1 and LOC2 coordinates. A plausible reason could to be the dietary differences between LOC2 population (non-vegetarian diet) and LOC1 population (plant-based diet), resulting into significant (FDR Adj. P-value = 0.0013) differences observed in their MGS abundance profiles (Additional File 5: Figure S2). We have now included this analysis and elaborated it in the results (Line: 182-188).

--An experiment which I would have liked to see - I am not saying that it is necessary, though - is an ordination of the 110 samples alone, i.e., not contrasting against samples from other studies but rather within the current study. I would be curious to know if there is substantial separation of samples according to region and/or diet.

Reply: We thank the reviewer for this suggestion and have now performed an ordination of samples based on gene relative abundance table of 110 Indian samples only and observed their separation according to region and diet (Please see Additional File 5: Figure S13). We have also performed polyserial correlation to observe the effect

of diet and location on separation of samples using gene abundance (Please see Additional File 13). The location and diet both were observed to be significantly associated (FDR Adj. P< 0.01) with PC1 explaining the maximum variation in the unsupervised clustering of Indian samples (Line: 288-292).

--Finally, I would strongly encourage the authors to be more careful with their conclusions on "the gut microbiome and its functional consequences on human health". The present study did not investigate "non-healthy" individuals from the respective regions. It might very well be that the same or very similar observations would have been made with respect to faecal/serum metabolite levels and correlations to respective microorganisms if "non-healthy" individuals were included

Reply: As suggested by the reviewer, we have revised the discussion and conclusion sections, and have carefully rewritten the interpretations and conclusions related to human health. We have also revised the title of the manuscript as suggested in the later comments.

--The Data Description section should be extended. It should include description of the metabolomic data that was generated as well as of the metadata which was collected (Age, BMI, etc.). Some of this information is provided in the Methods "Study design and subject enrolment" and should be moved to the Data Description instead.

Reply: As per the suggestion, we have now included the description of the metabolomic data, BMI, age, metadata, study design and subject enrolment in the Data Description section (Line: 109-132). Moreover we have now provided a separate table for data collected for different samples in Additional File 1.

--Instead of reporting "thresholded" p-values (e.g., "P<0.05)"), please report the actual p-values.

Reply: We have replaced the threshold P-values with the actual P-values at most places in the manuscript. However at places such as Line: 317, where multiple species/genes are mentioned we have reported a threshold P-value for considering significant ones.

--I would encourage the authors to include the version and parameters of tools that were used in the Methods.

Reply: We have now included the version and parameters of the tools that were used in the Methods section (Please see Methods section).

--Moreover, it appears that references are occasionally missing, e.g., for the WMW test, FDR-adjustment, Polyserial correlation/biserial correlations, Reporter features algorithm, etc.

Reply: Thanks for pointing it out. We have now added the references for the statistical tests used for the analysis.

--The readability of the manuscript should be further improved, e.g., by involving a professional editing service.

Reply: We have carefully read the manuscript and have made specific efforts to improve the readability. I hope you would find the revised manuscript much improved than the previous version.

My comments below refer to the second row of line numbers, i.e., the one _not_ in typewriter font.
TITLE
--Title: "its implications on human health": It is not clear what the "its" refers to. I would suggest adjusting the title accordingly. Moreover, while it has been shown that diet has an effect on the gut microbiome, I do not know whether "due" is the right wording here. I prefer how the authors phrased it in the abstract, e.g., "showed associations with". I

would thus recommend a more careful wording. Moreover, no "non-healthy" individuals were included in the present study, hence making the conclusion of "implications" rather difficult due to lack of supporting evidence (s.a., my general comments)

Reply: We thank the Reviewer for this suggestion. We have revised the title to provide more emphasis on the unique composition of Indian gut microbiome and the functional associations revealed through metabolomics approach. The revised title now reads as “The unique composition of Indian gut microbiome, gene catalogue and associated faecal metabolome deciphered using multi-omics approaches”. I hope the reviewers would find it more appropriate than the earlier title.

ABSTRACT

--L25: "comprehensively": This could be debated, e.g., at what sequencing depth would one consider to have covered the composition and/or function "comprehensively". Please remove this.

Reply: We have removed the word ‘comprehensive’ from this line. (Line: 25).

--L26: "including 16S rRNA marker gene and shotgun metagenomics": This sounds to me as if the "16S rRNA marker gene" sequencing is also considered "metagenomics", which it is not. I would thus suggest "including 16S rRNA gene amplicon sequencing, metagenomic sequencing, and ...".

Reply: We agree with the reviewer and understand that 16S rRNA marker gene sequencing is not metagenomics. While framing the sentence it appeared as one of the methods for metagenomics, and we thank the reviewer for pointing it out. We have now revised it in the manuscript (Line: 26-27).

--L32: "BCAA": This abbreviation was not introduced before. Same applies to "SCFA in L34". Please adjust accordingly throughout and for all other abbreviations in the manuscript.

Reply: We have now provided the expanded form of all abbreviations at the first instance of their inclusion in the manuscript and have made these changes at all required places (Line: 33, 36, 37).

--L37: "BCAAs were found higher": "higher" in what? I assume in concentration, but this should be clarified in the text.

Reply: Indeed, we were referring to the BCAA concentration, and we have now revised this sentence (Line: 38-40).

--L41: "its functional consequences on human health": I think that this is too strong of a claim here. In particular, this study involved only healthy individuals, hence, while there have been differences observed, these differences may not necessarily have a positive or negative effect, but could be neutral. Put differently, different gut microbiomes may be related to healthy individuals or "non-healthy" individuals might have revealed similar findings.

Reply: We agree with the Reviewer and have revised the sentence (Line: 43-44).

MAIN TEXT

--L63: "constitution": This typically refers to the "the highest laws of a sovereign state, a federated state, a country or other polity." (https://en.wikipedia.org/wiki/Constitution_(disambiguation)). The authors should consider reformulating this, e.g., by using "condition" or a more appropriate term. Maybe the authors were referring to "composition"? It is not really clear to me, especially with respect to "understanding its variability". It is not just the taxonomic but also the functional composition which has been shown to be of importance. Hence, I would encourage the authors to clarify their point more explicitly here. Finally, this sentence may be misleading as "dysbiosis" is typically used when comparing (at least) one phenotype (e.g., lean) to another (e.g., obese). However, this study is focussed only on one phenotype, i.e., "healthy".

Reply: We agree that the word ‘constitution’ can be replaced with ‘composition’ and have revised this sentence by including all the suggestions made by the reviewer (Lines: 54-55).

--L69: "WGS": This abbreviation was not properly introduced. Please make sure to do so for all abbreviations throughout the manuscript.

Reply: Thank you for this comment. We have now introduced this abbreviation and all other abbreviations in the manuscript at their first usage (Line: 59-60).

--L72: "Branch" -> "Branched".

Reply: We have corrected this word (Line: 62-63).

--L83: I would rephrase "from the major world populations".

Reply: We have rephrased this statement (Line: 74).

--L86: I would rephrase "equally dominated". Typically, "domination" is used when a single entity has a majority stake.

Reply: We have rephrased this word as ‘equal representation’ (Line: 77-78).

--L114: I am not sure if these two locations as well as the total cohort size (n = 110) qualify as being "representative". I would thus suggest to remove the respective wording. Same applies to "comprehensive" , s.a., my respective comment above.

Reply: We agree with the suggestion and have removed the word ‘representative’ and reframed the sentence. (Line: 104-105).

--L115: "16S rRNA sequencing" -> "16S rRNA gene sequencing".

Reply: We have made this change (Line: 105-106).

--L133ff: Was the assembly done on reads from individual samples or on the pooled set of reads? It is not clear as the authors emphasize pooling in the subsequent sentence which reads to me as if this was _not_ done to generate the 1,337,547 contigs. Please clarify.

Reply: We wish to clarify that the assembly was performed on individual samples separately. The reads were mapped back to the assembled contigs from individual samples and the reads that did not map to the contigs from each sample were pooled from all the samples and a denovo cross assembly was performed using the unmapped reads from all the samples. We have employed a similar strategy for contigs and gene catalogue construction as used in other studies [1]. We have now clearly clarified this point in the revised manuscript (Line: 139-144, 590-592).
References:
Qin et al; A human gut microbial gene catalogue established by metagenomic sequencing; Nature 2011 (see section Metagenomic sequencing of gut microbiomes).

--L139: Please remove "In addition". It sounds as if this is a result from the current paper but it is not.

Reply: We have removed this word and have reframed the sentence. (Line: 146).

--L141: "populations" seems inappropriate here as the HMP and MetaHIT projects both involved multiple populations themselves.

Reply: We agree with the reviewer and have now changed this word to “multiple populations”. (Line: 147- 148)

--L145 + L146: Please specify what the numbers in the brackets with the "plus-minus" mean. Are they representing the standard deviation?

Reply: As correctly pointed out by the reviewer, the ‘plus-minus’ represent standard deviation. We have now added standard deviation in the brackets, for example 69.2% (± 4.01% standard deviation). (Line: 153,155).

--L147f: I am not sure what the authors wanted to say here. Do they mean that reads from _other_ studies were mapped to the original IGC as well as to the updated IGC?

Reply: Here, we had mapped reads from microbiome samples of healthy individuals from three different studies (USA datasets from HMP, Denmark dataset from MetaHIT and Chinese datasets from Qin et al; 2012) on the original IGC and on the updated IGC. We have reframed this statement (Line: 158-162) and the mapping is shown in Fig. 1A. The results have been updated as per the revised gene catalogue.

--L150f: Please rephrase this to reflect that only a _subset_ of the genes of the 110 Indian gut samples in the current study are not represented in other gut microbiome datasets. After all, 718,360 of the 1,479,998 non-redundant genes were added to the original IGC but not the full extent of the current non-redundant genes.

Reply: We thank the Reviewer for this comment. We would like to mention that we aligned the set of non-redundant genes (after removal of redundancy) identified in Indian gut microbiome with the Integrated Gene Catalogue (IGC), and removed the genes sharing ≥90% identity with IGC genes. Thus, the remaining genes from Indian gut microbial gene catalogue which were unique to the IGC (sharing < 90% identity) were added to generate the updated IGC. As per the revised gene catalogue, 943,395 genes from Indian microbiome samples were added to IGC, thus forming an updated IGC containing only the non-redundant genes from Indian cohort. We have now reframed the sentence (Line: 148-153, 163-164).

--L157: "non-reference" -> "reference-independent".

Reply: We have replaced ‘non-reference’ with ‘reference-independent’ (Line: 171)

--L159: Please remove "higher", it does not seem to fit here.

Reply: We have removed the word ‘higher’ from the position (Line: 175)

--L164: "PCA" stands for "Principal Component Analysis", hence, the second "analysis" in the text is redundant.

Reply: We agree with reviewer and have removed the word ‘analysis’ (Line: 179-180)

--L166: Actually, if the data was projected to PC1, there would be quite some overlap. The separation is actually benefiting from _both_ dimension, PC1 _and_ PC2. I would suggest removing the "at PC1" altogether.

Reply: We agree with the reviewer and have removed ‘at PC1’ from this sentence (Line: 181-182).

--L174: "16S rRNA markers" -> "16S rRNA gene markers".

Reply: We have replaced ‘16S rRNA markers’ with ‘16S rRNA gene markers’ (Line: 198).

--L175f: While, indeed, the amplicon and, to some extent, the metagenomic data suggest members of the Prevotellaceae to be enriched in the present cohort, referring to this family as a marker should be supported by quantitative analyses, e.g., statistical analysis of differences in group means (t-Test or WMW-test) or a classification-based approach (feature selection).

Reply: We thank the Reviewer for this observation and suggesting the need for a statistical analysis to support it. We have now performed a feature selection test using Random Forest analysis (Please see Additional File 5: Figure S4) showing the selection of most important features (mean decrease in accuracy > 0.01; mean relative

abundance ≥ 1% in at least one population) and their relative abundance in different populations. The most discriminating features (families) which were able to classify Indian samples from other populations were plotted rank-wise (Additional File 5: Figure S5). The pairwise Wilcoxon rank sum test of important families between India and other populations was performed and represented using box plots (Please see Additional File 5: Figure S6). The analysis has been included in revised manuscript (Line: 199-203).

--L184ff: This paragraph needs to be revised as it currently is hard to read. The sentence in L193f was especially hard to read and I am still unsure about what "The proportion of essential genes covered by top-ranking nine eggNOG clusters" means: What is the meaning of "nine" in this context when the authors refer to 15,000 to 30,000 eggNOG clusters later.

Reply: We apologize for the typo error. We have removed the word “nine” from this statement. We have also revised this paragraph to make it more readable. Please see the changes made in the paragraph (Line: 215-220).

--L196f: It was not readily clear to me what "alpha diversity (Shannon) calculations using gene abundances" meant and I found the Methods lacking on this point. What gene(s) was/were used ? Moreover, Fig. S4's legend mentions "gene proportions". How does this relate to "gene abundances"? It seems, from the Methods, that rarefaction was used, while the remaining information is scarce on this point. However, this is an important point as the sequencing depth in the current study (mean of 4,545,280 reads/sample) is not particularly deep (cf. Table 1) and, hence, gut microbes' genomes may be covered only partially. In the study by Qin et al . (2010), an order of magnitude more reads per sample ("an average of 62.5 million reads") were produced, albeit at rather short sequencing lengths of 75 bp (compared to 150 bp in the current study).

Reply: We apologise for the lack of clarity in this part. We earlier did not use rarefaction at gene level but the entire gene proportions were used to calculate the diversity. We agree that sequencing depth can have large impact on diversity metrics. We have now used raw gene abundance table which were rarefied at a depth of 1,000,000 seqs/sample for n=30 iterations, and the mean Shannon index were calculated and plotted as box plot (Please see Additional File 5: Figure S9) (Kuang et al.; GigaScience; 2017). We have now included this information in the methods section in revised manuscript (Line: 228-230, 770-772).

--L202: What does "Eigen values, and their scores" mean, i.e., what is a "score" here? Moreover, they are spelled "eigenvalues", i.e., in one word. Please correct throughout.

Reply: We have now revised the statement and also corrected the term ‘eigenvalues’ throughout the text as per the suggestions (Line: 235).

--L203: I am not sure if the authors refer here to "szignificantly" in a statistical sense or not. If so, please include respective quantitive results to support this conclusion.

Reply: As you have rightly mentioned, we were referring to a statistically significant observation, and have now provided the FDR Adjusted P-value in this sentence (Line 236-237).

--L206: How was the odds-ratio computed? In the Methods, the description refers to LOC1 and LOC2, albeit, it seemed, i.e., I was not sure, that a comparison of Indian microbiome vs. "Other" microbiome was intended. If this is the case, the authors should clarify this in the Methods, i.e., that not only was LOC1 compared against LOC2 but also "Indian" vs. "Other" (maybe among other pairwise comparisons).

Reply: The Odds Ratio was computed to obtain the enrichment of species/genes between LOC1 and LOC2 as $OR(k) = [\sum_{s=LOC1} A_{sk} / \sum_{s=LOC1}(\sum_{i \neq k} A_{si})] / [\sum_{s=LOC2} A_{sk} / \sum_{s=LOC2}(\sum_{i \neq k} A_{si})]$, and also for enrichment in Indian microbiome compared to other datasets consisting of USA, Denmark and China referred as “OTHERS” : $OR(k) = ([\sum_{s=INDIA} A_{sk} / \sum_{s=INDIA}(\sum_{i \neq k} A_{si})] / [\sum_{s=OTHERS} A_{sk} / \sum_{s=OTHERS}(\sum_{i \neq k} A_{si})])$. We have now provided the details of comparison performed

Powered by Editorial Manager® and ProduXion Manager® from Aries Systems Corporation

in the Methods section (Line: 809-812).

--L216ff: I welcome the careful wording chosen by the authors here. It appears that there is no detailed dietary information available which could have been used to further support the authors' hypothesis, but they might want to highlight this as a window of opportunity for future study, i.e., including something like a food-frequency questionaire to be able to quantitatively assess possible links to diet.

Reply: We thank the reviewer for this suggestion. This is an important point and we have now included it in the revised manuscript (Line: 268-270).

--L227: Could the authors please elaborate on how the "Spearman's correlation coefficient" was used in this context? I would have applied Fisher's exact test here.

Reply: As suggested by the Reviewer, we have now used Fisher’s exact test here. Earlier, the Spearman’s correlations were applied to identify the correlation between KO based and Genus based cluster allocation. Using Fisher’s exact test, we found no differences between Genus level and KO level clustering (Fisher’s exact P-value = 0.6843) in the samples assignment (Line: 275). We have provided the file containing details of cluster allocation for each sample (Please see Additional File 11).

--L235: "16S rRNA" -> "16S rRNA gene"

Reply: We have replaced 16S rRNA with 16S rRNA gene at all the places in revised manuscript.

--L236: The term "PCA" has been used previously, so this is not the place to introduce the abbreviation.

Reply: We agree and have now removed this term (Line: 284-285).

--L240: It was not clear to me if "taxonomic and functional diversity" were combined here or not. However, this is important to clarify as taxonomy and function are only partially linked.

Reply: We agree with the Reviewer that taxonomic and functional diversity are only partially linked. We understand that the text could have led to this confusion. We have now revised the text in manuscript and hope that it would read fine now (Line: 292-293).

--L255: Is this analysis based on amplicon or based on metagenomic sequencing data? L247 indicates the former, while MGS/CAGs are defined based on the latter. Please clarify in the text.

Reply: The results mentioned in line number 300-302 were based on amplicon sequencing data analysis using Phylum abundance, whereas the results in lines 305-314 are based on taxonomic species identified from metagenomic sequencing data using reads mapped to reference genomes. The results in line 314-320 are based on the MGS analysis from clustering of gene abundance profiles. We apologize for this confusion. We have now provided this information in the revised manuscript.

--L260: Please list "the two species".

Reply: We apologize for the confusion. We were referring to the two species mentioned in the previous line. We have now revised the sentence to clearly refer to the above-mentioned two species (Line: 320-321).

--L262: Isn't "high fiber-rich" redundant? I.e., either "diet high in fiber" or "fiber-rich diet".

Reply: We agree with the Reviewer and we have now changed this word to fibre-rich diet (Line: 323)

--L274: The conclusion drawn by the authors about the OPLS-DA results is misleading,

s.a., https://www.ncbi.nlm.nih.gov/pmc/articles/PMC4990351/. Specifically, the OPLS-DA model integrates the class information with the aim to _increase_ the between-class separation. Hence, the separation observed in Fig. 4C may (partially) be a consequence of the method used and not of actual separation being in the data. An unsupervised method should be used to check for the presence of meaningful separation followed by a supervised method to perform quantitative evaluation, e.g., PERMANOVA, to check how much of the variance is explained by the respective covariates.

Reply: We agree with the reviewer that OPLS-DA model integrates class information (in this case location) and increases the between class separation. As per the reviewer's suggestion, in addition to OPLS-DA, we have now performed PERMANOVA on metabolite abundance table to assess the effect of covariates and identify the ones which explain maximum variation. We have now included the results of PERMANOVA in the manuscript (Please see Table 2). Moreover OPLS-DA models using class information for each of the covariates were used to calculate model Q2 which assesses the quality of the measurement for each of the covariate (Please see Table 3). Since invalid models can still produce higher Q2 values due to over-fitting, the class labels were randomly permuted for n=200 iterations and distribution of Q2 values were produced to assess the reliability of the Q2 values. The reliable model should yield significantly higher Q2 values compared to Q2 values generated from models with randomly permuted labels (Please see Additional File 5: Figure S17). Moreover, an unsupervised clustering of metabolite abundance is already performed (Please see Figure 4A), and its polyserial/biserial correlation with different covariates identified PC1 to be correlated with location, and PC2 with the diet (Line: 340-348).

--L298f: I am not sure if I understood the authors' point right here. "result of its inward transport in microbial cells by the BCAA transporters, thus leading to their accumulation in the colon lumen": Do the authors' mean "uptake by the bacteria, i.e., transport into the microbial cell"? If so, I would not expect an accumulation in the lumen as such.

Reply: We apologize for this confusion. We meant “faecal samples” here and not ‘colon lumen’. We have revised this text appropriately in the manuscript (Line: 364-365)

--L305: Where do the authors show this comparison (serum vs. faeces)? Fig. 6A compares Valine and Isoleucine in LOC1 samples and LOC2 samples, but not serum vs. faeces.

Reply: We have now modified figure 6A showing the comparison of BCAA levels in feaces vs serum (Please see revised Fig. 6A)

--L328: "the major pathway utilized by this species for BCAA biosynthesis": I am not sure in how much the metagenomic and metabolomic data in this study allow to draw this statement. Metatranscriptomic and metaproteomic data would likely be needed here. I would thus suggest that the authors qualify/nuance this statement.

Reply: We agree with the reviewer. We have revised this text appropriately mentioning the result rather than drawing any conclusion in the manuscript (Line: 391-395).

--L375ff: The average age of the cohort is rather low (mean of 29.72 years). Age, however, is an important factor for rheumatoid arthritis. Hence, "A probable explanation" could be toned down to "One aspect to this could be ...".

Reply: We thank the Reviewer for this suggestion. We have now revised this statement accordingly (Line: 446-448)

--L419: "isoluecine" -> "isoleucine".

Reply: We have corrected this word (Line: 488).

--L439f: The second part of the sentence is redundant with the first part and could be removed, or vice versa.

Powered by Editorial Manager® and ProduXion Manager® from Aries Systems Corporation

Reply: We have now removed the redundant part from this sentence (Line 508-510).

--L459 - 460: "which appears promising in reducing the metabolic risk factors originating through the interactions between diet and gut microbes to maintain a healthy gut flora": This reads misleading as the "diet" was binary, i.e., "vegetarian" vs. omnivorous" and such a statement likely requires for more fine-grained and specialized studies than were performed in this work. Please adjust accordingly.

Reply: We agree with the reviewer. We have now revised this statement and have toned down the general interpretations at various places in the Discussion section (Line: 512-514).

--L463ff: This entire paragraph reads redundant with the remainder of the Discussion and should thus be removed or substantially shortened.

Reply: We agree with the reviewer. We have now substantially shortened and revised this paragraph in the manuscript (Line: 515-520).

--L599: "non-reference" -> "reference-independent".

Reply: We have corrected this word (Line: 647).

--L610: Could the authors please, in analogy to their HMP+NCBI results, report how many of the remaining genes aligned to UNIREF?

Reply: In total, out of 10,839,539 genes present in the Updated gene catalogue, 2,773,591 genes were taxonomically annotated using NCBI + HMP reference genomes at nucleotide level. The remaining 8,049,540 genes were aligned against UNIREF database, and a total of 4,553,299 genes (56.56%) could be assigned with a taxonomic annotation. We have now mentioned this information in Methods section (Line: 656-660).

--L611f: This sentence should be rephrased.

Reply: We have now rephrased this sentence (Line: 660-662)

--L706f: How was this assessed and where can the interested reader find the results for this statement?

Reply: We have provided results of CHI index and prediction strength in Additional File 9 with the values. The information about these metrics is provided in Methods section (Line: 754-759).

--L709ff: It is not clear how the "Between class analysis" was peformed. The authors should provide the respective details, e.g., which test, implementation etc.

Reply: Between Class Analysis was performed to support the clustering and to identify the drivers of these clusters. The between class analysis is a type of principal component analysis with instrumental variables. As in this case, ‘Location’ is a variable for the separation between LOC1 and LOC2 within India, and “population” for separation between India and other datasets (USA, Denmark and China). It is a supervised projection of data where the distance between predefined classes (example clusters/location) is maximised. We have provided a clear explanation in the manuscript (Line: 761-767)

--L720: Does "geography" refer to "location" (LOC1 or LOC2) here?

Reply: As correctly pointed out by the reviewer, we meant the two locations (LOC1 and LOC2), and have changed the word ‘geography’ with ‘location’ throughout the manuscript (Line: 775)

--L732: Why was the negative correlation not considered?

Reply: We wish to mention that in this analysis, the objective was to observe the

positive association and link them in a network plot. Hence, the negative correlations were not considered. Moreover, plotting negative correlations was not possible in the plot using igraph package in R.

METHODS
--L485: Do you mean the respective table in "Additional_file_1.doc"? Not sure whether this is under the control of the authors, but it should be checked in the proof that the information is consistently named and can be readily found.

Reply: We apologize for this error. We have now changed the name ‘Supplementary Table’ to ‘Additional File 1’ in the revised manuscript. We hope that it could now be easily found.

--L507: "16S rRNA" -> "16S rRNA gene"

Reply: We have corrected this word at all places in the manuscript.

--L534: "phylogenetic distances between reads": Not sure, but did the authors mean "phylogenetic distances between the samples" here?

Reply: The phylogenetic distances were used to calculate Unifrac distances between the samples. The reads used here are the representative sequences from each OTU. Thus, the phylogenetic distances were calculated between each OTU using the representative sequences from OTUs. Using these phylogenetic distances, we calculated Unifrac distances between samples. We have now revised this sentence in manuscript (Line: 578-580, 772-774).

--L539f: How were host-origin reads identified? Which tool, version, and parameters?

Reply: Human reads were identified and removed from each sample using 18mer matches parameter in Best Match Tagger (BMTagger) version 3.101 (http://casbioinfo.cas.unt.edu/sop/mediawiki/index.php/Bmtagger). We have now mentioned this information in methods section (Line: 584-586).

--L561ff: This is probably for the formal proofs, but I would strongly encourage to properly format here as it seems that, e.g, "bi" is supposed to read "b subscript i".

Reply: Thanks for bringing it to our notice. We have now formatted the formula (Line: 610)

--L1037ff: Please check whether "<" and "> are used correctly here." Typically "p < 0.05 is " considered significant and _not_ "P-value>0.05".

Reply: The ‘>’ and ‘<’ are correctly used in Figures 2c, 2d and S3. We used P > 0.05 to show the non-significant dots plotted in ‘Red’ colour. The significant ones are shown in ‘Blue’ colour. We have now mentioned it in the figure legend (Line: 1112-1113).

TABLES
--I do not know whether the information provided in Table 2 necessitates a separate table. I leave this up to the authors to decide and to potentially discus this with the journal.

Reply: We have now removed this table from the manuscript and included PERMANOVA table as Table 2, which was also suggested by the reviewer in an earlier comment. Also, we have now provided Table 3 showing validation of OPLSDA models for each of covariate by generating a distribution of Q2 values from random permutation (n=200) of labels and evaluating the number of Q2 above the model Q2 for each covariate.

FIGURES
--5: "Logs-Odd Ratio" -> "Log-Odds Ratio"

Reply: Thanks for pointing out this typo. We have corrected it in Figure 5.

Powered by Editorial Manager® and ProduXion Manager® from Aries Systems Corporation

	--S6: The labels on the x-axis and y-axis were not readable. Please adjust accordingly. Moreover, I am not sure in how much the "clouds" add value here. They are not further discussed in the text and, hence, could be omitted for clarity.<br><br>Reply: The font-size of labels has been increased and we hope that it would be easily readable now. The clouds show the density of the unique KOs in the two groups. It has now been mentioned in the legends of this figure. The blue cloud represents the local density estimated from the coordinates of orthologous groups (KO).<br><br># LEGENDS<br>--Throughout: Please verify correct use of "16S rRNA" and "16S rRNA gene".<br><br>Reply: We have now changed 16S rRNA to 16S rRNA gene at all places throughout the manuscript.<br><br>--L1015: "MWAS": Shouldn't this be "MGWAS"?<br><br>Reply: Thank you for pointing this type. We have corrected it in the figure legend and also at all places in the manuscript.<br><br>--L1027: What does "Eigen values and their scores" mean, i.e., what is a "score" here?<br><br>Reply: The word ‘score’ has been removed, and ‘Eigen value’ have been replaced with ‘eigenvalue’ at all places in manuscript.<br><br>--L1092ff: This reads more like a discussion/conclusion and I would thus suggest to remove this from the figure legend.<br><br>Reply: The figure legend of Figure 7 has been revised as per the suggestion (Line: 1162-1164).
**Additional Information:**	
**Question**	**Response**
Are you submitting this manuscript to a special series or article collection?	No
**Experimental design and statistics**<br><br>Full details of the experimental design and statistical methods used should be given in the Methods section, as detailed in our Minimum Standards Reporting Checklist. Information essential to interpreting the data presented should be made available in the figure legends.<br><br>Have you included all the information requested in your manuscript?	Yes
**Resources**<br><br>A description of all resources used, including antibodies, cell lines, animals and software tools, with enough information to allow them to be uniquely	Yes

identified, should be included in the Methods section. Authors are strongly encouraged to cite Research Resource Identifiers (RRIDs) for antibodies, model organisms and tools, where possible.<br><br>Have you included the information requested as detailed in our Minimum Standards Reporting Checklist?	
**Availability of data and materials**<br><br>All datasets and code on which the conclusions of the paper rely must be either included in your submission or deposited in publicly available repositories (where available and ethically appropriate), referencing such data using a unique identifier in the references and in the “Availability of Data and Materials” section of your manuscript.<br><br>Have you have met the above requirement as detailed in our Minimum Standards Reporting Checklist?	Yes

Manuscript
Click here to access/download;Manuscript;Revised_Manuscript.docx
Click here to view linked References

Title: The unique composition of Indian gut microbiome, gene catalogue and associated faecal metabolome deciphered using multi-omics approaches

Authors: D.B. Dhakan[1†], A. Maji[1†], A.K. Sharma[1], R. Saxena[1], J. Pulikkan[2], T. Grace[2,3], A. Gomez[4], J. Scaria[5], K.R. Amato[6], V.K. Sharma[1]*

Affiliations: [1]Metagenomics and Systems Biology Laboratory, Department of Biological Sciences, Indian Institute of Science Education and Research Bhopal, India, [2]Department of Genomic Science, Central University of Kerala, India, [3]Division of Biology, Kansas State University USA, [4]Microbiomics Laboratory, Department of Animal Science, University of Minnesota, USA, [5]Animal Disease Research & Diagnostic Laboratory, Veterinary and Biomedical Sciences Department, South Dakota State University, USA, [6]Department of Anthropology, Northwestern University, USA.

Email IDs: darshan@iiserb.ac.in, abhi71084@gmail.com, ashoks773@gmail.com, ritus@iiserb.ac.in, puljobcmi@gmail.com, tonygrace99@gmail.com, gomeza@umn.edu, joy.scaria@sdstate.edu, katherine.amato@northwestern.edu, vineetks@iiserb.ac.in

[†] These authors contributed equally to this work

*Corresponding author

V.K. Sharma: vineetks@iiserb.ac.in

The revised manuscript text has been marked in Pink and Orange colours to indicate the changes made as per the suggestions of reviewer 1 and 2, respectively.

Abstract

Background

Metagenomic studies carried out in the past decade have led to an enhanced understanding of the gut microbiome in human health, however, the Indian gut microbiome is still not well explored. We analysed the gut microbiome of 110 healthy individuals from two distinct locations (North-Central and South) in India using multi-omics approaches, including 16S rRNA gene amplicon sequencing, whole genome shotgun metagenomic sequencing, and metabolomic profiling of faecal and serum samples.

Results

The gene catalogue established in this study emphasizes the uniqueness of the Indian gut microbiome in comparison to other populations. The gut microbiome of the cohort from North Central India, which was primarily consuming a plant-based diet, was found to be associated with *Prevotella*, and also showed an enrichment of Branched Chain Amino Acid (BCAA) and lipopolysaccharide (LPS) biosynthesis pathways. In contrast, the gut microbiome of the cohort from Southern India, which was consuming an omnivorous diet, showed associations with *Bacteroides*, *Ruminococcus* and *Faecalibacterium*, and had an enrichment of Short Chain Fatty Acid (SCFA) biosynthesis pathway and BCAA transporters. This corroborated well with the metabolomics results, which showed higher concentration of BCAAs in the serum metabolome of the North-Central cohort and an association with *Prevotella*. In contrast, the concentration of BCAAs were found higher in the faecal metabolome of the South Indian cohort, and showed a positive correlation with higher abundance of BCAA transporters.

Conclusions

The study revealed the unique composition of Indian gut microbiome, established the Indian gut microbial gene catalogue, and also compared it with the gut microbiomes from other populations. The functional associations revealed using metagenomic and metabolomic approaches provide novel insights on the gut-microbe-metabolic axis, which will be useful for future epidemiological and translational researches.

Keywords: Indian Gut Microbiome, Whole Genome Shotgun, Metagenomics, Metabolomics, Integrated Gene Catalog, Metagenome-Wide Association Study, Core gut microbiome, Short Chain Fatty Acids, Branched Chain Amino Acids

Background

Determining the taxonomic and functional composition of a healthy gut microbiome across different populations is essential for understanding its role in maintaining human health. Several large-scale, world-wide microbiome projects have revealed variability in the gut microbial composition of the healthy individuals due to factors such as mode of delivery, age, geographical location, diet, lifestyle, etc. [1-5]. Most gut microbiome studies have determined microbial taxonomy and functional diversity using 16S rRNA marker gene-based and/or Whole Genome Shotgun (WGS) approaches to understand the functional role of the gut microbiome. However, novel insights on the complex interplay between diet, gut microbes and human health, along with the role of key microbial metabolites, such as Short Chain Fatty Acids and Branched Chain Amino Acids, derived from the microbial fermentation of dietary fibres are beginning to emerge from

recent gut metabolomics studies [6, 7]. Moreover, the direct impact of the microbial metabolome on human health is also becoming apparent from the recent studies focusing on the 'gut microbiome- host metabolism axis' [8]. Therefore, an integrative approach using both metagenome and metabolome-based characterizations of the gut microbiome appears pragmatic for gaining deeper functional and mechanistic insights into the role of gut microbes on human health.

The large-scale studies carried out so far mainly represent the gut microbiome of urban populations primarily from Europe, US and other 'WEIRD' countries (i.e., the Western, Educated, Industrialized, Rich, and Democratic countries) [9, 10]. Only recently, some studies have characterized the human microbiome from diverse ethnic populations and found significant compositional variations compared to microbiome from other previously studied populations [11-14]. India is the seventh largest country in the world and harbours the second largest population spread across multiple geographical locations with enormous diversity in ethnicity, lifestyles and dietary habits. India is a home to the majority of world's vegetarian population but also has an almost equal representation of population consuming animal-based diets. Moreover, the Indian population has the highest prevalence of diabetes in the world [15]. According to the World Health Organization estimates (WHO, 2011), 53% of deaths in India in the year 2008 were attributed to metabolic conditions such as diabetes and cardiovascular diseases, which are predicted to reach ~75% by 2030 [16].

A few studies have investigated the gut microbiome of the Indian population. A recent study by Maji et al. has shown the functional association of human gut microbiome dysbiosis with tuberculosis through a time-course study carried on six tuberculosis patients in India [17]. The other studies were mainly limited by small cohort sizes and amplicon-based (16S rRNA gene)

sequencing and analysis [17-21]. Thus, several large-scale efforts are needed to identify the Indian population-specific microbiome biomarkers, and to understand the impact of gut microbiome on health and disease in the Indian population along with global comparisons.

However, to uncover the enormous gut microbiome diversity inherent in the different sub-populations of India, extensive sampling and analyses are required. Therefore, as the first large-scale study from India, we selected two prominent locations in North-Central India, i.e. LOC1: Bhopal city, Madhya Pradesh, and Southern India, i.e. LOC2: Kerala. The two locations also had very different dietary habits. The Southern-India population (LOC2) diet was consisting of rice, meat and fish, whereas the North-Central population (LOC1) diet was consisting of carbohydrate-rich food including plant-derived products, wheat and trans-fat food (high-fat dairy, sweets and fried snacks). In addition, the 'Human Development Index Report, UNDP' (United Nations Development Programme), India and SRS-based life-table (Sample Registration Survey, 2010-14) has revealed that the citizens from Kerala had the highest life-expectancy rates (>74 years) in India, whereas those in Madhya Pradesh (capital city 'Bhopal') exhibited the lowest (<65 years) [22]. Further, a higher predisposition of the North-Indian population towards diabetes, cardiovascular diseases and hypertension is known, which in contrast is much lower in Southern India, perhaps due to the lifestyle differences in the two regions [15, 23]. Thus, to gain deeper functional insights into the microbiome from these two distinct sub-populations of India, a multi-omics approach was carried out using amplicon-based profiling of taxonomic composition (16S rRNA gene sequencing), whole genome shotgun-based (WGS-based) profiling of metagenome, and GC-MS-based profiling of faecal and serum metabolomic signatures.

Data Description

The two selected locations, Bhopal (LOC1) and Kerala (LOC2) from North-Central and Southern parts of India were about 2,000 kms apart, and provided a distinct representation of the Indian population with respect to diets and lifestyle **(Additional File 1)**. The 110 (62 females, 58 males) individuals recruited in this study were not suffering from any disease as reported by personal medical history and physical examination, and confirmed no exposure to antibiotics for at least one month prior to sampling. All the recruited individuals had an average BMI of 21.16 (±5.23 standard deviation) and an average age of 29.72 (±17.41 standard deviation) and were not diagnosed with any disease at the time of sample collection, and thus were considered as 'healthy' (**Additional File 1**). Moreover they did not have a second-degree relative history of T2D. The recruitment of volunteers, sample collection, and other study-related procedures were carried out by following the guidelines and protocols approved by the Institute Ethics Committee of Indian Institute of Science Education and Research (IISER), Bhopal, India. Each faecal sample was frozen within 30 mins of the collection. The faecal samples were then used for 16S rRNA gene V3 hypervariable region amplicon sequencing, WGS-based metagenomic sequencing, and metabolomic analysis. The serum samples collected from a subset of volunteers were used for GC-MS based metabolomics analysis. The sequencing of V3 hyper-variable region of 16S rRNA gene and shotgun metagenome sequencing from the 110 faecal samples resulted into 54.87 million paired-end reads (503,460 ± 175,547 (mean ± standard deviation) reads/sample) and 499.98 million paired-end reads (4,545,280 ± 1,498,663 (mean ± standard deviation) reads/sample), respectively (Methods, **Additional File 2** and **Additional File 3)**. The metabolomics analysis was also performed on all faecal and subset of serum samples collected from the same healthy participants using GC-MS, and the resultant CDF files were used for further analysis. The data

description of participants and the data generated from each sample is provided in **Additional File 1** under the Metadata information section.

Analyses

Construction of an Indian gut microbial gene catalogue and updated integrated gene catalogue (IGC)

The first step for functional analysis was the construction of an extensive catalogue of gut microbial genes from the Indian population, since it was not previously available. A De Bruijn graph-based assembly of reads resulted in 2,165,507 contigs of length ≥500 bp with a total contig size of 3.086 Gbp representing 68.25% of total reads and a mean N50 value of 2,288bp. To obtain assemblies of low coverage genomic regions or genomes present in the Indian gut microbiome, the reads from each sample were mapped on assembled contigs obtained from their respective sample, and the remaining singletons (unassembled reads) from all the samples were pooled and re-assembled together into additional 45,839 contigs with length ≥500 bp and a total assembled length of 34.68 Mbp. A total of 1,551,581 non-redundant genes were predicted from contigs, which represent the gut microbial gene catalogue of the Indian cohorts.

The integrated gene catalogue (IGC) established by Li et al. in a previous multicohort study consisted of 9,879,896 genes identified from 1,267 gut metagenomes representing multiple populations [24]. A total of 943,395 genes (sharing < 90% identity with IGC) out of 1,551,581 from Indian gut microbial gene catalogue were identified as non-redundant genes and unique to IGC. The IGC was updated to construct an 'Updated-IGC' by adding these 943,395 non-redundant genes from the Indian gene catalogue. The updated-IGC consisting of 10,823,291 non-redundant genes (an 8.8% increase from IGC) was used as the reference gene catalogue for the subsequent

analysis performed in this study. A total of 70.74% (± 3.77% standard deviation) mean mapping coverage of reads from 110 Indian samples (~7.5% increase in the mapping of reads) was observed on the updated-IGC as compared to 63% (± 4.61% standard deviation) mean mapping on IGC (**Fig. 1A** and **Additional File 4**). The datasets from populations of USA (HMP), Denmark (MetaHIT) and China (a study from Qin et al.) mentioned in **Table 1** were used for a comparative analysis of microbiome of Indian population with other populations [7, 10, 25]. The mapping of reads from other three datasets (HMP, MetaHIT and China) on updated-IGC (mean mapping coverage: HMP = 67.74%, China = 77.44% and MetaHIT = 75.21%) did not show a significant ($P < 0.01$) increment compared to their mapping coverage on IGC (mean mapping coverage: HMP (USA) = 66.93%, China = 77.37% and MetaHIT (Denmark) = 75.02%) as observed in **Fig. 1A**. This shows that the addition of subset of non-redundant genes (sharing < 90% identity with IGC) from the Indian gut microbiome to the IGC significantly (FDR Adj. P-value = 10^{-16}; Wilcoxon rank-sum test) increased the mapping percentage of reads from Indian gut microbiome on the updated-IGC as compared to the other datasets.

Identification of taxonomic signatures of Indian gut microbiome

To determine the taxonomic and functional composition of the Indian gut microbiome and to identify Indian-specific gut-microbial signatures, a cross-population comparison was carried out using the 16S rRNA gene hyper-variable region and shotgun metagenomic data from other populations. A reference-independent metagenome-wide association study (MGWAS) was carried out to identify the Indian-specific gut metagenomic markers through a comparison with similar large-scale studies from other populations [26]. The genes from the metagenomic samples of four countries (India, China, USA and Denmark) were clustered (see Methods) into 924 clusters based on their co-occurrence and Pearson correlations ($\rho \geq 0.9$) across samples resulting into 335 MGS

(metagenomic species) having ≥700 genes in each cluster, and 589 CAGs (co-abundance gene groups) consisting of ≥100 genes in each cluster. Out of the 924 metagenomic clusters, 195 could be assigned up to species level using the taxonomic assignment strategy described in Methods. Canberra distances were calculated from MGS/CAG abundance profiles and their Principal Component Analysis (PCA) was carried out using 'countries' as factors for explaining the variance between samples, which showed that the Indian population formed a distinct cluster separated from the other populations in PCA (**Fig. 1B**). It is interesting to note that the samples from the Indian cohort were more widely spread owing to the higher inter-sample Canberra distances between Indian samples (mean = 0.689) as compared to other datasets having average inter-sample distances of 0.61, 0.59 and 0.54 for USA, China and Denmark populations, respectively (**Additional File 5: Figure S1**). This could be attributed to the significant (FDR Adj. P-value = 0.00013) differences in MGS abundance profiles between LOC1 and LOC2 populations as revealed on comparison of their principal coordinates (**Additional File 5: Figure S2**).

Further, the identification of enriched metagenomic species (MGS) from P-values calculated using negative binomial (NB) model-based Wald test (implemented in DESeq2) and Log Odds Ratio showed that the species belonging to the genera *Bacteroides*, *Alistipes*, *Clostridium*, and *Ruminococcus* were depleted in the Indian population (China, Denmark and USA; Log Odds Ratio < -2 and Adj P-value <0.01), whereas the MGS/CAGs annotated as *Prevotella*, *Mitsuokella*, *Dialister*, *Megasphaera*, and *Lactobacillus* were found to be associated with the Indian population (Adj P-value < 0.01; Log Odds Ratio > 2), and were the major drivers for separation of Indian samples from other populations (**Additional File 5: Figure S3; Additional File 6**). Furthermore, the distribution of microbial families across ten different populations was also calculated using 16S rRNA gene markers, which revealed Indian gut microbiome to have the highest abundance of

Prevotellaceae (**Fig. 1C**). The feature selection method applied using random forest along with pairwise Wilcoxon rank-sum test also identified Prevotellaceae to be significantly higher (FDR Adj. P< 0.05) in gut microbiome of Indian cohort compared to the other population datasets except Indonesia (P-value = 0.506) (**Additional File 5; Figure S4, S5 and S6**) where a comparable abundance of Prevotellaceae was present. The high abundance of Prevotellaceae in Indian population underscores its importance as the marker taxa for the Indian cohort.

Microbial functions enriched in the Indian population

Functional comparison of Indian microbiome with other populations was carried out by mapping the genes derived from assembled contigs to the EggNOG database. In total 69,386 EggNOG functions were identified from the Indian gut microbiome, including 2,328 novel functions obtained from clustering the unmapped genes (see Methods). The core microbial functions that are essential for microbial survival and present in almost 80% individuals were used for the functional comparison. The core microbiome was derived using a similar strategy as employed in MetaHIT (see Methods) [25]. A set of 1,890 essential genes from six bacterial species namely, Escherichia coli MG1655I and MG165II, Bacteroides thetaiotaomicron VPI-5482, Pseudomonas PA01, Salmonella enteric serovar Typhi and Staphylococcus aureus NCTC 8325 were obtained and were assigned with eggNOG annotations. The eggNOG abundance profile generated from relative abundance of genes observed in Indian and other population dataset were ranked based on their mean abundance in descending order. The range of eggNOGs that included 85% of the 1,890 essential genes were considered as part of the core microbial eggNOG set for each population dataset and was used for the analysis. Most of the essential genes were included in the top-ranking clusters suggesting that the essential genes are present in higher abundance than the accessory function genes (**Additional File 5: Figure S7**).

The core microbiome of Indian samples was compared with the core microbiome of USA, China and Denmark populations. The proportion of essential genes covered by top-ranking eggNOG clusters showed that 85% of the essential genes could be covered in the least number (15,300) of eggNOGs in the case of Indian population, whereas it was covered by a higher (30,900) number of eggNOGs in the case of USA (20,400), China (19,900) and Denmark populations (**Additional File 5: Figure S8**). These observations suggest that the core functional microbiome of Indian population is less diverse than the other populations. This corroborates well with the alpha diversity (mean Shannon index) calculated using gene abundance tables rarefied at 1,000,000 seqs/sample (for n=30 random iterations), which also showed that the Indian microbiome is significantly (P-value < 10^{-16}) less diverse than the microbiome of the other populations analysed in this study (**Additional File 5: Figure S9**).

In total, 5,588 eggNOGs were characterized as core functions commonly present in the core microbiome of all the four population datasets. The co-inertia (Procrustes) analysis and the eigenvalues calculated from PCA using both core and accessory functions also showed that the Indian gut microbiome was significantly (FDR Adj. P-value = 6.4×10^{-10} , 2×10^{-16} and 0.05 with China, Denmark and USA, respectively for PC1) different from the other datasets (**Fig. 2A & B**). These results also show the uniqueness of Indian gut microbial functions in composition and diversity at both core and accessory levels. The Indian gut microbiome was found to be enriched (FDR Adj. P<0.05, Log Odds Ratio >1.5) in functions for carbohydrate and energy metabolism including degradation of complex polysaccharides and glycogen and was also enriched for enzymes from TCA cycle, which corroborates well with the carbohydrate-rich diet of the Indian population (**Fig. 2C and 2D and Additional File 7:** Enriched KO and EggNOG functions).

Unsupervised clustering of Indian samples and their association with previously identified enterotypes

A study by Arumugam et al. classified the samples from multiple populations into clusters based on genus level profiles, and identified three prominent clusters called enterotypes [2]. In order to identify the enterotypes from Indian gut microbiome, a meta-analysis was performed using genus level abundances of samples from the four nations as used by Arumugam et al. along with the Indian cohort. There were three prominent clusters observed with majority (63.6%) of Indian population falling into enterotype-2, which was primarily driven by *Prevotella*. The analysis revealed differences in the distribution of samples from LOC1 and LOC2, where a higher number of samples from LOC1 (73.5%) were associated with enterotype-2 compared to LOC2 (54%). In contrast, LOC2 samples were associated with enterotype-1 (30.3%) and enterotype-3 (16.07%), which were driven by *Bacteroides* and *Ruminococcus*, respectively (**Fig. 3A; Additional File 8**). An independent microbial abundance-based clustering of Indian samples using Jensen Shannon distances revealed two prominent clusters. The clustering was validated using Calinski Harabasz index (CHI) and prediction strength, which uses a cross-validation approach to validate the robustness of clustering (**Additional File 9**). Cluster 1 was primarily enriched in species from genus *Prevotella* ($P< 10^{-10}$), and Cluster 2 was quite widely spread and was enriched in species belonging to *Bifidobacterium* ($P=10^{-13}$), *Ruminococcus* (P=0.031), *Clostridium* (P=0.04) and *Faecalibacterium* (P= 0.046) (**Additional File 5: Figure S10**, **Additional File 10**). The higher abundance of *Prevotella* in LOC1 and *Bacteroides* in LOC2 in India are perhaps due to the different dietary habits of the two locations. The LOC1 population was mainly consuming a carbohydrate-rich diet comprising of vegetable-based foods and grains, whereas the LOC2 population was consuming a diet consisting of rice, meat and fish. Similar variations in

microbiome diversity due to differences in dietary habits have also been observed in earlier studies [27, 28]. However, to confirm the above observations and to assess the quantitative effect of dietary habits on microbial variations, further longitudinal studies are necessary where detailed dietary information needs to collected through a food-frequency questionnaire.

A similar cluster analysis performed using the functional information derived from the abundance of KEGG Orthologs (KO) also showed the clustering of samples into two distinct clusters, namely C1 and C2 (**Additional File 5: Figure S11)**. In comparison to clusters derived from taxonomic information, only 14 out of 110 samples were placed in different clusters using the functional information showing a significant concordance (P-value = 0.6841; Fisher's exact test; **Additional File 11**). C1 was found enriched in genes coding for enzymes such as β- glucosidase (LOR = 3.364; P-value = 10^{-20}), and α-fucosidase (LOR = 0.73; P=10^{-8}), which are involved in the breakdown of plant-polysaccharides, whereas the genes coding for enzymes such as lipase (LOR = -1.34; P=10^{-12}), carnitine-coA dehydratase (LOR = -1.81; P-value = 0.029) and amino peptidase (LOR = -2.72; P=10^{-10}), which are involved in the metabolism of animal-based diet, were enriched in C2 (FDR Adj. P<0.05) (**Additional File 12**).

To identify the covariates explaining the maximum variations in microbial profiles across samples, unweighted unifrac distances were calculated using phylogenetic distances between OTU reference sequences and OTU table rarefied at 100,000 seqs/sample. The principal component analysis of Unifrac distances and the correlation of loadings for each sample with the covariates using polyserial/biserial correlation identified distinct locations (LOC1 and LOC2) and diet (vegetarian and omnivorous) to be the major covariates explaining the variation in taxonomic diversity between samples (**Additional File 5: Figure S12, Additional File 13**). An ordination of 110 Indian samples using gene abundance profiles from metagenomic data showed location and

diet to be significantly (FDR Adj. P-value < 0.01; Polyserial Correlation) associated with PC1 explaining the maximum variation between samples **(Additional File 5: Figure S13, Additional File 13).** A comparison of functional diversity using gene abundance curves with increasing number of samples performed between the two locations showed that the microbiome profiles of LOC2 populations were more diverse in their composition compared to LOC1 populations (**Additional File 5: Figure S14**). The inter-individual Bray-curtis distances calculated on normalized gene abundance profiles between LOC1 and LOC2 populations also showed significant differences (FDR Adj. P<0.05), where LOC2 population displayed higher inter-individual heterogeneity in their microbial community structure as compared to LOC1 population **(Additional File5: Figure S15)**.

Major differences in the microbiome profiles were apparent at Phylum level (using 16S rRNA gene amplicon sequencing) from the higher Bacteroidetes to Firmicutes ratio (P=0.002) in LOC1 (1.93) compared to LOC2 (0.86), which have been previously reported as a result of differences in dietary habits, i.e. vegetarian or plant-based (carbohydrate-rich) vs. omnivore or animal-based (protein-rich) diets (**Additional File 5: Figure S16**) [29, 30]. Notably, these variations were not attributable to BMI (Spearman's Rank correlation, FDR Adj. P=0.78). Taxonomic profiles generated from metagenomic datasets through reads mapped to reference genomes were compared between the two locations at genus and species level using NB model-based Wald test implemented in DESeq2. *Prevotella and Megasphaera* were observed to be higher in LOC1, whereas *Ruminococcus* and *Faecalibacterium* were higher in LOC2 (FDR Adj. P<0.05, Wilcoxon rank-sum test); (**Fig. 3B).** Within these genera, *P. copri*, *P. stercorea* species were significantly higher in LOC1, whereas *F. prausnitzii* and *R. bromii* belonging to genus *Faecalibacterium* and *Ruminococcus*, respectively were higher in LOC2. In addition, *Akkermansia muciniphila*,

Eubacterium siraeum and *Roseburia hominis* were observed higher in LOC2, and *M. funiformis* and *M. hypermegale* from genus *Megamonas* were higher in LOC1 **(Fig. 3C).** Moreover, the metagenomic species derived from clustering of gene profiles depicted that out of 86 differentially enriched MGS/CAG obtained from MGWAS, the MGS/CAGs annotated to *Prevotella copri* were found enriched in LOC1 (Log Odds Ratio > 2; Adj. P<0.05; 19 MGS/CAG), whereas MGS/CAGs annotated to SCFA producing species such as *Faecalibacterium prausnitzii* and *Roseburia inulinivorans* were enriched in LOC2 (Adj. P<0.05; Log Odds Ratio < -2; 67 MGS/CAG) (**Additional File 14**). Interestingly, both, *F. prausnitzii* and *R. inulinivorans,* species enriched in LOC2 are known SCFA producers, and are regarded as commensals with anti-inflammatory properties [31]. In contrast, *Prevotella*, which was abundant in the LOC1, is known to be associated with fibre-rich diet [32].

Defining the Indian gut metabolome

The analysis of microbial community structure and functions from the two locations having different lifestyle and diet revealed significant insights. Previous studies have shown a direct role of diet in shaping the different gut microbiomes [33]. Thus, to gain deeper insights into the metabolic activity of microbiomes from LOC1 and LOC2 as driven by different diets, faecal metabolites were analysed using a GC-MS-based metabolomics approach. An unsupervised between class analysis of metabolomic profiles separated the samples into three separate clusters, and the robustness was confirmed using prediction strength and Silhouette index (**Fig. 4A and 4B**). Polyserial correlation of covariates showed location to be the major factor explaining the variation at PC1 (FDR Adj. P<0.01) separating Cluster 1 from Cluster 2 and 3. In contrast, vegetarian and omnivorous diet groups emerged as other factors explaining the variation at PC2 (FDR Adj. P<0.01), and separating Cluster-2 from 3 (**Additional File 15**). The Cluster-1 was

associated with LOC1 and showed higher concentration of saturated fatty acids including palmitic acid, stearic acid, and valeric acid. Cluster-3 was associated with LOC2 and showed higher abundances of BCAAs, valine, leucine and isoleucine, and SCFAs, propionate and butyrate concentrations. Cluster-2 was enriched in D-glucose, galactose, mannose, lauric acid and cadaverine (a polyamine associated with meat consumption) [34]. To assess the effect of different covariates on the separation of samples, PERMANOVA was performed (**Table 2**). The location was found to explain maximum variation for separation of samples, whereas diet was the second most important variable in explaining the variance. The OPLS-DA model was used to expose the class separation for each of the covariates using Q^2 values which assesses the quality measurement (**Table 3**). The OPLS-DA models validated by random permutation (n=200) of class labels showed Q^2 values for location and diet to be higher than Q^2 values produced from random permutations with location showing highest Q^2 values (**Additional File 5: Figure S17**). The OPLS-DA model also showed clear separation of samples between locations as class of separation (**Fig. 4C**).

Positive correlation of BCAA transporters with BCAA levels in faecal metabolome

We also identified the marker metabolites, which showed significant (Spearman's correlation, FDR Adj. P<0.05) associations with LOC1 or LOC2. In total, 17 metabolite clusters were identified, of which nine were associated with LOC1, and eight were associated with LOC2 (**Additional File 16**). These marker metabolites showed a positive association with MGS/CAGs. For instance, *Prevotella* annotated clusters correlated significantly with valeric acid and sedoheptulose metabolite markers, which showed a higher relative abundance in LOC1. In contrast, MGS/CAGs belonging to *Faecalibacterium*, *Clostridium*, *Ruminococcus*, and *Alistipes* were positively associated with BCAAs, cadaverine, propanoate and lauric acid in LOC2 (**Fig. 5A**). In addition to the positive association of BCAAs with species enriched in LOC2, a correlation

analysis of significantly different (FDR Adj. P<0.05, DESeq2-based Wald test; **Additional File 17**) functional modules revealed that faecal BCAA abundances were positively correlated with BCAA transporter abundance in LOC2. In contrast, BCAA abundance in the faecal metabolome showed a negative correlation (P<0.05) with BCAA biosynthesis pathways (**Fig. 5B**).

The above observations are significant given that BCAAs are important metabolites involved in glucose homeostasis by stimulating insulin secretion [35]. Higher BCAA levels in the faecal samples could be a result of its inward transport in microbial cells by the BCAA transporters, leading to their accumulation in the microbial cells detected in faecal metabolome. This is concordant with higher relative abundance of *Bacteroides vulgatus* and *Eubacterium sireaeum* in LOC2 compared to LOC1, which are known to harbour higher abundance of BCAA transporters (**Fig.3C**) [8]. Further support for this hypothesis emerged from the correlation of circulating BCAA levels (valine and isoleucine) in serum with the corresponding concentrations in faeces. Interestingly, serum BCAA concentrations were significantly higher in LOC1 individuals as compared to LOC2 individuals, which is in contrast with their BCAA levels in the faecal metabolome (**Fig. 6A**). Thus, one possibility is that the accumulation of BCAA in the faeces of individuals of LOC2 was mediated by the inward transport of BCAA by the gut bacteria. In contrast, the lower BCAA accumulation in gut microbes and a higher BCAA biosynthesis by microbial species and its eventual absorption in serum appears to be a plausible reason for the higher BCAA concentrations in serum of LOC1 population.

Role of *Prevotella copri* in the regulation of BCAA levels

To explore the differences in association of functional pathway modules between the two locations, KOs within each module were correlated with KOs from other modules using Spearman's correlation coefficient. The KOs showing significant differences in correlations

between LOC1 and LOC2 were identified. This differential correlation analysis of BCAA biosynthetic modules with other pathways in LOC1 and LOC2 revealed that BCAA modules were independently driven in LOC1 and LOC2 (Spearman's rank correlation, FDR Adj. $P<0.01$) (**Additional File 5: Figure S18A & B**). To identify the species and the metabolic pathways that contributed most to the BCAA abundance in faecal and serum metabolome profiles, a correlation analysis with iterations leaving each species out was performed for each metabolic module (**Figure 6B**). The species whose removal leads to a maximum change in the correlation of metabolic pathway with metabolite was identified, and was considered as an important contributor of that metabolite.

Notably, the BCAA biosynthesis-dependent changes in BCAA levels were largely driven by *Prevotella* species through threonine-dependent and independent biosynthesis pathways as observed from Delta SCC_{bg} values when genes from this species were removed (see Methods). The correlation network analysis with differential MGS/CAGs revealed threonine-independent isoleucine biosynthesis pathway to be highly correlated with *Prevotella copri* in LOC1 (**Fig. 6C**). The first enzyme, D-citramalate synthase, catalysing the first step of threonine-independent isoleucine biosynthesis pathway was also observed as highly enriched (LOR = 1.7) in LOC1 [36]. Further, BCAA biosynthesis pathways was found higher in LOC1, whereas BCAA transporters were higher in LOC2 (**Fig. 6D**) leading to the dynamic changes in BCAA concentrations in faecal and serum metabolome in LOC1 and LOC2 as observed in Fig. 6A.

Discussion

Compositional and functional human gut microbiome studies in different populations have been instrumental in establishing the role of gut microbiome in human health [2, 28, 37, 38]. However, such population-specific signatures and their functional roles are yet unknown for the Indian gut

microbiome. This study provides the first insights into the Indian gut microbiome represented through a cohort of 110 individuals from two prominent locations to reveal the taxonomic and functional diversity using 16S rRNA gene, metagenomic analysis, and metabolomic profiling. Though, the sequencing depth in the study was not too high (1.5 ± 0.5 Gbp per sample, mean ± standard deviation), but due to the generation of longer paired-end reads (150x2 bp), the sequence data generated from a cohort of 110 individuals appears reasonably enough to provide the first insights on the Indian gut microbiome. Given the high diversity of diet and lifestyle in India, the selection of two distinct locations (Bhopal-LOC1, and Kerala-LOC2) as the representative sub-populations was an important consideration. LOC1 provided a representation of the population from North-Central India mainly consuming a carbohydrate and fat rich diet, whereas LOC2 represented a population from Southern India consuming an omnivorous diet with rice and animal-based products as the primary components.

This study established the gene catalogue of the Indian gut microbiome, which provides the first insights into the yet unknown functional gut microbiome of the Indian population. The genes encoding several transposons, peptidase, glucosidase, and plant polysaccharide degradation enzymes were unique to the Indian population and not represented in other microbiome datasets. The Updated-IGC (IGC+India) constructed by the addition of unique non-redundant genes from the Indian population to the Integrated gene catalogue is likely to act as a reference dataset for gut microbiome studies for global comparative studies, and particularly for studies involving South-Asian populations that have similar dietary habits and lifestyle.

In addition to the basic housekeeping functions of the gut microbiome, which were also found abundant in other datasets, the Indian gut microbiome was enriched in functions for carbohydrate and energy metabolism including degradation of complex polysaccharides, which corroborates

well with the typical carbohydrate-rich diet of the Indian population [39]. The distant clustering of Indian samples from other populations revealed the unique composition of the Indian gut microbiota (**Fig. 1B**). *Prevotella* emerged as the most discriminatory genus associated with the Indian population as revealed by both amplicon and MGWAS. Its abundance was also indicated in the previous 16S rRNA gene-based microbiome studies of the Indian population carried out in small to medium-sized cohorts [18, 19]. Recently, *Prevotella* has been commonly observed in different non-Western communities that consume a plant-rich diet, such as in the Papua New Guineans, native Africans, rural Malawians, BaAka pygmies, etc [11, 40]. and has also been associated with vegetarianism in the Western populations [41, 42]. However, it has not been observed at such high abundance in the western countries so far. The MGWAS approach in this study showed the presence of *Megasphaera*, *Lactobacillus* and *Mitsuokella* as the other major driver genera associated with the Indian gut microbiome.

Several recent studies have shown a relationship between the abundance of specific strains of *Prevotella* with inflammatory diseases, since it has a higher intrinsic capacity to stimulate Th17-mediated inflammation, which is generally not expected in a strict commensal bacteria [41, 43, 44]. However, the high abundance of *Prevotella* in the healthy gut microbiome of the Indian population does not corroborate with its potential inflammatory role reported so far. Since this study was only focussed on the gut microbiome of healthy individuals, it is difficult to draw conclusions on the potential inflammatory role of this species. One aspect to this could be the complex set of interactions between host genetic risk factors and environment in which the presence of *Prevotella* may be only one of the factors [45]. Further, strain-level variations are known in the inflammatory responses and not all species of *Prevotella* could be potentially inflammatory, as also evident from the known high genetic diversity within and between the

species of *Prevotella* [43]. Thus, the high abundance of *Prevotella* in the healthy microbiota emphasizes the requirement for larger cohort studies in different populations to gain deeper insights into the potential inflammatory roles of gut microbes.

The abundance of *Prevotella* has been associated with plant-based diets, and the typical carbohydrate-rich diet of the Indian population could be one of the reasons for the over-representation of this genus in the Indian gut microbiome [46]. Likewise, the predominance of other microbial species from genus *Lactobacillus*, *Megasphaera* and *Mitsuokella* could be due to the higher intake of fermented food and dairy products along with the carbohydrate-rich diet in LOC1 [46, 47]. Similarly, *Bacteroides* and *Clostridium*, which were abundant in LOC2, are associated with diets rich in animal-based products, consistent with the omnivorous diet of LOC2 [42]. Interestingly, taxonomy-based clusters 1 and 2 showed associations with the two locations LOC1 and LOC2, and also with the two KO-based clusters (C1 and C2) (**Additional File 5: Figure S10 and S11**). It is to be noted that C1 was enriched in enzymes involved in the degradation of carbohydrate and plant polysaccharides, which correlates well with the carbohydrate-rich diet in LOC1. In contrast, C2 was enriched in enzymes involved in lipid and protein degradation, which relate to the constituents of an omnivorous diet in LOC2. These observations further support the correlation between location, diet, and enterotype. Although, the concept of enterotype classification is sometimes criticised due to statistical weakness in some studies, however, a meta-analysis of Indian samples with samples from Arumugam et al. revealed three robust clusters with Indian samples mostly associated with enterotype-2 driven by *Prevotella* [2]. This statistically sound classification of samples from multiple population/studies into enterotypes has the potential to be clinically relevant in various aspects such as disease diagnosis, early-detection of disease, biomarker development, personalised treatments and xenobiotic metabolism [48]. It is a

representation of the major microbial species in the gut microbiome, and thus appears useful for microbiome-based population stratification. A robust statistical analysis with increased sample sizes, direct clinical associations, and detailed molecular interventions are essential for further strengthening its potential.

The study also established the previously unknown faecal metabolome of the Indian population, which showed strong clustering into three metabolomic clusters differentiated by location and diet. The metabolomic clusters also correlated well with the respective dietary habits of the two locations, where metabolomic Cluster-1 showed an association with LOC1 and was enriched in saturated fatty acids such as palmitic acid and stearic acid, whereas metabolomic Cluster-3 showed an association with LOC2, and was enriched in BCAAs such as isoleucine, valine and leucine, and SCFAs such as propionic acid, and butyric acid. A medium chain fatty acid (MCFA) 'lauric acid' was also found abundant in LOC2 perhaps due to the high dietary consumption of coconut oil in this location [49, 50]. Lauric acid has known health benefits such as preventing fat deposition in blood vessels and acting as an anti-inflammatory and anti-oxidative agent [51].

The major BCAA 'isoleucine' being produced through a less common threonine-independent pathway for isoleucine biosynthesis, and the higher enrichment of the key enzyme, D-citramalate synthase of the above pathway confirmed its higher abundance in LOC1 as compared to LOC2. Further, this pathway was found to be associated with a single species, *Prevotella copri* as reported earlier [8]. Taken together, it appears that the higher abundance of BCAA biosynthesis genes and a lower abundance of BCAA inward transporters in gut microbiome resulted in the lower BCAA accumulation in the fecal metabolome, and higher BCAA concentration in serum as observed in LOC1 (Fig. 7) [8]. However, a contrasting pattern was observed in the case of LOC2, where the lower abundance of BCAA biosynthesis genes and the higher abundance of BCAA inward

transporters correlated well with the higher and lower BCAA concentrations in faeces and serum, respectively.

The higher levels of SCFAs in LOC2 could be a consequence of the consumption of omnivorous diet, which is associated with a Firmicute-rich gut microbiome [52]. SCFAs have well-established roles in human health as an energy source, an anti-inflammatory agent, and for improving intestinal homeostasis by increasing IL-18 production [53]. In contrast, higher serum BCAA levels have well-known roles in promoting insulin resistance and Type-2 Diabetes (T2D), and were found higher in the serum in LOC1. Several reports on the role of a high-fat diet in the modulation of microbiota and alteration in intestinal barrier are emerging, which results in the increased absorption and circulating levels of branched-chain amino acid (BCAA) and in the reduction of SCFAs such as butyrate, acetate, propionate, and secondary bile acids, as also noted in the case of LOC1 [54, 55]. High-fat and carbohydrate-rich diets have also been associated with an increase in abundance of Bacteroidetes (gram-negative bacteria) leading to a skewed Bacteroidetes: Firmicutes ratio towards the former phylum [32]. Such a ratio was also apparent in this study in LOC1 dominated by *Prevotella* from the phylum Bacteroidetes. Further, a higher serum concentrations of circulating BCAA were also observed in LOC1. These results provide hints on the role of dietary habits in shaping the gut microbiome and its plausible impact on the BCAA and SCFA dynamics observed in these populations.

To conclude, this multi-omics based gut microbiome study of a healthy cohort populations from two different parts of India provides novel insights into the Indian gut microbiome and metabolome, and reveals the unique gene catalogue from the poorly characterized Indian population. Further studies using higher sequencing depths, and including both healthy and

diseased individuals will help in obtaining more comprehensive functional and taxonomic information of gut microbiome from Indian population and its impact on human health.

Methods

Study design and subject enrolment

The study cohort consisted of 110 healthy individuals belonging to different age groups from infants (<1 year) to aged (>50 years), with an average subject age of 29.72 ± 17.4 years (mean ± sd) from two different locations across India i.e., Bhopal (LOC1, n=53) and Kerala (LOC2, n=57), which are separated by ~1000 miles. LOC1 was located in North-Central India with the majority of population being vegetarian, whereas LOC2 was located in Southern India where the population with dietary habits mostly consisting of rice, seafood and red meat (Diet description section in **Additional File 1**). According to the 'Indian Food Composition Table', the primary Indian diet is rich in carbohydrates such as rice, wheat and potato, and in fat and proteins from milk and dairy products [56]. In addition, several accompaniments to the primary diet also exist including a variety of grains, vegetables, fruits, and usage of oil, spices and animal products.

The faecal samples for metagenomics and blood samples for serum metabolomics were collected from healthy participants and their metadata is provided in **Additional File 1** under the Metadata information section. The recruitment of volunteers, sample collection, and other study-related procedures were carried out by following the guidelines and protocols approved by the Institute Ethics Committee of Indian Institute of Science Education and Research (IISER), Bhopal, India. Each faecal sample was frozen within 30 mins of the collection. A written informed consent was obtained from all subjects prior to any study-related procedures, along with information on gender, age, and diet for a period of one month prior to the collection of faecal samples. The recruited

individuals did not undergo any medication at least one month prior to the sample collection. All the recruited individuals had an average BMI of 21.16 (±5.23), and were not diagnosed with T2D at the time of sample collection, and did not have a second-degree relative history of T2D. The above samples were then used for 16S rRNA gene V3 hypervariable region amplicon sequencing, shotgun metagenomic sequencing, and metabolomic analysis.

Faecal metagenomic DNA extraction

Metagenomic DNA was isolated from all the faecal samples using QIAamp Stool Mini Kit (Qiagen, CA, USA) according to the manufacturer's instructions. DNA concentration was estimated by Qubit HS dsDNA assay kit (Invitrogen, CA, USA), and quality was estimated by agarose gel electrophoresis. All the DNA samples were stored at -80 °C until sequencing.

16S rRNA gene amplicon and shotgun metagenome sequencing

The extracted DNA (5ng) was PCR amplified with seven different custom modified 5′-end adaptor-ligated 341F and 534R primers (See the primer details section in Additional File 1) targeting the V3 hypervariable region of 16S rRNA gene. After evaluating the amplified products on 2% w/v agarose gel, the products were purified using Ampure XP kit (Beckman Coulter, Brea, CA USA). Amplicon libraries were prepared by following the Illumina 16S rRNA gene metagenomic library preparation guide. Metagenomic libraries were prepared using Illumina Nextera XT sample preparation kit (Illumina Inc., USA) by following the manufacturer's protocol. Library size of all the libraries was assessed using Agilent 2100 Bioanalyzer (Agilent Technologies, Santa Clara, USA.), and quantified on a Qubit 2.0 fluorometer using Qubit dsDNA HS kit (Life technologies, USA) and by qPCR using KAPA SYBR FAST qPCR Master mix and Illumina standards and primer premix (KAPA Biosystems, Wilmington, MA, USA) following the Illumina suggested protocol. Both the amplicon and metagenomic libraries were loaded on

Illumina NextSeq 500 platform using NextSeq 500/550 v2 sequencing reagent kit (Illumina Inc., USA), and 150 bp paired-end sequencing was performed at the Next-Generation Sequencing (NGS) Facility, IISER Bhopal, India.

Amplicon-based taxonomic analysis

A total of 24 Gbps of data were retrieved on de-multiplexing of paired-end reads with an average of 210 Mbp per sample. The paired-end reads were assembled using FLASH and were quality filtered at Q20 (80% bases) Phred quality score using NGSQC Toolkit v 2.3.3 [57, 58]. The primer sequences were trimmed from the High Quality (HQ) reads. The reads were further clustered into OTUs using closed-reference OTU picking protocol of QIIME at ≥97% identity against ARB SILVA database release 132 (13th December 2017) [59, 60]. The most abundant read was selected as the representative sequence for each OTU and was assigned with taxonomy using the SILVA database. OTU table containing the abundance of each OTU for each sample was generated and used for further analysis. For phylogenetic analysis, representative 16S rRNA genes of phylotypes were aligned against a core set of 16S rRNA gene sequences using align_seqs.py with the PyNAST v.1.2.2 algorithm [61]. The unweighted unifrac distances between samples were calculated using rarefied OTU abundance (100,000 seqs/sample) table and phylogenetic distances between representative sequences from each OTUs [62].

Pre-processing of the Metagenomic reads

A total of 150 Gbp of metagenomic sequence data (mean = 1.36 Gb) was generated from 110 faecal samples. The metagenomic reads were filtered using NGSQC toolkit v2.3.3 with a cutoff ≥Q20 [57]. The high-quality reads were further filtered to remove the host-origin reads (human contamination) from bacterial metagenomic reads using 18mer matches parameter in Best Match Tagger BMTagger v3.101 (http://casbioinfo.cas.unt.edu/sop/mediawiki/ index.php/Bmtagger),

which resulted in the removal of an average of 1% reads. The reads from each sample were assembled separately into contigs using IDBA ud version 1.1.0 [63] with parameters "-mink 31 –maxk 87 –step 5". The reads from each samples were mapped to contigs to estimate read recruitment using FR-HIT version 0.7 [64]. The unmapped reads resulting from each sample were pooled together and denovo assembly was performed on the combined set of singleton (unmapped) reads from all samples. The ORFs from each contig (length ≥ 500bp) were predicted using MetaGeneMark v.3.38 [65]. Pair-wise alignment of genes was performed using BLAT version 2.7.6 [66], and the genes which had an identity ≥ 95% and alignment coverage ≥ 90% were clustered into a single set of non-redundant genes, from which the longest gene was selected as the representative ORF to construct the non-redundant gene catalog.

Integrated Gene Catalog (IGC), which represents 1,297 human gut metagenomic samples comprising of HMP, MetaHIT and Chinese datasets, was retrieved [24]. The gene catalogue constructed from Indian samples was combined with the IGC to construct a non-redundant gene catalog (using identity ≥ 95% and alignment coverage ≥ 90%) and is referred to as 'Updated-IGC' in the subsequent analysis.

Quantification of gene content

The quantification of gene content was carried out using the strategy performed by Qin et al., [7] where the high-quality reads were aligned against the updated IGC using SOAP2 in SOAP aligner version 2.21 with an identity cut off ≥ 90% [67]. Two types of alignments were considered for sequence-based profiling:

(1) The entire paired-end read mapped to the gene.

(2) One end of paired-end read mapped to a gene and other end outside genic region.

In both cases, the mapped read was counted as one copy.

The relative abundance of a gene within the sample was calculated as: $a_i = \frac{b_i}{\sum j b_j}$

a_i: relative abundance of gene in sample S; x_i: The times in which gene i was detected in sample S (the number of mapped reads); b_i: copy number of gene i in sequenced data from sample S.

Phylogenetic assignment of reads

A total of 4,097 reference microbial genomes were obtained from Human Microbiome Project (HMP) and National Centre for Biotechnology Information (NCBI) on 5th December 2015 (**Additional File 18**). The databases were independently indexed into two Bowtie indexes using Bowtie-2 version 2.2.9 [68]. The metagenomic reads were aligned to the reference microbial genomes using Bowtie-2. The mapped reads from both indexes were merged by selecting the alignment having the higher identity (≥ 90% identity). The percent identity was calculated using the formula: %identity = 100*(matches/total aligned length). The normalized abundance of a microbial genome was calculated by summing the total number of reads aligned to its reference genome. For reads showing hits to both indexed databases with equal identity, each genome was assigned 0.5 read count. The relative abundance of each genome was calculated by adding the normalized abundance of each genome divided by the total abundance. The Calinski Harabasz index (CHI) was used to calculate the variance between the clusters compared to the variance within clusters [2].

Construction of common core microbial functions

To identify the core microbial functions in the gut microbiome of Indian populations and to understand their abundance compared to the other populations, the core microbiome was

constructed using a similar strategy as mentioned in MetaHIT [25]. However, to construct a comprehensive core functional microbiome, the information of essential functions from six different microbes including two strains of *Escherichia coli*, *Bacteroides thetaiotaomicron*, *Pseudomonas aeruginosa*, *Salmonella enteric* and *Staphylococcus aureus*, was used instead of considering a single microorganism. The list of essential genes was collected from DEG database v5.0 [69]. 1,890 genes were identified as essential genes in all the six microorganisms. These genes were aligned against eggnog v4.1 database using diamond and were annotated with eggNOG ID [70, 71]. The core gut microbiome functions were also calculated using the above strategy for the USA, Denmark and Chinese population gut microbial samples to remove the variations arising due to differences in data analysis procedures. Apart from identifying the clusters that represented ≥85% genes within the range of essential gene functions, the low prevalent eggNOG functions, which were present in ≥ 0.0001% abundance in ≥ 80% of samples in that population, were further filtered out. This added filtration step helped in removing all the low abundant functions. To represent the core, the variance of these functions was also calculated between the two Indian locations. The eggNOGs showing significant deviations in variations (P-value≤ 0.05; Levene's test) [72] were further filtered out from the analysis.

Construction of Metagenomic Species for MGWAS

To identify metagenomic markers using a reference-independent approach on metagenomic samples, a metagenome-wide association study was performed for 340 samples (age and gender matched) including India (both locations), USA, China and Denmark populations. The genes present in at least ≥10% of samples were considered and clustered using the canopy-mgs algorithm as described [73]. The genes having Pearson's correlation coefficient (≥0.9) were clustered into

CAGs. Furthermore, the genes for which ≥ 90% abundance was obtained from a single sample were discarded.

To determine the taxonomic origin of each MGS/CAG (metagenomic cluster), all the genes were aligned against reference microbial genomes of 4,097 genomes from HMP and NCBI at nucleotide level using BLASTN [74]. The alignment hits were filtered using an E-value $\leq 10^{-6}$ and alignment coverage ≥ 80% of the gene length, and 2,773,591 (25.6%) genes showed alignments against the reference genomes. The remaining 8,049,540 unassigned genes were aligned against UNIREF database (UniRef 50) at protein sequences [75], of which 4,553,299 genes (56.56%) could be assigned with taxonomic annotations. The sequences that found multiple top hits with equal % sequence identity and scores were further assigned taxonomy based on LCA (Lowest Common Ancestor) method. The genes were finally assigned to taxa based on comprehensive parameters of sequence similarity across phylogenetic ranks as described earlier [76]. The identity threshold of ≥95% was used for assignment up to species level, ≥85% identity threshold for assignment up to genus level, and ≥65% identity was used for phylum level assignment using BLASTN. The taxonomic assignments of MGS/CAGs were performed with the criteria that ≥50% genes in each MGS should map to the same lowest phylogenetic group. Thus, if a particular species is assigned ≥50% genes out of the total genes, the assignment will be carried out at species level rather than at genus or higher orders. The relative abundance of MGS/CAGs in each sample was estimated by using relative abundance values of all genes from that MGS/CAG. A Poisson distribution was fitted to the relative abundance values of the data. The mean estimated from Poisson distribution was assigned as the relative abundance of that MGS. The profile of MGS/CAGs were generated and used for further analysis.

Faecal and Serum metabolomic sample preparation and derivatization

Lyophilized faecal samples were used to achieve better metabolite coverage as described previously [77]. Metabolites were extracted with 1 mL of ice-cold methanol: water (8:2) from 80 mg of lyophilized samples in a bath ultrasonicator (Bioruptor TM UCD-200, Diagenode, USA) at 4°C for 30 min followed by 2 min of vortexing. The supernatant was extracted by centrifugation at 18,000 g for 15 min at 4°C and dried at 50°C under a gentle stream of nitrogen gas. To remove the residual water molecules from the samples, 100uL of toluene was added to the dry residue and evaporated completely at 50°C under nitrogen gas. Dry extracted metabolites were first derivatized with 50 uL of methoxyamine hydrochloride (MOX) in pyridine (20 mg/mL) at 60°C for 2 hours, and the second derivatization was performed with 100 uL of MSTFA in 1% TMCS at 60°C for 45 min to form trimethylsilyl (TMS) derivatives. Finally, 150 uL of the TMS derivatives was transferred into a GC glass vial inserts and subjected to GC/TOFMS analysis. Serum samples were prepared (polar metabolites only) and derivatized as described by Psychogium et al., 2011 [78].

Method development and validation

Matrix dilution approach was used for validating the linearity and range of dilution [77]. Pooled faecal samples were used to create the reference peaks to validate the peaks coming from individual samples, which were needed due to the presence of a relatively high abundance of faecal metabolites in the pooled samples. The supernatant of feces after extraction was serially diluted 2, 5, 10, 50, 100, 200 and 500 times with methanol: water (8:2). At dilution 2, the maximum numbers of peaks were seen and were processed with the same dilution factor for all the samples. A total of 30 chemical standards mixture and the pooled faecal samples were used to validate the method. Each stock solution of test standard was carefully prepared in deionized water or with pure ethanol (50,150 350, 500 um) for the determination of linear range, regression coefficient (R2), limit of

detection (LOD), and repeatability. L-norvaline (1, 2.5, 5, 10, 20 mg/ml in ethanol) was used as a spiked external standard for the optimized derivatization of the method.

GC-MS analysis

GC-MS was performed on an in-house Agilent 7890A gas chromatograph with 5975C MS system. An HP-5 (25 m × 320 um × 0.25 um i.d.) fused silica capillary column (Agilent J&W Scientific, Folsom, CA) was used with the open split interface. The injector, transfer line and ion source temperatures were maintained at 220, 220 and 250 °C, respectively. Oven temperature was programmed at 70°C for 0.2 min, and increased at 10°C/min to 270°C where it was sustained for 5 min, and further increased at 40°C/min to 310°C where it was held for 11 minutes. The MS was operated in the electron impact ionization mode at 70eV. Mass data were acquired in full scan mode from m/z 40 to 600 with an acquisition rate of 20 spectra per second. To detect retention time shifts and enable Kovats retention index (RI) calculation, a standard Alkane series mixture (C10–C40) was injected periodically during the sample analysis. RIs are relative retention times normalized to n-alkanes eluted adjacently. For serum samples, we used 2uL aliquot with a split ratio of 4:1 on the same column as described above. The injector port temperature was held at 250°C, and the helium gas flow rate was set to 1mL/min at an initial oven temperature of 50°C. The oven temperature was increased at 10°C/min to 310°C for 11min and mass data were acquired in full scan mode from m/z 40 to 600 with an acquisition rate of 20 spectra per second.

Metabolomic analysis and metabolite profile generation

Raw CDF files were used for peak identification and filtering, and the XCMS package in R were used for pre-processing of the peaks. First, the parameters used for pre-processing of the reads were optimized by calculating the reliability index using the formula given below:

Reliability index = (number of reliable peaks)^2 /number of unreliable peaks.

The reliable peaks were identified for each of the settings such as fwhm, S/N and bw, with a predefined range of values and regression coefficient was calculated for dilutions of QC samples. The number of peaks with a high coefficient of determination ($R^2 \geq 0.9$) were considered reliable, whereas the peaks with very low R^2 (≤ 0.05) were considered unreliable peaks [79]. The finally optimized parameters were: profmethod = bin, method = matched Filter, fwhm =8 and 5 for faecal and serum samples, respectively, and S/N = 12 and 3 for faecal and serum samples, respectively, bw =5 (for first grouping), smooth = linear, family = gaussian, extra = 1, plot type = mdevden, missing =8, bw = 3 (for second grouping). Further, to compare across multiple samples, the peak intensities were normalized (root transformed) and scaled using z-transformation. These normalized and scaled peak intensities were used for further statistical analysis.

A multivariate statistical method, Orthogonal Projections to Latent Structures Discriminant Analysis (OPLS-DA) [80], was used to identify differences between LOC1 samples (n=53) and LOC2 (n=55) samples. Metabolites driving the differences were identified in metabolic profiles of LOC1 and LOC2 samples using correlations coefficients. The clusters of co-abundant metabolite profiles were identified using R package "WGCNA" [81]. Signed weighted metabolite co-abundance correlation after scaling and centering was calculated across all samples. The soft threshold of β = 15 was chosen for scale-free topology. The dynamic hybrid tree cutting algorithm was used to identify the clusters with a deepsplit = 4 and minimum cluster size = 4. The profile of each faecal metabolite cluster was summarized using eigenvector. The abundance profile of each cluster of metabolites (MES) was calculated using the same methodology as used for MGS cluster abundance profiles.

Retention index (RI) calculation

GC-MS data obtained from the alkene series run was used to calculate the RI for each peak in the samples, and the obtained RI values were further used at the time of library search for the identification of individual metabolite.

$$I = 100\ X\ [n + (logtx - \log tn)/\ (\log tn + 1 - \log tn)$$

Where, tx = retention time of the peak, tn = retention time of preceding alkane, and tn+1 = retention time of the following alkane.

Clustering and enterotype Analysis

Cluster of samples in the dataset were identified from the relative abundance profiles of Genus or Orthologous groups (OG) in the samples. The Jensen-Shannon distances (which estimates the probability distributions between the samples) were calculated and the abundance profiles were clustered using PAM (partitioning around medoids) clustering algorithm as mentioned previously [82]. The optimal number of clusters was assessed using Calinski Harabasz index (CHI) that has shown good performance in recovering the optimal number of clusters [83]. Similarly, the prediction strength from 'fpc' package in R which used cross-validation approach was also employed as another metric for cluster validation. Both the CHI and prediction strength showed quite significantly correlated results. For clustering, CHI and prediction strength gave non-identical values, silhouette index was calculated to estimate the robustness of clusters.

Between class analysis

The between class analysis was performed to identify the drivers and support the clustering of the genus/species/OG abundance profiles into clusters. The between class analysis is a type of principal component analysis with instrumental variables which maximizes the separation between

classes of this variable. The instrumental variables here is the cluster classification using PAM clustering and the top species, which contributed the maximum to the principal components obtained from between class analysis were identified as driver species/genus/OG based on their eigenvalues. The analysis was performed using ade4 package in R.

Diversity Analysis

The inter-sample Canberra distances were also calculated using MGS Abundance between populations. The richness of microbiome samples across populations was obtained from Shannon index calculated using raw gene abundance table rarefied at equal depth (1,000,000 seqs/sample) over n=30 random samplings. The beta diversity for 16S rRNA genes (between the samples) was calculated as unweighted UniFrac distances using OTU tables rarefied at 100,000 seqs/sample and phylogenetic distance between representative sequences from each OTU [84]. The effect of covariates such as age, diet, location (LOC1 and LOC2) and gender were compared for correlation with principal components identified from principal component analysis using UniFrac distances. The polyserial correlations with P-values were calculated for categorical variables and the significance of the covariates for explaining the variation was estimated at each principal component.

Network Analysis

Spearman's rank correlations were computed between each of the species/MGS and the between MGS and functional modules/metabolites. The correlations with significant P-values were selected and were used for the network analysis. The undirected links were generated between correlated nodes (species/KOs/modules) and the strength of the links were given weights based on their correlation coefficients. The network structure was generated using "igraph" package in R. The modularity of the network for KOs association was generated with each module representing the

functional modules defined in KEGG database. The negative correlation was not considered in generating the network modules. Moreover, the positive correlations were filtered ($\rho \geq 0.6$) for most of the network analysis.

Supervised learning

Predictive models were built using supervised machine learning algorithm Random Forest (RF)[85]. The models were optimized using 10,000 trees and default settings of mtry (number for variables used to build the model). The mean three-fold cross-validation error rates were calculated for each of the binary tree and the ensemble of trees. The mean decrease in accuracy, which is the increase in error rates on leaving the variable out, was calculated for each prediction and tree and was used to estimate the importance score. The variables showing a higher mean decrease in accuracy of prediction were considered important for the segregation of the datasets into groups based on the categorical variable.

Statistical Analysis

All the statistical comparisons between groups were performed using Negative Binomial model-based Wald test implemented in DESeq2 and non-parametric Wilcoxon Rank-Sum Test with FDR Adjusted P-Values to control for multiple comparisons [86-88]. The correlations between two variables and the correlations within were calculated using Spearman's Correlation Coefficient with Adjusted P-Values [89]. The correlations between categorical and numeric variables were performed using Polyserial correlation/biserial correlations [90]. To identify the enrichment of enzymes/species associated with a host, Odds Ratio was used as a measure of the enrichment of a feature in a group. The Odds Ratio was calculated as $OR(k) = [\sum_{s=LOC1} A_{sk} / \sum_{s=LOC1}(\sum_{i \neq k} A_{si})] / [\sum_{s=LOC2} A_{sk} / \sum_{s=LOC2} (\sum_{i \neq k} A_{si})]$ for enrichment of genes/species between two locations, where A_{sk} denotes abundance of species/gene k in sample S. Also the enrichment of species/genes

between Indian microbiome compared to other datasets consisting of USA, Denmark and China referred as "OTHERS" were computed as OR(k) $([\sum_{s=INDIA} A_{sk} / \sum_{s=INDIA}(\sum_{i \neq k} A_{si})] / [\sum_{s=OTHERS} A_{sk} / \sum_{s=OTHERS} (\sum_{i \neq k} A_{si})])$. All the graphs and plots were generated using the ggplot2 package in R.

Correlation analysis between functional modules and metabolite clusters

To calculate the association of microbial functional modules with faecal metabolite clusters, the Spearman's correlation coefficients were calculated to rank KOs for association with metabolite clusters and Metabotypes. To quantify the shift in Spearman correlation between given KEGG module and the metabolite cluster compared to the background distribution, the background adjusted median Spearman's correlation was calculated for a given KEGG module m as:

$$SCC_{bg.adj} = \text{median}\ (SCC_{KOs\ \epsilon\ KEGG\ Module\ m}) - \text{median}\ (SCC_{KOs\ KEGG\ Module\ m})$$

Where SCC_{KO} is the partial Spearman's correlation coefficient between KO and the metabolite cluster.

Identification of microbial species driving the association between KEGG Module and metabolite abundance was done by iterating the correlation between KO belonging to the KEGG module and the metabolite after excluding the genes annotated to that KO from each species. The change in median Spearman's correlation coefficient between the KOs and the metabolite, when genes from that species are excluded from the analysis, was calculated as described previously [8]. The species showing the maximum change in the overall correlation of module with metabotype was plotted.

List of abbreviations

Indian Gut Microbiome (IGM), Enterotypes (ET), Integrated Gene Catalog (IGC), Metagenome-Wide Association Study (MGWAS), Short Chain Fatty Acids (SCFAs), Branched Chain Amino Acids (BCAAs).

Declarations

Collection of Datasets for Comparative analysis

The 74 HMP metagenomes were collected from http://hmpdacc.org/HMASM or NCBI SRA (accession SRR059347). The 85 Danish fecal metagenomes from METAHIT were obtained from European Nucleotide Archive (http://www.ebi.ac.uk/ena, study accession number ERP000108). The 71 Chinese metagenome samples were obtained from NCBI SRA (accession number – SRR341581).

Ethics approval and consent to participate

The recruitment of volunteers, sample collection, and other study-related procedures were carried out by following the guidelines and protocols approved by the Institute Ethics Committee of Indian Institute of Science Education and Research (IISER), Bhopal, India. A written informed consent was obtained from all the subjects prior to any study-related procedures.

Consent for publication

Not applicable

Availability of data and materials

The datasets generated and/or analysed during the current study have been deposited in the National Centre for Biotechnology Information (NCBI) BioProject database under the project number PRJNA397112, and is publicly available for academic use.

Competing interests

The authors declare that they have no competing interests.

Funding

This work was supported by the intramural funding received from IISER Bhopal, Madhya Pradesh, India.

Author’s contributions

VKS and AM conceived the work and participated in the design of the study. AM and JP collected all the samples in collaboration with TG. AM designed the study protocols and performed sample processing, DNA extraction, metabolite extraction and profiling from faecal and blood samples. RS and AM carried out the library preparation and sequencing work. DBD carried out all metagenomic data and statistical analysis. AKS and DBD analyzed the metabolomics data. AM and DBD did the primary data interpretation of analytical outcomes under the supervision of VKS. AM, DBD, AKS, RS, AG, JS, KRA and VKS drafted the manuscript. All authors read and approved the final manuscript.

Acknowledgments

The sequencing and computational analysis were performed at the NGS Facility and HPC and computing facility, respectively, at IISER Bhopal. DBD, AM, RS and JP received fellowships from the UGC (University Grants Commission), Centre for Research on Environment and Sustainable Technologies (CREST, IISER Bhopal), DST-INSPIRE and Central University of Kerala, respectively.

References:

1. Turnbaugh PJ, Ley RE, Hamady M, Fraser-Liggett CM, Knight R and Gordon JI. The human microbiome project. Nature. 2007;449 7164:804-10.
2. Arumugam M, Raes J, Pelletier E, Le Paslier D, Yamada T, Mende DR, et al. Enterotypes of the human gut microbiome. Nature. 2011;473 7346:174-80.
3. Yatsunenko T, Rey FE, Manary MJ, Trehan I, Dominguez-Bello MG, Contreras M, et al. Human gut microbiome viewed across age and geography. Nature. 2012;486 7402:222-7.
4. Dominguez-Bello MG, Costello EK, Contreras M, Magris M, Hidalgo G, Fierer N, et al. Delivery mode shapes the acquisition and structure of the initial microbiota across multiple body habitats in newborns. Proc Natl Acad Sci U S A. 2010;107 26:11971-5.
5. Saxena R and Sharma V. A metagenomic insight into the human microbiome: Its implications in health and disease. Medical and Health Genomics. Elsevier; 2015. p. 107-19.
6. Schwiertz A, Taras D, Schafer K, Beijer S, Bos NA, Donus C, et al. Microbiota and SCFA in lean and overweight healthy subjects. Obesity (Silver Spring). 2010;18 1:190-5.
7. Qin J, Li Y, Cai Z, Li S, Zhu J, Zhang F, et al. A metagenome-wide association study of gut microbiota in type 2 diabetes. Nature. 2012;490 7418:55-60.
8. Pedersen HK, Gudmundsdottir V, Nielsen HB, Hyotylainen T, Nielsen T, Jensen BA, et al. Human gut microbes impact host serum metabolome and insulin sensitivity. Nature. 2016;535 7612:376-81.
9. Gupta VK, Paul S and Dutta C. Geography, Ethnicity or Subsistence-Specific Variations in Human Microbiome Composition and Diversity. Front Microbiol. 2017;8:1162.
10. Human Microbiome Project C. Structure, function and diversity of the healthy human microbiome. Nature. 2012;486 7402:207-14.
11. Gomez A, Petrzelkova KJ, Burns MB, Yeoman CJ, Amato KR, Vlckova K, et al. Gut Microbiome of Coexisting BaAka Pygmies and Bantu Reflects Gradients of Traditional Subsistence Patterns. Cell Rep. 2016;14 9:2142-53.
12. Obregon-Tito AJ, Tito RY, Metcalf J, Sankaranarayanan K, Clemente JC, Ursell LK, et al. Subsistence strategies in traditional societies distinguish gut microbiomes. Nat Commun. 2015;6:6505.
13. Schnorr SL, Candela M, Rampelli S, Centanni M, Consolandi C, Basaglia G, et al. Gut microbiome of the Hadza hunter-gatherers. Nat Commun. 2014;5:3654.
14. Hasler R, Kautz C, Rehman A, Podschun R, Gassling V, Brzoska P, et al. The antibiotic resistome and microbiota landscape of refugees from Syria, Iraq and Afghanistan in Germany. Microbiome. 2018;6 1:37.
15. Mohan V, Sandeep S, Deepa R, Shah B and Varghese C. Epidemiology of type 2 diabetes: Indian scenario. Indian J Med Res. 2007;125 3:217-30.
16. Mushtaq MU, Gull S, Abdullah HM, Shahid U, Shad MA and Akram J. Waist circumference, waist-hip ratio and waist-height ratio percentiles and central obesity among Pakistani children aged five to twelve years. BMC Pediatr. 2011;11:105.
17. Maji A, Misra R, Dhakan DB, Gupta V, Mahato NK, Saxena R, et al. Gut microbiome contributes to impairment of immunity in pulmonary tuberculosis patients by alteration of butyrate and propionate producers. Environ Microbiol. 2018;20 1:402-19.
18. Pulikkan J, Maji A, Dhakan DB, Saxena R, Mohan B, Anto MM, et al. Gut Microbial Dysbiosis in Indian Children with Autism Spectrum Disorders. Microb Ecol. 2018;76 4:1102-14.
19. Bhute S, Pande P, Shetty SA, Shelar R, Mane S, Kumbhare SV, et al. Molecular Characterization and Meta-Analysis of Gut Microbial Communities Illustrate Enrichment of Prevotella and Megasphaera in Indian Subjects. Front Microbiol. 2016;7:660.

20. Shetty SA, Marathe NP and Shouche YS. Opportunities and challenges for gut microbiome studies in the Indian population. Microbiome. 2013;1 1:24.
21. Tandon D, Haque MM, R S, Shaikh S, P S, Dubey AK, et al. A snapshot of gut microbiota of an adult urban population from Western region of India. PLoS One. 2018;13 4:e0195643.
22. Suryanarayana M, Agrawal A and Prabhu KS. Inequality-adjusted human development index for India's states. United Nations Development Programme (UNDP) India. http://www. undp. org/content/dam/india/docs/inequality_adjusted_human_development_index_for_indias_state 1. pdf [NS], 2011.
23. Misra A, Pandey RM, Devi JR, Sharma R, Vikram NK and Khanna N. High prevalence of diabetes, obesity and dyslipidaemia in urban slum population in northern India. Int J Obes Relat Metab Disord. 2001;25 11:1722-9.
24. Li J, Jia H, Cai X, Zhong H, Feng Q, Sunagawa S, et al. An integrated catalog of reference genes in the human gut microbiome. Nat Biotechnol. 2014;32 8:834-41.
25. Qin J, Li R, Raes J, Arumugam M, Burgdorf KS, Manichanh C, et al. A human gut microbial gene catalogue established by metagenomic sequencing. Nature. 2010;464 7285:59-65.
26. Wang J and Jia H. Metagenome-wide association studies: fine-mining the microbiome. Nat Rev Microbiol. 2016;14 8:508-22.
27. Tyakht AV, Kostryukova ES, Popenko AS, Belenikin MS, Pavlenko AV, Larin AK, et al. Human gut microbiota community structures in urban and rural populations in Russia. Nat Commun. 2013;4:2469.
28. Liang C, Tseng HC, Chen HM, Wang WC, Chiu CM, Chang JY, et al. Diversity and enterotype in gut bacterial community of adults in Taiwan. BMC Genomics. 2017;18 Suppl 1:932.
29. Aleksandrowicz L, Tak M, Green R, Kinra S and Haines A. Comparison of food consumption in Indian adults between national and sub-national dietary data sources. Br J Nutr. 2017;117 7:1013-9.
30. Joy EJ, Green R, Agrawal S, Aleksandrowicz L, Bowen L, Kinra S, et al. Dietary patterns and non-communicable disease risk in Indian adults: secondary analysis of Indian Migration Study data. Public Health Nutr. 2017;20 11:1963-72.
31. Rios-Covian D, Ruas-Madiedo P, Margolles A, Gueimonde M, de Los Reyes-Gavilan CG and Salazar N. Intestinal Short Chain Fatty Acids and their Link with Diet and Human Health. Front Microbiol. 2016;7:185.
32. Kovatcheva-Datchary P, Nilsson A, Akrami R, Lee YS, De Vadder F, Arora T, et al. Dietary Fiber-Induced Improvement in Glucose Metabolism Is Associated with Increased Abundance of Prevotella. Cell Metab. 2015;22 6:971-82.
33. De Filippo C, Cavalieri D, Di Paola M, Ramazzotti M, Poullet JB, Massart S, et al. Impact of diet in shaping gut microbiota revealed by a comparative study in children from Europe and rural Africa. Proc Natl Acad Sci U S A. 2010;107 33:14691-6.
34. Ruiz-Capillas C and Jimenez-Colmenero F. Biogenic amines in meat and meat products. Crit Rev Food Sci Nutr. 2004;44 7-8:489-99.
35. Layman DK. The role of leucine in weight loss diets and glucose homeostasis. J Nutr. 2003;133 1:261S-7S.
36. Drevland RM, Waheed A and Graham DE. Enzymology and evolution of the pyruvate pathway to 2-oxobutyrate in Methanocaldococcus jannaschii. J Bacteriol. 2007;189 12:4391-400.
37. Wu GD, Chen J, Hoffmann C, Bittinger K, Chen YY, Keilbaugh SA, et al. Linking long-term dietary patterns with gut microbial enterotypes. Science. 2011;334 6052:105-8.
38. Cho I and Blaser MJ. The human microbiome: at the interface of health and disease. Nat Rev Genet. 2012;13 4:260-70.

39. Green R, Milner J, Joy EJ, Agrawal S and Dangour AD. Dietary patterns in India: a systematic review. Br J Nutr. 2016;116 1:142-8.
40. Martinez I, Stegen JC, Maldonado-Gomez MX, Eren AM, Siba PM, Greenhill AR, et al. The gut microbiota of rural papua new guineans: composition, diversity patterns, and ecological processes. Cell Rep. 2015;11 4:527-38.
41. Ley RE. Gut microbiota in 2015: Prevotella in the gut: choose carefully. Nat Rev Gastroenterol Hepatol. 2016;13 2:69-70.
42. Losasso C, Eckert EM, Mastrorilli E, Villiger J, Mancin M, Patuzzi I, et al. Assessing the Influence of Vegan, Vegetarian and Omnivore Oriented Westernized Dietary Styles on Human Gut Microbiota: A Cross Sectional Study. Front Microbiol. 2018;9:317.
43. Larsen JM. The immune response to Prevotella bacteria in chronic inflammatory disease. Immunology. 2017;151 4:363-74.
44. Scher JU, Sczesnak A, Longman RS, Segata N, Ubeda C, Bielski C, et al. Expansion of intestinal Prevotella copri correlates with enhanced susceptibility to arthritis. Elife. 2013;2:e01202.
45. Renz H, von Mutius E, Brandtzaeg P, Cookson WO, Autenrieth IB and Haller D. Gene-environment interactions in chronic inflammatory disease. Nat Immunol. 2011;12 4:273-7.
46. Tremaroli V and Backhed F. Functional interactions between the gut microbiota and host metabolism. Nature. 2012;489 7415:242-9.
47. Selhub EM, Logan AC and Bested AC. Fermented foods, microbiota, and mental health: ancient practice meets nutritional psychiatry. J Physiol Anthropol. 2014;33:2.
48. Costea PI, Hildebrand F, Arumugam M, Backhed F, Blaser MJ, Bushman FD, et al. Enterotypes in the landscape of gut microbial community composition. Nat Microbiol. 2018;3 1:8-16.
49. Jaarin K, Norliana M, Kamisah Y, Nursyafiza M and Qodriyah HMSJECC. Potential role of virgin coconut oil in reducing cardiovascular risk factors. 2014;20 8:3399-410.
50. Boemeke L, Marcadenti A, Busnello FM, Gottschall CBAJOJoE and Diseases M. Effects of coconut oil on human health. 2015;5 07:84.
51. Intahphuak S, Khonsung P and Panthong AJPb. Anti-inflammatory, analgesic, and antipyretic activities of virgin coconut oil. 2010;48 2:151-7.
52. Nicholson JK, Holmes E, Kinross J, Burcelin R, Gibson G, Jia W, et al. Host-gut microbiota metabolic interactions. Science. 2012;336 6086:1262-7.
53. Boulange CL, Neves AL, Chilloux J, Nicholson JK and Dumas ME. Impact of the gut microbiota on inflammation, obesity, and metabolic disease. Genome Med. 2016;8 1:42.
54. Neis EP, Dejong CH and Rensen SS. The role of microbial amino acid metabolism in host metabolism. Nutrients. 2015;7 4:2930-46.
55. Li X, Shimizu Y and Kimura I. Gut microbial metabolite short-chain fatty acids and obesity. Biosci Microbiota Food Health. 2017;36 4:135-40.
56. Longvah T, Anantan I, Bhaskarachary K and Venkaiah K. Indian food composition tables. National Institute of Nutrition, Indian Council of Medical Research; 2017.
57. Patel RK and Jain M. NGS QC Toolkit: a toolkit for quality control of next generation sequencing data. PloS one. 2012;7 2:e30619.
58. Magoc T and Salzberg SL. FLASH: fast length adjustment of short reads to improve genome assemblies. Bioinformatics. 2011;27 21:2957-63.
59. Quast C, Pruesse E, Yilmaz P, Gerken J, Schweer T, Yarza P, et al. The SILVA ribosomal RNA gene database project: improved data processing and web-based tools. Nucleic Acids Res. 2013;41 Database issue:D590-6.
60. Kuczynski J, Stombaugh J, Walters WA, Gonzalez A, Caporaso JG and Knight R. Using QIIME to analyze 16S rRNA gene sequences from microbial communities. Curr Protoc Bioinformatics. 2011;Chapter 10:Unit 10 7.

61. Caporaso JG, Bittinger K, Bushman FD, DeSantis TZ, Andersen GL and Knight R. PyNAST: a flexible tool for aligning sequences to a template alignment. Bioinformatics. 2010;26 2:266-7.
62. Lozupone C, Lladser ME, Knights D, Stombaugh J and Knight R. UniFrac: an effective distance metric for microbial community comparison. ISME J. 2011;5 2:169-72.
63. Peng Y, Leung HC, Yiu SM and Chin FY. IDBA-UD: a de novo assembler for single-cell and metagenomic sequencing data with highly uneven depth. Bioinformatics. 2012;28 11:1420-8.
64. Niu B, Zhu Z, Fu L, Wu S and Li W. FR-HIT, a very fast program to recruit metagenomic reads to homologous reference genomes. Bioinformatics. 2011;27 12:1704-5.
65. Zhu W, Lomsadze A and Borodovsky M. Ab initio gene identification in metagenomic sequences. Nucleic acids research. 2010;38 12:e132-e.
66. Kent WJ. BLAT--the BLAST-like alignment tool. Genome Res. 2002;12 4:656-64.
67. Li R, Yu C, Li Y, Lam T-W, Yiu S-M, Kristiansen K, et al. SOAP2: an improved ultrafast tool for short read alignment. Bioinformatics. 2009;25 15:1966-7.
68. Langmead B and Salzberg SL. Fast gapped-read alignment with Bowtie 2. Nature methods. 2012;9 4:357.
69. Zhang R and Lin Y. DEG 5.0, a database of essential genes in both prokaryotes and eukaryotes. Nucleic acids research. 2008;37 suppl_1:D455-D8.
70. Buchfink B, Xie C and Huson DH. Fast and sensitive protein alignment using DIAMOND. Nature methods. 2014;12 1:59.
71. Powell S, Forslund K, Szklarczyk D, Trachana K, Roth A, Huerta-Cepas J, et al. eggNOG v4. 0: nested orthology inference across 3686 organisms. Nucleic acids research. 2014;42 D1:D231-D9.
72. Lim T-S and Loh W-Y. A comparison of tests of equality of variances. Computational Statistics & Data Analysis. 1996;22 3:287-301.
73. Nielsen HB, Almeida M, Juncker AS, Rasmussen S, Li J, Sunagawa S, et al. Identification and assembly of genomes and genetic elements in complex metagenomic samples without using reference genomes. Nat Biotechnol. 2014;32 8:822-8.
74. Altschul SF, Gish W, Miller W, Myers EW and Lipman DJ. Basic local alignment search tool. Journal of molecular biology. 1990;215 3:403-10.
75. Suzek BE, Huang H, McGarvey P, Mazumder R and Wu CH. UniRef: comprehensive and non-redundant UniProt reference clusters. Bioinformatics. 2007;23 10:1282-8.
76. Huson DH, Auch AF, Qi J and Schuster SC. MEGAN analysis of metagenomic data. Genome research. 2007;17 3:377-86.
77. Phua LC, Koh PK, Cheah PY, Ho HK and Chan ECY. Global gas chromatography/time-of-flight mass spectrometry (GC/TOFMS)-based metabonomic profiling of lyophilized human feces. Journal of Chromatography B. 2013;937:103-13.
78. Psychogios N, Hau DD, Peng J, Guo AC, Mandal R, Bouatra S, et al. The human serum metabolome. PloS one. 2011;6 2:e16957.
79. Gao X, Pujos-Guillot E, Martin J-F, Galan P, Juste C, Jia W, et al. Metabolite analysis of human fecal water by gas chromatography/mass spectrometry with ethyl chloroformate derivatization. Analytical biochemistry. 2009;393 2:163-75.
80. Worley B and Powers R. Multivariate Analysis in Metabolomics. Curr Metabolomics. 2013;1 1:92-107.
81. Langfelder P and Horvath S. WGCNA: an R package for weighted correlation network analysis. BMC Bioinformatics. 2008;9:559.
82. Kaufman L and Rousseeuw PJ. Partitioning around medoids (program pam). Finding groups in data: an introduction to cluster analysis. 1990:68-125.

83. Liao M, Li Y, Kianifard F, Obi E and Arcona S. Cluster analysis and its application to healthcare claims data: a study of end-stage renal disease patients who initiated hemodialysis. BMC Nephrol. 2016;17:25.

84. McMurdie PJ and Holmes S. Waste not, want not: why rarefying microbiome data is inadmissible. PLoS Comput Biol. 2014;10 4:e1003531.

85. Liaw A and Wiener MJRn. Classification and regression by randomForest. 2002;2 3:18-22.

86. Wilcoxon F. Individual comparisons of grouped data by ranking methods. Journal of economic entomology. 1946;39 2:269-70.

87. Benjamini Y, Drai D, Elmer G, Kafkafi N and Golani I. Controlling the false discovery rate in behavior genetics research. Behavioural brain research. 2001;125 1-2:279-84.

88. Love M, Anders S and Huber W. Differential analysis of count data–the DESeq2 package. Genome Biol. 2014;15 550:10.1186.

89. Kendall MG. Rank correlation methods. 1955.

90. Olsson U, Drasgow F and Dorans NJ. The polyserial correlation coefficient. Psychometrika. 1982;47 3:337-47.

Table 1. Metagenomic datasets used for comparative analysis (Meta-analysis) of the microbiome and MGWAS

Dataset	No. of samples	Sequence data (GB)	No. of genes
INDIA	110	110	4,809,378
USA	74	441	6,521,885
DENMARK	85	103.87	7,141,214
CHINA	71	180.78	5,464,702

Table2. PERMANOVA to assess the effect of Covariates on metabolomics profiles of samples

Variable	Sum of Sq	Mean Sq	F-Model	R^2	P-value
**Location**	0.05841	0.058406	4.9423	0.04455	0.0009
**Diet**	0.04701	0.04701	4.2132	0.03586	0.0009
**Age**	0.01618	0.01618	1.4505	0.0123	0.161
**Gender**	0.00488	0.00488	0.4370	0.00373	0.927

Table3. OPLS-DA model and its validation for different covariates as class of separation

Variable	R^2X	Q^2 (cumulative)	pR^2	pQ^2
**Location**	**0.165**	**0.205**	**0.005*** **	**0.005*** **
Diet	0.168	0.123	0.005***	0.005***
Age	0.155	-0.00067	0.075	0.065
Gender	0.106	-0.247	0.145	0.96
Cluster (Genus based)	0.16	0.15	0.005***	0.005***

pR^2 and pQ^2 show p-values for validation of OPLS-DA model with p value < 0.01 shown as significant (*)

Figure title and legends

Fig. 1. Comparison of Indian gut microbiome with other major populations using 16S rRNA gene and metagenomic datasets. (A) Percentage of total reads that could be mapped to IGC and updated IGC containing Indian gene catalogue. Plotted are interquartile ranges (IQR in boxes), median (as dark lines in the boxes), lowest and highest values within 1.5 times the IQR (shown as whiskers extending from boxes) and outliers as points beyond these whiskers. The blue and red boxes showed percentage of reads mapped to IGC and updated IGC (containing the Indian microbial genes). **(B)** Principal Component Analysis using MGS/CAG proportion derived from MGWAS. The samples are plotted along with the MGS/CAGs having taxonomic annotations. The MGS/CAGs are coloured according to their phylum. Variations across populations are shown using PC1 and PC2 along with factor loadings of major MGS/CAGs as biplots. **(C)** Illustration of proportions of bacterial families in different populations and their composition as determined from 16S rRNA gene datasets (adult population only). The mean family compositions of abundant families (≥1%) are represented in separate pie plots from 10 different country-wise datasets, showing their overall microbial composition compared to Indian population.

Fig. 2. Functional variations and differences between Indian populations and other populations determined from core & accessory microbial functions. (A) Procrustes analysis was performed on Bray Curtis distances calculated from core EggNOG and accessory EggNOG abundance tables in all populations. PCA analysis shows the concordance of core and accessory functions in India, Denmark, USA and China populations. The red and black lines are associated with core and accessory datasets, respectively. **(B)** Eigenvalues calculated from PCA of samples using core EggNOGs and accessory EggNOGs are plotted. The boxplots showing for core and accessory eigenvalues for all samples in different populations are shown. Each box plot represents

the median shown as white line between the boxes, the upper and lower ends of the boxes representing upper quartile (75th percentile) and lower quartile (25th percentile). The whiskers extending on both the ends represent 2.5* IQR (Inter Quartile Range). The different coloured dots overlaid for each sample are plotted over the box. The enrichment or depletion of (C) Eggnog, and (D) Kegg functions in India compared to other populations are shown as volcano plots. The log-transformed FDR Adj. P-values calculated from negative binomial-based Wald test from DESeq2 are plotted on the x-axis. The log odds ratio calculated for India vs Other datasets are plotted on the y-axis. The EggNOGs/KOs with P-value<0.05 are shown in Blue whereas those having P-values>0.05 are shown in Red. The EggNOGs/KOs extending on right and left side and with P-value>0.05 are labelled as highly enriched in India and other datasets, respectively.

Fig. 3. Variations in gut microbiome at the two locations. (A) Between class Analysis, which visualizes results from PCA and clustering, using genus level abundance from 37 cross national dataset and genus abundance of 110 Indian samples obtained from mapping of reads to reference genomes. The samples from LOC1 (cyan), LOC2 (pink) and 37 cross national samples from Arumugam et al. (grey and labelled) are placed into three distinct enterotypes based on clustering. **(B)** Significantly different genera (FDR Adj. P-value < 0.05; NB model-based Wald test) between the two locations are shown as boxplots with boxes representing interquartile range (IQR), dark lines between the boxes representing median values and whiskers representing the 1.5 x IQR on each side. **(C)** Scatterplot of log-transformed mean values of species abundance in LOC1 (n=53) and LOC2 (n = 57) individuals. Red colour gradient points represent differentially abundant (FDR Adj. P< 0.05; NB model-based Wald test) species with lower p-values from Red to Blue.

Fig. 4. Between class analysis to identify metabotypes and their associated metabolites. (A) Metabolite clusters (MES) abundance profiles of samples were generated and their clustering was

performed using PAM (partition around medoids) clustering. The between class and PCA of JSD distances and PAM clustering identified 3 clusters to be optimum for their segregation using **(B)** Silhouette index. The metabolites valeric acids, and saturated fatty acids such as palmitic acid and stearic acid, were found higher in Cluster1. The carbohydrates such as glucose and galactose were found higher in Cluster2. The branched chain amino acids, lauric acid and butyric acid were found higher in Cluster3. **(C)** OPLS-DA analysis using locations as classes shows locations as differentiating factors in separating the samples based on their metabolomic profiles.

Fig. 5. Spearman’s Rank correlations of metabolites with species and metabolic modules. (A) Spearman's Rank Correlation coefficients were calculated between significantly different metagenomic species and significantly different metabolites between LOC1 and LOC2 populations. The correlations showing significant FDR Adj. P <0.05 are plotted. The bars on the right show the Log Odds Ratio of the abundance of MGS with positive values indicating enrichment in LOC1, and the negative values indicating enrichment in LOC2. **(B)** Spearman's Rank correlations between significantly different (FDR Adj. P<0.05, NB model-based Wald test) pathway modules and significantly different metabolite abundances in all samples. The significant (P<0.05) correlations are plotted and the colour intensities depict the correlation coefficients. The correlation of metabolites with locations is shown with labels in dark red colours showing association with LOC2, and the labels in green colours showing correlation with LOC1.

Fig. 6. BCAA abundance and their differential correlation with LOC1 and LOC2. (A) Bar plot showing z-normalized values of serum and faecal BCAA (Valine and Isoleucine) relative concentration in LOC1 and LOC2. **(B)** The effect of specific microbial species on associations between BCAA biosynthesis pathways and BCAA levels in faecal metabolome, illustrated by change in background adjusted Spearman’s correlation coefficient when a given species has been

1151 excluded from analysis is shown (see Methods). The density plot shows the distribution of correlation for species and the changes caused by specific species as marked by lines below. **(C)** Network analysis of Spearman's correlations between the branched chain amino acids biosynthesis, degradation and transport KEGG modules with MGS abundance in both LOC1 and LOC2 populations. The node size is proportional to the degree of interactions and the links between module and MGS show interactions or significant correlations (FDR Adj. P < 0.05) with negative (in Red) and positive (in Blue) correlation coefficients. **(D)** Plot showing relative abundance of KOs associated with different modules of BCAA biosynthesis and transporters in LOC1 and LOC2.

Fig. 7. BCAA transporters playing a key role in maintaining the levels of BCAAs in faeces and serum

The dynamics of BCAA concentration levels in faecal and serum metabolome influenced by microbial BCAA biosynthesis and transport pathways and their differential abundance in LOC1 and LOC2 is shown

Additional Files

Additional File 1: Supplementary data containing the metadata and sample information

Additional File 2: Summary of sequencing statistics showing the number of reads per sample for 16S rRNA gene amplicon dataset

Additional File 3: Summary of sequencing statistics showing the number of reads per sample for Whole Genome Shotgun metagenomic dataset

Additional File 4: Summary of the reads mapped to Integrated Gene Catalogue and Indian catalogue combined with IGC.

Additional File 5: Figures S1 to S18

Additional File 6: Differentially abundant MGS between India and other populations

Additional File 7: Differentially abundant functions (Kegg Orthologues (KOs) and EggNOGs) between India and other populations.

Additional File 8: Sample-wise representation of Indian samples into Enterotypes identified from Meta-analysis with 37 samples from four nations used in Arumugam et al.

Additional File 9: Calinski Harabasz index and prediction strength calculated for clusters derived from 16S rRNA gene based genus abundance, metagenome based species abundance and metagenome based KO abundance profiles.

Additional File 10: Mean relative abundance of genus in Cluster-1 and Cluster-2 and their associated P-values of difference calculated using NB model based Wald test.

Additional File 11: The sample-wise association into clusters using Genus based and KO based clustering and their differences.

Additional File 12: Differentially abundant KEGG orthologue functions between Cluster-1 and Cluster-2.

Additional File 13: Polyserial correlation of covariates with principal components explaining variations across samples using unweighted UniFrac distances.

Additional File 14: Differentially abundant MGS observed between two locations and their enrichment calculated using Log Odds ratio and NB model based P-values.

Additional File 15: Polyserial correlation of covariates with principal components explaining variations across samples using metabolomics data.

Additional File 16: Table shows the Spearman's rank correlation coefficient values of metabolites with Metabotypes.

Additional File 17: Table shows the differential abundance of KEGG Modules between LOC1 and LOC2

Additional File 18: List of reference genomes from NCBI and HMP databases for reference mapping

Fig.1

Click here to access/download;Figure;FIGURE 1.jpg

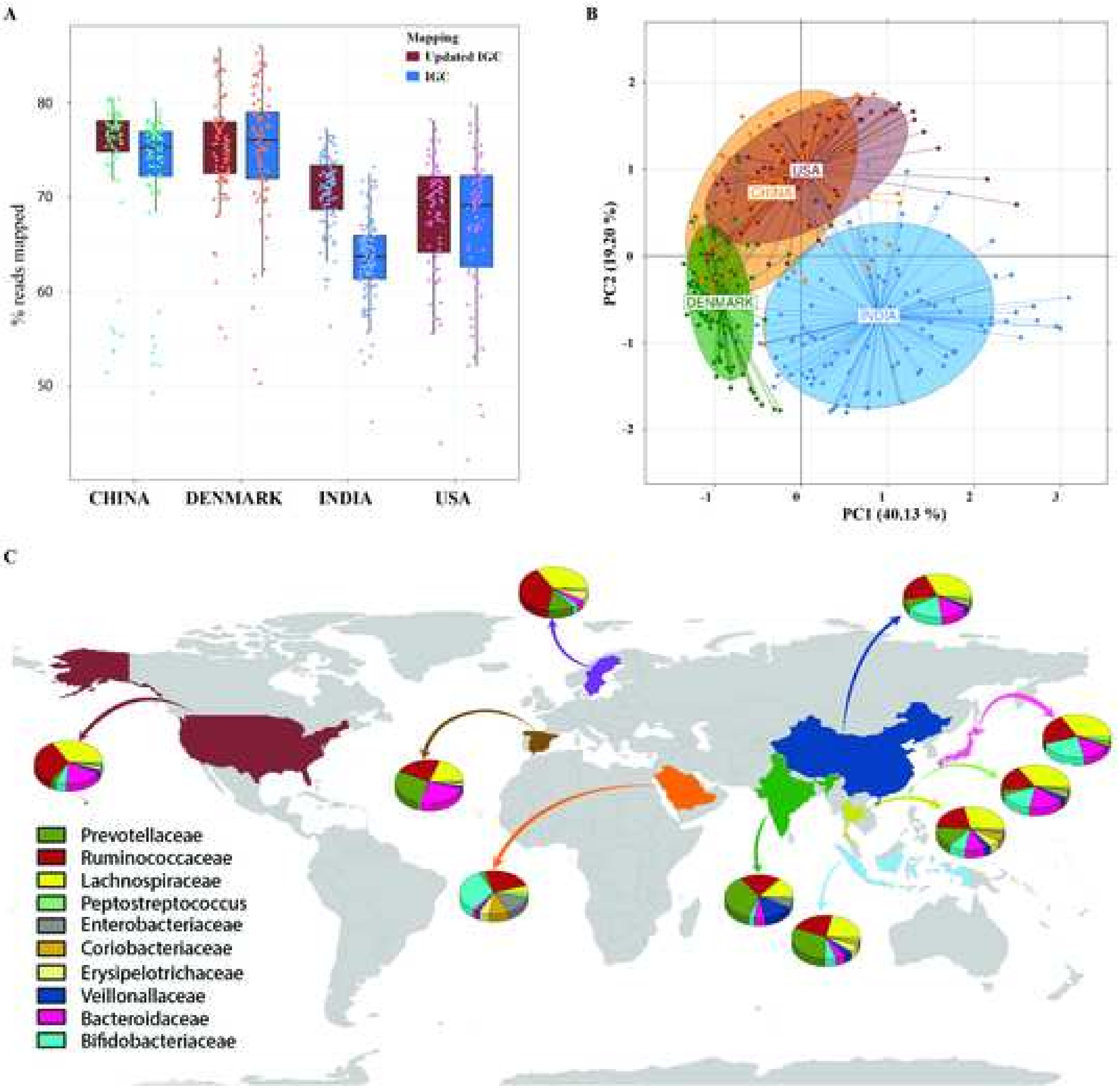

Fig.2

Click here to access/download;Figure;FIGURE 2.jpg

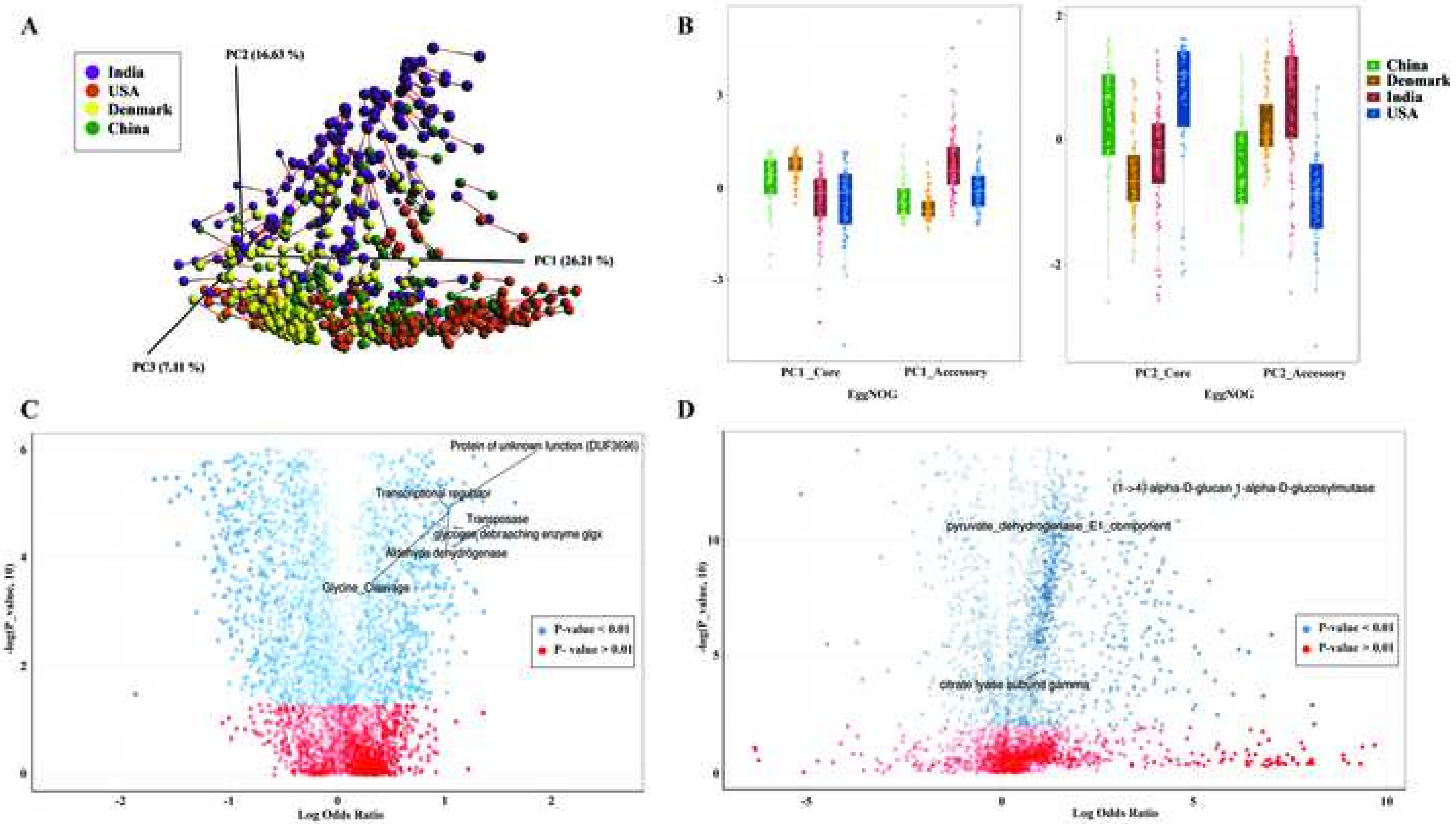

Fig.3

Click here to access/download;Figure;FIGURE 3.jpg

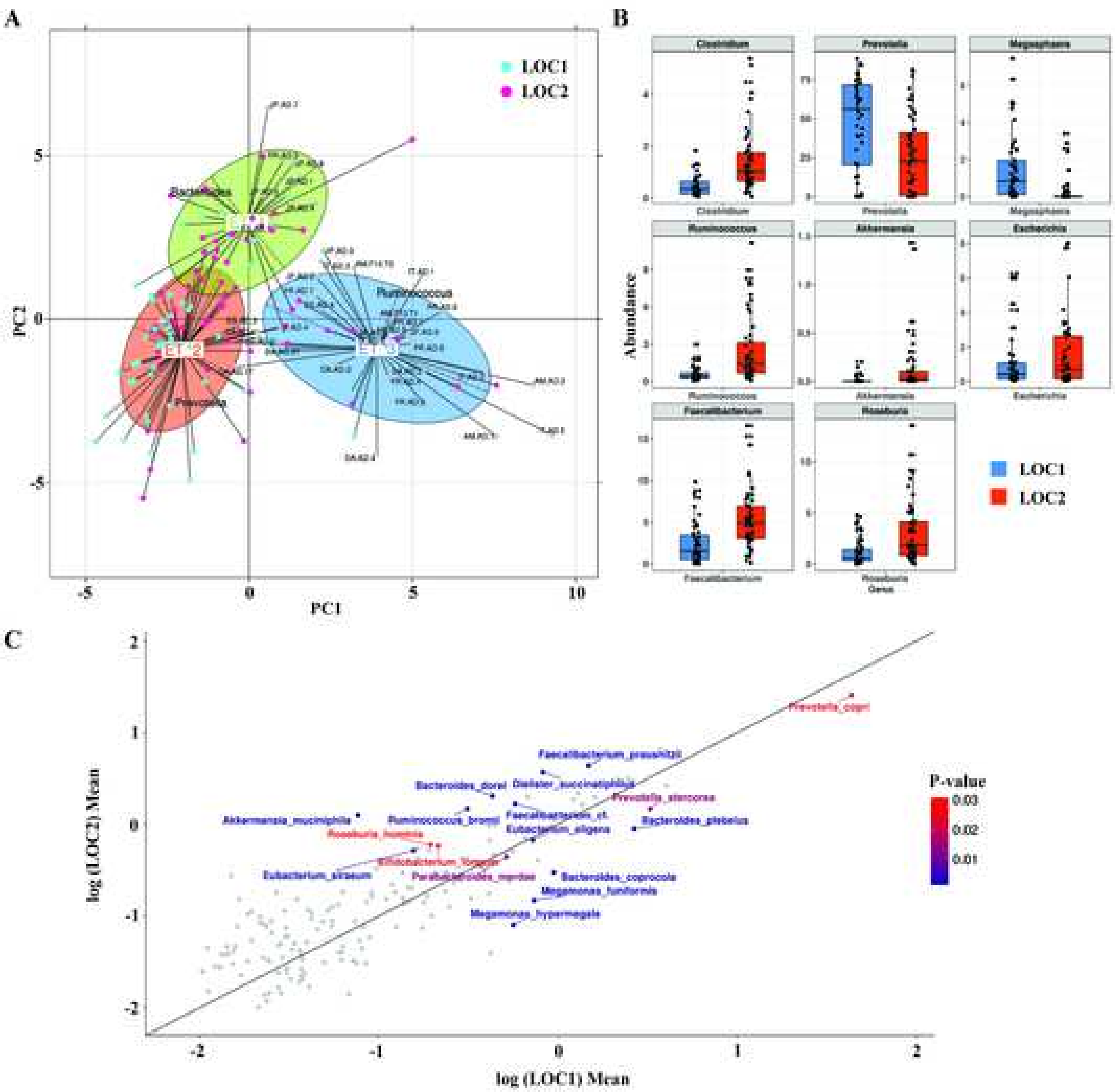

Fig.4

Click here to access/download;Figure;FIGURE 4.jpg

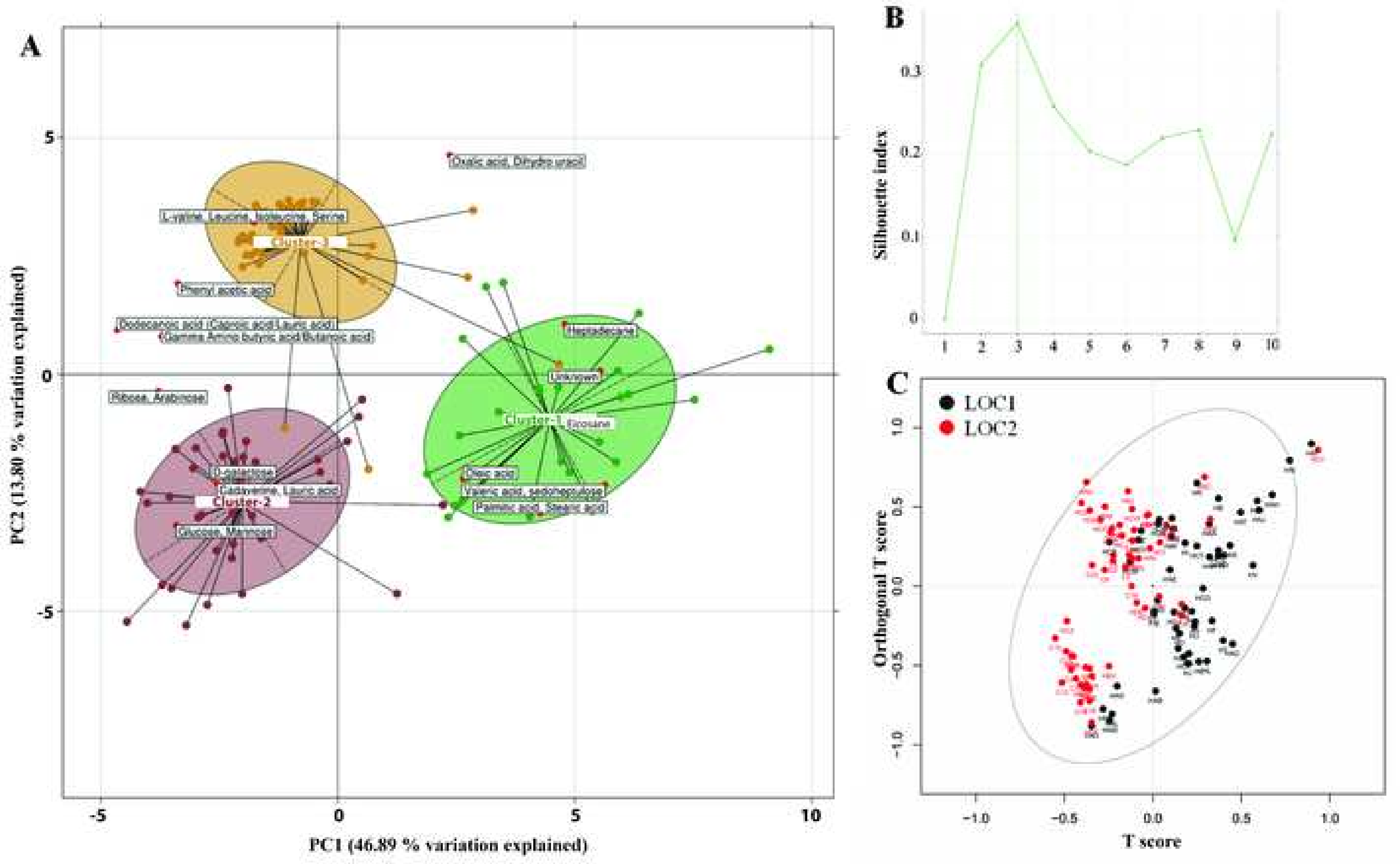

Fig.5

Click here to access/download;Figure;FIGURE 5.jpg

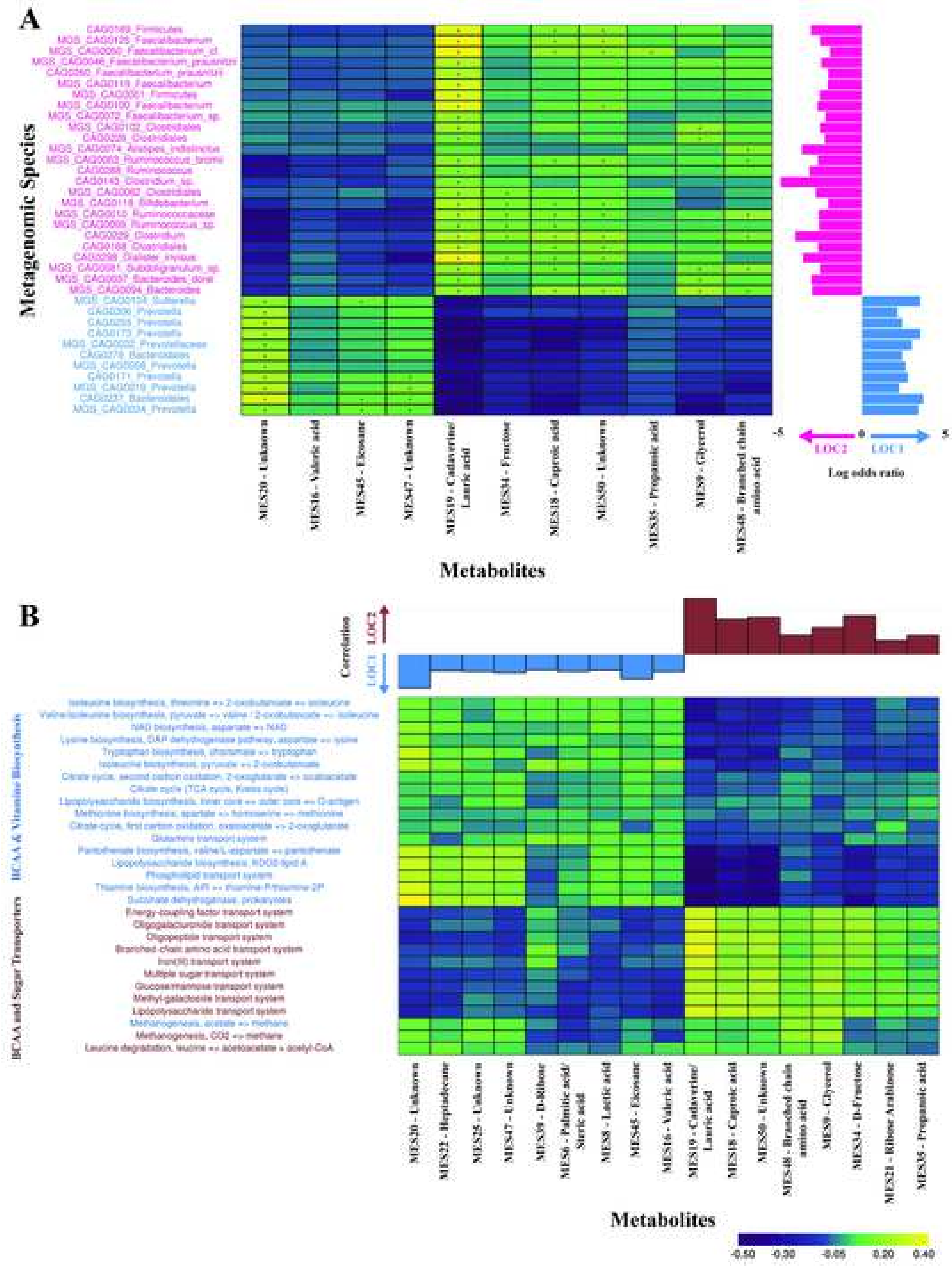

Fig.6

Click here to access/download;Figure;FIGURE 6.jpg

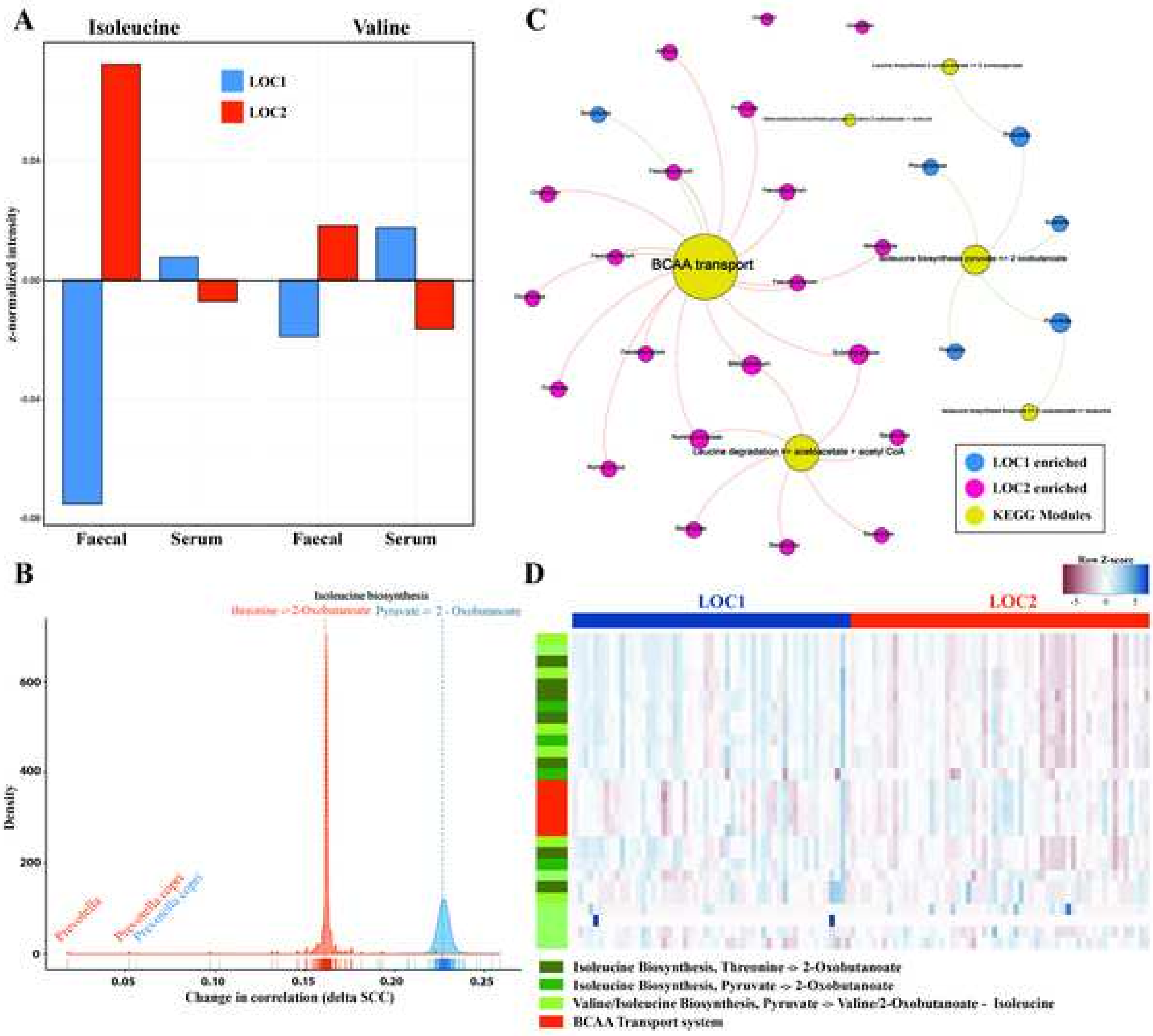

Fig.7

Click here to access/download;Figure;FIGURE 7.TIF

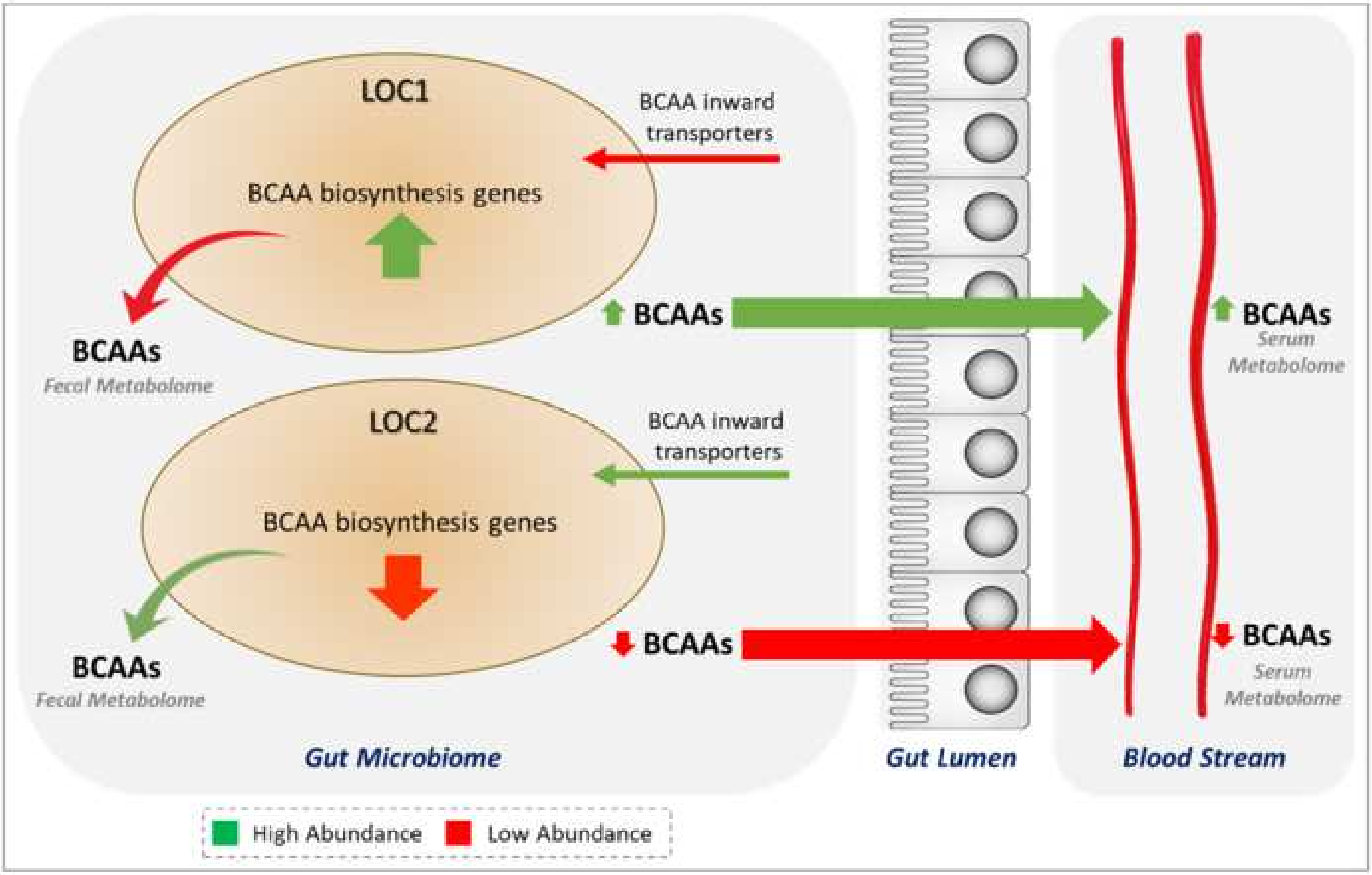

Additional File 1

Click here to access/download
Supplementary Material
Additional_file_1.doc

Additional File 2

Click here to access/download
Supplementary Material
Additional_file_2.xlsx

Additional File 3

Click here to access/download
Supplementary Material
Additional_file_3.xlsx

Additional File 4

Click here to access/download
Supplementary Material
Additional_file_4.xlsx

Additional File 5

Click here to access/download
Supplementary Material
Additional_file5.docx

Additional File 6

Click here to access/download
Supplementary Material
Additional_File_6.xlsx

Additional File 7

Click here to access/download
Supplementary Material
Additional_file_7.xlsx

Additional File 8

Click here to access/download
Supplementary Material
Additional_file_8.xlsx

Additional File 9

Click here to access/download
Supplementary Material
Additional_file_9.xlsx

Additional File 10

Click here to access/download
Supplementary Material
Additional_file_10.xlsx

Additional File 11

Click here to access/download
Supplementary Material
Additional_file_11.xlsx

Additional File 12

Click here to access/download
Supplementary Material
Additional_file_12.xlsx

Additional File 13

Click here to access/download
Supplementary Material
Additional_file_13.xlsx

Additional File 14

Click here to access/download
Supplementary Material
Additional_file_14.xlsx

Additional File 15

Click here to access/download
Supplementary Material
Additional_file_15.xlsx

Additional File 16

Click here to access/download
Supplementary Material
Additional_file_16.xlsx

Additional File 17

Click here to access/download
Supplementary Material
Additional_file_17.xlsx

Additional File 18

Click here to access/download
Supplementary Material
Additional_file_18.xlsx

Supplementary Figure 1

Click here to access/download
Supplementary Material
Supplementary_Figure_1.jpg

Supplementary Table 1

Click here to access/download
Supplementary Material
Supplementary_Table_1.xlsx

Cover Letter
Click here to access/download;Personal Cover;Cover_Letter.docx

To
The Editor
GigaScience

1st November 2018

Dear Editor,

On behalf of the co-authors, I wish to submit the revised manuscript entitled **‘The unique composition of Indian gut microbiome, gene catalogue and associated faecal metabolome revealed using multi-omics approaches’** for your kind consideration for publication in the GigaScience journal.

We sincerely thank both the reviewers and editor for the valuable comments and suggestions that helped in making significant improvements in the manuscript. We have revised the manuscript as per the reviewer’s suggestions and have provided detailed replies to each comment. The revised manuscript text has been marked in Pink and Orange colours to indicate the changes made as per the suggestions of reviewer 1 and 2, respectively. The detailed reply to reviewer’s comments is also provided at the end of this letter. The prominent revisions made in the manuscript are mentioned below.

- The title of the manuscript is revised as per the suggestions of reviewer 2 and editor.
- The raw data has been released for public access at NCBI, and was also uploaded on the GigaScience ftp server.
- ARB-SILVA taxonomy database is now used as reference database for 16S rRNA gene-based taxonomic analysis in place of Greengenes database.
- The gut microbial gene catalogue of Indian cohort has been further improved and the downstream analysis using it has been updated.
- Statistical analysis has been revised with DESeq2-based normalization and Wald test in place of Wilcoxon test. Addition analysis such as ordination of samples from Indian cohort as suggested by reviewer 2, and enterotype analysis using samples from Arumugam et al. 2011, as suggested by reviewer 1 have now been performed and included.
- The manuscript text, figures, tables and supplementary data have been revised as per the reviewer’s suggestions and analysis performed during the revision.
- The discussion has been toned-down and revised at several places as per the suggestions, particularly relating to the impact of microbiome composition on health.

The revised manuscript presents the first large-scale multi-omics data and analysis of the gut microbiome of 110 healthy Indian individuals from two sub-populations (North-central and Southern India) with distinct dietary habits. The study reveals the unique composition of Indian gut microbiome, and provides significant clues on the role of diet in shaping the gut microbiome. The study also established the previously unknown faecal metabolome of the Indian population. The ‘Updated-IGC’ constructed in this study consisting of both Indian and Integrated Gene Catalogues (India+IGC) will act as a valuable resource for the International gut microbiome community. We believe that the manuscript will be of interest to a wide range of readers in the field of gut microbiome research.

We confirm that we have not discussed this work with any board members of *GigaScience* and further affirm that the reported work is original and is not under consideration in any other journal for

publication. I confirm that the authors have no competing interests and all the authors have read and approved the manuscript.

We hope that you will find the revised manuscript suitable for publication in your esteemed journal.

Sincerely,

Dr. Vineet K. Sharma
Associate Professor
Metagenomics and Systems Biology Group,
Department of Biological Sciences,
Indian Institute of Science Education and Research Bhopal, India
Email: vineetks@iiserb.ac.in, vineetks@gmail.com

Replies to Comments -Reviewer 1

The revised manuscript text has been marked in Pink and Orange colours to indicate the changes made as per the suggestions of reviewer 1 and 2, respectively.

Reviewer #1: The study entitled "Multi-omics analysis reveals Indian gut microbiome variations due to diet and location and its implications on human health" describes an in-depth sequencing and metabolomic analysis of a unique set of samples from two distinct locations in India. The authors correlate bacterial species composition and fecal metabolites in order to draw conclusions about health in the two geographic locations and the link with diet and disease risk. Specifically, the North Central, primarily vegetarian population, consumes a high proportion of high-fat and sugary foods and ranks among the lowest for life-expectancy. This is compared to a Southern location with an omnivorous population with a much higher life expectancy and lower risks of T2D and cardiovascular disease.

The correlation and discussion of specific metabolites and risk factors in the North Indian population versus the Southern population, and the conclusions appears to be supported by the data. The authors concentrate on a limited number of major metabolites, BCAAs and SCFAs, and link these to pathways identified in the bacterial species that are present in the populations. This focused approach is quite effective and the subsequent detailed discussion of P. Copri is very relevant (previous association with rheumatoid arthritis). The importance of bacteria-driven metabolism and its association with vegetarian diets are all interesting points where this study of the Indian population brings news perspectives. Indeed the uniqueness of the Indian population, an under-sampled population, is a major contribution to the available databases. It is for this reason that I consider the work appropriate for publication with a certain number of minor revisions prior to publication:

Reply: We thank the reviewer for appreciating our work and providing suggestions which really helped in improving the manuscript. We have tried our best to satisfactorily address the comments and have performed all the suggested analysis. Additionally, we have improved the metagenomic assembly of Indian gut microbiome using IDBA-UD assembler (Kuang et al.; GigaScience; 2017: Please see Methods section). The mean N50 values across all samples showed an increase from 946 bp to 2,288 bp, and the total contig size increased from 1.78 Gbp to 3.086 Gbp (Please see Supplementary Figure 1) in the revised assembly. The updated non-redundant gene catalogue for Indian gut microbiome now consists of 1,551,581 genes. The genes from Indian gene catalogue were added to the Integrated Gene Catalogue (IGC) to construct the 'Updated Integrated Gene Catalogue' (India+IGC), which now consists of 10,823,291 non-redundant genes. We have updated all the corresponding results as per the revised Updated IGC and the suggestions provided by reviewer.

Reference

Kuang et al.; Connections between the human gut microbiome and gestational diabetes mellitus; GigaScience; 2017; doi 10.1093/gigascience/gix058

General comments:

-Subjects were excluded if there was reported use of antibiotics during the previous month. How was this cutoff determined and was any analysis performed on the cohort to determine if there

was any residual effect of antibiotic use (a known issue in India)? This could be as simple as a PCoA plot, using time since last antibiotics exposure as a variable in the 16s diversity analysis.

Reply: We agree with the Reviewer that antibiotic treatment can have residual effects on the gut microbiome and is an important consideration while collecting the samples. A few recent studies have specifically examined these effects, such as the study carried out by Suez et al. demonstrated that a period of 28 days was sufficient for spontaneous recovery of microbiome composition after antibiotic treatment (Please refer Figure 2 of the article [1]). A recent study by Ruixin Liu et al. [2] has also used the same criteria, where the subjects who did not receive any antibiotic treatment for at least one month prior to sample collection were selected (Please refer to Online Methods: 'Faecal sample collection and DNA extraction' section of the cited manuscript). Dethlefsen and Relman [3] show that microbiome communities return to their initial state within one week after the end of antibiotic course. However, we agree that the return of microbiome composition to initial state do vary depending on the type of antibiotic used and can be incomplete. We also agree with the Reviewer's suggestion that a PCoA using time as variable since last antibiotic exposure and estimating its effect would help to identify the effect of treatment on microbiome composition. However, we did not collect this data during the sample collection, and thus could not perform this analysis. Nevertheless, as per the above mentioned studies including the recent ones, we were very careful in recruiting only those volunteers who were not exposed to any antibiotic treatment for over a month.

References

1. Jotham Suez et al; Post-Antibiotic Gut Mucosal Microbiome Reconstitution is Impaired by Probiotics and Improved by Autologous FMT; Cell; 2018; doi:10.1016/j.cell.2018.08.047
2. Ruixin Liu et al; Gut microbiome and serum metabolome alterations in obesity and after weight-loss intervention; Nature Medicine; 2017; doi:10.1038/nm.4358
3. Les Dethlefsen and David Relman; Incomplete recovery and individualised responses of the human distal gut microbiota to repeated antibiotic perturbation; PNAS; 2011; doi:10.1073/pnas.1000087107

-Could the authors please explain their use of Greengenes 13_5? This release dates to 2013. Was SILVA tested?

Reply: We used the Greengenes database because of its wide use in large number of microbiome studies (Yatsunenko et al; Nature; 2011 & Nakayama et al; Sci Rep; 2016) and also in some of our early publications (Maji et al; Environ Microbiol; 2018, Pullikan J et al; Microb Ecol; 2018). We agree with the Reviewer's suggestion of using ARB SILVA database for taxonomic classification of 16S rRNA gene sequences since the Greengenes database has not been updated after May 2013, which justifies the use of more recently updated SILVA database.

As per Reviewer's suggestion, we have now repeated the 16S rRNA gene analysis using ARB SILVA database release 132 (13th December 2017) as reference database for taxonomic annotation. In order to visualize the differences in the results generated from analysis using the two databases, we compared the taxonomies and OTUs generated from the two databases. The Supplementary Table 1 provides details on the percentage of reads assigned at different hierarchical levels using Greengenes and ARB Silva database as reference. There was a marked increase in assignment of OTUs at genus level using ARB SILVA database (95.2%) compared to Greengenes database (54.56%). The increase in the taxonomic annotation was also observed for other population datasets used in the comparison (Supplementary Table 1).

After the reanalysis of 16S rRNA gene data using the annotations from ARB SILVA database, the results have been updated in the revised manuscript in the Results and Figures (please see Figure 1C,

Additional File 5: Figure S3, Figure S5 and Figure S10). We observed similar trends with significant improvements in the annotations of OTUs at the genus level.

References

Tanya Yatsunenko et al; Human gut microbiome viewed across age and geography; Nature; 2012; doi:10.1038/nature11053

Jiro Nakayama; Diversity in the gut bacterial community of school-age children in Asia; Nature Scientific Reports; 2015; doi:10.1038/srep08397

Maji A. et al; Gut microbiome contributes to impairment of immunity in pulmonary tuberculosis patients by alteration of butyrate and propionate producers; Environmental Microbiology; 2018; doi:10.1111/1462-2920.14015

Pullikan J. et al; Gut microbial dysbiosis in Indian children with Autism Spectrum Disorders; Microbial Ecology; 2018; doi:10.1007/s00248-018-1176-2

-I am convinced of the utility of the study, despite some of the additional comments below. Therefore, I would request that the raw shotgun metagenomics data also be made available, and not just the assembled contigs as is currently the case. This is extremely important so that future groups can improve on assemblies and annotations as more data is generated from future studies.

Reply: As per the reviewer’s suggestion, we have now released the raw reads data which can be found at NCBI SRA (https://www.ncbi.nlm.nih.gov/sra) with Project ID: PRJNA397112. The assembled contigs, genes and gene catalogue will also be uploaded on the Giga Science ftp server, which can be accessed by any researcher for the future studies.

Specific comments:

Line 209: "Detection of Enterotypes" The authors use the term 'analysis of enterotypes', referring to Arumugam et al., for the analysis performed in this section and relate the results to those found in the previous study. However the resulting two enterotypes are more accurately, and simply, called clusters, as they are based on two distinct populations in the current study only. This is in contrast to four-country, 22-metagenome analysis performed in Arumugam et al. I would suggest that the terminology be revised. This same type of nomenclature is repeated in line 272: 'metabotype.' I thank that referring to these as clusters is more accurate and more consistent.

It is also present in the discussion (lines 400-401) and methods (699). I would just stress again that two distinct geographical locations which can be statistically separated into two groups, within a single study, does not constitute an enterotype as defined in Arumugam et al. As LOC1 and LOC2 are distinct in this study, factoring this information into clinically relevant models (lines 403-408) does not require a further variable. The analysis and conclusions about the two groups, nevertheless, appear valid.

My suggestion, if the authors wish to use the "enterotype" comparison, would be to explore how this new dataset of 110 individuals fits when combined with that from Arumugam et al. Do the samples still classify into three enterotypes, and what is the distribution across LOC1 and LOC2?

Reply: We agree with the Reviewer’s suggestion that the term ‘enterotype’ should be used when referring to cross national clusters resulting from similarities in microbiome profiles of different populations and their clustering into groups.

We thank the reviewer for the valuable suggestion to compare the Indian samples with that of Arumugam et al., and see if the Indian samples could still be classified into the three enterotypes. Thus, we performed the meta-analysis of 37 samples from the four nations used in Arumugam et al. with our Indian cohort consisting of 110 samples (Please see Figure 3A and Additional File 8). We were able to classify the Indian samples into three enterotypes using genus-level abundance of 110 Indian + 37 samples from four countries (Arumugam et al.). We also identified the distribution of samples from LOC1 and LOC2 in these three enterotypes. We could observe clear differences in representation of samples from India and the other four populations. We could also identify the differences in representation of samples from LOC1 and LOC2 among these enterotypes. We thank the Reviewer for suggesting this analysis, which helped in confirming the previous analysis and results. We have revised the results section '**Line: 246-255**' to include the above analysis and have highlighted in pink. We have also revised the terminology from 'enterotypes' to 'clusters' when referring to the clusters using only Indian datasets in all the sections.

Line 235: 16S Data Analysis

The authors use rarefied reads for downstream analysis. This type of normalization, while useful for calculating UniFrac distances, is no longer accepted as the gold standard for statistical analysis of 16s data. See (McMurdie PJ, Holmes S. Waste not, want not: why rarefying microbiome data is inadmissible. PLoS computational biology. 2014). The authors should explain why they decided to use sub-sampling normalization. How the threshold of 100K was determined?

Reply: We thank the reviewer for this important suggestion on normalizing the 16S rRNA gene counts. Regarding the threshold of 100K, it was a cut-off based on the lowest sequencing depth among all the samples. We agree with the reviewer that the rarefactions method is useful for calculating UniFrac distances, however for comparative analysis it is not the gold standard now, and should be replaced with the methods used in study by McMurdie et al; PLOS Computational biology, as highlighted by the Reviewer. We would like to mention that we did not use rarefaction in any of our statistical analysis or comparisons except for diversity estimations (Alpha and Beta Diversity). For statistical analysis, we used relative abundance of taxa. As per the reviewer's suggestions, we have now revised all the statistical analysis performed using DESeq2 package in R as mentioned in the study (McMurdie et al.) suggested by the Reviewer. The Unifrac analysis has been revised based on OTUs picked using SILVA database (Please see Additional File 5: Figure S11 and Additional File 13).

The differential analysis performed in relation to clinical data and location (lines 247-255) should be reanalyzed using current normalization methods (e.g. DeSeq2 or edgeR packages exist for R).

Reply: We appreciate and agree with the reviewer's suggestions on normalization. Earlier, we had calculated relative abundance by normalizing the raw count of each taxon with total number of reads in each sample. However, as per the reviewer's suggestion we have now re-run all the differential analysis on raw counts at taxonomic level using negative Binomial model based-Wald test in DESeq2. The genera that showed significant difference between Location 1 and Location 2 were plotted (Please see Figure 3B). We also reanalysed the differential species between LOC1 and LOC2 using DESeq2 based normalization on raw abundances of species obtained from mapping of metagenomic reads to the reference genomes (Please see Figure 3C). Further, differential analysis between clusters was also performed using DeSeq2 based normalization on raw counts (Please see Additional File 10). The results and figures have now been updated according to the latest analysis carried out using DESeq2.

Lines 347-352: The addition of 110 individuals is a major contribution. Yet, I think that the authors would agree, any future metagenomics analysis of the intestinal microbiota, even those focusing on South-Asia populations, would best be accomplished using the IGC + this study's additional database. Analysis would not be performed using this study's catalog alone. Please consider rewording here to accurately present the impact of the study.

Reply: We agree with the reviewer's suggestion that IGC+ Indian gene catalogue (constructed in this study), referred to as 'Updated-IGC', would be more useful as a reference database than the Indian gene catalog alone even when studying the South-Asian populations. Thus, we have now also uploaded the 'Updated IGC' at the GigaScience web server. We have also revised the **line 421-424** to include these changes.

Line 561: The authors appear to perform normalization in relation to gene length, probably RPKM. Like 16s analysis, it has been demonstrated that this type of normalization is not the most appropriate for whole genome metagenomics analysis (https://doi.org/10.1186/s12864-016-2386-y). The authors should rerun the analysis to validate that the bacterial species cited in the manuscript remain significant after applying a modern normalization method such as DESeq2 or edgeR. Perhaps other significant species will also be identified.

Reply: We do agree with the Reviewer that the method of normalization can have an impact on the results. As per the reviewer's suggestion, we have now recalculated gene abundance for all the datasets as raw counts instead of normalizing them by gene length, or as proportions. The raw read counts of genes were used for MGWAS analysis and the construction of MGS was performed. The MGS abundance was recalculated, and reanalysed using DESeq2. The P-values obtained were used for further analysis. The differential abundance of MGS between India and other datasets were determined using negative binomial model-based Wald test implemented in DESeq2 for calculating the P-values (Please see Additional File 5: Figure S2, Additional File 6). Moreover, the differential abundance (P-value calculation) of MGS between LOC1 and LOC2 was also determined using DESeq2 based normalization (Please see Additional File 14). Using the raw abundance, we also re-calculated abundance of EggNOG, KEGG Orthologues (KO) and KEGG Modules and performed differential analysis using NB model based Wald test in DESeq2 (Please see Figure 2C, 2D and Additional File 7, Additional File 12, Additional File 17). We have now revised the manuscript at the above mentioned places to include the revised results.

Line 603: The reference cited does not describe the canopy-mgs algorithm. The correct reference is Nature Biotechnology volume 32, pages 822-828 (2014); 'Identification and assembly of genomes and genetic elements in complex metagenomic samples without using reference genomes.' This reference also describes MGS (metagenomic species) that the authors refer to (Line 726, and elsewhere in text).

Reply: We thank the Reviewer for pointing out this error. We have now corrected this reference in the manuscript (**Line: 650**).

Reply to Comments- Reviewer 2

The revised manuscript text has been marked in Pink and Orange colours to indicate the changes made as per the suggestions of reviewer 1 and 2, respectively.

Reviewer #2: # SUMMARY

In this manuscript Dhakan & Maji et al. report on their multi-omic analyses of 110 healthy individuals from two distinct regions in India. The authors obtained 16S rRNA gene (V3 region) amplicon sequencing data, metagenomic sequencing data, and metabolomic data from volunteers' faecal samples. In addition, metabolomic data from serum samples were obtained. Using the metagenomic sequencing data, the existing Integrated Gene Catalog (IGC) was expanded by adding novel, non-redundant genes derived from the India cohort. This represents an important addition to the IGC, thereby further complementing the global, human gut-derived microbial gene catalog. The authors compared the taxonomic composition (amplicon and metagenomic data) and the functional potential (metagenomic data) of Indian-derived gut samples to samples from earlier studies (China, Denmark, USA) and found the Indian microbiome to be largely distinct. The authors conclude that diet is likely to be a strong factor in this, especially since the eating habits are often strongly conserved according to region. Using the metabolomic data, Dhakan & Maji et al. identified differences in the faecal and serum concentrations according to region.

GENERAL COMMENTS

Overall, I think that this study nicely complements existing microbiome studies by further expanding gut microbiome characterization to include samples derived from an Indian population and from different diets (plant-based and omnivorous). Moreover, it highlights the importance of complementary omics, here, metabolomics, in the study of host-microbe interactions.

Reply: We thank the reviewer for appreciating our work and providing suggestions which really helped in improving the manuscript. We have tried our best to satisfactorily address the comments and have performed all the suggested analysis. Additionally, we have improved the metagenomic assembly of Indian gut microbiome using IDBA-UD assembler (Kuang et al.; GigaScience; 2017: Please see Methods section). The mean N50 values across all samples showed an increase from 946 bp to 2,288 bp, and the total contig size increased from 1.78 Gbp to 3.086 Gbp (Please see Supplementary Figure 1) in the revised assembly. The updated non-redundant gene catalogue for Indian gut microbiome now consists of 1,551,581 genes. The genes from Indian gene catalogue were added to the Integrated Gene Catalogue (IGC), to construct the ‘Updated Integrated Gene Catalogue’ (India+IGC) and now consists of 10,823,291 non-redundant genes. We have updated all the corresponding results as per the revised Updated IGC and the suggestions provided by reviewer.

Reference

Kuang et al.; Connections between the human gut microbiome and gestational diabetes mellitus; GigaScience; 2017; doi 10.1093/gigascience/gix058

While many of the authors' conclusions are supported by the reported results, I found that some conclusions need to be toned down as there is not sufficient supporting evidence for these conclusions. Please also see my detailed comments.

Reply: We have made our best efforts to address all the comments and have provided below a point-wise reply to the comments and suggestions. We have also revised the Discussion section at several places to tone down the conclusions correlating the impact of microbiome composition on health as suggested by the reviewer.

The metagenomic sequencing depth in this study is unfortunately not particularly deep, but neither is it shallow. While sequencing depth is always a limiting factor, it is an important factor if the objective is the recovery of novel genetic/genomic information. This needs to be considered when concluding.

Reply: We agree with the reviewer that sequencing depth is a limiting factor in metagenomic studies. In this study, the sequencing depth was not too high (1.5 ± 0.5 Gbp per sample, mean ± standard deviation), compared to the datasets from other microbiome studies (METAHIT: 4.5 Gbp, 100bp reads; Human Microbiome Project: 2.9 Gb, 100bp reads; Qin et al; 2012: 2.61Gbp, 100 bp reads) that were used for comparison with Indian microbiome. However, through a read length of 150bp and a decent paired-end sequencing depth (1.5Gbp) of 110 individuals in this study, we have been able to provide the first insights on the Indian gut microbiome and reveal its unique composition. The increase in sequencing depth certainly would recover more novel genetic information from low abundant microbes which is an important point to consider while making the conclusions. We have now mentioned it in the discussion section and have also considered it while interpreting the results and deriving conclusions (**Line: 408-411, 518-520**).

References

Qin et a; A human gut microbial gene catalogue established by metagenomic sequencing; Nature; 2010; doi 10.1038/nature08821.

The Human Microbiome Project Consortium; Structure, function and diversity of the healthy human microbiome; Nature; 2012; doi 10.1038/nature11234.

Qin et al; A metagenome-wide association study of gut microbiota in type-2 diabetes; Nature; 2012; doi 10.1038/nature11450.

Moreover, I found the variation/spread of the samples from the Indian cohort exceptionally large (Fig. 1 B). This might be something the authors could elaborate on.

Reply: We agree with the reviewer that the spread of the samples from the Indian cohort needs to be discussed in the manuscript. The reason for this variation/spread is the higher inter-sample distances between samples from Indian population compared to other populations (**Additional File 5: Figure S1**). We have now analysed the principal coordinates from PCA in Figure 1B (**Please see Additional File 5; Figure S2**). The Wilcoxon rank sum test of coordinates at PC1 revealed significant difference between LOC1 and LOC2 coordinates. A plausible reason could to be the dietary differences between LOC2 population (non-vegetarian diet) and LOC1 population (plant-based diet), resulting into significant (FDR Adj. P-value = 0.0013) differences observed in their MGS abundance profiles (**Additional File 5: Figure S2**). We have now included this analysis and elaborated it in the results (**Line: 182-188**).

An experiment which I would have liked to see - I am not saying that it is necessary, though - is an ordination of the 110 samples alone, i.e., not contrasting against samples from other studies

but rather within the current study. I would be curious to know if there is substantial separation of samples according to region and/or diet.

Reply: We thank the reviewer for this suggestion and have now performed an ordination of samples based on gene relative abundance table of 110 Indian samples only and observed their separation according to region and diet (**Please see Additional File 5: Figure S13**). We have also performed polyserial correlation to observe the effect of diet and location on separation of samples using gene abundance (**Please see Additional File 13**). The location and diet both were observed to be significantly associated (FDR Adj. P< 0.01) with PC1 explaining the maximum variation in the unsupervised clustering of Indian samples (**Line: 288-292**).

Finally, I would strongly encourage the authors to be more careful with their conclusions on "the gut microbiome and its functional consequences on human health". The present study did not investigate "non-healthy" individuals from the respective regions. It might very well be that the same or very similar observations would have been made with respect to faecal/serum metabolite levels and correlations to respective microorganisms if "non-healthy" individuals were included

Reply: As suggested by the reviewer, we have revised the discussion and conclusion sections, and have carefully rewritten the interpretations and conclusions related to human health. We have also revised the title of the manuscript as suggested in the later comments.

The Data Description section should be extended. It should include description of the metabolomic data that was generated as well as of the metadata which was collected (Age, BMI, etc.). Some of this information is provided in the Methods "Study design and subject enrolment" and should be moved to the Data Description instead.

Reply: As per the suggestion, we have now included the description of the metabolomic data, BMI, age, metadata, study design and subject enrolment in the Data Description section (**Line: 109-132**). Moreover we have now provided a separate table for data collected for different samples in **Additional File 1**.

Instead of reporting "thresholded" p-values (e.g., "P<0.05)"), please report the actual p-values.

Reply: We have replaced the threshold P-values with the actual P-values at most places in the manuscript. However at places such as Line: 317, where multiple species/genes are mentioned we have reported a threshold P-value for considering significant ones.

I would encourage the authors to include the version and parameters of tools that were used in the Methods.

Reply: We have now included the version and parameters of the tools that were used in the Methods section (Please see Methods section).

Moreover, it appears that references are occasionally missing, e.g., for the WMW test, FDR-adjustment, Polyserial correlation/biserial correlations, Reporter features algorithm, etc.

Reply: Thanks for pointing it out. We have now added the references for the statistical tests used for the analysis.

The readability of the manuscript should be further improved, e.g., by involving a professional editing service.

Reply: We have carefully read the manuscript and have made specific efforts to improve the readability. I hope you would find the revised manuscript much improved than the previous version.

My comments below refer to the second row of line numbers, i.e., the one _not_ in typewriter font.

# TITLE

Title: "its implications on human health": It is not clear what the "its" refers to. I would suggest adjusting the title accordingly. Moreover, while it has been shown that diet has an effect on the gut microbiome, I do not know whether "due" is the right wording here. I prefer how the authors phrased it in the abstract, e.g., "showed associations with". I would thus recommend a more careful wording. Moreover, no "non-healthy" individuals were included in the present study, hence making the conclusion of "implications" rather difficult due to lack of supporting evidence (s.a., my general comments)

Reply: We thank the Reviewer for this suggestion. We have revised the title to provide more emphasis on the unique composition of Indian gut microbiome and the functional associations revealed through metabolomics approach. The revised title now reads as "The unique composition of Indian gut microbiome, gene catalogue and associated faecal metabolome deciphered using multi-omics approaches". I hope the reviewers would find it more appropriate than the earlier title.

ABSTRACT

L25: "comprehensively": This could be debated, e.g., at what sequencing depth would one consider to have covered the composition and/or function "comprehensively". Please remove this.

Reply: We have removed the word 'comprehensive' from this line. (**Line: 25**).

L26: "including 16S rRNA marker gene and shotgun metagenomics": This sounds to me as if the "16S rRNA marker gene" sequencing is also considered "metagenomics", which it is not. I would thus suggest "including 16S rRNA gene amplicon sequencing, metagenomic sequencing, and ...".

Reply: We agree with the reviewer and understand that 16S rRNA marker gene sequencing is not metagenomics. While framing the sentence it appeared as one of the methods for metagenomics, and we thank the reviewer for pointing it out. We have now revised it in the manuscript (**Line: 26-27**).

L32: "BCAA": This abbreviation was not introduced before. Same applies to "SCFA in L34". Please adjust accordingly throughout and for all other abbreviations in the manuscript.

Reply: We have now provided the expanded form of all abbreviations at the first instance of their inclusion in the manuscript and have made these changes at all required places (**Line: 33, 36, 37**).

L37: "BCAAs were found higher": "higher" in what? I assume in concentration, but this should be clarified in the text.

Reply: Indeed, we were referring to the BCAA concentration, and we have now revised this sentence (**Line: 38-40**).

L41: "its functional consequences on human health": I think that this is too strong of a claim here. In particular, this study involved only healthy individuals, hence, while there have been differences observed, these differences may not necessarily have a positive or negative effect, but could be neutral. Put differently, different gut microbiomes may be related to healthy individuals or "non-healthy" individuals might have revealed similar findings.

Reply: We agree with the Reviewer and have revised the sentence (**Line: 43-44**).

# MAIN TEXT

L63: "constitution": This typically refers to the "the highest laws of a sovereign state, a federated state, a country or other polity." (https://en.wikipedia.org/wiki/Constitution_(disambiguation)). The authors should consider reformulating this, e.g., by using "condition" or a more appropriate term. Maybe the authors were referring to "composition"? It is not really clear to me, especially with respect to "understanding its variability". It is not just the taxonomic but also the functional composition which has been shown to be of importance. Hence, I would encourage the authors to clarify their point more explicitly here. Finally, this sentence may be misleading as "dysbiosis" is typically used when comparing (at least) one phenotype (e.g., lean) to another (e.g., obese). However, this study is focussed only on one phenotype, i.e., "healthy".

Reply: We agree that the word ‘constitution’ can be replaced with ‘composition’ and have revised this sentence by including all the suggestions made by the reviewer (**Lines: 54-55**).

L69: "WGS": This abbreviation was not properly introduced. Please make sure to do so for all abbreviations throughout the manuscript.

Reply: Thank you for this comment. We have now introduced this abbreviation and all other abbreviations in the manuscript at their first usage (**Line: 59-60**).

L72: "Branch" -> "Branched".

Reply: We have corrected this word (**Line: 62-63**).

L83: I would rephrase "from the major world populations".

Reply: We have rephrased this statement (**Line: 74**).

L86: I would rephrase "equally dominated". Typically, "domination" is used when a single entity has a majority stake.

Reply: We have rephrased this word as ‘equal representation’ (**Line: 77-78**).

L114: I am not sure if these two locations as well as the total cohort size (n = 110) qualify as being

"representative". I would thus suggest to remove the respective wording. Same applies to "comprehensive" , s.a., my respective comment above.

Reply: We agree with the suggestion and have removed the word 'representative' and reframed the sentence. (**Line: 104-105**).

L115: "16S rRNA sequencing" -> "16S rRNA gene sequencing".

Reply: We have made this change (**Line: 105-106**).

L133ff: Was the assembly done on reads from individual samples or on the pooled set of reads? It is not clear as the authors emphasize pooling in the subsequent sentence which reads to me as if this was _not_ done to generate the 1,337,547 contigs. Please clarify.

Reply: We wish to clarify that the assembly was performed on individual samples separately. The reads were mapped back to the assembled contigs from individual samples and the reads that did not map to the contigs from each sample were pooled from all the samples and a denovo cross assembly was performed using the unmapped reads from all the samples. We have employed a similar strategy for contigs and gene catalogue construction as used in other studies [1]. We have now clearly clarified this point in the revised manuscript (**Line: 139-144, 590-592**).

References:

Qin et al; A human gut microbial gene catalogue established by metagenomic sequencing; Nature 2011 (see section Metagenomic sequencing of gut microbiomes).

L139: Please remove "In addition". It sounds as if this is a result from the current paper but it is not.

Reply: We have removed this word and have reframed the sentence. (**Line: 146**).

L141: "populations" seems inappropriate here as the HMP and MetaHIT projects both involved multiple populations themselves.

Reply: We agree with the reviewer and have now changed this word to "multiple populations". (**Line: 147- 148**)

L145 + L146: Please specify what the numbers in the brackets with the "plus-minus" mean. Are they representing the standard deviation?

Reply: As correctly pointed out by the reviewer, the 'plus-minus' represent standard deviation. We have now added standard deviation in the brackets, for example 69.2% (± 4.01% standard deviation). (**Line: 153,155**).

L147f: I am not sure what the authors wanted to say here. Do they mean that reads from _other_ studies were mapped to the original IGC as well as to the updated IGC?

Reply: Here, we had mapped reads from microbiome samples of healthy individuals from three different studies (USA datasets from HMP, Denmark dataset from MetaHIT and Chinese datasets from Qin et al; 2012) on the original IGC and on the updated IGC. We have reframed this statement (**Line:**

158-162) and the mapping is shown in **Fig. 1A**. The results have been updated as per the revised gene catalogue.

L150f: Please rephrase this to reflect that only a _subset_ of the genes of the 110 Indian gut samples in the current study are not represented in other gut microbiome datasets. After all, 718,360 of the 1,479,998 non-redundant genes were added to the original IGC but not the full extent of the current non-redundant genes.

Reply: We thank the Reviewer for this comment. We would like to mention that we aligned the set of non-redundant genes (after removal of redundancy) identified in Indian gut microbiome with the Integrated Gene Catalogue (IGC), and removed the genes sharing ≥90% identity with IGC genes. Thus, the remaining genes from Indian gut microbial gene catalogue which were unique to the IGC (sharing < 90% identity) were added to generate the updated IGC. As per the revised gene catalogue, 943,395 genes from Indian microbiome samples were added to IGC, thus forming an updated IGC containing only the non-redundant genes from Indian cohort. We have now reframed the sentence (**Line: 148-153, 163-164**).

L157: "non-reference" -> "reference-independent".

Reply: We have replaced 'non-reference' with 'reference-independent' (**Line: 171**)

L159: Please remove "higher", it does not seem to fit here.

Reply: We have removed the word 'higher' from the position (**Line: 175**)

L164: "PCA" stands for "Principal Component Analysis", hence, the second "analysis" in the text is redundant.

Reply: We agree with reviewer and have removed the word 'analysis' (**Line: 179-180**)

L166: Actually, if the data was projected to PC1, there would be quite some overlap. The separation is actually benefiting from _both_ dimension, PC1 _and_ PC2. I would suggest removing the "at PC1" altogether.

Reply: We agree with the reviewer and have removed 'at PC1' from this sentence (**Line: 181-182**).

L174: "16S rRNA markers" -> "16S rRNA gene markers".

Reply: We have replaced '16S rRNA markers' with '16S rRNA gene markers' (**Line: 198**).

L175f: While, indeed, the amplicon and, to some extent, the metagenomic data suggest members of the Prevotellaceae to be enriched in the present cohort, referring to this family as a marker should be supported by quantitative analyses, e.g., statistical analysis of differences in group means (t-Test or WMW-test) or a classification-based approach (feature selection).

Reply: We thank the Reviewer for this observation and suggesting the need for a statistical analysis to support it. We have now performed a feature selection test using Random Forest analysis (**Please see Additional File 5: Figure S4**) showing the selection of most important features (mean decrease in accuracy > 0.01; mean relative abundance ≥ 1% in at least one population) and their relative abundance

in different populations. The most discriminating features (families) which were able to classify Indian samples from other populations were plotted rank-wise (**Additional File 5: Figure S5**). The pairwise Wilcoxon rank sum test of important families between India and other populations was performed and represented using box plots (**Please see Additional File 5: Figure S6**). The analysis has been included in revised manuscript (**Line: 199-203**).

L184ff: This paragraph needs to be revised as it currently is hard to read. The sentence in L193f was especially hard to read and I am still unsure about what "The proportion of essential genes covered by top-ranking nine eggNOG clusters" means: What is the meaning of "nine" in this context when the authors refer to 15,000 to 30,000 eggNOG clusters later.

Reply: We apologize for the typo error. We have removed the word "nine" from this statement. We have also revised this paragraph to make it more readable. Please see the changes made in the paragraph (**Line: 215-220**).

L196f: It was not readily clear to me what "alpha diversity (Shannon) calculations using gene abundances" meant and I found the Methods lacking on this point. What gene(s) was/were used ? Moreover, Fig. S4's legend mentions "gene proportions". How does this relate to "gene abundances"? It seems, from the Methods, that rarefaction was used, while the remaining information is scarce on this point. However, this is an important point as the sequencing depth in the current study (mean of 4,545,280 reads/sample) is not particularly deep (cf. Table 1) and, hence, gut microbes' genomes may be covered only partially. In the study by Qin et al . (2010), an order of magnitude more reads per sample ("an average of 62.5 million reads") were produced, albeit at rather short sequencing lengths of 75 bp (compared to 150 bp in the current study).

Reply: We apologise for the lack of clarity in this part. We earlier did not use rarefaction at gene level but the entire gene proportions were used to calculate the diversity. We agree that sequencing depth can have large impact on diversity metrics. We have now used raw gene abundance table which were rarefied at a depth of 1,000,000 seqs/sample for n=30 iterations, and the mean Shannon index were calculated and plotted as box plot (Please see **Additional File 5: Figure S9) (Kuang et al.; GigaScience; 2017)**. We have now included this information in the methods section in revised manuscript (**Line: 228-230, 770-772**).

L202: What does "Eigen values, and their scores" mean, i.e., what is a "score" here? Moreover, they are spelled "eigenvalues", i.e., in one word. Please correct throughout.

Reply: We have now revised the statement and also corrected the term 'eigenvalues' throughout the text as per the suggestions (**Line: 235**).

L203: I am not sure if the authors refer here to "szignificantly" in a statistical sense or not. If so, please include respective quantitive results to support this conclusion.

Reply: As you have rightly mentioned, we were referring to a statistically significant observation, and have now provided the FDR Adjusted P-value in this sentence (**Line 236-237**).

L206: How was the odds-ratio computed? In the Methods, the description refers to LOC1 and LOC2, albeit, it seemed, i.e., I was not sure, that a comparison of Indian microbiome vs. "Other"

microbiome was intended. If this is the case, the authors should clarify this in the Methods, i.e., that not only was LOC1 compared against LOC2 but also "Indian" vs. "Other" (maybe among other pairwise comparisons).

Reply: The Odds Ratio was computed to obtain the enrichment of species/genes between LOC1 and LOC2 as $OR\ (k) = [\sum_{s=LOC1} A_{sk} / \sum_{s=LOC1}(\sum_{i \neq k} A_{si})] / [\sum_{s=LOC2} A_{sk} / \sum_{s=LOC2} (\sum_{i \neq k} A_{si})]$, and also for enrichment in Indian microbiome compared to other datasets consisting of USA, Denmark and China referred as "OTHERS" : $OR\ (k) = ([\sum_{s=INDIA} A_{sk} / \sum_{s=INDIA}(\sum_{i \neq k} A_{si})] / [\sum_{s=OTHERS} A_{sk} / \sum_{s=OTHERS} (\sum_{i \neq k} A_{si})])$.

We have now provided the details of comparison performed in the Methods section (**Line: 809-812**).

L216ff: I welcome the careful wording chosen by the authors here. It appears that there is no detailed dietary information available which could have been used to further support the authors' hypothesis, but they might want to highlight this as a window of opportunity for future study, i.e., including something like a food-frequency questionaire to be able to quantitatively assess possible links to diet.

Reply: We thank the reviewer for this suggestion. This is an important point and we have now included it in the revised manuscript (**Line: 268-270**).

L227: Could the authors please elaborate on how the "Spearman's correlation coefficient" was used in this context? I would have applied Fisher's exact test here.

Reply: As suggested by the Reviewer, we have now used Fisher's exact test here. Earlier, the Spearman's correlations were applied to identify the correlation between KO based and Genus based cluster allocation. Using Fisher's exact test, we found no differences between Genus level and KO level clustering (Fisher's exact P-value = 0.6843) in the samples assignment (**Line: 275**). We have provided the file containing details of cluster allocation for each sample (**Please see Additional File 11)**.

L235: "16S rRNA" -> "16S rRNA gene"

Reply: We have replaced 16S rRNA with 16S rRNA gene at all the places in revised manuscript.

L236: The term "PCA" has been used previously, so this is not the place to introduce the abbreviation.

Reply: We agree and have now removed this term (**Line: 284-285**).

L240: It was not clear to me if "taxonomic and functional diversity" were combined here or not. However, this is important to clarify as taxonomy and function are only partially linked.

Reply: We agree with the Reviewer that taxonomic and functional diversity are only partially linked. We understand that the text could have led to this confusion. We have now revised the text in manuscript and hope that it would read fine now (**Line: 292-293**).

L255: Is this analysis based on amplicon or based on metagenomic sequencing data? L247 indicates the former, while MGS/CAGs are defined based on the latter. Please clarify in the text.

Reply: The results mentioned in line number **300-302** were based on amplicon sequencing data analysis using Phylum abundance, whereas the results in **lines 305-314** are based on taxonomic species identified from metagenomic sequencing data using reads mapped to reference genomes. The results in **line 314-320** are based on the MGS analysis from clustering of gene abundance profiles. We apologize for this confusion. We have now provided this information in the revised manuscript.

L260: Please list "the two species".

Reply: We apologize for the confusion. We were referring to the two species mentioned in the previous line. We have now revised the sentence to clearly refer to the above-mentioned two species (**Line: 320-321**).

L262: Isn't "high fiber-rich" redundant? I.e., either "diet high in fiber" or "fiber-rich diet".

Reply: We agree with the Reviewer and we have now changed this word to fibre-rich diet (**Line: 323**)

L274: The conclusion drawn by the authors about the OPLS-DA results is misleading, s.a., https://www.ncbi.nlm.nih.gov/pmc/articles/PMC4990351/. Specifically, the OPLS-DA model integrates the class information with the aim to _increase_ the between-class separation. Hence, the separation observed in Fig. 4C may (partially) be a consequence of the method used and not of actual separation being in the data. An unsupervised method should be used to check for the presence of meaningful separation followed by a supervised method to perform quantitative evaluation, e.g., PERMANOVA, to check how much of the variance is explained by the respective covariates.

Reply: We agree with the reviewer that OPLS-DA model integrates class information (in this case location) and increases the between class separation. As per the reviewer's suggestion, in addition to OPLS-DA, we have now performed PERMANOVA on metabolite abundance table to assess the effect of covariates and identify the ones which explain maximum variation. We have now included the results of PERMANOVA in the manuscript (Please see **Table 2**). Moreover OPLS-DA models using class information for each of the covariates were used to calculate model Q^2 which assesses the quality of the measurement for each of the covariate (**Please see Table 3**). Since invalid models can still produce higher Q^2 values due to over-fitting, the class labels were randomly permuted for n=200 iterations and distribution of Q2 values were produced to assess the reliability of the Q^2 values. The reliable model should yield significantly higher Q^2 values compared to Q^2 values generated from models with randomly permuted labels (**Please see Additional File 5: Figure S17**). Moreover, an unsupervised clustering of metabolite abundance is already performed (**Please see Figure 4A**), and its polyserial/biserial correlation with different covariates identified PC1 to be correlated with location, and PC2 with the diet (**Line: 340-348**).

L298f: I am not sure if I understood the authors' point right here. "result of its inward transport in microbial cells by the BCAA transporters, thus leading to their accumulation in the colon lumen": Do the authors' mean "uptake by the bacteria, i.e., transport into the microbial cell"? If so, I would not expect an accumulation in the lumen as such.

Reply: We apologize for this confusion. We meant "faecal samples" here and not 'colon lumen'. We have revised this text appropriately in the manuscript (**Line: 364-365**)

L305: Where do the authors show this comparison (serum vs. faeces)? Fig. 6A compares Valine and Isoleucine in LOC1 samples and LOC2 samples, but not serum vs. faeces.

Reply: We have now modified figure 6A showing the comparison of BCAA levels in feaces vs serum (**Please see revised Fig. 6A**)

L328: "the major pathway utilized by this species for BCAA biosynthesis": I am not sure in how much the metagenomic and metabolomic data in this study allow to draw this statement. Metatranscriptomic and metaproteomic data would likely be needed here. I would thus suggest that the authors qualify/nuance this statement.

Reply: We agree with the reviewer. We have revised this text appropriately mentioning the result rather than drawing any conclusion in the manuscript (**Line: 391-395**).

L375ff: The average age of the cohort is rather low (mean of 29.72 years). Age, however, is an important factor for rheumatoid arthritis. Hence, "A probable explanation" could be toned down to "One aspect to this could be ...".

Reply: We thank the Reviewer for this suggestion. We have now revised this statement accordingly (**Line: 446-448**)

L419: "isoluecine" -> "isoleucine".

Reply: We have corrected this word (**Line: 488**).

L439f: The second part of the sentence is redundant with the first part and could be removed, or vice versa.

Reply: We have now removed the redundant part from this sentence (**Line 508-510**).

L459 - 460: "which appears promising in reducing the metabolic risk factors originating through the interactions between diet and gut microbes to maintain a healthy gut flora": This reads misleading as the "diet" was binary, i.e., "vegetarian" vs. omnivorous" and such a statement likely requires for more fine-grained and specialized studies than were performed in this work. Please adjust accordingly.

Reply: We agree with the reviewer. We have now revised this statement and have toned down the general interpretations at various places in the Discussion section (**Line: 512-514**).

L463ff: This entire paragraph reads redundant with the remainder of the Discussion and should thus be removed or substantially shortened.

Reply: We agree with the reviewer. We have now substantially shortened and revised this paragraph in the manuscript (**Line: 515-520**).

L599: "non-reference" -> "reference-independent".

Reply: We have corrected this word (**Line: 647**).

L610: Could the authors please, in analogy to their HMP+NCBI results, report how many of the remaining genes aligned to UNIREF?

Reply: In total, out of 10,839,539 genes present in the Updated gene catalogue, 2,773,591 genes were taxonomically annotated using NCBI + HMP reference genomes at nucleotide level. The remaining 8,049,540 genes were aligned against UNIREF database, and a total of 4,553,299 genes (56.56%) could be assigned with a taxonomic annotation. We have now mentioned this information in Methods section (**Line: 656-660**).

L611f: This sentence should be rephrased.

Reply: We have now rephrased this sentence (**Line: 660-662**)

L706f: How was this assessed and where can the interested reader find the results for this statement?

Reply: We have provided results of CHI index and prediction strength in Additional File 9 with the values. The information about these metrics is provided in Methods section (**Line: 754-759**).

L709ff: It is not clear how the "Between class analysis" was peformed. The authors should provide the respective details, e.g., which test, implementation etc.

Reply: Between Class Analysis was performed to support the clustering and to identify the drivers of these clusters. The between class analysis is a type of principal component analysis with instrumental variables. As in this case, 'Location' is a variable for the separation between LOC1 and LOC2 within India, and "population" for separation between India and other datasets (USA, Denmark and China). It is a supervised projection of data where the distance between predefined classes (example clusters/location) is maximised. We have provided a clear explanation in the manuscript (**Line: 761-767**)

L720: Does "geography" refer to "location" (LOC1 or LOC2) here?

Reply: As correctly pointed out by the reviewer, we meant the two locations (LOC1 and LOC2), and have changed the word 'geography' with 'location' throughout the manuscript (**Line: 775**)

L732: Why was the negative correlation not considered?

Reply: We wish to mention that in this analysis, the objective was to observe the positive association and link them in a network plot. Hence, the negative correlations were not considered. Moreover, plotting negative correlations was not possible in the plot using igraph package in R.

# METHODS

L485: Do you mean the respective table in "Additional_file_1.doc"? Not sure whether this is under the control of the authors, but it should be checked in the proof that the information is consistently named and can be readily found.

Reply: We apologize for this error. We have now changed the name 'Supplementary Table' to 'Additional File 1' in the revised manuscript. We hope that it could now be easily found.

L507: "16S rRNA" -> "16S rRNA gene"

Reply: We have corrected this word at all places in the manuscript.

L534: "phylogenetic distances between reads": Not sure, but did the authors mean "phylogenetic distances between the samples" here?

Reply: The phylogenetic distances were used to calculate Unifrac distances between the samples. The reads used here are the representative sequences from each OTU. Thus, the phylogenetic distances were calculated between each OTU using the representative sequences from OTUs. Using these phylogenetic distances, we calculated Unifrac distances between samples. We have now revised this sentence in manuscript (**Line: 578-580, 772-774**).

L539f: How were host-origin reads identified? Which tool, version, and parameters?

Reply: Human reads were identified and removed from each sample using 18mer matches parameter in Best Match Tagger (BMTagger) version 3.101 (http://casbioinfo.cas.unt.edu/sop/mediawiki/index.php/Bmtagger). We have now mentioned this information in methods section (**Line: 584-586**).

L561ff: This is probably for the formal proofs, but I would strongly encourage to properly format here as it seems that, e.g, "bi" is supposed to read "b subscript i".

Reply: Thanks for bringing it to our notice. We have now formatted the formula (**Line: 610**)

L1037ff: Please check whether "<" and "> are used correctly here." Typically "p < 0.05 is " considered significant and _not_ "P-value>0.05".

Reply: The '>' and '<' are correctly used in Figures 2c, 2d and S3. We used P > 0.05 to show the non-significant dots plotted in 'Red' colour. The significant ones are shown in 'Blue' colour. We have now mentioned it in the figure legend (**Line: 1112-1113**).

# TABLES
I do not know whether the information provided in Table 2 necessitates a separate table. I leave this up to the authors to decide and to potentially discus this with the journal.

Reply: We have now removed this table from the manuscript and included PERMANOVA table as Table 2, which was also suggested by the reviewer in an earlier comment. Also, we have now provided Table 3 showing validation of OPLSDA models for each of covariate by generating a distribution of Q^2 values from random permutation (n=200) of labels and evaluating the number of Q^2 above the model Q^2 for each covariate.

# FIGURES
5: "Logs-Odd Ratio" -> "Log-Odds Ratio"

Reply: Thanks for pointing out this typo. We have corrected it in Figure 5.

S6: The labels on the x-axis and y-axis were not readable. Please adjust accordingly. Moreover,

I am not sure in how much the "clouds" add value here. They are not further discussed in the text and, hence, could be omitted for clarity.

Reply: The font-size of labels has been increased and we hope that it would be easily readable now. The clouds show the density of the unique KOs in the two groups. It has now been mentioned in the legends of this figure. The blue cloud represents the local density estimated from the coordinates of orthologous groups (KO).

# LEGENDS
Throughout: Please verify correct use of "16S rRNA" and "16S rRNA gene".

Reply: We have now changed 16S rRNA to 16S rRNA gene at all places throughout the manuscript.

L1015: "MWAS": Shouldn't this be "MGWAS"?

Reply: Thank you for pointing this type. We have corrected it in the figure legend and also at all places in the manuscript.

L1027: What does "Eigen values and their scores" mean, i.e., what is a "score" here?

Reply: The word 'score' has been removed, and 'Eigen value' have been replaced with 'eigenvalue' at all places in manuscript.

L1092ff: This reads more like a discussion/conclusion and I would thus suggest to remove this from the figure legend.

Reply: The figure legend of Figure 7 has been revised as per the suggestion (**Line: 1162-1164**).
