# Supplementary material for: The unique composition of Indian gut microbiome, gene catalogue, and associated fecal metabolome deciphered using multi-omics approaches
Source: Gigascience. 2019 Jan 30;8(3):giz004. doi: 10.1093/gigascience/giz004 (PMC6394208; doi:10.1093/gigascience/giz004)
Supplement: giga-d-18-00212_revision_2.pdf [file giz004_giga-d-18-00212_revision_2.pdf]

GigaScience

The unique composition of Indian gut microbiome, gene catalogue and associated faecal metabolome deciphered using multi-omics approaches

--Manuscript Draft--

**Manuscript Number:**	GIGA-D-18-00212R2
**Full Title:**	The unique composition of Indian gut microbiome, gene catalogue and associated faecal metabolome deciphered using multi-omics approaches
**Article Type:**	Research
**Funding Information:**	
**Abstract:**	Background<br><br>Metagenomic studies carried out in the past decade have led to an enhanced understanding of the gut microbiome in human health, however, the Indian gut microbiome is still not well explored. We analysed the gut microbiome of 110 healthy individuals from two distinct locations (North-Central and South) in India using multi-omics approaches, including 16S rRNA gene amplicon sequencing, whole genome shotgun metagenomic sequencing, and metabolomic profiling of faecal and serum samples.<br><br>Results<br><br>The gene catalogue established in this study emphasizes the uniqueness of the Indian gut microbiome in comparison to other populations. The gut microbiome of the cohort from North Central India, which was primarily consuming a plant-based diet, was found to be associated with Prevotella, and also showed an enrichment of Branched Chain Amino Acid (BCAA) and lipopolysaccharide (LPS) biosynthesis pathways. In contrast, the gut microbiome of the cohort from Southern India, which was consuming an omnivorous diet, showed associations with Bacteroides, Ruminococcus and Faecalibacterium, and had an enrichment of Short Chain Fatty Acid (SCFA) biosynthesis pathway and BCAA transporters. This corroborated well with the metabolomics results, which showed higher concentration of BCAAs in the serum metabolome of the North-Central cohort and an association with Prevotella. In contrast, the concentration of BCAAs were found higher in the faecal metabolome of the South Indian cohort, and showed a positive correlation with higher abundance of BCAA transporters.<br><br>Conclusions<br><br>The study revealed the unique composition of Indian gut microbiome, established the Indian gut microbial gene catalogue, and also compared it with the gut microbiomes from other populations. The functional associations revealed using metagenomic and metabolomic approaches provide novel insights on the gut-microbe-metabolic axis, which will be useful for future epidemiological and translational researches
**Corresponding Author:**	Vineet Kumar Sharma, Ph.D.<br>Indian Institute of Science Education and Research Bhopal<br>Bhopal, Madhya Pradesh INDIA
**Corresponding Author Secondary Information:**	
**Corresponding Author's Institution:**	Indian Institute of Science Education and Research Bhopal
**Corresponding Author's Secondary Institution:**	
**First Author:**	Darshan B Dhakan
**First Author Secondary Information:**	
**Order of Authors:**	Darshan B Dhakan

Powered by Editorial Manager® and ProduXion Manager® from Aries Systems Corporation

	Abhijit Maji
	Ashok K Sharma
	Rituja Saxena
	Joby Pulikkan
	Tony Grace
	Andres Gomez
	Joy Scaria
	Katherine R Amato
	Vineet Kumar Sharma, Ph.D.
**Order of Authors Secondary Information:**	
**Response to Reviewers:**	--Reviewer #1<br><br>--I commend the authors on their openness and responsiveness to the comments from both reviewers. The additional analyses performed and the clarifications in the manuscript have resulted in a much-improved draft. The availability of both the raw amplicon and WGS data in NCBI's Sequence Read Archive is a great service to the scientific community and should ensure open access to this data and its inclusion in future studies. The origin of the samples and the sequencing data is well-documented and cohort sample collection and storage appears to adhere to ethical and technical standards.<br><br>--More specifically, the updated manuscript addresses and corrects all points that I raised in the first review. These include a major reanalysis of 16S data with an updated database (SILVA December 2017 versus GreenGenes 2013) and an implementation of statistical analysis with normalization performed using DESeq2. They clarify their use of downsized data for Unifrac analysis. Further, the authors now combine their data and run downstream analysis using an "Updated-IGC." This clearly aids their analysis and broadens the appeal of the manuscript as a whole. The 9% of additional genes appears to be unique to the Indian cohort. The authors also performed the suggested enterotype comparisons with the data from Arumugam et al.<br><br>--Based on this new version of the manuscript, my recommendation is the manuscript can be accepted without further scientific revision. The authors should, nevertheless, have a careful review of the text to address remaining grammatical errors and awkward phrasing. A few, non-exhaustive, examples are given:<br><br>The changes suggested by reviewers have been highlighted (orange coloured) in the manuscript.<br><br>Reply: We thank the reviewer for appreciating our efforts and recommending the manuscript for publication. We have carefully read the manuscript for any grammatical errors and have also made all the suggested changes in the manuscript text.<br><br>--Line 114: Change "All the recruited individuals" to "Recruited individuals"<br><br>Reply: As per reviewer’s suggestion, we have made this change (Line: 114).<br><br>--Line 216: Should be "relative abundances"<br><br>Reply: We have corrected these words (Line: 217).<br><br>--Line 365: "its inward transport in microbial cells by the BCAA transporters" would be better as "its uptake by microbes via BCAA transporters"<br><br>Reply: We agree with the rephrasing and have revised this sentence as suggested (Line: 369).<br><br>--Line 408: Change "Though, the sequencing depth in the study was not too high..." to

Powered by Editorial Manager® and ProduXion Manager® from Aries Systems Corporation

"Although sequencing depth was modest….longer paired-ends reads, from the cohort of 110 individuals appears sufficient to provide the first insights on the Indian gut microbiome"

Reply: We have revised the sentence as per the suggestions from both reviewers (Line: 412-415).

--Line 446: "One aspect to this could" could be better written as "One potential explanation could be…"

Reply: We have revised this sentence as per the suggestion (Line: 450)

--Line 486: "has known health benefits..." might be better as "has been reported to be beneficial by preventing…."

Reply: We have revised this sentence as per the reviewer's suggestion (Line: 490).

--Line 505: "are emerging, which results in the increased…." is better as "are emerging, with results showing increased….

Reply: We have revised this sentence as per the reviewer's suggestion (Line: 509).

In addition, we have also carefully checked the manuscript for any grammatical or phrasing errors and hope that the revised manuscript is much better in reading.

Reviewer #2

--The authors have reasonably addressed the comments I raised in the original submission.
Only one general comment and a few minor comments remain, which should all be readily addressable by the authors.

General comments:
--L286-291: It would be good to test whether the location and diet are correlated and to which extent. In fact, given the information from the authors, I would expect them to be correlated. Hence, the observed results (Fig. S12) are to be expected and this should be qualified. If no such test is performed, I would recommend to at least reemphasize the (strong) influence of location on the diet of the studied Indian populations. This is also important with respect to the results in L333-335.

The changes suggested by reviewers have been highlighted (orange coloured) in the manuscript

Reply: We thank the reviewer for the suggestion. We have now performed a correlation of location and diet across all samples and observed a high correlation (ρ= 0.708; FDR Adj. P-value = 2*10-16). We have included these results at both places in the revised manuscript (Line: 293-294, 337-338).

Minor comments:
--Throughout: Frequently, "the"/"a" is missing, e.g., L158 "analysis of microbiome", L159 "reads from other three datasets", L163 "This shows that the addition of subset".

Reply: As suggested by the Reviewer, we have added the/a in the manuscript. We have also carefully checked and corrected the manuscript for any such errors.

--L149-150 - "and unique to IGC": This reads as if the 943,395 genes are unique to the IGC, but aren't his unique to the newly constructed Indian microbial gene catalogue?

Reply: We agree with the reviewer that these 943,395 genes identified from Indian gut microbiome are unique to Indian microbial gene catalogue and not present in IGC. We have rephrased this sentence for clarity (Line 149-150).

Powered by Editorial Manager® and ProduXion Manager® from Aries Systems Corporation

--L161 - "did not show a significant (P< 0.01)": Not sure if the signficance level (alpha = 0.01) is meant here or if the p-value was "< 0.01". In the latter case, it woud be considered significant at alpha = 0.01. Please clarify and verify throughout.

Reply: We apologize for this confusion. We have now provided the exact P-values for HMP, MetaHIT and China datasets, and the P-values were not significant for all the three datasets as found using the student's t-test, whereas it was significant for Indian dataset. The P-values are mentioned at all places in the manuscript where the results were significant (Line 155-156, 161-162, 166).

--L212-214: Species names should be italicized.

Reply: We have made this correction (Line: 213-215).

--L270: "be" is missing -> "needs to be collected".

Reply: We have corrected this sentence (Line: 271).

--L275: The text suggests a "significance", yet the p-value is listed as 0.6841. Please clarify.

Reply: We apologize for this confusion. We have removed the word "significance" and have rephrased the sentence for clarity. Here, we observed a high concordance in allocation of samples to clusters using both taxonomic (genus abundance) and functional (KEGG abundance) information. Using Fisher's exact test as suggested by reviewer 2 during the earlier revision, no significant difference was observed in cluster allocation (P-value =0.6841) thus showing similarity in clustering of samples using taxonomic and functional information. We have also provided this information of cluster allocation in Additional File 11. (Line: 274-277).

--Supplements: Fig S11 still contains a reference to "enterotypes" which, as suggested by Reviewer 1 (and I agree) should be generally avoided, unless in combination with the non-Indian populations. Please check this throughout.

Reply: We agree with the reviewer and we have replaced the word 'enterotypes' with "clusters" in Additional File 5.

--L304-305: This is not a necessity for the revision, but rather a question out of curiosity: Was an association with age tested here, in addition to BMI?

Reply: We had examined the association of multiple covariates including age and BMI, with taxonomic and functional data. We have provided the details of these associations in Additional File 13 and Additional File 15, which were also provided with the earlier submitted manuscript.

--L317 + L319: What do "19 MGS/CAG" and "67 MGS/CAG" refer to here? Are these the numbers of MGSs/CAGs that were annotated to likely be P. copri populations, i.e., multiple strains/sub-species of P. copri were identified? Please clarify this.

Reply: Here, we were referring to the total 19 MGS/CAGs found enriched in LOC1, and 67 MGS/CAGs found enriched in LOC2. We have reframed this sentence for clarity (Line: 317-322).

--L339: Did Cluster-2 show *no* association with location, i.e, was a mixture of samples from LOC1 and LOC2?
Reply: Cluster-2 did show an association with location. Out of a total of 36 samples assigned to Cluster-2 it included 13 samples from LOC-1 and 23 samples from LOC-2. We have mentioned this in the manuscript (Line: 343-344).

--Legend Fig.S17: "OPLD-DA" -> "OPLS-DA "

Reply: We have corrected this word in the legend of Fig. S17 (Additional File 5).

--Fig.S18: Panel A is rather small and the fonts are hard to read. Please increase the

size of the panel.

Reply: We have now increased the font size of Panel A in Figure S18.

--L409-411: I welcome the qualification of the sequencing depth here. Nevertheless, the argument of 2x150bp sequencing is misleading here. Read-length clearly plays a role, so does the overall sequencing depth. While 2x150bp is commonly used currently, and hence the current study is up-to-date, I would suggest the authors to rephrase this slightly. My suggestion would be: "... deviation), the inclusion of 110 individuals from two distinct geographic locations as well as the identification of Indian gut microbiome-specific genes provide a first insight into the Indian gut microbiome and are thus considered important additions to the field."

Reply: We thank the reviewer for this suggestion, and have revised this text as per the suggestion (Line: 412-415).

--L411-413: This sentence reads contradictory in itself. If there is a high diversity, how can (only) two locations be considered representative? I would suggest to rephrase this.

Reply: We have removed the word 'representative' and have rephrased this sentence (Line: 415-417).

--L431: It is not readily clear what "Its" refers to here. I assume it is "Prevotella", yet this should be clarified.

Reply: Yes, the word 'its' was referring to Prevotella. We have reframed this statement for more clarity (Line: 435).

--L439: Please consider removing "driver" unless you can show a causation rather than the association which was presented in the results.

Reply: As per the suggestion, we have removed the word 'driver' from the sentence (Line: 443).

--L442: "bacteria" -> "bacterium"

Reply: We have corrected this word (Line: 446).

--L470-471: The "statistically sound" is not readily clear here. Please consider removing this as I do not find it relevant in this context.

Reply: As per the reviewer's suggestion, we have revised this sentence and have removed the phrase 'statistically sound' (Line: 474).

--L500: "Firmicute" -> "Firmicutes"

Reply: We have corrected this word (Line: 504).

--L515: Please remove "populations", it does not fit in here.

Reply: We have removed this word (Line: 519).

--L578: Please check correct capitalization.

Reply: We thank the reviewer for pointing it out. We have corrected this word to 'UniFrac' and checked it throughout the manuscript (Line: 582).

--L582: Please be consistent in the numbers: "mean = 1.36 Gb" vs. "1.5" (L408).

Reply: Thanks for pointing out this typo, we have corrected this number in line number 412.

--L585: Consider removing "bacterial" unless there was some enrichment step for

Powered by Editorial Manager® and ProduXion Manager® from Aries Systems Corporation

	bacterial DNA.<br><br>Reply: We thank the reviewer for pointing it out. We have removed the word “bacterial” from this sentence (Line: 589).
**Additional Information:**	
**Question**	**Response**
Are you submitting this manuscript to a special series or article collection?	No
**Experimental design and statistics**<br><br>Full details of the experimental design and statistical methods used should be given in the Methods section, as detailed in our Minimum Standards Reporting Checklist. Information essential to interpreting the data presented should be made available in the figure legends.<br><br>Have you included all the information requested in your manuscript?	Yes
**Resources**<br><br>A description of all resources used, including antibodies, cell lines, animals and software tools, with enough information to allow them to be uniquely identified, should be included in the Methods section. Authors are strongly encouraged to cite Research Resource Identifiers (RRIDs) for antibodies, model organisms and tools, where possible.<br><br>Have you included the information requested as detailed in our Minimum Standards Reporting Checklist?	Yes
**Availability of data and materials**<br><br>All datasets and code on which the conclusions of the paper rely must be either included in your submission or deposited in publicly available repositories (where available and ethically appropriate), referencing such data using	Yes

a unique identifier in the references and in the “Availability of Data and Materials” section of your manuscript.<br><br>Have you have met the above requirement as detailed in our Minimum Standards Reporting Checklist?	

Powered by Editorial Manager® and ProduXion Manager® from Aries Systems Corporation

Manuscript
Click here to access/download;Manuscript;IndianGut_GIGA-D-18-00212_10_1_19.docx
Click here to view linked References

Title: The unique composition of Indian gut microbiome, gene catalogue and associated faecal metabolome deciphered using multi-omics approaches

Authors: D.B. Dhakan[1†], A. Maji[1†], A.K. Sharma[1], R. Saxena[1], J. Pulikkan[2], T. Grace[2,3], A. Gomez[4], J. Scaria[5], K.R. Amato[6], V.K. Sharma[1]*

Affiliations: [1]Metagenomics and Systems Biology Laboratory, Department of Biological Sciences, Indian Institute of Science Education and Research Bhopal, India, [2]Department of Genomic Science, Central University of Kerala, India, [3]Division of Biology, Kansas State University USA, [4]Microbiomics Laboratory, Department of Animal Science, University of Minnesota, USA, [5]Animal Disease Research & Diagnostic Laboratory, Veterinary and Biomedical Sciences Department, South Dakota State University, USA, [6]Department of Anthropology, Northwestern University, USA.

Email IDs: darshan@iiserb.ac.in, abhi71084@gmail.com, ashoks773@gmail.com, ritus@iiserb.ac.in, puljobcmi@gmail.com, tonygrace99@gmail.com, gomeza@umn.edu, joy.scaria@sdstate.edu, katherine.amato@northwestern.edu, vineetks@iiserb.ac.in

[†] These authors contributed equally to this work

*Corresponding author

V.K. Sharma: vineetks@iiserb.ac.in

Abstract

Background

Metagenomic studies carried out in the past decade have led to an enhanced understanding of the gut microbiome in human health, however, the Indian gut microbiome is not well explored yet. We analysed the gut microbiome of 110 healthy individuals from two distinct locations (North-Central and Southern) in India using multi-omics approaches, including 16S rRNA gene amplicon sequencing, whole genome shotgun metagenomic sequencing, and metabolomic profiling of faecal and serum samples.

Results

The gene catalogue established in this study emphasizes the uniqueness of the Indian gut microbiome in comparison to other populations. The gut microbiome of the cohort from North-Central India, which was primarily consuming a plant-based diet, was found to be associated with *Prevotella*, and also showed an enrichment of Branched Chain Amino Acid (BCAA) and lipopolysaccharide (LPS) biosynthesis pathways. In contrast, the gut microbiome of the cohort from Southern India, which was consuming an omnivorous diet, showed associations with *Bacteroides*, *Ruminococcus* and *Faecalibacterium*, and had an enrichment of Short Chain Fatty Acid (SCFA) biosynthesis pathway and BCAA transporters. This corroborated well with the metabolomics results, which showed higher concentration of BCAAs in the serum metabolome of the North-Central cohort and an association with *Prevotella*. In contrast, the concentration of BCAAs were found higher in the faecal metabolome of the Southern-India cohort, and showed a positive correlation with the higher abundance of BCAA transporters.

Conclusions

The study reveals the unique composition of Indian gut microbiome, establishes the Indian gut microbial gene catalogue, and compares it with the gut microbiome of other populations. The functional associations revealed using metagenomic and metabolomic approaches provide novel insights on the gut-microbe-metabolic axis, which will be useful for future epidemiological and translational researches.

Keywords: Indian Gut Microbiome, Whole Genome Shotgun, Metagenomics, Metabolomics, Integrated Gene Catalog, Metagenome-Wide Association Study, Core gut microbiome, Short Chain Fatty Acids, Branched Chain Amino Acids

Background

Determining the taxonomic and functional composition of a healthy gut microbiome across different populations is essential for understanding its role in maintaining human health. Several large-scale, world-wide microbiome projects have revealed variability in the gut microbial composition of the healthy individuals due to factors such as mode of delivery, age, geographical location, diet, lifestyle, etc. [1-5]. Majority of the gut microbiome studies have determined microbial taxonomy and functional diversity using 16S rRNA marker gene-based and/or Whole Genome Shotgun (WGS) approaches to understand the functional role of the gut microbiome. However, novel insights on the complex interplay between diet, gut microbes and human health, along with the role of key microbial metabolites, such as Short Chain Fatty Acids and Branched Chain Amino Acids, derived from the microbial fermentation of dietary fibres are beginning to emerge from recent gut metabolomics studies [6, 7]. Moreover, the direct impact of microbial metabolome on human health is also becoming apparent from the recent studies focusing on the 'gut microbiome- host metabolism axis' [8]. Therefore, an integrative approach using both metagenome and metabolome-based characterizations of the gut microbiome appears pragmatic for gaining deeper functional and mechanistic insights into the role of gut microbes on human health.

The large-scale studies carried out so far mainly represent the gut microbiome of urban populations primarily from Europe, US and other 'WEIRD' countries (i.e., the Western, Educated, Industrialized, Rich, and Democratic countries) [9, 10]. Only recently, some studies have characterized the human microbiome from diverse ethnic populations and found significant compositional variations compared to microbiome from other previously studied populations [11-14]. India is the seventh largest country in the world and harbours the second largest population

spread across multiple geographical locations with enormous diversity in ethnicity, lifestyles and dietary habits. India is a home to the majority of world's vegetarian population but also has an almost equal representation of population consuming animal-based diets. The Indian population also has the highest prevalence of diabetes in the world [15]. According to the World Health Organization estimates (WHO, 2011), 53% of deaths in India in the year 2008 were attributed to metabolic conditions such as diabetes and cardiovascular diseases, which are predicted to reach ~75% by 2030 [16].

A few studies have investigated the gut microbiome of the Indian population. A recent study by Maji et al. has shown the functional association of human gut microbiome dysbiosis with tuberculosis through a time-course study carried on six tuberculosis patients in India [17]. However, other gut microbiome studies were mainly limited by small cohort sizes and amplicon-based (16S rRNA gene) sequencing and analysis [17-21]. Thus, several large-scale efforts are needed to identify the Indian population-specific microbiome biomarkers, and to understand the impact of gut microbiome on health and disease in the Indian population along with global comparisons.

However, to uncover the enormous gut microbiome diversity inherent in the different sub-populations of India, extensive sampling and analyses are required. Therefore, as the first large-scale study from India, we selected two prominent locations in North-Central India, i.e. LOC1: Bhopal city, Madhya Pradesh, and Southern India, i.e. LOC2: Kerala. The two locations also had different dietary habits. The Southern-India (LOC2) diet consisted of rice, meat and fish, whereas the North-Central (LOC1) diet consisted of carbohydrate-rich food including plant-derived products, wheat and trans-fat food (high-fat dairy, sweets and fried snacks). The 'Human Development Index Report, UNDP' (United Nations Development Programme), India and SRS-

based life-table (Sample Registration Survey, 2010-14) has revealed that the citizens from Kerala had the highest life-expectancy rates (>74 years) in India, whereas those in Madhya Pradesh (capital city 'Bhopal') exhibited the lowest (<65 years) [22]. Further, a higher predisposition of the North-Indian population towards diabetes, cardiovascular diseases and hypertension is also known, which in contrast is much lower in Southern India, perhaps due to the lifestyle differences in the two regions [15, 23]. Thus, to gain deeper functional insights into the microbiome from these two distinct sub-populations of India, a multi-omics approach was carried out using amplicon-based profiling of taxonomic composition (16S rRNA gene sequencing), whole genome shotgun-based (WGS-based) profiling of metagenome, and GC-MS-based profiling of faecal and serum metabolomic signatures.

Data Description

The two selected locations, Bhopal (LOC1) and Kerala (LOC2) from North-Central and Southern parts of India were about 2,000 kms apart and provided a distinct representation of the Indian population with respect to diet and lifestyle **(Additional File 1)**. The 110 (62 females, 58 males) individuals recruited in this study were not suffering from any disease as reported by personal medical history and physical examination, and confirmed no exposure to antibiotics for at least one month prior to sampling. Recruited individuals had an average BMI of 21.16 (±5.23 standard deviation), an average age of 29.72 (±17.41 standard deviation) and were not diagnosed with any disease at the time of sample collection, and thus were considered as 'healthy' (**Additional File 1**). Moreover, they did not have a second-degree relative history of T2D. The recruitment of volunteers, sample collection, and other study-related procedures were carried out by following the guidelines and protocols approved by the Institute Ethics Committee of Indian Institute of Science Education and Research (IISER), Bhopal, India. The faecal samples were frozen within

30 mins of collection and were then used for 16S rRNA gene V3 hypervariable region amplicon sequencing, WGS-based metagenomic sequencing, and metabolomic analysis. Further, the serum samples collected from a subset of volunteers were used for GC-MS based metabolomics analysis. The sequencing of V3 hypervariable region of 16S rRNA gene and shotgun metagenome sequencing from the 110 faecal samples resulted into 54.87 million paired-end reads (503,460 ± 175,547 (mean ± standard deviation) reads/sample) and 499.98 million paired-end reads (4,545,280 ± 1,498,663 (mean ± standard deviation) reads/sample), respectively (Methods, **Additional File 2** and **Additional File 3)**. The metabolomic analysis was also performed on all faecal and subset of serum samples collected from the same healthy participants using GC-MS, and the resultant CDF files were used for further analysis. The data description of participants and the data generated from each sample is provided in **Additional File 1** under the Metadata information section.

Analyses

Construction of an Indian gut microbial gene catalogue and updated integrated gene catalogue (IGC)

The first step for functional analysis was the construction of an extensive catalogue of gut microbial genes from the Indian population, which was not available previously. A De Bruijn graph-based assembly of reads resulted in 2,165,507 contigs of length ≥500 bp with a total contig size of 3.086 Gbp representing 68.25% of total reads and a mean N50 value of 2,288 bp. To obtain assemblies of low coverage genomic regions or genomes present in the Indian gut microbiome, the reads from each sample were mapped on assembled contigs obtained from their respective sample, and the remaining singletons (unassembled reads) were pooled and re-assembled together

into additional 45,839 contigs with length ≥500 bp and a total assembled length of 34.68 Mbp. A total of 1,551,581 non-redundant genes were predicted from contigs, which represent the gut microbial gene catalogue of the Indian cohorts.

The integrated gene catalogue (IGC) established by Li et al. in a previous multicohort study consisted of 9,879,896 genes identified from 1,267 gut metagenomes representing multiple populations [24]. A total of 943,395 genes (sharing < 90% identity with IGC) out of 1,551,581 from Indian gut microbial gene catalogue were identified as unique to the Indian microbial gene catalogue. The IGC was updated to construct an 'Updated-IGC' by adding these 943,395 non-redundant genes from the Indian gene catalogue. The updated-IGC consisting of 10,823,291 non-redundant genes (an 8.8% increase from IGC) was used as the reference gene catalogue for the subsequent analysis performed in this study. A total of 70.74% (± 3.77% standard deviation) mapping coverage of reads (~7.5% increase in the mapping of reads) was observed from the 110 Indian samples on the updated-IGC as compared to 63% (± 4.61% standard deviation) on IGC, showing a significant (FDR Adj. P-value = 10^{-16}; Student's t-test) increase in mapping of Indian microbial dataset (**Fig. 1A** and **Additional File 4**). The datasets from populations of USA (HMP), Denmark (MetaHIT) and China (a study from Qin et al.) mentioned in **Table 1** were used for a comparative analysis of the microbiome of Indian population with other populations [7, 10, 25]. The mapping of reads from these three datasets (HMP, MetaHIT and China) on updated-IGC (mean mapping coverage: HMP = 67.74%, China = 77.44% and MetaHIT = 75.21%) did not show a significant (P-values: HMP=0.5, MetaHIT=0.85 and China = 0.17; Student's t-test) increment from their mapping coverage on IGC (mean mapping coverage: HMP (USA) = 66.93%, China = 77.37% and MetaHIT (Denmark) = 75.02%) as observed in **Fig. 1A**. This shows that the addition of a subset of non-redundant genes (sharing < 90% identity with IGC) from the Indian gut

microbiome to the IGC significantly increased (FDR Adj. P-value = 10^{-16}; Student's t-test) the mapping percentage of reads from Indian microbiome dataset as compared to the other datasets.

Identification of taxonomic signatures of Indian gut microbiome

To determine the taxonomic and functional composition of the Indian gut microbiome and to identify Indian-specific gut-microbial signatures, a cross-population comparison was carried out using the 16S rRNA gene hypervariable region and shotgun metagenomic data from the other populations. A reference-independent metagenome-wide association study (MGWAS) was carried out to identify the Indian-specific gut metagenomic markers through a comparison with similar large-scale studies from other populations [26]. The genes from the metagenomic samples of four countries (India, China, USA and Denmark) were clustered (see Methods) into 924 clusters based on their co-occurrence and Pearson correlations ($\rho \geq 0.9$) across samples resulting into 335 MGS (metagenomic species) having ≥700 genes in each cluster, and 589 CAGs (co-abundance gene groups) consisting of ≥100 genes in each cluster. Out of the 924 metagenomic clusters, 195 could be assigned up to species level using the taxonomic assignment strategy described in Methods. Canberra distances were calculated from MGS/CAG abundance profiles and their Principal Component Analysis (PCA) was carried out using 'countries' as factors for explaining the variance between samples, which showed that the Indian population formed a distinct cluster from the other populations in PCA (**Fig. 1B**). It is interesting to note that the samples from the Indian cohort were more widely spread owing to the higher inter-sample Canberra distances between Indian samples (mean = 0.689) as compared to other datasets having average inter-sample distances of 0.61, 0.59 and 0.54 for USA, China and Denmark populations, respectively (**Additional File 5: Figure S1**). This could be attributed to the significant (FDR Adj. P-value = 0.00013) differences in MGS

abundance profiles between LOC1 and LOC2 populations as revealed on comparison of their principal coordinates (**Additional File 5: Figure S2**).

Further, the identification of enriched metagenomic species (MGS) from P-values calculated using negative binomial (NB) model-based Wald test (implemented in DESeq2) and Log Odds Ratio showed that the species belonging to the genera *Bacteroides*, *Alistipes*, *Clostridium*, and *Ruminococcus* were depleted in the Indian population (China, Denmark and USA; Log Odds Ratio < -2 and Adj. P-value <0.01), whereas the MGS/CAGs annotated as *Prevotella*, *Mitsuokella*, *Dialister*, *Megasphaera*, and *Lactobacillus* were found to be associated with the Indian population (Adj. P-value < 0.01; Log Odds Ratio > 2), and were the major drivers for separation of Indian samples from other populations (**Additional File 5: Figure S3; Additional File 6**). Furthermore, the distribution of microbial families across ten different populations was also calculated using 16S rRNA gene markers, which revealed Indian gut microbiome to have the highest abundance of Prevotellaceae (**Fig. 1C**). The feature selection method applied using random forest along with pairwise Wilcoxon rank-sum test also identified Prevotellaceae to be significantly higher (FDR Adj. P< 0.05) in gut microbiome of Indian cohort compared to the other population datasets except Indonesia (P-value = 0.506) (**Additional File 5; Figure S4, S5 and S6**) where a comparable abundance of Prevotellaceae was present. The high abundance of Prevotellaceae in Indian population underscores its importance as the marker taxa for the Indian cohort.

Microbial functions enriched in the Indian population

Functional comparison of Indian microbiome with other populations was carried out by mapping the genes derived from assembled contigs to the EggNOG database. In total 69,386 EggNOG functions were identified from the Indian gut microbiome, including 2,328 novel functions obtained from clustering the unmapped genes (see Methods). The core microbial functions that are

essential for microbial survival and present in almost 80% individuals were used for the functional comparison. The core microbiome was derived using a similar strategy as employed in MetaHIT (see Methods) [25]. A set of 1,890 essential genes from six bacterial species namely, *Escherichia coli* MG1655I and MG165II, *Bacteroides thetaiotaomicron* VPI-5482, *Pseudomonas* PA01, *Salmonella enteric* serovar *Typhi* and *Staphylococcus aureus* NCTC 8325 were obtained and were assigned with eggNOG annotations. The eggNOG abundance profile generated from relative abundances of genes observed in Indian and other population dataset were ranked based on their mean abundance in descending order. The range of eggNOGs that included 85% of the 1,890 essential genes were considered as a part of the core microbial eggNOG set for each population dataset and was used for the analysis. Most of the essential genes were included in the top-ranking clusters suggesting that the essential genes are present in higher abundance than the accessory function genes (**Additional File 5: Figure S7**).

The core microbiome of Indian samples was compared with the core microbiome of USA, China and Denmark populations. The proportion of essential genes covered by top-ranking eggNOG clusters showed that 85% of the essential genes could be covered in the least number (15,300) of eggNOGs in the case of Indian population, whereas it was covered by a higher (30,900) number of eggNOGs in the case of USA (20,400), China (19,900) and Denmark populations (**Additional File 5: Figure S8**). These observations suggest that the core functional microbiome of Indian population is less diverse than the other populations. This corroborates well with the alpha diversity (mean Shannon index) calculated using gene abundance tables rarefied at 1,000,000 seqs/sample (for n=30 random iterations), which also showed that the Indian microbiome is significantly (P-value $< 10^{-16}$) less diverse than the microbiome of the other populations analysed in this study (**Additional File 5: Figure S9**).

In total, 5,588 eggNOGs were characterized as core functions commonly present in the core microbiome of all the four population datasets. The co-inertia (Procrustes) analysis and the eigenvalues calculated from PCA using both core and accessory functions also showed that the Indian gut microbiome was significantly (FDR Adj. P-value = 6.4×10^{-10} , 2×10^{-16} and 0.05 with China, Denmark and USA, respectively for PC1) different from the other datasets (**Fig. 2A & B**). These results also show the uniqueness of Indian gut microbial functions in composition and diversity at both core and accessory levels. The Indian gut microbiome was found to be enriched (FDR Adj. P<0.05, Log Odds Ratio >1.5) in functions for carbohydrate and energy metabolism including degradation of complex polysaccharides and glycogen and was also enriched for enzymes from TCA cycle, which corroborates well with the carbohydrate-rich diet of the Indian population (**Fig. 2C and 2D and Additional File 7:** Enriched KO and EggNOG functions).

Unsupervised clustering of Indian samples and their association with previously identified enterotypes

A study by Arumugam et al. classified the samples from multiple populations into clusters based on genus level profiles, and identified three prominent clusters called enterotypes [2]. In order to identify the enterotypes from Indian gut microbiome, a meta-analysis was performed using genus level abundances of samples from the four nations as used by Arumugam et al. along with the Indian cohort. There were three prominent clusters observed with majority (63.6%) of Indian population falling into enterotype-2, which was primarily driven by *Prevotella*. The analysis revealed differences in the distribution of samples from LOC1 and LOC2, where a higher number of samples from LOC1 (73.5%) were associated with enterotype-2 compared to LOC2 (54%). In contrast, LOC2 samples were associated with enterotype-1 (30.3%) and enterotype-3 (16.07%), which were driven by *Bacteroides* and *Ruminococcus*, respectively (**Fig. 3A; Additional File 8**).

An independent microbial abundance-based clustering of Indian samples using Jensen Shannon distances revealed two prominent clusters. The clustering was validated using Calinski Harabasz index (CHI) and prediction strength, which uses a cross-validation approach to validate the robustness of clustering (**Additional File 9**). Cluster 1 was primarily enriched in species from genus *Prevotella* ($P< 10^{-10}$), and Cluster 2 was quite widely spread and was enriched in species belonging to *Bifidobacterium* ($P=10^{-13}$), *Ruminococcus* (P=0.031), *Clostridium* (P=0.04) and *Faecalibacterium* (P= 0.046) (**Additional File 5: Figure S10**, **Additional File 10**). The higher abundance of *Prevotella* in LOC1 and *Bacteroides* in LOC2 in India are perhaps due to the different dietary habits of the two locations. The LOC1 population was mainly consuming a carbohydrate-rich diet comprising of vegetable-based foods and grains, whereas the LOC2 population was consuming a diet consisting of rice, meat and fish. Similar variations in microbiome diversity due to differences in dietary habits have also been observed in earlier studies [27, 28]. However, to confirm the above observations and to assess the quantitative effect of dietary habits on microbial variations, further longitudinal studies are necessary where detailed dietary information needs to be collected through a food-frequency questionnaire.

A similar cluster analysis performed using functional information derived from the abundance of KEGG Orthologs (KO) also showed the clustering of samples into two distinct clusters, namely C1 and C2 (**Additional File 5: Figure S11)**. In comparison to clusters derived from taxonomic information, only 14 out of 110 samples were placed in different clusters using the functional information showing a similarity (P-value = 0.6841; Fisher's exact test; **Additional File 11**) in cluster allocation using both taxonomic and functional information. C1 was found enriched in genes coding for enzymes such as β- glucosidase (LOR = 3.364; P-value = 10^{-20}), and α-fucosidase (LOR = 0.73; $P=10^{-8}$), which are involved in the breakdown of plant-polysaccharides, whereas the

genes coding for enzymes such as lipase (LOR = -1.34; $P=10^{-12}$), carnitine-coA dehydratase (LOR = -1.81; P-value = 0.029) and amino peptidase (LOR = -2.72; $P=10^{-10}$), which are involved in the metabolism of animal-based diet, were enriched in C2 (FDR Adj. P<0.05) (**Additional File 12**).

To identify the covariates explaining the maximum variations in microbial profiles across samples, unweighted UniFrac distances were calculated using phylogenetic distances between OTU reference sequences and OTU table rarefied at 100,000 seqs/sample. The principal component analysis of UniFrac distances and the correlation of loadings for each sample with the covariates using polyserial/biserial correlation identified distinct locations (LOC1 and LOC2) and diet (vegetarian and omnivorous) to be the major covariates explaining the variation in taxonomic diversity between the samples (**Additional File 5: Figure S12, Additional File 13**). An ordination of 110 Indian samples using gene abundance profiles from metagenomic data showed location and diet to be significantly (FDR Adj. P-value < 0.01; Polyserial Correlation) associated with PC1 explaining the maximum variation between samples **(Additional File 5: Figure S13, Additional File 13).** A significant correlation (ρ = 0.708; P-value=$2x10^{-16}$ Spearman's rank correlation) was also observed between location and diet covariates. A comparison of functional diversity using gene abundance curves with increasing number of samples performed between the two locations showed that the microbiome profiles of LOC2 populations were more diverse in their composition compared to LOC1 populations (**Additional File 5: Figure S14**). The inter-individual Bray-curtis distances calculated on normalized gene abundance profiles between LOC1 and LOC2 populations also showed significant differences (FDR Adj. P<0.05), where LOC2 population displayed higher inter-individual heterogeneity in their microbial community structure as compared to LOC1 population **(Additional File5: Figure S15)**.

Major differences in the microbiome profiles were apparent at the Phylum level (using 16S rRNA gene amplicon sequencing) from the higher Bacteroidetes to Firmicutes ratio (P=0.002) in LOC1 (1.93) compared to LOC2 (0.86), which have been previously reported as a result of differences in dietary habits, i.e. vegetarian or plant-based (carbohydrate-rich) vs. omnivore or animal-based (protein-rich) diets (**Additional File 5: Figure S16**) [29, 30]. Notably, these variations were not attributable to BMI (Spearman's Rank correlation, FDR Adj. P=0.78). Taxonomic profiles generated from metagenomic datasets through reads mapped to reference genomes were compared between the two locations at genus and species level using NB model-based Wald test implemented in DESeq2. *Prevotella and Megasphaera* were observed to be higher in LOC1, whereas *Ruminococcus* and *Faecalibacterium* were higher in LOC2 (FDR Adj. P<0.05, Wilcoxon rank-sum test); (**Fig. 3B).** Within these genera, *P. copri*, *P. stercorea* species were significantly higher in LOC1, whereas *F. prausnitzii* and *R. bromii* belonging to genus *Faecalibacterium* and *Ruminococcus*, respectively were higher in LOC2. In addition, *Akkermansia muciniphila*, *Eubacterium siraeum* and *Roseburia hominis* were observed higher in LOC2, and *M. funiformis* and *M. hypermegale* from genus *Megamonas* were higher in LOC1 **(Fig. 3C).** Moreover, the metagenomic species derived from clustering of gene profiles showed that a total of 19 MGS/CAGs were enriched in LOC1 (Log Odds Ratio > 2; Adj. P<0.05), of which 7 MGS/CAGs were annotated to *Prevotella copri*. Similarly, 67 MGS/CAGs were found enriched in LOC2 (Adj. P<0.05; Log Odds Ratio < -2) and included 8 and 3 MGS/CAGs annotated to SCFA producing species *Faecalibacterium prausnitzii* and *Roseburia inulinivorans*, respectively (**Additional File 14**). Interestingly, both, *F. prausnitzii* and *R. inulinivorans*, species enriched in LOC2 are known SCFA producers, and are regarded as commensals with anti-inflammatory properties [31]. In

contrast, *Prevotella*, which was abundant in the LOC1, is known to be associated with fibre-rich diet [32].

Defining the Indian gut metabolome

The analysis of microbial community structure and functions from the two locations having different lifestyle and diet revealed significant insights. Previous studies have shown a direct role of diet in shaping the different gut microbiomes [33]. Thus, to gain deeper insights into the metabolic activity of microbiomes from LOC1 and LOC2 as driven by different diets, faecal metabolites were analysed using a GC-MS-based metabolomics approach. An unsupervised between class analysis of metabolomic profiles separated the samples into three separate clusters, and the robustness was confirmed using prediction strength and Silhouette index (**Fig. 4A and 4B**). Polyserial correlation of covariates showed location to be the major factor explaining the variation at PC1 (FDR Adj. $P<0.01$) separating Cluster 1 from Cluster 2 and 3. In contrast, vegetarian and omnivorous diet groups emerged as other factors explaining the variation at PC2 (FDR Adj. $P<0.01$), and separating Cluster-2 from 3 (**Additional File 15**). The covariates location and diet were also observed to be highly correlated variables showing their strong influence on gut microbiome. Cluster-1 was associated with LOC1 and showed higher concentration of saturated fatty acids including palmitic acid, stearic acid, and valeric acid. Cluster-3 was associated with LOC2 and showed higher abundances of BCAAs, valine, leucine and isoleucine, and SCFAs, propionate and butyrate concentrations. Cluster-2 was enriched in D-glucose, galactose, mannose, lauric acid and cadaverine (a polyamine associated with meat consumption) and was also observed to be associated with LOC2 [34]. To assess the effect of different covariates on the separation of samples, PERMANOVA was performed (**Table 2**). The location was found to explain maximum variation for separation of samples, whereas diet was the second most important variable in

explaining the variance. The OPLS-DA model was used to expose the class separation for each of the covariates using Q^2 values which assesses the quality measurement (**Table 3**). The OPLS-DA models validated by random permutation (n=200) of class labels showed Q^2 values for location and diet to be higher than Q^2 values produced from random permutations with location showing highest Q^2 values (**Additional File 5: Figure S17**). The OPLS-DA model also showed clear separation of samples between locations as class of separation (**Fig. 4C**).

Positive correlation of BCAA transporters with BCAA levels in faecal metabolome

We also identified the marker metabolites, which showed significant (Spearman's correlation, FDR Adj. P<0.05) associations with LOC1 or LOC2. In total, 17 metabolite clusters were identified, of which nine were associated with LOC1, and eight were associated with LOC2 (**Additional File 16**). These marker metabolites showed a positive association with MGS/CAGs. For instance, *Prevotella* annotated clusters correlated significantly with valeric acid and sedoheptulose metabolite markers, which showed a higher relative abundance in LOC1. In contrast, MGS/CAGs belonging to *Faecalibacterium*, *Clostridium*, *Ruminococcus*, and *Alistipes* were positively associated with BCAAs, cadaverine, propanoate and lauric acid in LOC2 (**Fig. 5A**). In addition to the positive association of BCAAs with species enriched in LOC2, a correlation analysis of significantly different (FDR Adj. P<0.05, DESeq2-based Wald test; **Additional File 17**) functional modules revealed that faecal BCAA abundances were positively correlated with BCAA transporter abundance in LOC2. In contrast, BCAA abundance in the faecal metabolome showed a negative correlation (P<0.05) with BCAA biosynthesis pathways (**Fig. 5B**).

The above observations are significant given that BCAAs are important metabolites involved in glucose homeostasis by stimulating insulin secretion [35]. Higher BCAA levels in the faecal samples could be a result of its uptake by microbes via BCAA transporters, leading to their

accumulation in the microbial cells detected in faecal metabolome. This is concordant with higher relative abundance of *Bacteroides vulgatus* and *Eubacterium sireaeum* in LOC2 compared to LOC1, which are known to harbour higher abundance of BCAA transporters (**Fig.3C**) [8]. Further support for this hypothesis emerged from the correlation of circulating BCAA levels (valine and isoleucine) in serum with the corresponding concentrations in faeces. Interestingly, serum BCAA concentrations were significantly higher in LOC1 individuals as compared to LOC2 individuals, which is in contrast with their BCAA levels in the faecal metabolome (**Fig. 6A**). Thus, one possibility is that the accumulation of BCAA in the faeces of individuals of LOC2 was mediated by the inward transport of BCAA by the gut bacteria. In contrast, the lower BCAA accumulation in gut microbes and a higher BCAA biosynthesis by microbial species and its eventual absorption in serum appears to be a plausible reason for the higher BCAA concentrations in serum of LOC1 population.

Role of *Prevotella copri* in the regulation of BCAA levels

To explore the differences in association of functional pathway modules between the two locations, KOs within each module were correlated with KOs from other modules using Spearman's correlation coefficient. The KOs showing significant differences in correlations between LOC1 and LOC2 were identified. This differential correlation analysis of BCAA biosynthetic modules with other pathways in LOC1 and LOC2 revealed that BCAA modules were independently driven in LOC1 and LOC2 (Spearman's rank correlation, FDR Adj. $P<0.01$) (**Additional File 5: Figure S18A & B**). To identify the species and the metabolic pathways that contributed most to the BCAA abundance in faecal and serum metabolome profiles, a correlation analysis with iterations leaving each species out was performed for each metabolic module (**Figure 6B**). The species whose removal leads to a maximum change in the correlation of metabolic

pathway with metabolite was identified, and was considered as an important contributor of that metabolite.

Notably, the BCAA biosynthesis-dependent changes in BCAA levels were largely driven by *Prevotella* species through threonine-dependent and independent biosynthesis pathways as observed from Delta SCC_{bg} values when genes from this species were removed (see Methods). The correlation network analysis with differential MGS/CAGs revealed threonine-independent isoleucine biosynthesis pathway to be highly correlated with *Prevotella copri* in LOC1 (**Fig. 6C**). The first enzyme, D-citramalate synthase, catalysing the first step of threonine-independent isoleucine biosynthesis pathway was also observed as highly enriched (LOR = 1.7) in LOC1 [36]. Further, BCAA biosynthesis pathways was found higher in LOC1, whereas BCAA transporters were higher in LOC2 (**Fig. 6D**) leading to the dynamic changes in BCAA concentrations in faecal and serum metabolome in LOC1 and LOC2 as observed in Fig. 6A.

Discussion

Compositional and functional human gut microbiome studies in different populations have been instrumental in establishing the role of gut microbiome in human health [2, 28, 37, 38]. However, such population-specific signatures and their functional roles are yet unknown for the Indian gut microbiome. This study provides the first insights into the Indian gut microbiome represented through a cohort of 110 individuals from two prominent locations to reveal the taxonomic and functional diversity using 16S rRNA gene, metagenomic analysis, and metabolomic profiling. Although the sequencing depth was modest (1.36 ± 0.5 Gbp per sample, mean ± standard deviation), the inclusion of 110 individuals from two distinct geographic locations as well as the identification of Indian gut microbiome-specific genes provide a first insight into the Indian gut microbiome and are thus considered important additions to the field. The selection of two distinct

sub-populations (Bhopal-LOC1, and Kerala-LOC2) was an important consideration to capture the microbiome variations resulting from different diet and lifestyle of these two cohorts. LOC1 provided a representation of the population from North-Central India mainly consuming a carbohydrate and fat rich diet, whereas LOC2 represented a population from Southern India consuming an omnivorous diet with rice and animal-based products as the primary components.

This study established the gene catalogue of the Indian gut microbiome, which provides the first insights into the yet unknown functional gut microbiome of the Indian population. The genes encoding several transposons, peptidase, glucosidase, and plant polysaccharide degradation enzymes were unique to the Indian population and not represented in other microbiome datasets. The Updated-IGC (IGC+India) constructed by the addition of unique non-redundant genes from the Indian population to the Integrated gene catalogue is likely to act as a reference dataset for gut microbiome studies for global comparative studies, and particularly for studies involving South-Asian populations that have similar dietary habits and lifestyle.

In addition to the basic housekeeping functions of the gut microbiome, which were also found abundant in other datasets, the Indian gut microbiome was enriched in functions for carbohydrate and energy metabolism including degradation of complex polysaccharides, which corroborates well with the typical carbohydrate-rich diet of the Indian population [39]. The distant clustering of Indian samples from other populations revealed the unique composition of the Indian gut microbiota (**Fig. 1B**). *Prevotella* emerged as the most discriminatory genus associated with the Indian population as revealed by both amplicon and MGWAS. The abundance of *Prevotella* was also indicated in the previous 16S rRNA gene-based microbiome studies of the Indian population carried out in small to medium-sized cohorts [18, 19]. Recently, *Prevotella* has been commonly observed in different non-Western communities that consume a plant-rich diet, such as in the

Papua New Guineans, native Africans, rural Malawians, BaAka pygmies, etc [11, 40]. and has also been associated with vegetarianism in the Western populations [41, 42]. However, it has not been observed at such high abundance in the western countries so far. The MGWAS approach in this study showed the presence of *Megasphaera*, *Lactobacillus* and *Mitsuokella* as the other major genera associated with the Indian gut microbiome.

Several recent studies have shown a relationship between the abundance of specific strains of *Prevotella* with inflammatory diseases, since it has a higher intrinsic capacity to stimulate Th17-mediated inflammation, which is generally not expected in a strict commensal bacterium [41, 43, 44]. However, the high abundance of *Prevotella* in the healthy gut microbiome of the Indian population does not corroborate with its potential inflammatory role reported so far. Since this study was only focussed on the gut microbiome of healthy individuals, it is difficult to draw conclusions on the potential inflammatory role of this species. One potential explanation could be the complex set of interactions between host genetic risk factors and environment in which the presence of *Prevotella* may be only one of the factors [45]. Further, strain-level variations are known in the inflammatory responses and not all species of *Prevotella* could be potentially inflammatory, as also evident from the known high genetic diversity within and between the species of *Prevotella* [43]. Thus, the high abundance of *Prevotella* in the healthy microbiota emphasizes the requirement for larger cohort studies in different populations to gain deeper insights into the potential inflammatory roles of gut microbes.

The abundance of *Prevotella* has been associated with plant-based diets, and the typical carbohydrate-rich diet of the Indian population could be one of the reasons for the over-representation of this genus in the Indian gut microbiome [46]. Likewise, the predominance of other microbial species from genus *Lactobacillus*, *Megasphaera* and *Mitsuokella* could be due to

the higher intake of fermented food and dairy products along with the carbohydrate-rich diet in LOC1 [46, 47]. Similarly, *Bacteroides* and *Clostridium*, which were abundant in LOC2, are associated with diets rich in animal-based products, consistent with the omnivorous diet of LOC2 [42]. Interestingly, taxonomy-based clusters 1 and 2 showed associations with the two locations LOC1 and LOC2, and also with the two KO-based clusters (C1 and C2) (**Additional File 5: Figure S10 and S11**). It is to be noted that C1 was enriched in enzymes involved in the degradation of carbohydrate and plant polysaccharides, which correlates well with the carbohydrate-rich diet in LOC1. In contrast, C2 was enriched in enzymes involved in lipid and protein degradation, which relate to the constituents of an omnivorous diet in LOC2. These observations further support the correlation between location, diet and enterotype. Although, the concept of enterotype classification is sometimes criticised due to statistical weakness in some studies, however, a meta-analysis of Indian samples with samples from Arumugam et al. revealed three robust clusters with Indian samples mostly associated with enterotype-2 driven by *Prevotella* [2]. This classification of samples from multiple population/studies into enterotypes has the potential to be clinically relevant in various aspects such as disease diagnosis, early-detection of disease, biomarker development, personalised treatments and xenobiotic metabolism [48]. It is a representation of the major microbial species in the gut microbiome, and thus appears useful for microbiome-based population stratification. A robust statistical analysis with increased sample sizes, direct clinical associations, and detailed molecular interventions are essential for further strengthening its potential.

The study also established the previously unknown faecal metabolome of the Indian population, which showed strong clustering into three metabolomic clusters differentiated by location and diet. The metabolomic clusters also correlated well with the respective dietary habits of the two

locations, where metabolomic Cluster-1 showed an association with LOC1 and was enriched in saturated fatty acids such as palmitic acid and stearic acid, whereas metabolomic Cluster-3 showed an association with LOC2, and was enriched in BCAAs such as isoleucine, valine and leucine, and SCFAs such as propionic acid, and butyric acid. A medium chain fatty acid (MCFA) 'lauric acid' was also found abundant in LOC2 perhaps due to the high dietary consumption of coconut oil in this location [49, 50]. Lauric acid has been reported to be beneficial by preventing fat deposition in blood vessels and acting as an anti-inflammatory and anti-oxidative agent [51].

The major BCAA 'isoleucine' being produced through a less common threonine-independent pathway for isoleucine biosynthesis, and the higher enrichment of the key enzyme, D-citramalate synthase of the above pathway confirmed its higher abundance in LOC1 as compared to LOC2. Further, this pathway was found to be associated with a single species, *Prevotella copri* as reported earlier [8]. Taken together, it appears that the higher abundance of BCAA biosynthesis genes and a lower abundance of BCAA inward transporters in gut microbiome resulted in the lower BCAA accumulation in the fecal metabolome, and higher BCAA concentration in serum as observed in LOC1 (Fig. 7) [8]. However, a contrasting pattern was observed in the case of LOC2, where the lower abundance of BCAA biosynthesis genes and the higher abundance of BCAA inward transporters correlated well with the higher and lower BCAA concentrations in faeces and serum, respectively.

The higher levels of SCFAs in LOC2 could be a consequence of the consumption of omnivorous diet, which is associated with a Firmicutes-rich gut microbiome [52]. SCFAs have well-established roles in human health as an energy source, an anti-inflammatory agent, and for improving intestinal homeostasis by increasing IL-18 production [53]. In contrast, higher serum BCAA levels have well-known roles in promoting insulin resistance and Type-2 Diabetes (T2D), and were found

higher in the serum in LOC1. Several reports on the role of a high-fat diet in the modulation of microbiota and alteration in intestinal barrier are emerging with results showing increased absorption and circulating levels of branched-chain amino acid (BCAA) and reduction of SCFAs such as butyrate, acetate, propionate, and secondary bile acids, as also noted in the case of LOC1 [54, 55]. High-fat and carbohydrate-rich diets have also been associated with an increase in abundance of Bacteroidetes (gram-negative bacteria) leading to a skewed Bacteroidetes: Firmicutes ratio towards the former phylum [32]. Such a ratio was also apparent in this study in LOC1 dominated by *Prevotella* from the phylum Bacteroidetes. Further, a higher serum concentrations of circulating BCAA were also observed in LOC1. These results provide hints on the role of dietary habits in shaping the gut microbiome and its plausible impact on the BCAA and SCFA dynamics observed in these populations.

To conclude, this multi-omics based gut microbiome study of a healthy cohort from two different parts of India provides novel insights into the Indian gut microbiome and metabolome, and reveals the unique gene catalogue from the poorly characterized Indian population. Further studies using higher sequencing depths, and including both healthy and diseased individuals will help in obtaining more comprehensive functional and taxonomic information of gut microbiome from Indian population and its impact on human health.

Methods

Study design and subject enrolment

The study cohort consisted of 110 healthy individuals belonging to different age groups from infants (<1 year) to aged (>50 years), with an average subject age of 29.72 ± 17.4 years (mean ± sd) from two different locations across India i.e., Bhopal (LOC1, n=53) and Kerala (LOC2, n=57),

which are separated by ~1000 miles. LOC1 was located in North-Central India with the majority of population being vegetarian, whereas LOC2 was located in Southern India where the population with dietary habits mostly consisting of rice, seafood and red meat (Diet description section in **Additional File 1**). According to the 'Indian Food Composition Table', the primary Indian diet is rich in carbohydrates such as rice, wheat and potato, and in fat and proteins from milk and dairy products [56]. In addition, several accompaniments to the primary diet also exist including a variety of grains, vegetables, fruits, and usage of oil, spices and animal products.

The faecal samples for metagenomics and blood samples for serum metabolomics were collected from healthy participants and their metadata is provided in **Additional File 1** under the Metadata information section. The recruitment of volunteers, sample collection, and other study-related procedures were carried out by following the guidelines and protocols approved by the Institute Ethics Committee of Indian Institute of Science Education and Research (IISER), Bhopal, India. Each faecal sample was frozen within 30 mins of collection. A written informed consent was obtained from all subjects prior to any study-related procedures, along with information on gender, age, and diet for a period of one month prior to the collection of faecal samples. The recruited individuals did not undergo any medication at least one month prior to the sample collection. All the recruited individuals had an average BMI of 21.16 (±5.23), and were not diagnosed with T2D at the time of sample collection, and did not have a second-degree relative history of T2D. The above samples were then used for 16S rRNA gene V3 hypervariable region amplicon sequencing, shotgun metagenomic sequencing, and metabolomic analysis.

Faecal metagenomic DNA extraction

Metagenomic DNA was isolated from all the faecal samples using QIAamp Stool Mini Kit (Qiagen, CA, USA) according to the manufacturer's instructions. DNA concentration was

estimated by Qubit HS dsDNA assay kit (Invitrogen, CA, USA), and quality was estimated by agarose gel electrophoresis. All the DNA samples were stored at -80 °C until sequencing.

16S rRNA gene amplicon and shotgun metagenome sequencing

The extracted DNA (5ng) was PCR amplified with seven different custom modified 5′-end adaptor-ligated 341F and 534R primers (See the primer details section in Additional File 1) targeting the V3 hypervariable region of 16S rRNA gene. After evaluating the amplified products on 2% w/v agarose gel, the products were purified using Ampure XP kit (Beckman Coulter, Brea, CA USA). Amplicon libraries were prepared by following the Illumina 16S rRNA gene metagenomic library preparation guide. Metagenomic libraries were prepared using Illumina Nextera XT sample preparation kit (Illumina Inc., USA) by following the manufacturer's protocol. Library size of all the libraries was assessed using Agilent 2100 Bioanalyzer (Agilent Technologies, Santa Clara, USA.), and quantified on a Qubit 2.0 fluorometer using Qubit dsDNA HS kit (Life technologies, USA) and by qPCR using KAPA SYBR FAST qPCR Master mix and Illumina standards and primer premix (KAPA Biosystems, Wilmington, MA, USA) following the Illumina suggested protocol. Both the amplicon and metagenomic libraries were loaded on Illumina NextSeq 500 platform using NextSeq 500/550 v2 sequencing reagent kit (Illumina Inc., USA), and 150 bp paired-end sequencing was performed at the Next-Generation Sequencing (NGS) Facility, IISER Bhopal, India.

Amplicon-based taxonomic analysis

A total of 24 Gbps of data were retrieved on de-multiplexing of paired-end reads with an average of 210 Mbp per sample. The paired-end reads were assembled using FLASH and were quality filtered at Q20 (80% bases) Phred quality score using NGSQC Toolkit v 2.3.3 [57, 58]. The primer sequences were trimmed from the High Quality (HQ) reads. The reads were further clustered into

OTUs using closed-reference OTU picking protocol of QIIME at ≥97% identity against ARB SILVA database release 132 (13th December 2017) [59, 60]. The most abundant read was selected as the representative sequence for each OTU and was assigned with taxonomy using the SILVA database. OTU table containing the abundance of each OTU for each sample was generated and used for further analysis. For phylogenetic analysis, representative 16S rRNA genes of phylotypes were aligned against a core set of 16S rRNA gene sequences using align_seqs.py with the PyNAST v.1.2.2 algorithm [61]. The unweighted UniFrac distances between samples were calculated using rarefied OTU abundance (100,000 seqs/sample) table and phylogenetic distances between representative sequences from each OTUs [62].

Pre-processing of the Metagenomic reads

A total of 150 Gbp of metagenomic sequence data (mean = 1.36 Gb) was generated from 110 faecal samples. The metagenomic reads were filtered using NGSQC toolkit v2.3.3 with a cutoff ≥Q20 [57]. The high-quality reads were further filtered to remove the host-origin reads (human contamination) from metagenomic reads using 18mer matches parameter in Best Match Tagger BMTagger v3.101 (BMTagger, RRID:SCR_014619; http://casbioinfo.cas.unt.edu/sop/mediawiki/index.php/Bmtagger), which resulted in the removal of an average of 1% reads. The reads from each sample were assembled separately into contigs using IDBA ud version 1.1.0 [63] with parameters "-mink 31 –maxk 87 –step 5". The reads from each samples were mapped to contigs to estimate read recruitment using FR-HIT version 0.7 [64]. The unmapped reads resulting from each sample were pooled together and denovo assembly was performed on the combined set of singleton (unmapped) reads from all samples. The ORFs from each contig (length ≥ 500bp) were predicted using MetaGeneMark v.3.38 [65]. Pair-wise alignment of genes was performed using BLAT version 2.7.6 [66], and the genes which had an identity ≥ 95% and alignment coverage ≥

90% were clustered into a single set of non-redundant genes, from which the longest gene was selected as the representative ORF to construct the non-redundant gene catalog.

Integrated Gene Catalog (IGC), which represents 1,297 human gut metagenomic samples comprising of HMP, MetaHIT and Chinese datasets, was retrieved [24]. The gene catalogue constructed from Indian samples was combined with the IGC to construct a non-redundant gene catalog (using identity ≥ 95% and alignment coverage ≥ 90%) and is referred to as 'Updated-IGC' in the subsequent analysis.

Quantification of gene content

The quantification of gene content was carried out using the strategy performed by Qin et al., [7] where the high-quality reads were aligned against the updated IGC using SOAP2 in SOAP aligner version 2.21 with an identity cut off ≥ 90% [67]. Two types of alignments were considered for sequence-based profiling:

(1) The entire paired-end read mapped to the gene.
(2) One end of paired-end read mapped to a gene and other end outside genic region.

In both cases, the mapped read was counted as one copy.

The relative abundance of a gene within the sample was calculated as: $a_i = \frac{b_i}{\sum j b_j}$

a_i: relative abundance of gene in sample S; x_i: The times in which gene i was detected in sample S (the number of mapped reads); b_i: copy number of gene i in sequenced data from sample S.

Phylogenetic assignment of reads

A total of 4,097 reference microbial genomes were obtained from Human Microbiome Project (HMP) and National Centre for Biotechnology Information (NCBI) on 5th December 2015 (**Additional File 18**). The databases were independently indexed into two Bowtie indexes using Bowtie-2 version 2.2.9 (Bowtie 2, RRID:SCR_016368) [68]. The metagenomic reads were aligned to the reference microbial genomes using Bowtie-2. The mapped reads from both indexes were merged by selecting the alignment having the higher identity (≥ 90% identity). The percent identity was calculated using the formula: %identity = 100*(matches/total aligned length). The normalized abundance of a microbial genome was calculated by summing the total number of reads aligned to its reference genome. For reads showing hits to both indexed databases with equal identity, each genome was assigned 0.5 read count. The relative abundance of each genome was calculated by adding the normalized abundance of each genome divided by the total abundance. The Calinski Harabasz index (CHI) was used to calculate the variance between the clusters compared to the variance within clusters [2].

Construction of common core microbial functions

To identify the core microbial functions in the gut microbiome of Indian populations and to understand their abundance compared to the other populations, the core microbiome was constructed using a similar strategy as mentioned in MetaHIT [25]. However, to construct a comprehensive core functional microbiome, the information of essential functions from six different microbes including two strains of *Escherichia coli*, *Bacteroides thetaiotaomicron*, *Pseudomonas aeruginosa*, *Salmonella enteric* and *Staphylococcus aureus*, was used instead of considering a single microorganism. The list of essential genes was collected from DEG database v5.0 [69]. 1,890 genes were identified as essential genes in all the six microorganisms. These genes were aligned against eggnog v4.1 database using diamond and were annotated with eggNOG ID

[70, 71]. The core gut microbiome functions were also calculated using the above strategy for the USA, Denmark and Chinese population gut microbial samples to remove the variations arising due to differences in data analysis procedures. Apart from identifying the clusters that represented ≥85% genes within the range of essential gene functions, the low prevalent eggNOG functions, which were present in ≥ 0.0001% abundance in ≥ 80% of samples in that population, were further filtered out. This added filtration step helped in removing all the low abundant functions. To represent the core, the variance of these functions was also calculated between the two Indian locations. The eggNOGs showing significant deviations in variations (P-value≤ 0.05; Levene's test) [72] were further filtered out from the analysis.

Construction of Metagenomic Species for MGWAS

To identify metagenomic markers using a reference-independent approach on metagenomic samples, a metagenome-wide association study was performed for 340 samples (age and gender matched) including India (both locations), USA, China and Denmark populations. The genes present in at least ≥10% of samples were considered and clustered using the canopy-mgs algorithm as described [73]. The genes having Pearson's correlation coefficient (≥0.9) were clustered into CAGs. Furthermore, the genes for which ≥ 90% abundance was obtained from a single sample were discarded.

To determine the taxonomic origin of each MGS/CAG (metagenomic cluster), all the genes were aligned against reference microbial genomes of 4,097 genomes from HMP and NCBI at nucleotide level using BLASTN [74]. The alignment hits were filtered using an E-value $\leq 10^{-6}$ and alignment coverage ≥ 80% of the gene length, and 2,773,591 (25.6%) genes showed alignments against the reference genomes. The remaining 8,049,540 unassigned genes were aligned against UNIREF database (UniRef 50) at protein sequences [75], of which 4,553,299 genes (56.56%) could be

assigned with taxonomic annotations. The sequences that found multiple top hits with equal % sequence identity and scores were further assigned taxonomy based on LCA (Lowest Common Ancestor) method. The genes were finally assigned to taxa based on comprehensive parameters of sequence similarity across phylogenetic ranks as described earlier [76]. The identity threshold of ≥95% was used for assignment up to species level, ≥85% identity threshold for assignment up to genus level, and ≥65% identity was used for phylum level assignment using BLASTN. The taxonomic assignments of MGS/CAGs were performed with the criteria that ≥50% genes in each MGS should map to the same lowest phylogenetic group. Thus, if a particular species is assigned ≥50% genes out of the total genes, the assignment will be carried out at species level rather than at genus or higher orders. The relative abundance of MGS/CAGs in each sample was estimated by using relative abundance values of all genes from that MGS/CAG. A Poisson distribution was fitted to the relative abundance values of the data. The mean estimated from Poisson distribution was assigned as the relative abundance of that MGS. The profile of MGS/CAGs were generated and used for further analysis.

Faecal and Serum metabolomic sample preparation and derivatization

Lyophilized faecal samples were used to achieve better metabolite coverage as described previously [77]. Metabolites were extracted with 1 mL of ice-cold methanol: water (8:2) from 80 mg of lyophilized samples in a bath ultrasonicator (Bioruptor ™ UCD-200, Diagenode, USA) at 4°C for 30 min followed by 2 min of vortexing. The supernatant was extracted by centrifugation at 18,000 g for 15 min at 4°C and dried at 50°C under a gentle stream of nitrogen gas. To remove the residual water molecules from the samples, 100uL of toluene was added to the dry residue and evaporated completely at 50°C under nitrogen gas. Dry extracted metabolites were first derivatized with 50 uL of methoxyamine hydrochloride (MOX) in pyridine (20 mg/mL) at 60°C for 2 hours,

and the second derivatization was performed with 100 uL of MSTFA in 1% TMCS at 60°C for 45 min to form trimethylsilyl (TMS) derivatives. Finally, 150 uL of the TMS derivatives was transferred into a GC glass vial inserts and subjected to GC/TOFMS analysis. Serum samples were prepared (polar metabolites only) and derivatized as described by Psychogium et al., 2011 [78].

Method development and validation

Matrix dilution approach was used for validating the linearity and range of dilution [77]. Pooled faecal samples were used to create the reference peaks to validate the peaks coming from individual samples, which were needed due to the presence of a relatively high abundance of faecal metabolites in the pooled samples. The supernatant of feces after extraction was serially diluted 2, 5, 10, 50, 100, 200 and 500 times with methanol: water (8:2). At dilution 2, the maximum numbers of peaks were seen and were processed with the same dilution factor for all the samples. A total of 30 chemical standards mixture and the pooled faecal samples were used to validate the method. Each stock solution of test standard was carefully prepared in deionized water or with pure ethanol (50,150 350, 500 um) for the determination of linear range, regression coefficient (R2), limit of detection (LOD), and repeatability. L-norvaline (1, 2.5, 5, 10, 20 mg/ml in ethanol) was used as a spiked external standard for the optimized derivatization of the method.

GC-MS analysis

GC-MS was performed on an in-house Agilent 7890A gas chromatograph with 5975C MS system. An HP-5 (25 m × 320 um × 0.25 um i.d.) fused silica capillary column (Agilent J&W Scientific, Folsom, CA) was used with the open split interface. The injector, transfer line and ion source temperatures were maintained at 220, 220 and 250 °C, respectively. Oven temperature was programmed at 70°C for 0.2 min, and increased at 10°C/min to 270°C where it was sustained for 5 min, and further increased at 40°C/min to 310°C where it was held for 11 minutes. The MS was

operated in the electron impact ionization mode at 70eV. Mass data were acquired in full scan mode from m/z 40 to 600 with an acquisition rate of 20 spectra per second. To detect retention time shifts and enable Kovats retention index (RI) calculation, a standard Alkane series mixture (C10–C40) was injected periodically during the sample analysis. RIs are relative retention times normalized to n-alkanes eluted adjacently. For serum samples, we used 2uL aliquot with a split ratio of 4:1 on the same column as described above. The injector port temperature was held at 250°C, and the helium gas flow rate was set to 1mL/min at an initial oven temperature of 50°C. The oven temperature was increased at 10°C/min to 310°C for 11min and mass data were acquired in full scan mode from m/z 40 to 600 with an acquisition rate of 20 spectra per second.

Metabolomic analysis and metabolite profile generation

Raw CDF files were used for peak identification and filtering, and the XCMS package in R were used for pre-processing of the peaks. First, the parameters used for pre-processing of the reads were optimized by calculating the reliability index using the formula given below:

Reliability index = (number of reliable peaks)^2 /number of unreliable peaks.

The reliable peaks were identified for each of the settings such as fwhm, S/N and bw, with a predefined range of values and regression coefficient was calculated for dilutions of QC samples. The number of peaks with a high coefficient of determination ($R^2 \geq 0.9$) were considered reliable, whereas the peaks with very low R^2 (≤ 0.05) were considered unreliable peaks [79]. The finally optimized parameters were: profmethod = bin, method = matched Filter, fwhm =8 and 5 for faecal and serum samples, respectively, and S/N = 12 and 3 for faecal and serum samples, respectively, bw =5 (for first grouping), smooth = linear, family = gaussian, extra = 1, plot type = mdevden, missing =8, bw = 3 (for second grouping). Further, to compare across multiple samples, the peak intensities were normalized (root transformed) and scaled using z-

transformation. These normalized and scaled peak intensities were used for further statistical analysis.

A multivariate statistical method, Orthogonal Projections to Latent Structures Discriminant Analysis (OPLS-DA) [80], was used to identify differences between LOC1 samples (n=53) and LOC2 (n=55) samples. Metabolites driving the differences were identified in metabolic profiles of LOC1 and LOC2 samples using correlations coefficients. The clusters of co-abundant metabolite profiles were identified using R package "WGCNA" [81]. Signed weighted metabolite co-abundance correlation after scaling and centering was calculated across all samples. The soft threshold of β = 15 was chosen for scale-free topology. The dynamic hybrid tree cutting algorithm was used to identify the clusters with a deepsplit = 4 and minimum cluster size = 4. The profile of each faecal metabolite cluster was summarized using eigenvector. The abundance profile of each cluster of metabolites (MES) was calculated using the same methodology as used for MGS cluster abundance profiles.

Retention index (RI) calculation

GC-MS data obtained from the alkene series run was used to calculate the RI for each peak in the samples, and the obtained RI values were further used at the time of library search for the identification of individual metabolite.

$$I = 100\ X\ [n + (logtx - \log tn)/\ (\log tn + 1 - \log tn)$$

Where, tx = retention time of the peak, tn = retention time of preceding alkane, and tn+1 = retention time of the following alkane.

Clustering and enterotype Analysis

Cluster of samples in the dataset were identified from the relative abundance profiles of Genus or Orthologous groups (OG) in the samples. The Jensen-Shannon distances (which estimates the probability distributions between the samples) were calculated and the abundance profiles were clustered using PAM (partitioning around medoids) clustering algorithm as mentioned previously [82]. The optimal number of clusters was assessed using Calinski Harabasz index (CHI) that has shown good performance in recovering the optimal number of clusters [83]. Similarly, the prediction strength from 'fpc' package in R which used cross-validation approach was also employed as another metric for cluster validation. Both the CHI and prediction strength showed quite significantly correlated results. For clustering, CHI and prediction strength gave non-identical values, silhouette index was calculated to estimate the robustness of clusters.

Between class analysis

The between class analysis was performed to identify the drivers and support the clustering of the genus/species/OG abundance profiles into clusters. The between class analysis is a type of principal component analysis with instrumental variables which maximizes the separation between classes of this variable. The instrumental variables here is the cluster classification using PAM clustering and the top species, which contributed the maximum to the principal components obtained from between class analysis were identified as driver species/genus/OG based on their eigenvalues. The analysis was performed using ade4 package in R.

Diversity Analysis

The inter-sample Canberra distances were also calculated using MGS Abundance between populations. The richness of microbiome samples across populations was obtained from Shannon index calculated using raw gene abundance table rarefied at equal depth (1,000,000 seqs/sample) over n=30 random samplings. The beta diversity for 16S rRNA genes (between the samples) was

calculated as unweighted UniFrac distances using OTU tables rarefied at 100,000 seqs/sample and phylogenetic distance between representative sequences from each OTU [84]. The effect of covariates such as age, diet, location (LOC1 and LOC2) and gender were compared for correlation with principal components identified from principal component analysis using UniFrac distances. The polyserial correlations with P-values were calculated for categorical variables and the significance of the covariates for explaining the variation was estimated at each principal component.

Network Analysis

Spearman's rank correlations were computed between each of the species/MGS and the between MGS and functional modules/metabolites. The correlations with significant P-values were selected and were used for the network analysis. The undirected links were generated between correlated nodes (species/KOs/modules) and the strength of the links were given weights based on their correlation coefficients. The network structure was generated using "igraph" package in R. The modularity of the network for KOs association was generated with each module representing the functional modules defined in KEGG database. The negative correlation was not considered in generating the network modules. Moreover, the positive correlations were filtered ($\rho \geq 0.6$) for most of the network analysis.

Supervised learning

Predictive models were built using supervised machine learning algorithm Random Forest (RF)[85]. The models were optimized using 10,000 trees and default settings of mtry (number for variables used to build the model). The mean three-fold cross-validation error rates were calculated for each of the binary tree and the ensemble of trees. The mean decrease in accuracy, which is the increase in error rates on leaving the variable out, was calculated for each prediction and tree and

was used to estimate the importance score. The variables showing a higher mean decrease in accuracy of prediction were considered important for the segregation of the datasets into groups based on the categorical variable.

Statistical Analysis

All the statistical comparisons between groups were performed using Negative Binomial model-based Wald test implemented in DESeq2 and non-parametric Wilcoxon Rank-Sum Test with FDR Adjusted P-Values to control for multiple comparisons [86-88]. The correlations between two variables and the correlations within were calculated using Spearman’s Correlation Coefficient with Adjusted P-Values [89]. The correlations between categorical and numeric variables were performed using Polyserial correlation/biserial correlations [90]. To identify the enrichment of enzymes/species associated with a host, Odds Ratio was used as a measure of the enrichment of a feature in a group. The Odds Ratio was calculated as OR (k) = $[\sum_{s=LOC1} A_{sk} / \sum_{s=LOC1}(\sum_{i\neq k} A_{si})] / [\sum_{s=LOC2} A_{sk} / \sum_{s=LOC2} (\sum_{i\neq k} A_{si})]$ for enrichment of genes/species between two locations, where A_{sk} denotes abundance of species/gene k in sample S. Also the enrichment of species/genes between Indian microbiome compared to other datasets consisting of USA, Denmark and China referred as “OTHERS” were computed as OR(k) $([\sum_{s=INDIA} A_{sk} / \sum_{s=INDIA}(\sum_{i\neq k} A_{si})] / [\sum_{s=OTHERS} A_{sk} / \sum_{s=OTHERS} (\sum_{i\neq k} A_{si})])$. All the graphs and plots were generated using the ggplot2 package in R.

Correlation analysis between functional modules and metabolite clusters

To calculate the association of microbial functional modules with faecal metabolite clusters, the Spearman's correlation coefficients were calculated to rank KOs for association with metabolite clusters and Metabotypes. To quantify the shift in Spearman correlation between given KEGG module and the metabolite cluster compared to the background distribution, the background adjusted median Spearman's correlation was calculated for a given KEGG module m as:

$$SCC_{bg.adj} = \text{median}\ (SCC_{KOs\ \epsilon\ KEGG\ Module\ m}) - \text{median}\ (SCC_{KOs\ KEGG\ Module\ m})$$

Where SCC_{KO} is the partial Spearman's correlation coefficient between KO and the metabolite cluster.

Identification of microbial species driving the association between KEGG Module and metabolite abundance was done by iterating the correlation between KO belonging to the KEGG module and the metabolite after excluding the genes annotated to that KO from each species. The change in median Spearman’s correlation coefficient between the KOs and the metabolite, when genes from that species are excluded from the analysis, was calculated as described previously [8]. The species showing the maximum change in the overall correlation of module with metabotype was plotted.

List of abbreviations

Indian Gut Microbiome (IGM), Enterotypes (ET), Integrated Gene Catalog (IGC), Metagenome-Wide Association Study (MGWAS), Short Chain Fatty Acids (SCFAs), Branched Chain Amino Acids (BCAAs).

Declarations

Collection of Datasets for Comparative analysis

The 74 HMP metagenomes were collected from http://hmpdacc.org/HMASM or NCBI SRA (accession SRR059347). The 85 Danish fecal metagenomes from METAHIT were obtained from European Nucleotide Archive (http://www.ebi.ac.uk/ena, study accession number ERP000108). The 71 Chinese metagenome samples were obtained from NCBI SRA (accession number – SRR341581).

Ethics approval and consent to participate

The recruitment of volunteers, sample collection, and other study-related procedures were carried out by following the guidelines and protocols approved by the Institute Ethics Committee of Indian Institute of Science Education and Research (IISER), Bhopal, India. Written informed consent was obtained from all subjects prior to any study-related procedures.

Consent for publication

Not applicable

Availability of data

The datasets generated and/or analysed during the current study have been deposited in the National Centre for Biotechnology Information (NCBI) BioProject database under the project number PRJNA397112. Metabolomics data are available via the MetaboLights database (accession number MTBLS803). Supporting data are also available via the *GigaScience* repository, GigaDB [91].

Competing interests

The authors declare that they have no competing interests.

Funding

This work was supported by the intramural funding received from IISER Bhopal, Madhya Pradesh, India.

Author's contributions

VKS and AM conceived the work and participated in the design of the study. AM and JP collected all the samples in collaboration with TG. AM designed the study protocols and performed sample processing, DNA extraction, metabolite extraction and profiling from faecal and blood samples. RS and AM carried out the library preparation and sequencing work. DBD carried out all

metagenomic data and statistical analysis. AKS and DBD analyzed the metabolomics data. AM and DBD did the primary data interpretation of analytical outcomes under the supervision of VKS. AM, DBD, AKS, RS, AG, JS, KRA and VKS drafted the manuscript. All authors read and approved the final manuscript.

Acknowledgments

The sequencing and computational analysis were performed at the NGS Facility and HPC and computing facility, respectively, at IISER Bhopal. DBD, AM, RS and JP received fellowships from the UGC (University Grants Commission), Centre for Research on Environment and Sustainable Technologies (CREST, IISER Bhopal), DST-INSPIRE and Central University of Kerala, respectively.

References:

1. Turnbaugh PJ, Ley RE, Hamady M, Fraser-Liggett CM, Knight R and Gordon JI. The human microbiome project. Nature. 2007;449 7164:804-10.
2. Arumugam M, Raes J, Pelletier E, Le Paslier D, Yamada T, Mende DR, et al. Enterotypes of the human gut microbiome. Nature. 2011;473 7346:174-80.
3. Yatsunenko T, Rey FE, Manary MJ, Trehan I, Dominguez-Bello MG, Contreras M, et al. Human gut microbiome viewed across age and geography. Nature. 2012;486 7402:222-7.
4. Dominguez-Bello MG, Costello EK, Contreras M, Magris M, Hidalgo G, Fierer N, et al. Delivery mode shapes the acquisition and structure of the initial microbiota across multiple body habitats in newborns. Proc Natl Acad Sci U S A. 2010;107 26:11971-5.
5. Saxena R and Sharma V. A metagenomic insight into the human microbiome: Its implications in health and disease. Medical and Health Genomics. Elsevier; 2015. p. 107-19.
6. Schwiertz A, Taras D, Schafer K, Beijer S, Bos NA, Donus C, et al. Microbiota and SCFA in lean and overweight healthy subjects. Obesity (Silver Spring). 2010;18 1:190-5.
7. Qin J, Li Y, Cai Z, Li S, Zhu J, Zhang F, et al. A metagenome-wide association study of gut microbiota in type 2 diabetes. Nature. 2012;490 7418:55-60.
8. Pedersen HK, Gudmundsdottir V, Nielsen HB, Hyotylainen T, Nielsen T, Jensen BA, et al. Human gut microbes impact host serum metabolome and insulin sensitivity. Nature. 2016;535 7612:376-81.
9. Gupta VK, Paul S and Dutta C. Geography, Ethnicity or Subsistence-Specific Variations in Human Microbiome Composition and Diversity. Front Microbiol. 2017;8:1162.
10. Human Microbiome Project C. Structure, function and diversity of the healthy human microbiome. Nature. 2012;486 7402:207-14.
11. Gomez A, Petrzelkova KJ, Burns MB, Yeoman CJ, Amato KR, Vlckova K, et al. Gut Microbiome of Coexisting BaAka Pygmies and Bantu Reflects Gradients of Traditional Subsistence Patterns. Cell Rep. 2016;14 9:2142-53.
12. Obregon-Tito AJ, Tito RY, Metcalf J, Sankaranarayanan K, Clemente JC, Ursell LK, et al. Subsistence strategies in traditional societies distinguish gut microbiomes. Nat Commun. 2015;6:6505.
13. Schnorr SL, Candela M, Rampelli S, Centanni M, Consolandi C, Basaglia G, et al. Gut microbiome of the Hadza hunter-gatherers. Nat Commun. 2014;5:3654.
14. Hasler R, Kautz C, Rehman A, Podschun R, Gassling V, Brzoska P, et al. The antibiotic resistome and microbiota landscape of refugees from Syria, Iraq and Afghanistan in Germany. Microbiome. 2018;6 1:37.
15. Mohan V, Sandeep S, Deepa R, Shah B and Varghese C. Epidemiology of type 2 diabetes: Indian scenario. Indian J Med Res. 2007;125 3:217-30.
16. Mushtaq MU, Gull S, Abdullah HM, Shahid U, Shad MA and Akram J. Waist circumference, waist-hip ratio and waist-height ratio percentiles and central obesity among Pakistani children aged five to twelve years. BMC Pediatr. 2011;11:105.
17. Maji A, Misra R, Dhakan DB, Gupta V, Mahato NK, Saxena R, et al. Gut microbiome contributes to impairment of immunity in pulmonary tuberculosis patients by alteration of butyrate and propionate producers. Environ Microbiol. 2018;20 1:402-19.
18. Pulikkan J, Maji A, Dhakan DB, Saxena R, Mohan B, Anto MM, et al. Gut Microbial Dysbiosis in Indian Children with Autism Spectrum Disorders. Microb Ecol. 2018;76 4:1102-14.
19. Bhute S, Pande P, Shetty SA, Shelar R, Mane S, Kumbhare SV, et al. Molecular Characterization and Meta-Analysis of Gut Microbial Communities Illustrate Enrichment of Prevotella and Megasphaera in Indian Subjects. Front Microbiol. 2016;7:660.

20. Shetty SA, Marathe NP and Shouche YS. Opportunities and challenges for gut microbiome studies in the Indian population. Microbiome. 2013;1 1:24.
21. Tandon D, Haque MM, R S, Shaikh S, P S, Dubey AK, et al. A snapshot of gut microbiota of an adult urban population from Western region of India. PLoS One. 2018;13 4:e0195643.
22. Suryanarayana M, Agrawal A and Prabhu KS. Inequality-adjusted human development index for India's states. United Nations Development Programme (UNDP) India. http://www. undp. org/content/dam/india/docs/inequality_adjusted_human_development_index_for_indias_state 1. pdf [NS], 2011.
23. Misra A, Pandey RM, Devi JR, Sharma R, Vikram NK and Khanna N. High prevalence of diabetes, obesity and dyslipidaemia in urban slum population in northern India. Int J Obes Relat Metab Disord. 2001;25 11:1722-9.
24. Li J, Jia H, Cai X, Zhong H, Feng Q, Sunagawa S, et al. An integrated catalog of reference genes in the human gut microbiome. Nat Biotechnol. 2014;32 8:834-41.
25. Qin J, Li R, Raes J, Arumugam M, Burgdorf KS, Manichanh C, et al. A human gut microbial gene catalogue established by metagenomic sequencing. Nature. 2010;464 7285:59-65.
26. Wang J and Jia H. Metagenome-wide association studies: fine-mining the microbiome. Nat Rev Microbiol. 2016;14 8:508-22.
27. Tyakht AV, Kostryukova ES, Popenko AS, Belenikin MS, Pavlenko AV, Larin AK, et al. Human gut microbiota community structures in urban and rural populations in Russia. Nat Commun. 2013;4:2469.
28. Liang C, Tseng HC, Chen HM, Wang WC, Chiu CM, Chang JY, et al. Diversity and enterotype in gut bacterial community of adults in Taiwan. BMC Genomics. 2017;18 Suppl 1:932.
29. Aleksandrowicz L, Tak M, Green R, Kinra S and Haines A. Comparison of food consumption in Indian adults between national and sub-national dietary data sources. Br J Nutr. 2017;117 7:1013-9.
30. Joy EJ, Green R, Agrawal S, Aleksandrowicz L, Bowen L, Kinra S, et al. Dietary patterns and non-communicable disease risk in Indian adults: secondary analysis of Indian Migration Study data. Public Health Nutr. 2017;20 11:1963-72.
31. Rios-Covian D, Ruas-Madiedo P, Margolles A, Gueimonde M, de Los Reyes-Gavilan CG and Salazar N. Intestinal Short Chain Fatty Acids and their Link with Diet and Human Health. Front Microbiol. 2016;7:185.
32. Kovatcheva-Datchary P, Nilsson A, Akrami R, Lee YS, De Vadder F, Arora T, et al. Dietary Fiber-Induced Improvement in Glucose Metabolism Is Associated with Increased Abundance of Prevotella. Cell Metab. 2015;22 6:971-82.
33. De Filippo C, Cavalieri D, Di Paola M, Ramazzotti M, Poullet JB, Massart S, et al. Impact of diet in shaping gut microbiota revealed by a comparative study in children from Europe and rural Africa. Proc Natl Acad Sci U S A. 2010;107 33:14691-6.
34. Ruiz-Capillas C and Jimenez-Colmenero F. Biogenic amines in meat and meat products. Crit Rev Food Sci Nutr. 2004;44 7-8:489-99.
35. Layman DK. The role of leucine in weight loss diets and glucose homeostasis. J Nutr. 2003;133 1:261S-7S.
36. Drevland RM, Waheed A and Graham DE. Enzymology and evolution of the pyruvate pathway to 2-oxobutyrate in Methanocaldococcus jannaschii. J Bacteriol. 2007;189 12:4391-400.
37. Wu GD, Chen J, Hoffmann C, Bittinger K, Chen YY, Keilbaugh SA, et al. Linking long-term dietary patterns with gut microbial enterotypes. Science. 2011;334 6052:105-8.
38. Cho I and Blaser MJ. The human microbiome: at the interface of health and disease. Nat Rev Genet. 2012;13 4:260-70.

39. Green R, Milner J, Joy EJ, Agrawal S and Dangour AD. Dietary patterns in India: a systematic review. Br J Nutr. 2016;116 1:142-8.
40. Martinez I, Stegen JC, Maldonado-Gomez MX, Eren AM, Siba PM, Greenhill AR, et al. The gut microbiota of rural papua new guineans: composition, diversity patterns, and ecological processes. Cell Rep. 2015;11 4:527-38.
41. Ley RE. Gut microbiota in 2015: Prevotella in the gut: choose carefully. Nat Rev Gastroenterol Hepatol. 2016;13 2:69-70.
42. Losasso C, Eckert EM, Mastrorilli E, Villiger J, Mancin M, Patuzzi I, et al. Assessing the Influence of Vegan, Vegetarian and Omnivore Oriented Westernized Dietary Styles on Human Gut Microbiota: A Cross Sectional Study. Front Microbiol. 2018;9:317.
43. Larsen JM. The immune response to Prevotella bacteria in chronic inflammatory disease. Immunology. 2017;151 4:363-74.
44. Scher JU, Sczesnak A, Longman RS, Segata N, Ubeda C, Bielski C, et al. Expansion of intestinal Prevotella copri correlates with enhanced susceptibility to arthritis. Elife. 2013;2:e01202.
45. Renz H, von Mutius E, Brandtzaeg P, Cookson WO, Autenrieth IB and Haller D. Gene-environment interactions in chronic inflammatory disease. Nat Immunol. 2011;12 4:273-7.
46. Tremaroli V and Backhed F. Functional interactions between the gut microbiota and host metabolism. Nature. 2012;489 7415:242-9.
47. Selhub EM, Logan AC and Bested AC. Fermented foods, microbiota, and mental health: ancient practice meets nutritional psychiatry. J Physiol Anthropol. 2014;33:2.
48. Costea PI, Hildebrand F, Arumugam M, Backhed F, Blaser MJ, Bushman FD, et al. Enterotypes in the landscape of gut microbial community composition. Nat Microbiol. 2018;3 1:8-16.
49. Jaarin K, Norliana M, Kamisah Y, Nursyafiza M and Qodriyah HMSJECC. Potential role of virgin coconut oil in reducing cardiovascular risk factors. 2014;20 8:3399-410.
50. Boemeke L, Marcadenti A, Busnello FM, Gottschall CBAJOJoE and Diseases M. Effects of coconut oil on human health. 2015;5 07:84.
51. Intahphuak S, Khonsung P and Panthong AJPb. Anti-inflammatory, analgesic, and antipyretic activities of virgin coconut oil. 2010;48 2:151-7.
52. Nicholson JK, Holmes E, Kinross J, Burcelin R, Gibson G, Jia W, et al. Host-gut microbiota metabolic interactions. Science. 2012;336 6086:1262-7.
53. Boulange CL, Neves AL, Chilloux J, Nicholson JK and Dumas ME. Impact of the gut microbiota on inflammation, obesity, and metabolic disease. Genome Med. 2016;8 1:42.
54. Neis EP, Dejong CH and Rensen SS. The role of microbial amino acid metabolism in host metabolism. Nutrients. 2015;7 4:2930-46.
55. Li X, Shimizu Y and Kimura I. Gut microbial metabolite short-chain fatty acids and obesity. Biosci Microbiota Food Health. 2017;36 4:135-40.
56. Longvah T, Anantan I, Bhaskarachary K and Venkaiah K. Indian food composition tables. National Institute of Nutrition, Indian Council of Medical Research; 2017.
57. Patel RK and Jain M. NGS QC Toolkit: a toolkit for quality control of next generation sequencing data. PloS one. 2012;7 2:e30619.
58. Magoc T and Salzberg SL. FLASH: fast length adjustment of short reads to improve genome assemblies. Bioinformatics. 2011;27 21:2957-63.
59. Quast C, Pruesse E, Yilmaz P, Gerken J, Schweer T, Yarza P, et al. The SILVA ribosomal RNA gene database project: improved data processing and web-based tools. Nucleic Acids Res. 2013;41 Database issue:D590-6.
60. Kuczynski J, Stombaugh J, Walters WA, Gonzalez A, Caporaso JG and Knight R. Using QIIME to analyze 16S rRNA gene sequences from microbial communities. Curr Protoc Bioinformatics. 2011;Chapter 10:Unit 10 7.

61. Caporaso JG, Bittinger K, Bushman FD, DeSantis TZ, Andersen GL and Knight R. PyNAST: a flexible tool for aligning sequences to a template alignment. Bioinformatics. 2010;26 2:266-7.
62. Lozupone C, Lladser ME, Knights D, Stombaugh J and Knight R. UniFrac: an effective distance metric for microbial community comparison. ISME J. 2011;5 2:169-72.
63. Peng Y, Leung HC, Yiu SM and Chin FY. IDBA-UD: a de novo assembler for single-cell and metagenomic sequencing data with highly uneven depth. Bioinformatics. 2012;28 11:1420-8.
64. Niu B, Zhu Z, Fu L, Wu S and Li W. FR-HIT, a very fast program to recruit metagenomic reads to homologous reference genomes. Bioinformatics. 2011;27 12:1704-5.
65. Zhu W, Lomsadze A and Borodovsky M. Ab initio gene identification in metagenomic sequences. Nucleic acids research. 2010;38 12:e132-e.
66. Kent WJ. BLAT--the BLAST-like alignment tool. Genome Res. 2002;12 4:656-64.
67. Li R, Yu C, Li Y, Lam T-W, Yiu S-M, Kristiansen K, et al. SOAP2: an improved ultrafast tool for short read alignment. Bioinformatics. 2009;25 15:1966-7.
68. Langmead B and Salzberg SL. Fast gapped-read alignment with Bowtie 2. Nature methods. 2012;9 4:357.
69. Zhang R and Lin Y. DEG 5.0, a database of essential genes in both prokaryotes and eukaryotes. Nucleic acids research. 2008;37 suppl_1:D455-D8.
70. Buchfink B, Xie C and Huson DH. Fast and sensitive protein alignment using DIAMOND. Nature methods. 2014;12 1:59.
71. Powell S, Forslund K, Szklarczyk D, Trachana K, Roth A, Huerta-Cepas J, et al. eggNOG v4. 0: nested orthology inference across 3686 organisms. Nucleic acids research. 2014;42 D1:D231-D9.
72. Lim T-S and Loh W-Y. A comparison of tests of equality of variances. Computational Statistics & Data Analysis. 1996;22 3:287-301.
73. Nielsen HB, Almeida M, Juncker AS, Rasmussen S, Li J, Sunagawa S, et al. Identification and assembly of genomes and genetic elements in complex metagenomic samples without using reference genomes. Nat Biotechnol. 2014;32 8:822-8.
74. Altschul SF, Gish W, Miller W, Myers EW and Lipman DJ. Basic local alignment search tool. Journal of molecular biology. 1990;215 3:403-10.
75. Suzek BE, Huang H, McGarvey P, Mazumder R and Wu CH. UniRef: comprehensive and non-redundant UniProt reference clusters. Bioinformatics. 2007;23 10:1282-8.
76. Huson DH, Auch AF, Qi J and Schuster SC. MEGAN analysis of metagenomic data. Genome research. 2007;17 3:377-86.
77. Phua LC, Koh PK, Cheah PY, Ho HK and Chan ECY. Global gas chromatography/time-of-flight mass spectrometry (GC/TOFMS)-based metabonomic profiling of lyophilized human feces. Journal of Chromatography B. 2013;937:103-13.
78. Psychogios N, Hau DD, Peng J, Guo AC, Mandal R, Bouatra S, et al. The human serum metabolome. PloS one. 2011;6 2:e16957.
79. Gao X, Pujos-Guillot E, Martin J-F, Galan P, Juste C, Jia W, et al. Metabolite analysis of human fecal water by gas chromatography/mass spectrometry with ethyl chloroformate derivatization. Analytical biochemistry. 2009;393 2:163-75.
80. Worley B and Powers R. Multivariate Analysis in Metabolomics. Curr Metabolomics. 2013;1 1:92-107.
81. Langfelder P and Horvath S. WGCNA: an R package for weighted correlation network analysis. BMC Bioinformatics. 2008;9:559.
82. Kaufman L and Rousseeuw PJ. Partitioning around medoids (program pam). Finding groups in data: an introduction to cluster analysis. 1990:68-125.

83. Liao M, Li Y, Kianifard F, Obi E and Arcona S. Cluster analysis and its application to healthcare claims data: a study of end-stage renal disease patients who initiated hemodialysis. BMC Nephrol. 2016;17:25.
84. McMurdie PJ and Holmes S. Waste not, want not: why rarefying microbiome data is inadmissible. PLoS Comput Biol. 2014;10 4:e1003531.
85. Liaw A and Wiener MJRn. Classification and regression by randomForest. 2002;2 3:18-22.
86. Wilcoxon F. Individual comparisons of grouped data by ranking methods. Journal of economic entomology. 1946;39 2:269-70.
87. Benjamini Y, Drai D, Elmer G, Kafkafi N and Golani I. Controlling the false discovery rate in behavior genetics research. Behavioural brain research. 2001;125 1-2:279-84.
88. Love M, Anders S and Huber W. Differential analysis of count data–the DESeq2 package. Genome Biol. 2014;15 550:10.1186.
89. Kendall MG. Rank correlation methods. 1955.
90. Olsson U, Drasgow F and Dorans NJ. The polyserial correlation coefficient. Psychometrika. 1982;47 3:337-47.
91. Dhakan DB, Maji A, Sharma AK, Saxena R, Pulikkan J, Grace T, et al. Supporting data for "The unique composition of Indian gut microbiome, gene catalogue and associated faecal metabolome deciphered using multi-omics approaches" *GigaScience* Database 2019. http://dx.doi.org/10.5524/100548

Table 1. Metagenomic datasets used for comparative analysis (Meta-analysis) of the microbiome and MGWAS

Dataset	No. of samples	Sequence data (GB)	No. of genes
INDIA	110	110	4,809,378
USA	74	441	6,521,885
DENMARK	85	103.87	7,141,214
CHINA	71	180.78	5,464,702

Table2. PERMANOVA to assess the effect of Covariates on metabolomics profiles of samples

Variable	Sum of Sq	Mean Sq	F-Model	R^2	P-value
**Location**	0.05841	0.058406	4.9423	0.04455	0.0009
**Diet**	0.04701	0.04701	4.2132	0.03586	0.0009
**Age**	0.01618	0.01618	1.4505	0.0123	0.161
**Gender**	0.00488	0.00488	0.4370	0.00373	0.927

Table3. OPLS-DA model and its validation for different covariates as class of separation

Variable	R^2X	Q^2 (cumulative)	pR^2	pQ^2
**Location**	**0.165**	**0.205**	**0.005*****	**0.005*****
Diet	0.168	0.123	0.005***	0.005***
Age	0.155	-0.00067	0.075	0.065
Gender	0.106	-0.247	0.145	0.96
Cluster (Genus based)	0.16	0.15	0.005***	0.005***

pR^2 and pQ^2 show p-values for validation of OPLS-DA model with p value < 0.01 shown as significant (*)

Figure title and legends

Fig. 1. Comparison of Indian gut microbiome with other major populations using 16S rRNA gene and metagenomic datasets. (A) Percentage of total reads that could be mapped to IGC and updated IGC containing Indian gene catalogue. Plotted are interquartile ranges (IQR in boxes), median (as dark lines in the boxes), lowest and highest values within 1.5 times the IQR (shown as whiskers extending from boxes) and outliers as points beyond these whiskers. The blue and red boxes showed percentage of reads mapped to IGC and updated IGC (containing the Indian microbial genes). **(B)** Principal Component Analysis using MGS/CAG proportion derived from MGWAS. The samples are plotted along with the MGS/CAGs having taxonomic annotations. The MGS/CAGs are coloured according to their phylum. Variations across populations are shown using PC1 and PC2 along with factor loadings of major MGS/CAGs as biplots. **(C)** Illustration of proportions of bacterial families in different populations and their composition as determined from 16S rRNA gene datasets (adult population only). The mean family compositions of abundant families (≥1%) are represented in separate pie plots from 10 different country-wise datasets, showing their overall microbial composition compared to Indian population.

Fig. 2. Functional variations and differences between Indian populations and other populations determined from core & accessory microbial functions. (A) Procrustes analysis was performed on Bray Curtis distances calculated from core EggNOG and accessory EggNOG abundance tables in all populations. PCA analysis shows the concordance of core and accessory functions in India, Denmark, USA and China populations. The red and black lines are associated with core and accessory datasets, respectively. **(B)** Eigenvalues calculated from PCA of samples using core EggNOGs and accessory EggNOGs are plotted. The boxplots showing for core and accessory eigenvalues for all samples in different populations are shown. Each box plot represents

the median shown as white line between the boxes, the upper and lower ends of the boxes representing upper quartile (75th percentile) and lower quartile (25th percentile). The whiskers extending on both the ends represent 2.5* IQR (Inter Quartile Range). The different coloured dots overlaid for each sample are plotted over the box. The enrichment or depletion of (C) Eggnog, and (D) Kegg functions in India compared to other populations are shown as volcano plots. The log-transformed FDR Adj. P-values calculated from negative binomial-based Wald test from DESeq2 are plotted on the x-axis. The log odds ratio calculated for India vs Other datasets are plotted on the y-axis. The EggNOGs/KOs with P-value<0.05 are shown in Blue whereas those having P-values>0.05 are shown in Red. The EggNOGs/KOs extending on right and left side and with P-value>0.05 are labelled as highly enriched in India and other datasets, respectively.

Fig. 3. Variations in gut microbiome at the two locations. (A) Between class Analysis, which visualizes results from PCA and clustering, using genus level abundance from 37 cross national dataset and genus abundance of 110 Indian samples obtained from mapping of reads to reference genomes. The samples from LOC1 (cyan), LOC2 (pink) and 37 cross national samples from Arumugam et al. (grey and labelled) are placed into three distinct enterotypes based on clustering. **(B)** Significantly different genera (FDR Adj. P-value < 0.05; NB model-based Wald test) between the two locations are shown as boxplots with boxes representing interquartile range (IQR), dark lines between the boxes representing median values and whiskers representing the 1.5 x IQR on each side. **(C)** Scatterplot of log-transformed mean values of species abundance in LOC1 (n=53) and LOC2 (n = 57) individuals. Red colour gradient points represent differentially abundant (FDR Adj. P< 0.05; NB model-based Wald test) species with lower p-values from Red to Blue.

Fig. 4. Between class analysis to identify metabotypes and their associated metabolites. (A) Metabolite clusters (MES) abundance profiles of samples were generated and their clustering was

performed using PAM (partition around medoids) clustering. The between class and PCA of JSD distances and PAM clustering identified 3 clusters to be optimum for their segregation using **(B)** Silhouette index. The metabolites valeric acids, and saturated fatty acids such as palmitic acid and stearic acid, were found higher in Cluster1. The carbohydrates such as glucose and galactose were found higher in Cluster2. The branched chain amino acids, lauric acid and butyric acid were found higher in Cluster3. **(C)** OPLS-DA analysis using locations as classes shows locations as differentiating factors in separating the samples based on their metabolomic profiles.

Fig. 5. Spearman's Rank correlations of metabolites with species and metabolic modules. (A) Spearman's Rank Correlation coefficients were calculated between significantly different metagenomic species and significantly different metabolites between LOC1 and LOC2 populations. The correlations showing significant FDR Adj. $P < 0.05$ are plotted. The bars on the right show the Log Odds Ratio of the abundance of MGS with positive values indicating enrichment in LOC1, and the negative values indicating enrichment in LOC2. **(B)** Spearman's Rank correlations between significantly different (FDR Adj. $P<0.05$, NB model-based Wald test) pathway modules and significantly different metabolite abundances in all samples. The significant ($P<0.05$) correlations are plotted and the colour intensities depict the correlation coefficients. The correlation of metabolites with locations is shown with labels in dark red colours showing association with LOC2, and the labels in green colours showing correlation with LOC1.

Fig. 6. BCAA abundance and their differential correlation with LOC1 and LOC2. (A) Bar plot showing z-normalized values of serum and faecal BCAA (Valine and Isoleucine) relative concentration in LOC1 and LOC2. **(B)** The effect of specific microbial species on associations between BCAA biosynthesis pathways and BCAA levels in faecal metabolome, illustrated by change in background adjusted Spearman's correlation coefficient when a given species has been

excluded from analysis is shown (see Methods). The density plot shows the distribution of correlation for species and the changes caused by specific species as marked by lines below. **(C)** Network analysis of Spearman's correlations between the branched chain amino acids biosynthesis, degradation and transport KEGG modules with MGS abundance in both LOC1 and LOC2 populations. The node size is proportional to the degree of interactions and the links between module and MGS show interactions or significant correlations (FDR Adj. $P < 0.05$) with negative (in Red) and positive (in Blue) correlation coefficients. **(D)** Plot showing relative abundance of KOs associated with different modules of BCAA biosynthesis and transporters in LOC1 and LOC2.

Fig. 7. BCAA transporters playing a key role in maintaining the levels of BCAAs in faeces and serum

The dynamics of BCAA concentration levels in faecal and serum metabolome influenced by microbial BCAA biosynthesis and transport pathways and their differential abundance in LOC1 and LOC2 is shown

Additional Files

Additional File 1: Supplementary data containing the metadata and sample information

Additional File 2: Summary of sequencing statistics showing the number of reads per sample for 16S rRNA gene amplicon dataset

Additional File 3: Summary of sequencing statistics showing the number of reads per sample for Whole Genome Shotgun metagenomic dataset

Additional File 4: Summary of the reads mapped to Integrated Gene Catalogue and Indian catalogue combined with IGC.

Additional File 5: Figures S1 to S18

Additional File 6: Differentially abundant MGS between India and other populations

Additional File 7: Differentially abundant functions (Kegg Orthologues (KOs) and EggNOGs) between India and other populations.

Additional File 8: Sample-wise representation of Indian samples into Enterotypes identified from Meta-analysis with 37 samples from four nations used in Arumugam et al.

Additional File 9: Calinski Harabasz index and prediction strength calculated for clusters derived from 16S rRNA gene based genus abundance, metagenome based species abundance and metagenome based KO abundance profiles.

Additional File 10: Mean relative abundance of genus in Cluster-1 and Cluster-2 and their associated P-values of difference calculated using NB model based Wald test.

Additional File 11: The sample-wise association into clusters using Genus based and KO based clustering and their differences.

Additional File 12: Differentially abundant KEGG orthologue functions between Cluster-1 and Cluster-2.

Additional File 13: Polyserial correlation of covariates with principal components explaining variations across samples using unweighted UniFrac distances.

Additional File 14: Differentially abundant MGS observed between two locations and their enrichment calculated using Log Odds ratio and NB model based P-values.

Additional File 15: Polyserial correlation of covariates with principal components explaining variations across samples using metabolomics data.

Additional File 16: Table shows the Spearman's rank correlation coefficient values of metabolites with Metabotypes.

Additional File 17: Table shows the differential abundance of KEGG Modules between LOC1 and LOC2

Additional File 18: List of reference genomes from NCBI and HMP databases for reference mapping

Fig.1

Click here to access/download;Figure;FIGURE 1.jpg

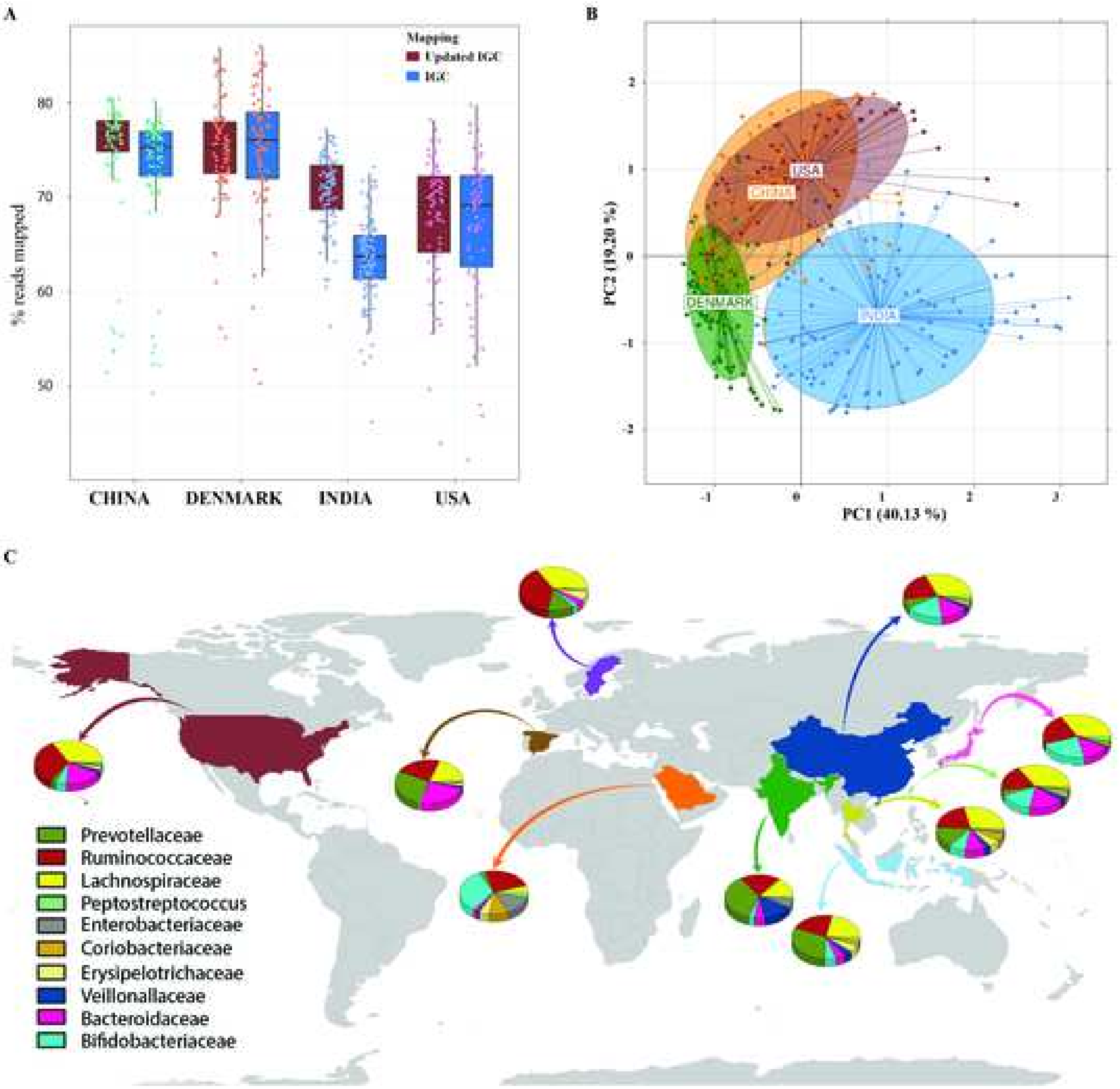

Fig.2

Click here to access/download;Figure;FIGURE 2.jpg

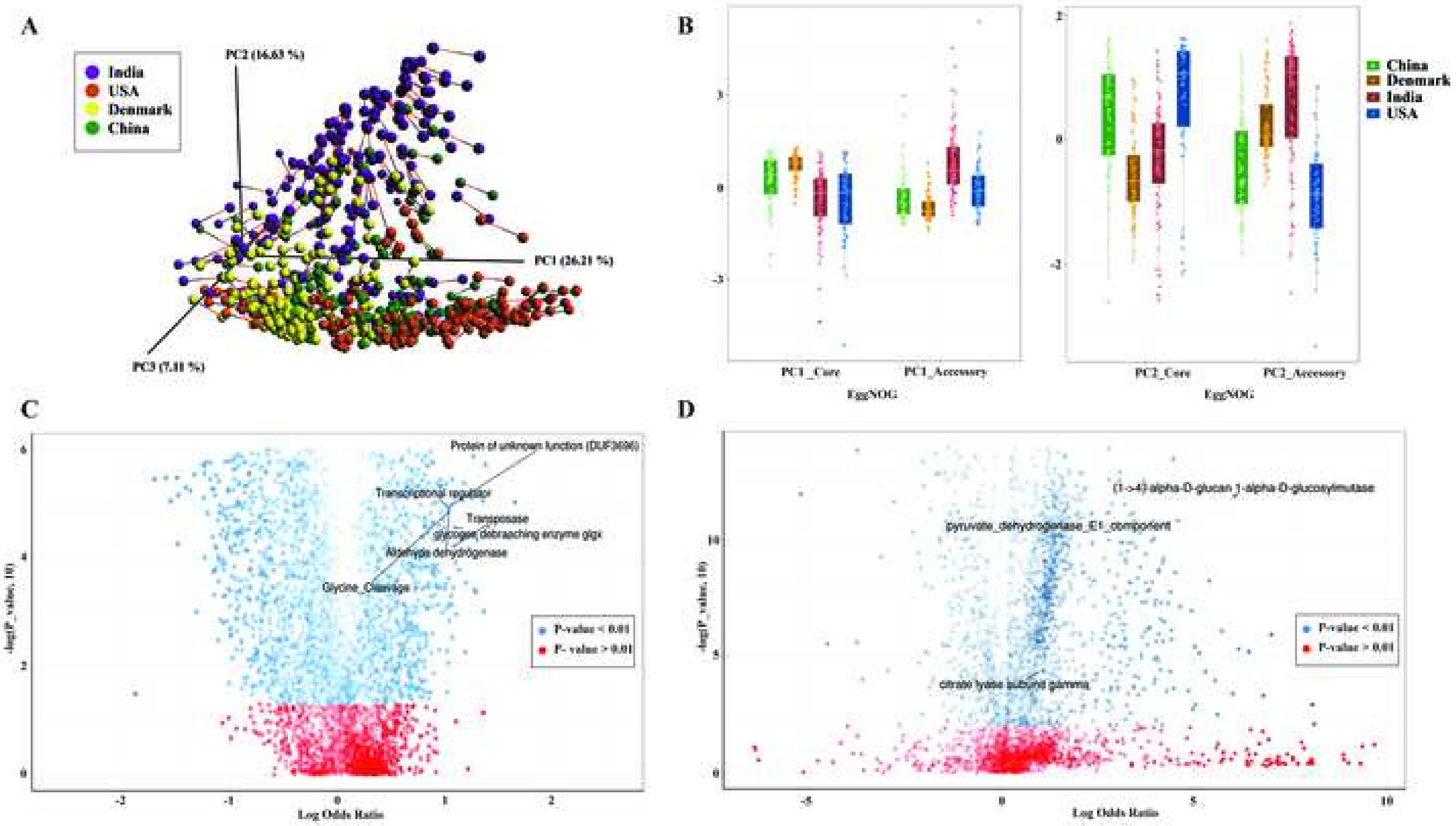

Fig.3

Click here to access/download;Figure;FIGURE 3.jpg

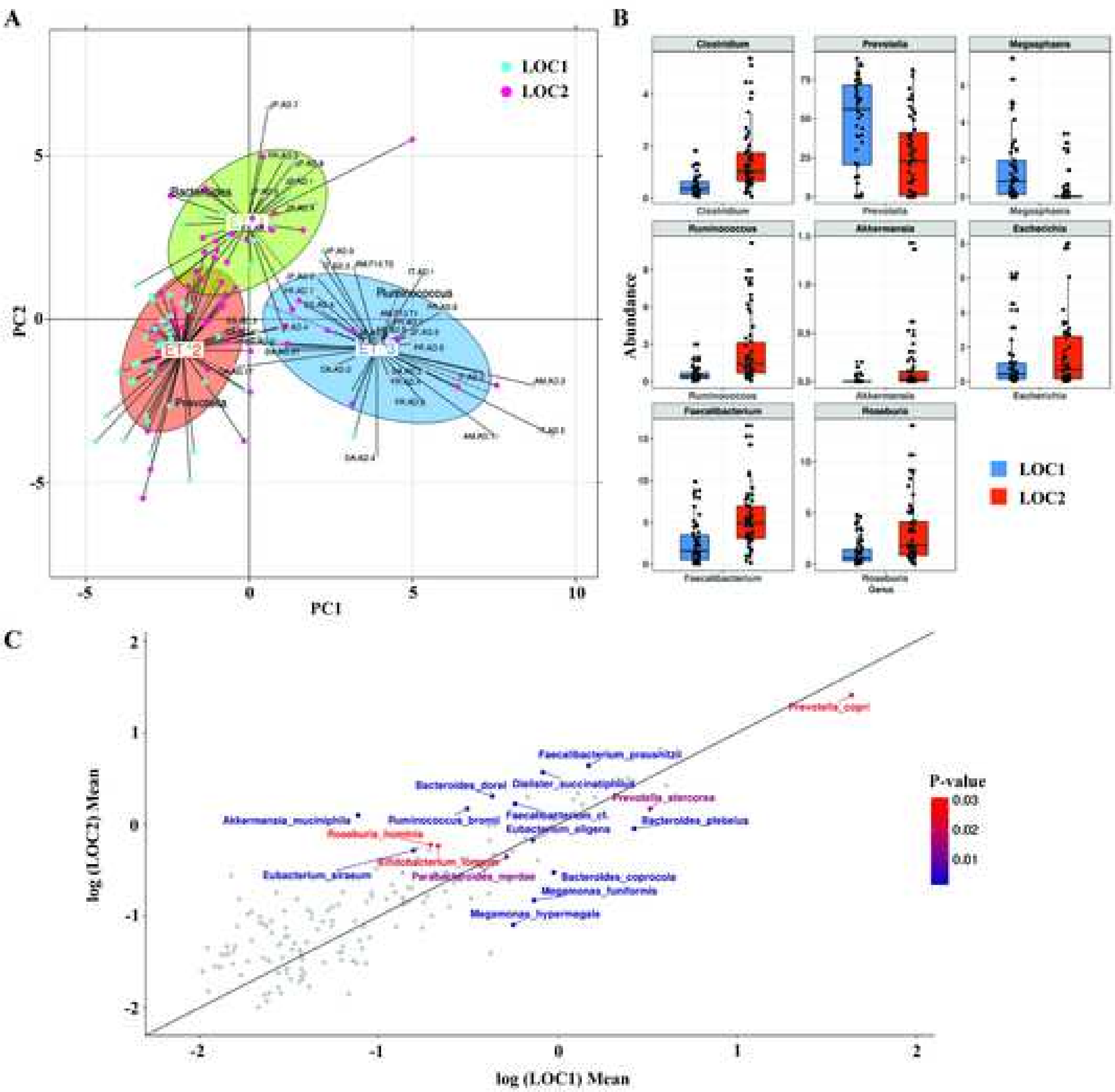

Fig.4

Click here to access/download;Figure;FIGURE 4.jpg

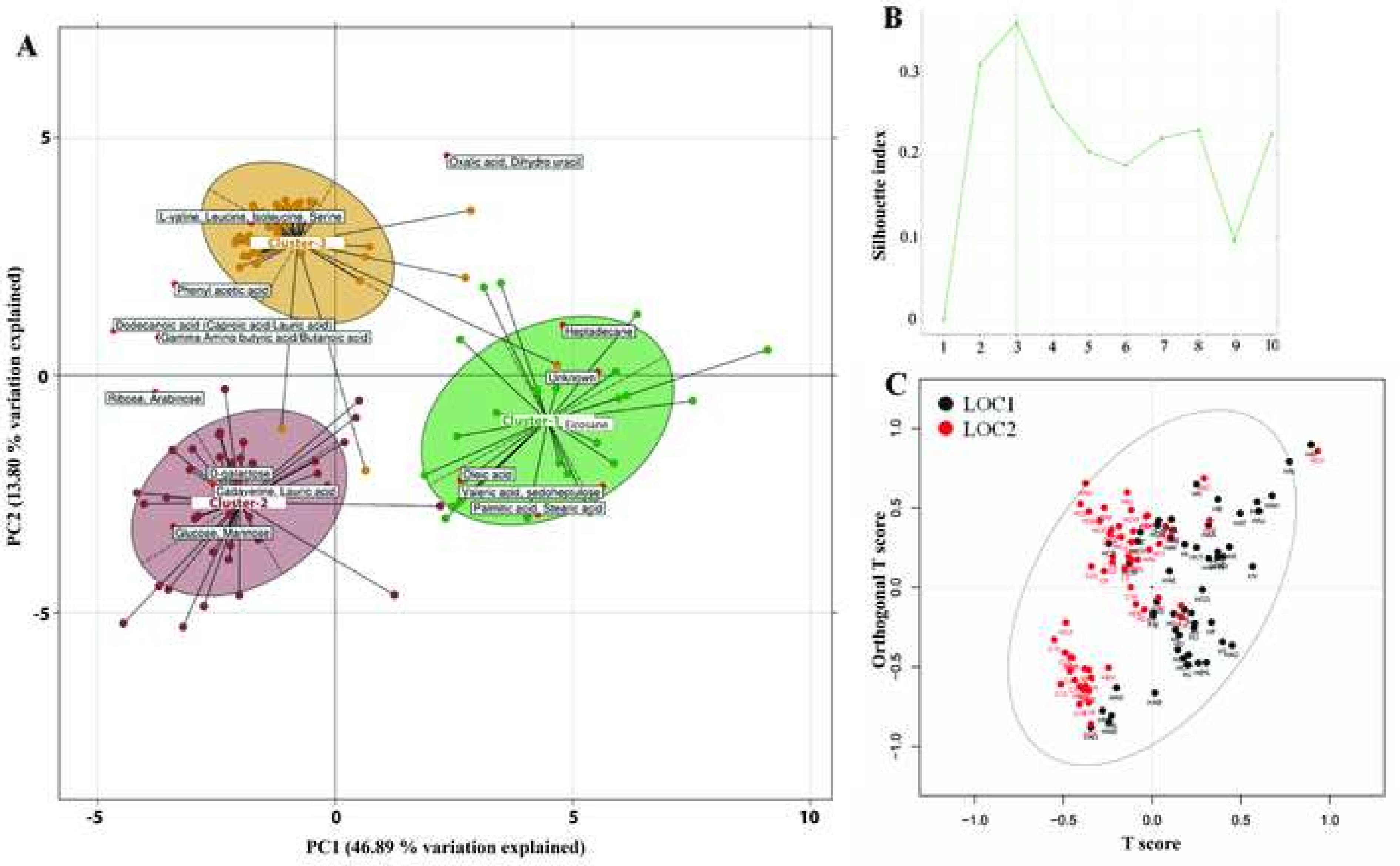

Fig.5

Click here to access/download;Figure;FIGURE 5.jpg

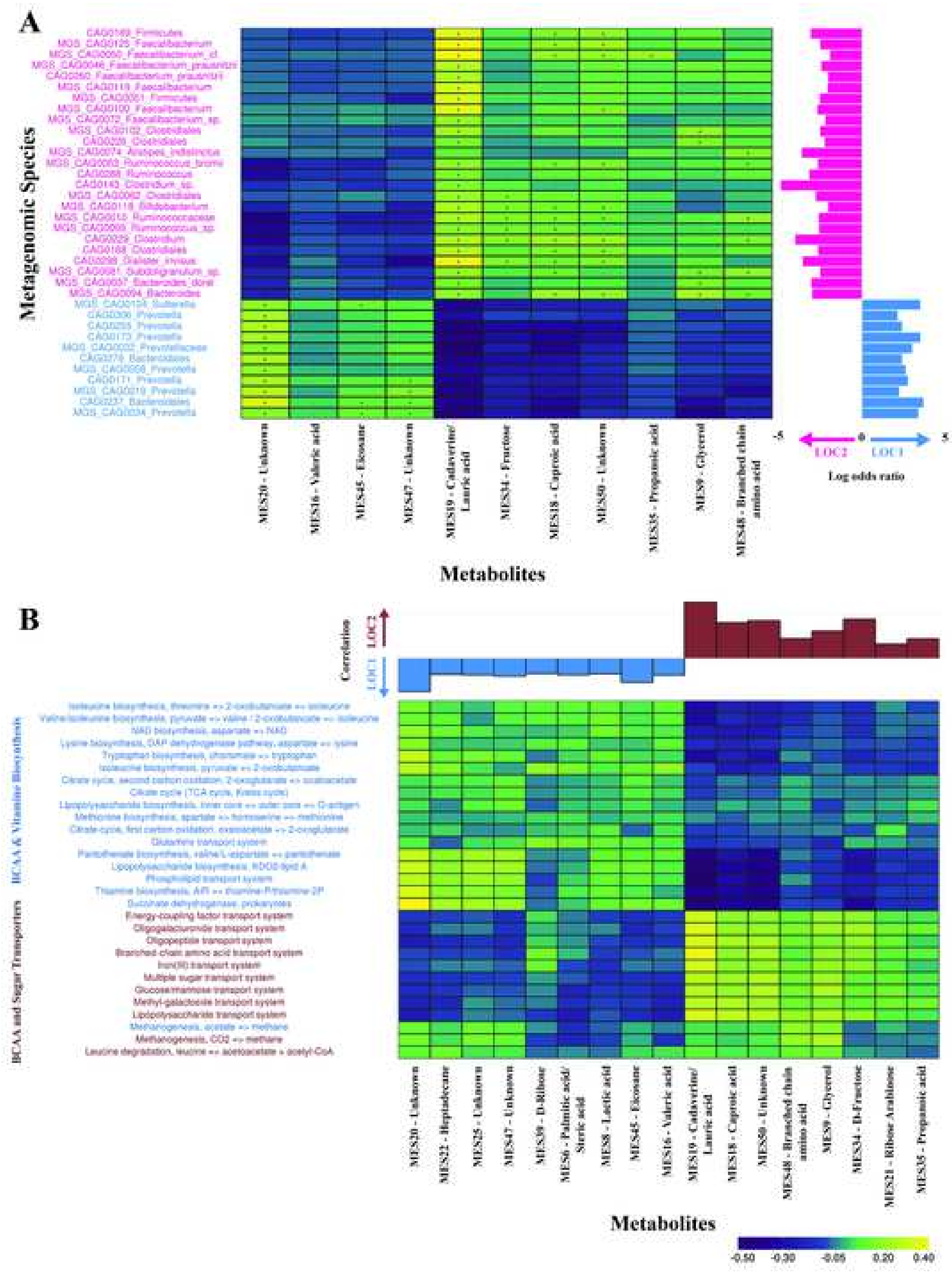

Fig.6

Click here to access/download;Figure;FIGURE 6.jpg

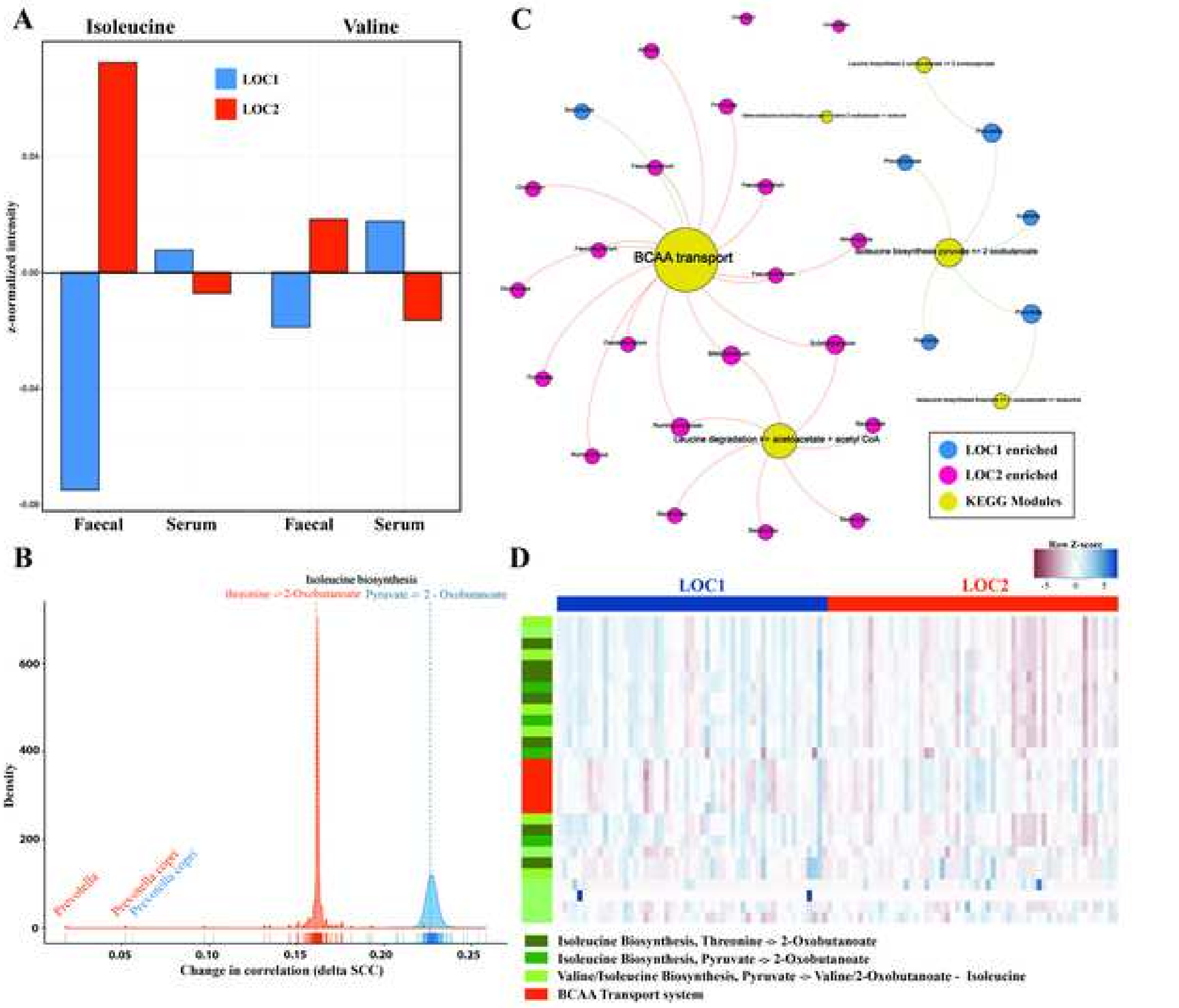

Fig.7

Click here to access/download;Figure;FIGURE 7.TIF

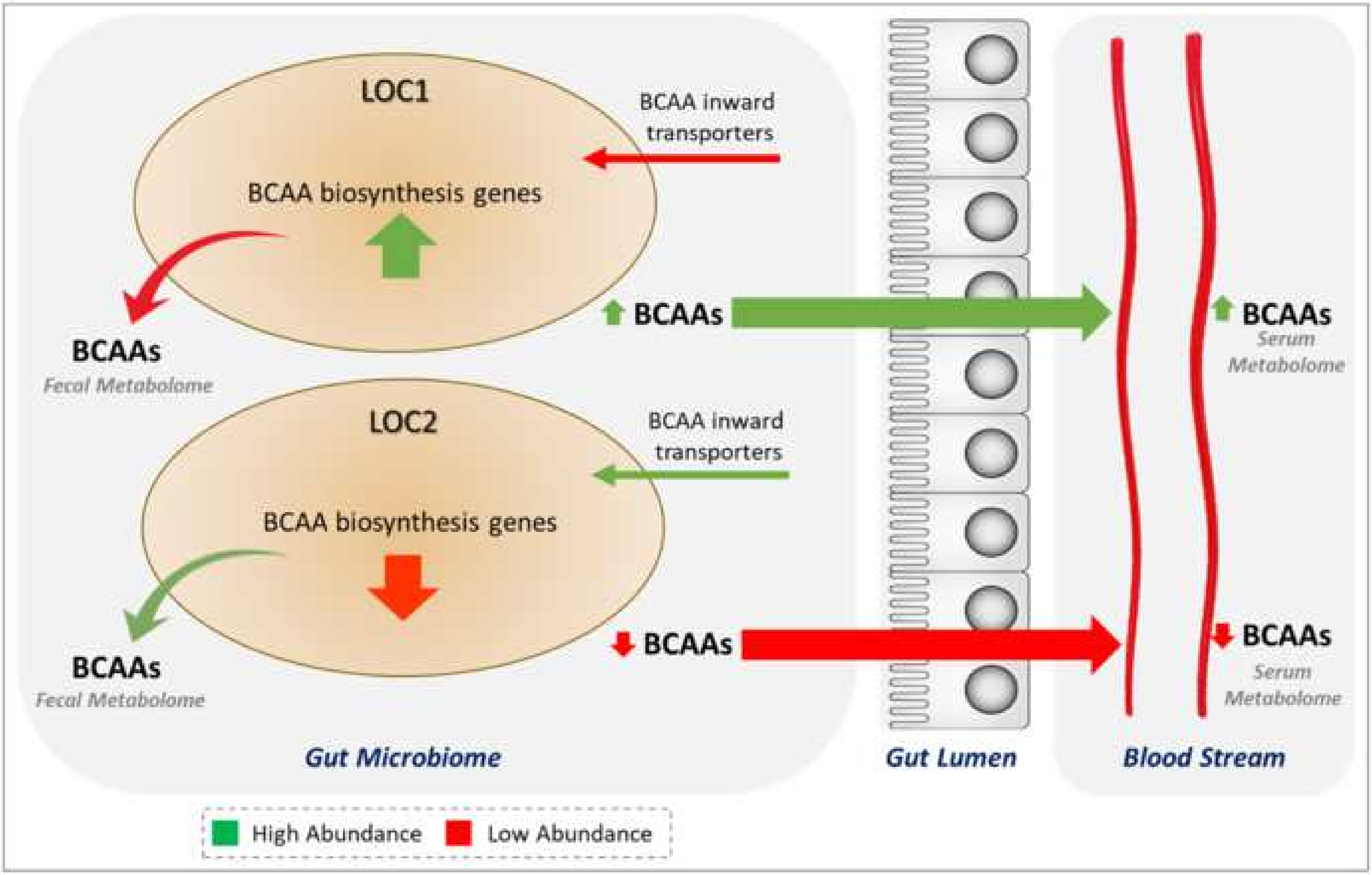

Additional File 1

Click here to access/download
Supplementary Material
Additional_file_1.doc

Additional File 2

Click here to access/download
Supplementary Material
Additional_file_2.xlsx

Additional File 3

Click here to access/download
Supplementary Material
Additional_file_3.xlsx

Additional File 4

Click here to access/download
Supplementary Material
Additional_file_4.xlsx

Additional File 5

Click here to access/download
Supplementary Material
Additional_file_5.docx

Additional File 6

Click here to access/download
Supplementary Material
Additional_File_6.xlsx

Additional File 7

Click here to access/download
Supplementary Material
Additional_file_7.xlsx

Additional File 8

Click here to access/download
Supplementary Material
Additional_file_8.xlsx

Additional File 9

Click here to access/download
Supplementary Material
Additional_file_9.xlsx

Click here to access/download
Supplementary Material
Additional_file_10.xlsx

Additional File 11

Click here to access/download
Supplementary Material
Additional_file_11.xlsx

Additional File 12

Click here to access/download
Supplementary Material
Additional_file_12.xlsx

Additional File 13

Click here to access/download
Supplementary Material
Additional_file_13.xlsx

Additional File 14

Click here to access/download
Supplementary Material
Additional_file_14.xlsx

Additional File 15

Click here to access/download
Supplementary Material
Additional_file_15.xlsx

Additional File 16

Click here to access/download
Supplementary Material
Additional_file_16.xlsx

Additional File 17

Click here to access/download
Supplementary Material
Additional_file_17.xlsx

Additional File 18

Click here to access/download
Supplementary Material
Additional_file_18.xlsx

Supplementary Figure 1

Click here to access/download
Supplementary Material
Supplementary_Figure_1.jpg

Supplementary Table 1

Click here to access/download
Supplementary Material
Supplementary_Table_1.xlsx
