# Supplementary material for: The unique composition of Indian gut microbiome, gene catalogue, and associated fecal metabolome deciphered using multi-omics approaches
Source: Gigascience. 2019 Jan 30;8(3):giz004. doi: 10.1093/gigascience/giz004 (PMC6394208; doi:10.1093/gigascience/giz004)
Supplement: Supplemental Files [file giz004_supplemental_files.zip › Additional_file_5.docx]

**Additional File 5**

**
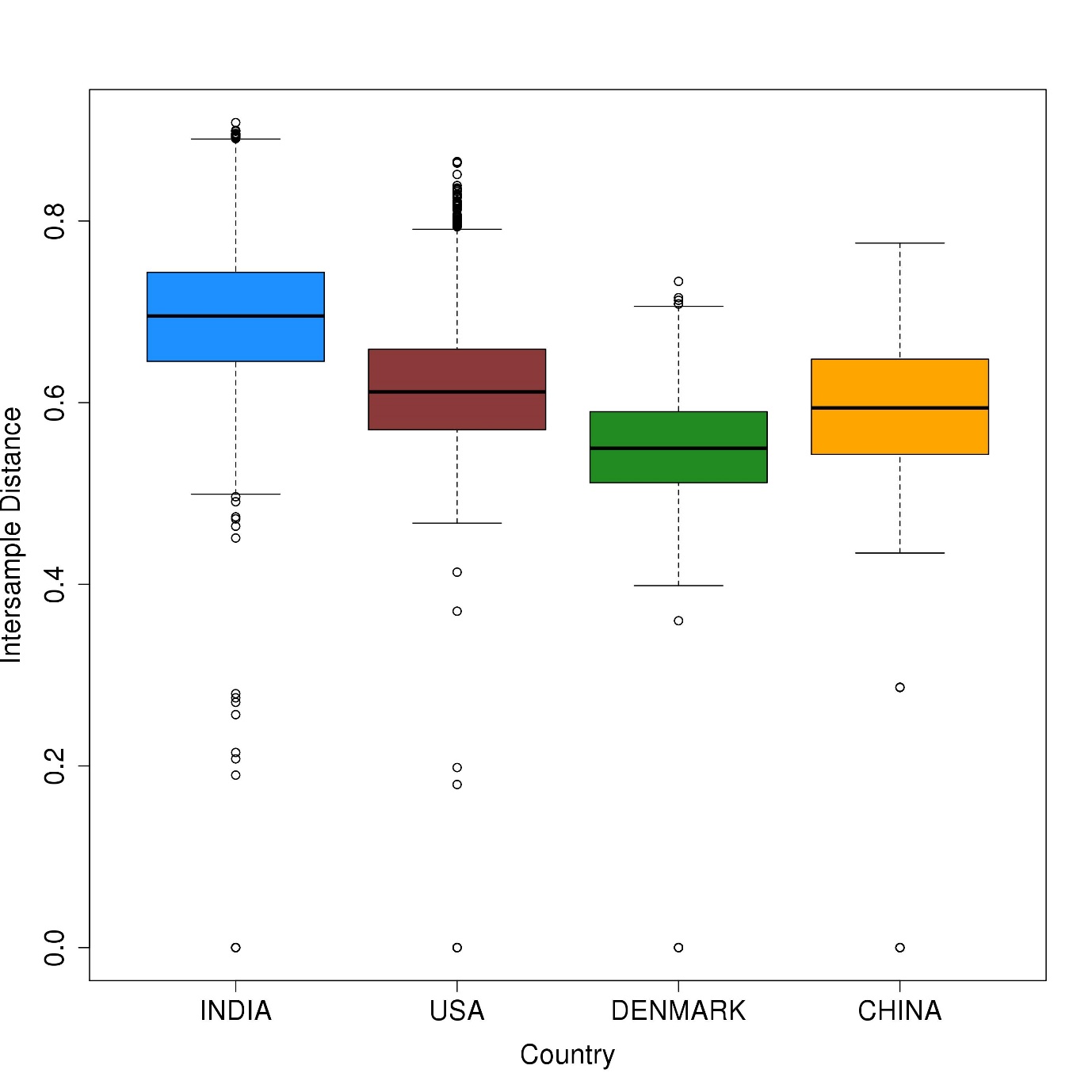
**

**Figure S1. Inter-sample Canberra distances between samples from four studies and their comparison**

The inter-sample Canberra distances calculated using MGS abundance between samples from India, USA, Denmark and China are shown using boxplots. The inter-sample distances between samples within each country shown as median values represented by dark line between boxes, the ends of boxes representing upper quartile (75^th^ percentile) and lower quartile (25^th^ percentile). The whiskers are extended on both sides upto 1.5*IQR (Interquartile range). The unfilled circles represent outliers that fall outside 1.5*IQR range.

**
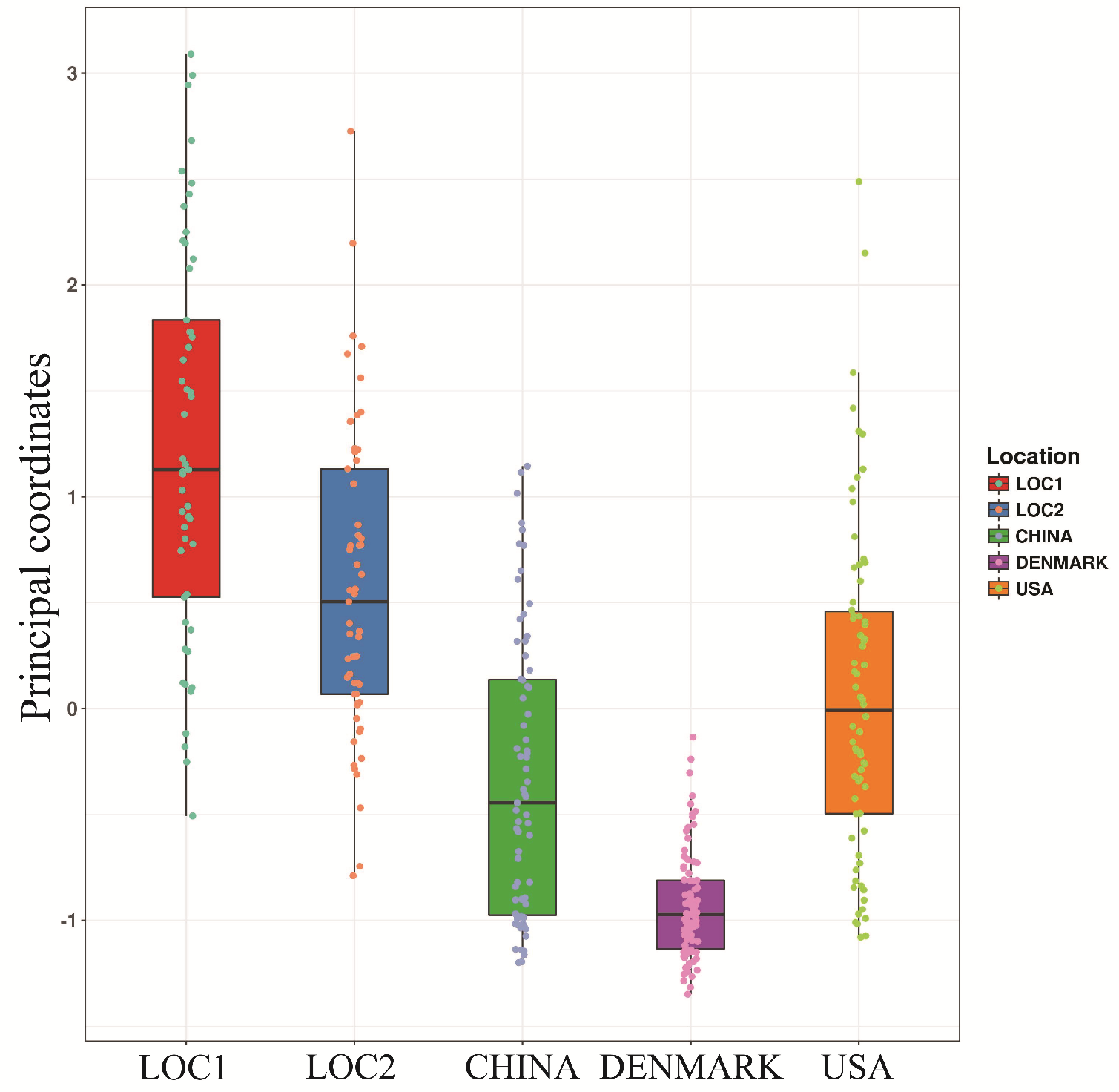
**

**Figure S2.** **Principal coordinates of samples from different location in a multicohort analysis using MGS abundance.**

The boxplots represent coordinates of samples at PC1 plotted using MGS relative abundance. The differences in loadings of Indian samples from LOC1 and LOC2 which resulted in spread of samples from Indian population are shown. The median is represented as a line between the boxes, the ends of which represent upper quartile (75^th^ percentile) and lower quartile (25^th^ percentile). The whiskers are extending on both sides upto 1.5 * IQR (Inter-quartile range).

**
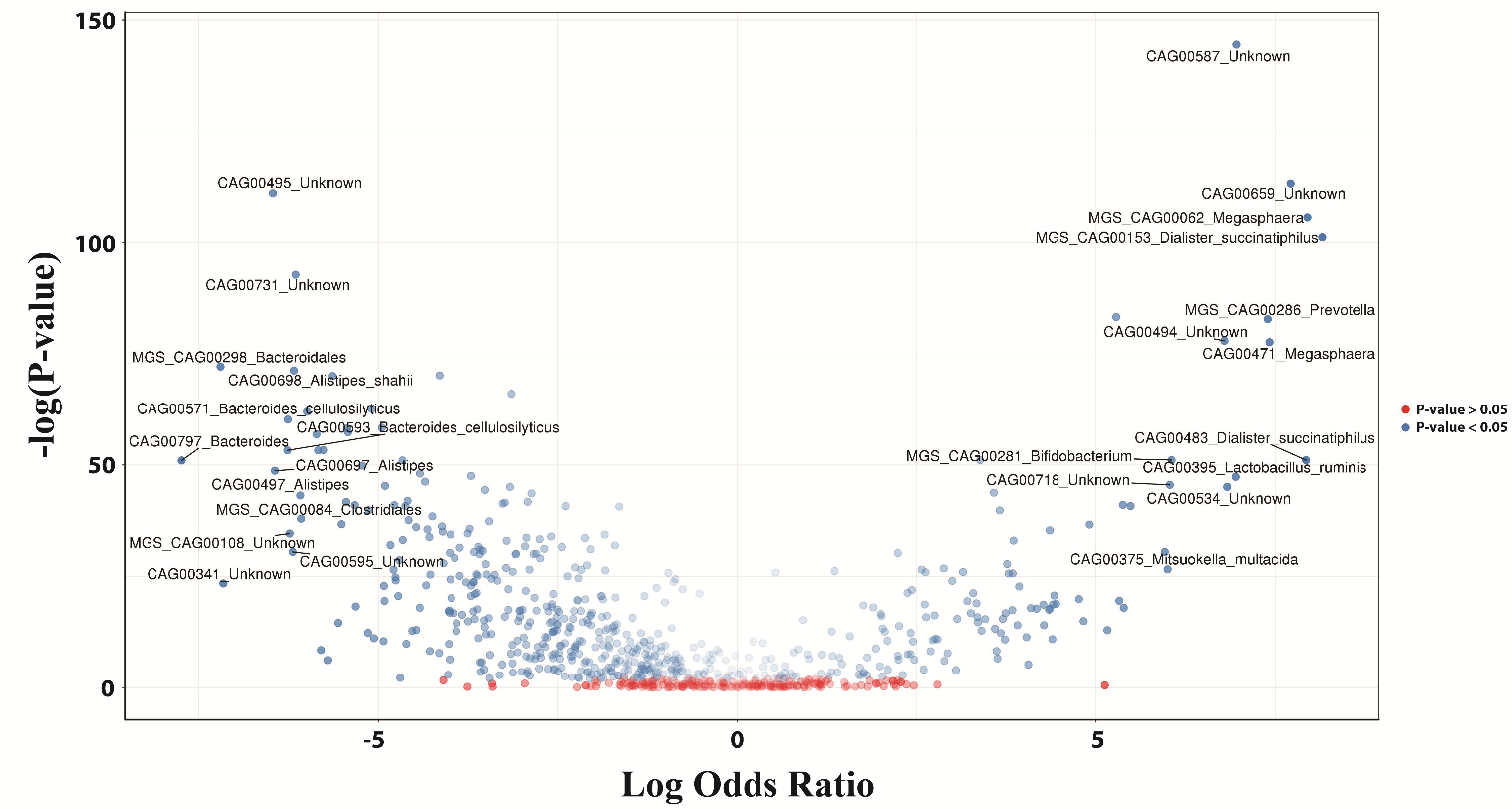
**

**Figure S3. Enrichment of MGS/CAGs in Indian population compared to other populations across the globe.**

The volcano plot shows the MGS/CAGs enriched in the Indian gut microbiome (Log Odds Ratio >1 and FDR Adj. P-Value <0.05). The functions shown in red coloured dots are non-significant (P>0.01), whereas blue coloured dots are significantly distinct between the two populations (P<0.01). The points having Log Odds Ratio > 2 are significantly enriched in Indian population, whereas the points with Log Odds Ratio < -2 are significantly depleted in Indian population.

**
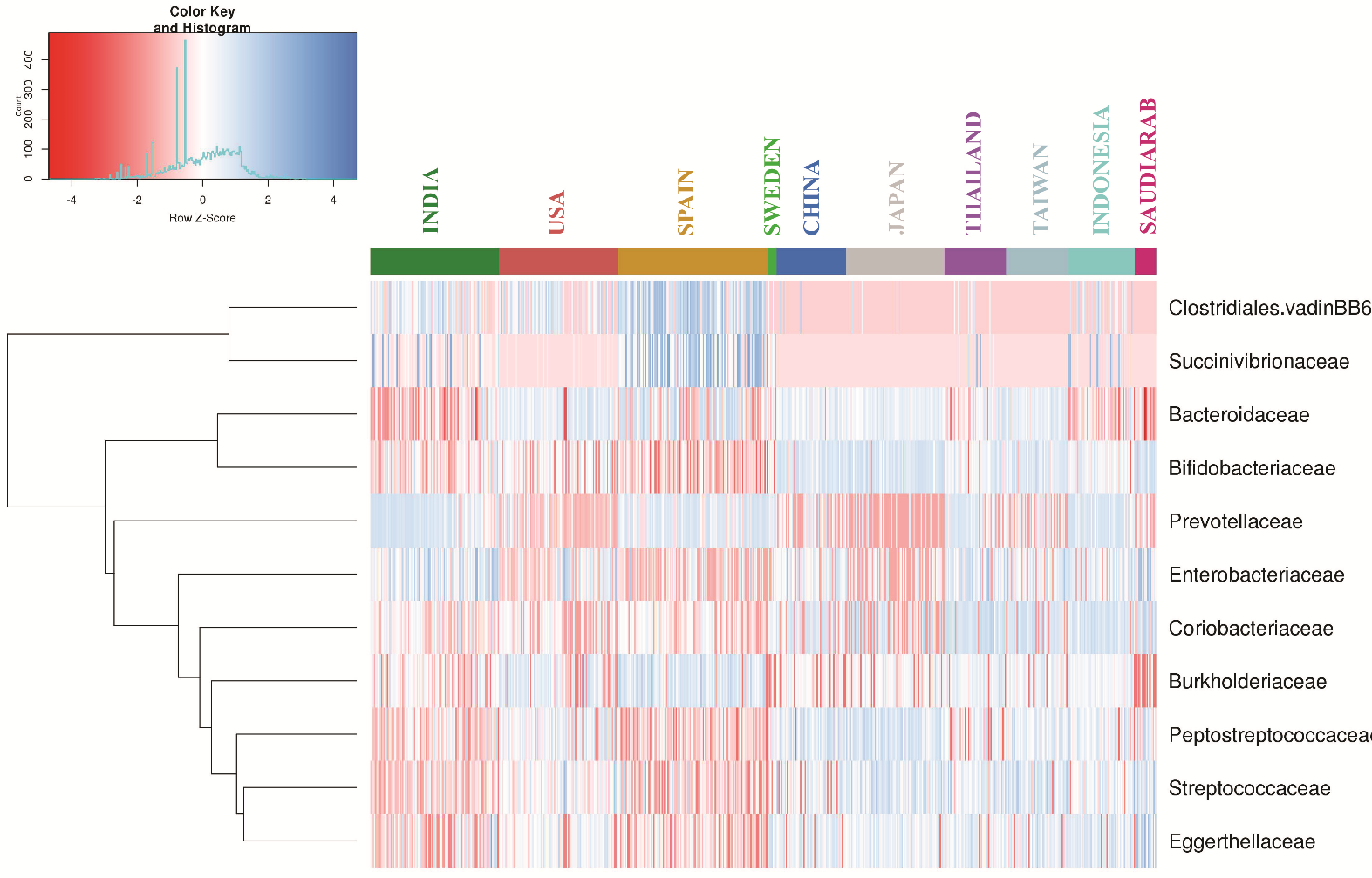
**

**Figure S4. Heatmap representation of the relative abundance of important families in 640 samples from 10 different nations.**

The relative abundance of most important families having mean decrease in accuracy > 0.01 in random forest analysis were chosen to create heatmap with dendrogram showing clustering of the families in 10 different populations using 16Sr RNA markers. The relative abundance of these families in each of the participants were subjected to log_10_ transformation followed by hierarchical clustering and were plotted on heatmap.


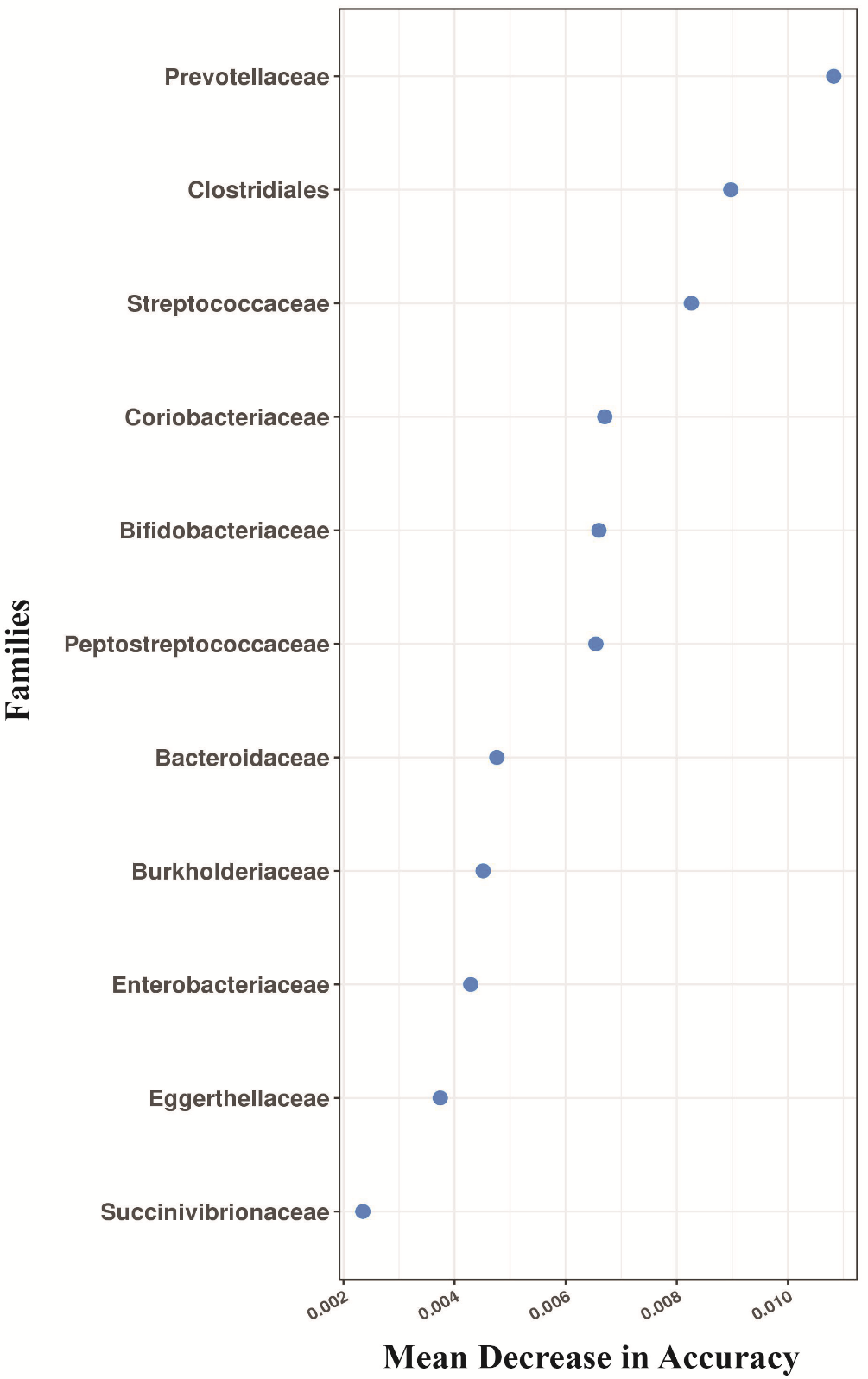


**Figure S5. Mean decrease in accuracy of abundant families for the classification of Indian samples using Random Forest.**

The Random Forest models trained using family level abundance (10000 trees and default settings) and predictive models evaluated with cross-validation error were used to calculate importance of variables iteratively. The mean decrease in accuracy of the classification of Indian samples after removal of each family are used as importance values and are ranked and plotted in decreasing order.


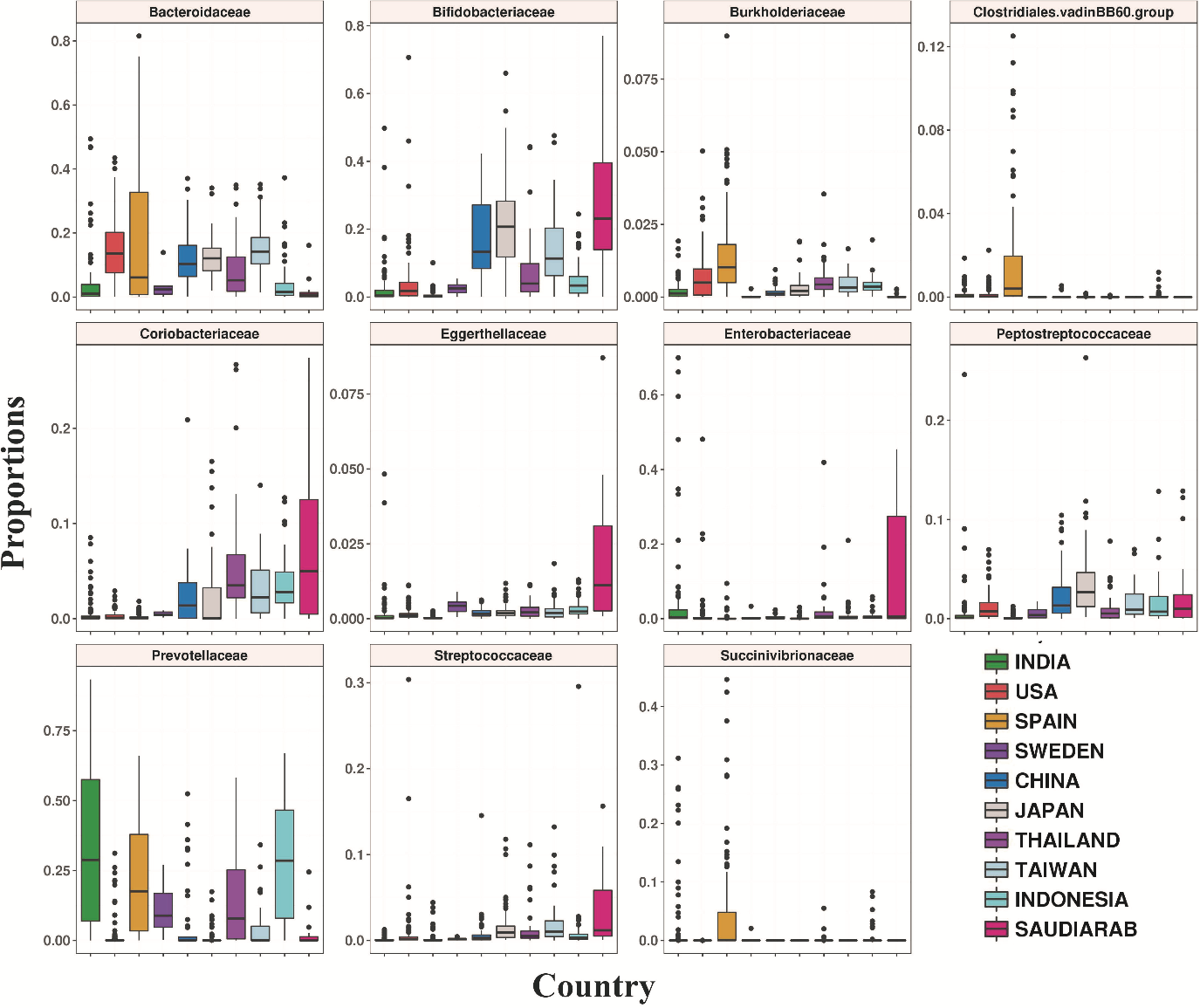


**Figure S6. Box plots showing relative abundance of important families in 10 different populations.**

The important families identified through feature selection method using random forests were subjected to pairwise Wilcoxon rank sum test. The boxplots showing the median (50^th^ percentile), upper (75^th^ percentile) and lower (25^th^ percentile) quartiles of relative abundance of families in each of the countries datasets are represented. The black points represent outliers that fall outside the range of 1.5 * IQR (interquartile range).

**
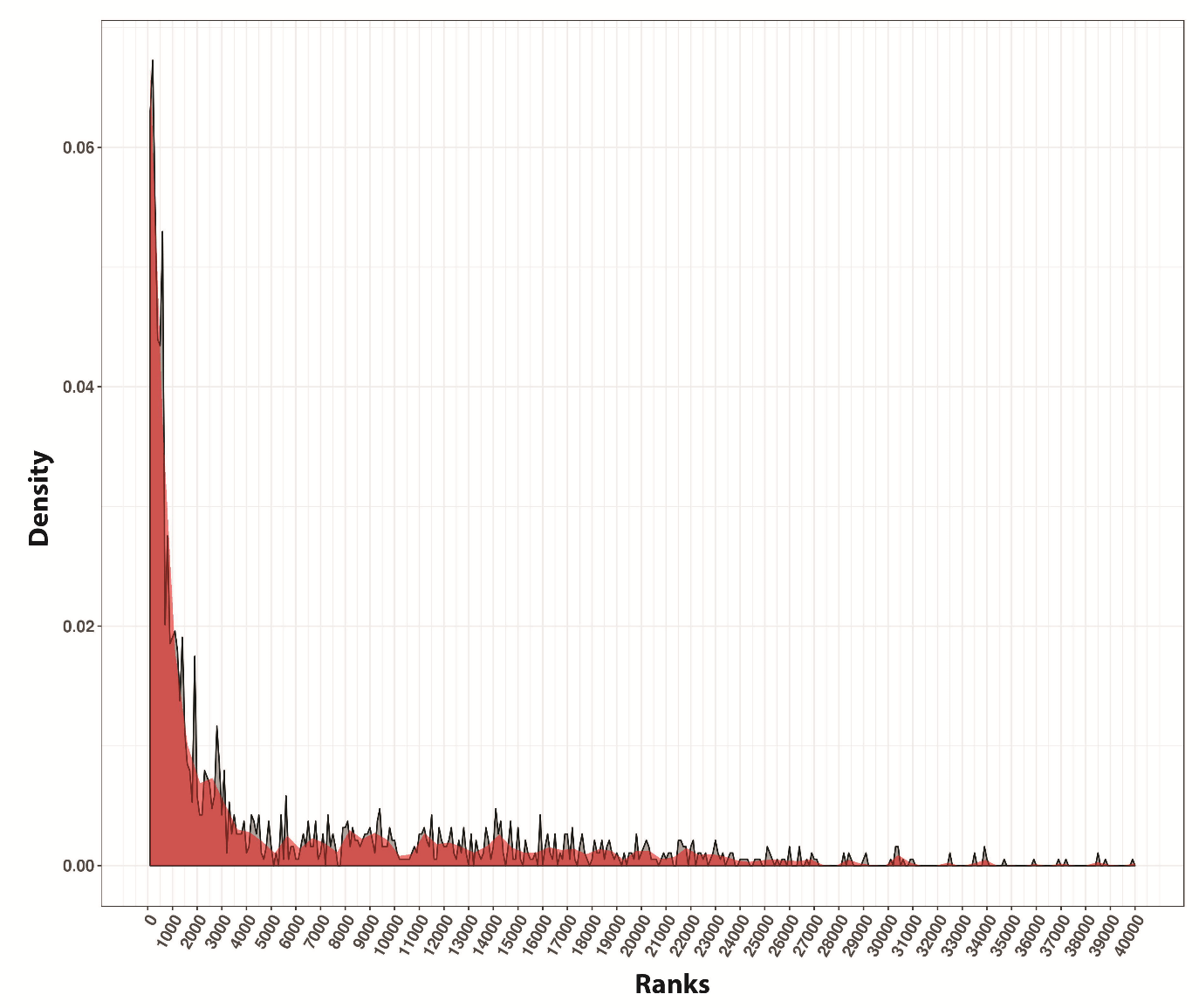
**

**Figure S7. Clusters of EggNOGs containing essential genes and their proportions.**

The EggNOGs are ranked in decreasing order by the number of genes they contain and their abundance are shown on x-axis, whereas the y-axis showing the proportion of essential genes covered by these EggNOGs clusters (clustered as 100 EggNOGs per cluster) are plotted. The number of essential genes covered by EggNOGS ranked in decreasing order shows top ranked clusters cover maximum of the essential genes.

**
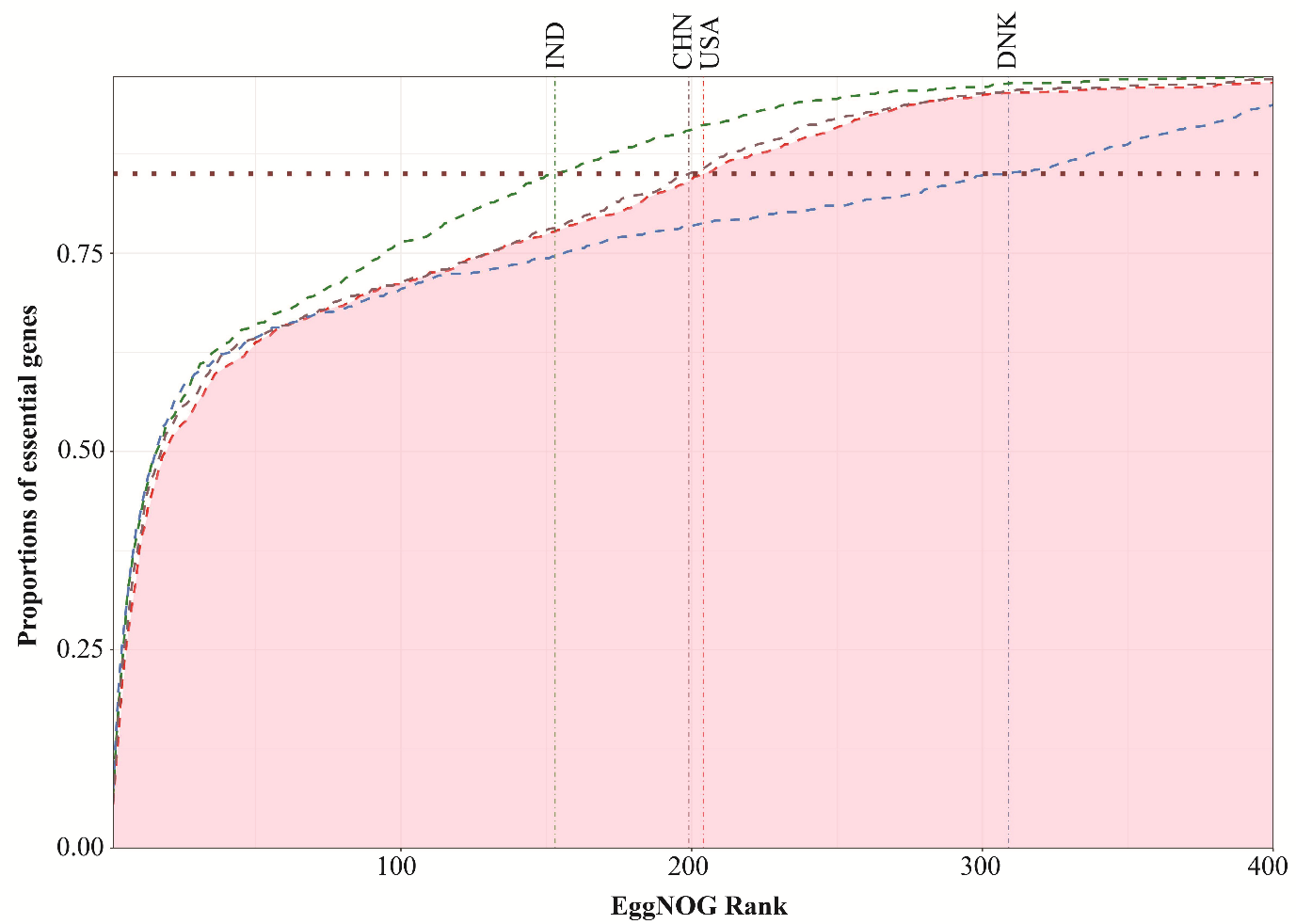
**

**Figure S8. Cumulative Frequency curves of essential genes from the four country datasets.**

Cumulative Frequency curve showing the number of clusters (grouped into 100 ranks) from all four populations covering 85% of the essential genes is plotted. The proportion of essential genes covered by highly ranked gene clusters for each of the populations (India, Denmark, USA, China) is showed. India showed highest coverage of essential genes with least number of clusters compared to other datasets.

**
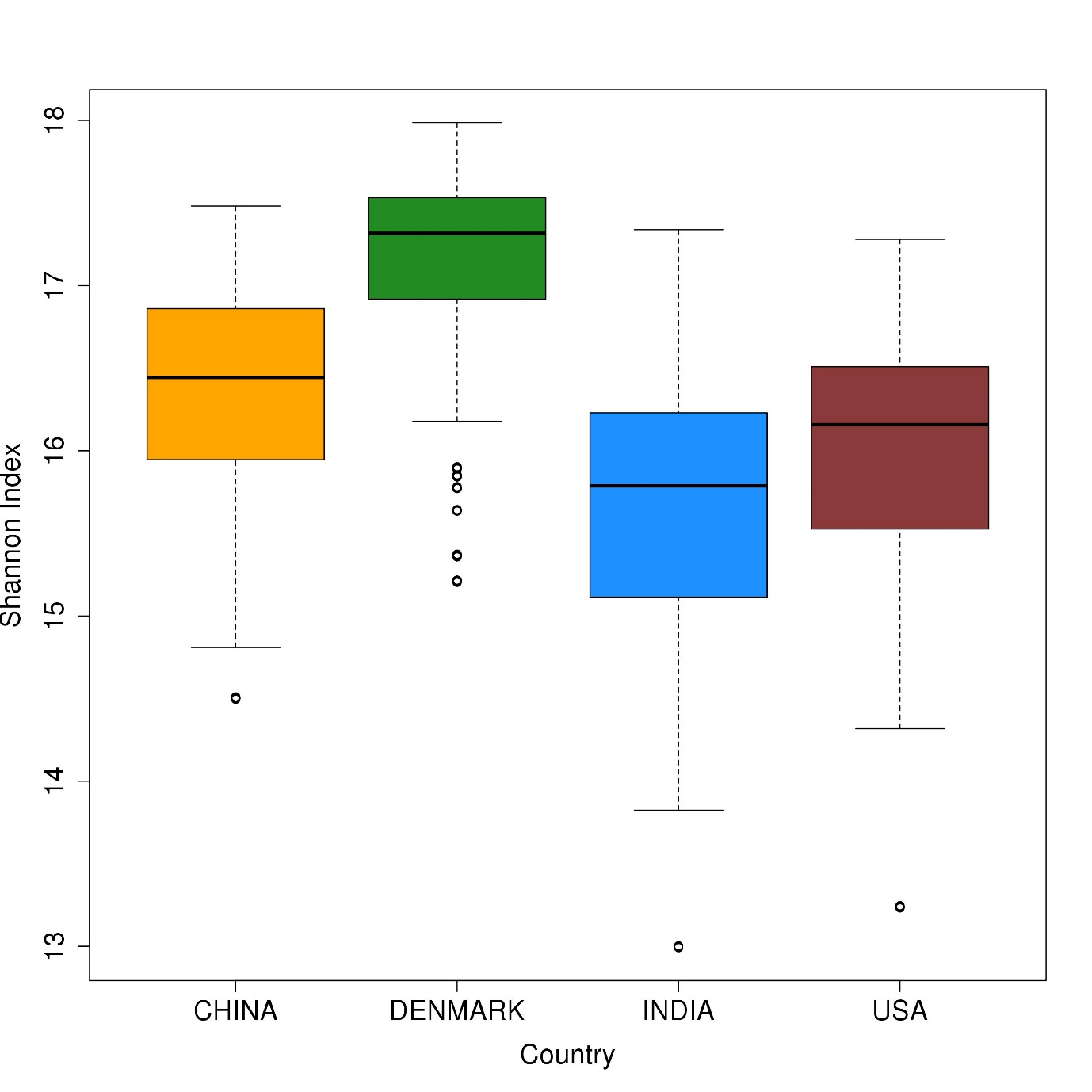
Figure S9. Comparison of alpha diversity of three prominent country datasets with Indian gut microbiome.**

The mean Shannon diversity indices were calculated using gene abundance tables rarefied at equal depth of 100,000 seqs/sample and n=30 iterations for India, China, Denmark and USA metagenomic datasets. The Box plots showing median (50^th^ percentile), upper quartile (75^th^ percentile) and lower quartile (25^th^ percentile) represent the variations in diversity across populations. The circles represent outliers present in each dataset. India showed significantly lower diversity (P-value <10^-16^) from other populations.

**
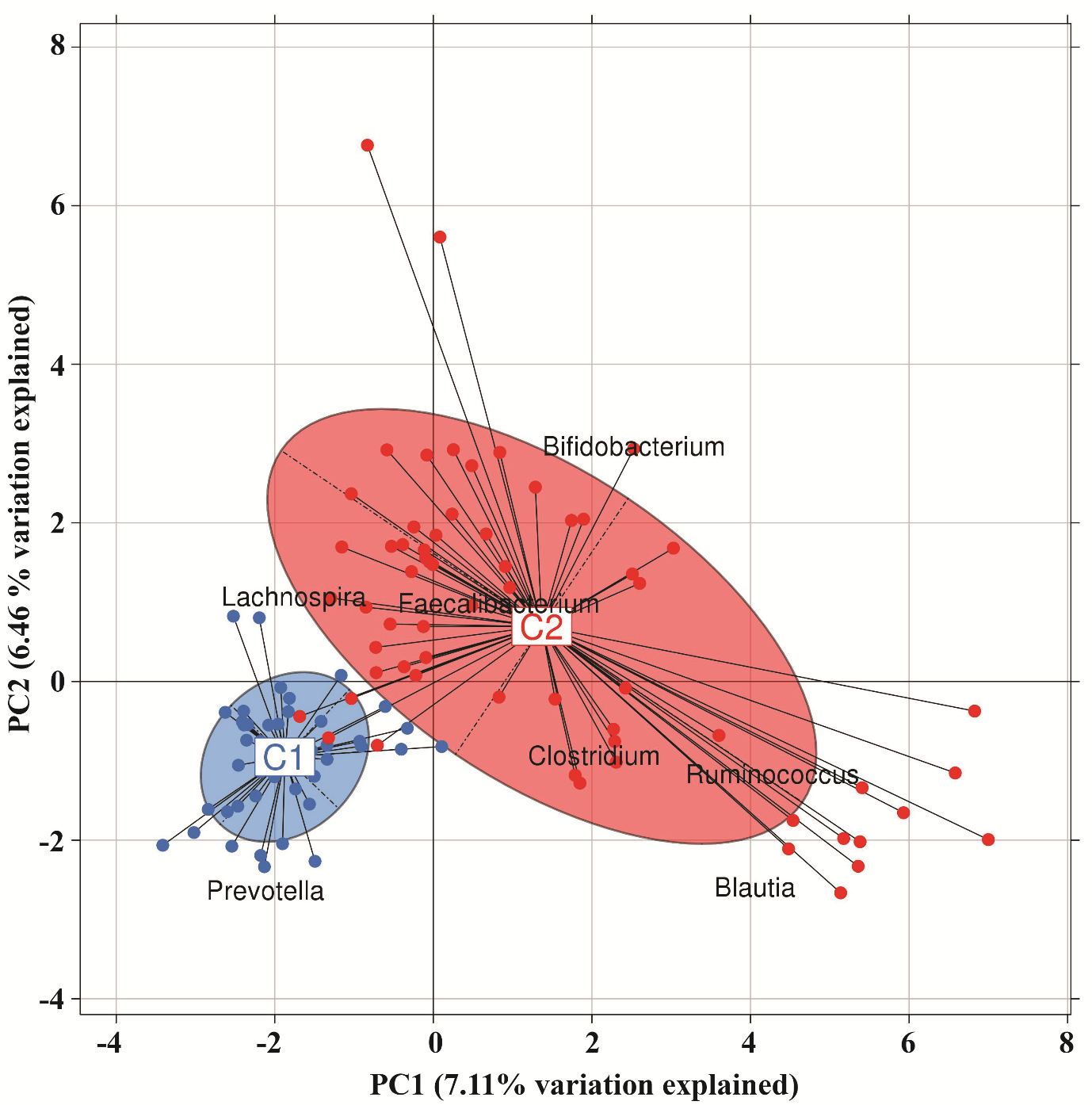
**

**Figure S10. Principal Component Analysis and clustering of Indian gut microbiome samples into distinct clusters using genus level composition derived from 16S rRNA sequencing.** The clustering was performed using Jensen-Shannon distances (JSD) and PAM clustering. The optimal number of clusters was chosen by maximizing the Calinski Harabasz (CH) index and was validated based on prediction strength. The between class analysis was performed to visualize the clustering. The topmost genus and species having maximum factor loading were considered as the driver species of these clusters. The PC1 and PC2 with their variance explained are shown in x and y-axis.


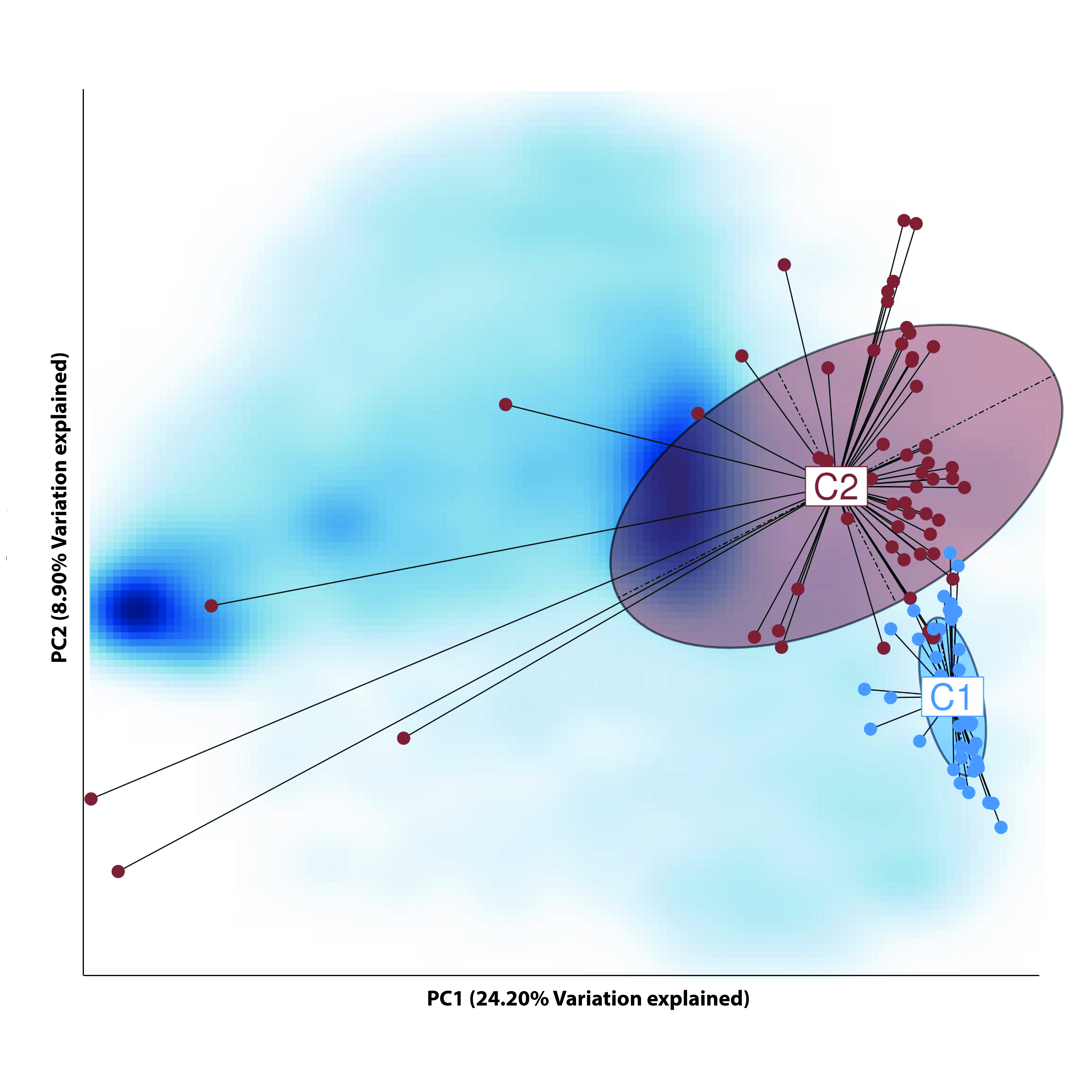


**Figure S11. Principal Component Analysis and clustering of Indian gut microbiome samples into distinct clusters using KO abundance derived from metagenomic datasets.** The clustering was performed using Jensen-Shannon distances (JSD) and PAM clustering. The optimal number of clusters was chosen by maximizing the Calinski Harabasz (CH) index and was validated based on prediction strength. The between class analysis was performed to visualize the clustering. The clouds representing density of KOs in each group are shown. The PC1 and PC2 with their variance explained are shown in x and y-axis.

**
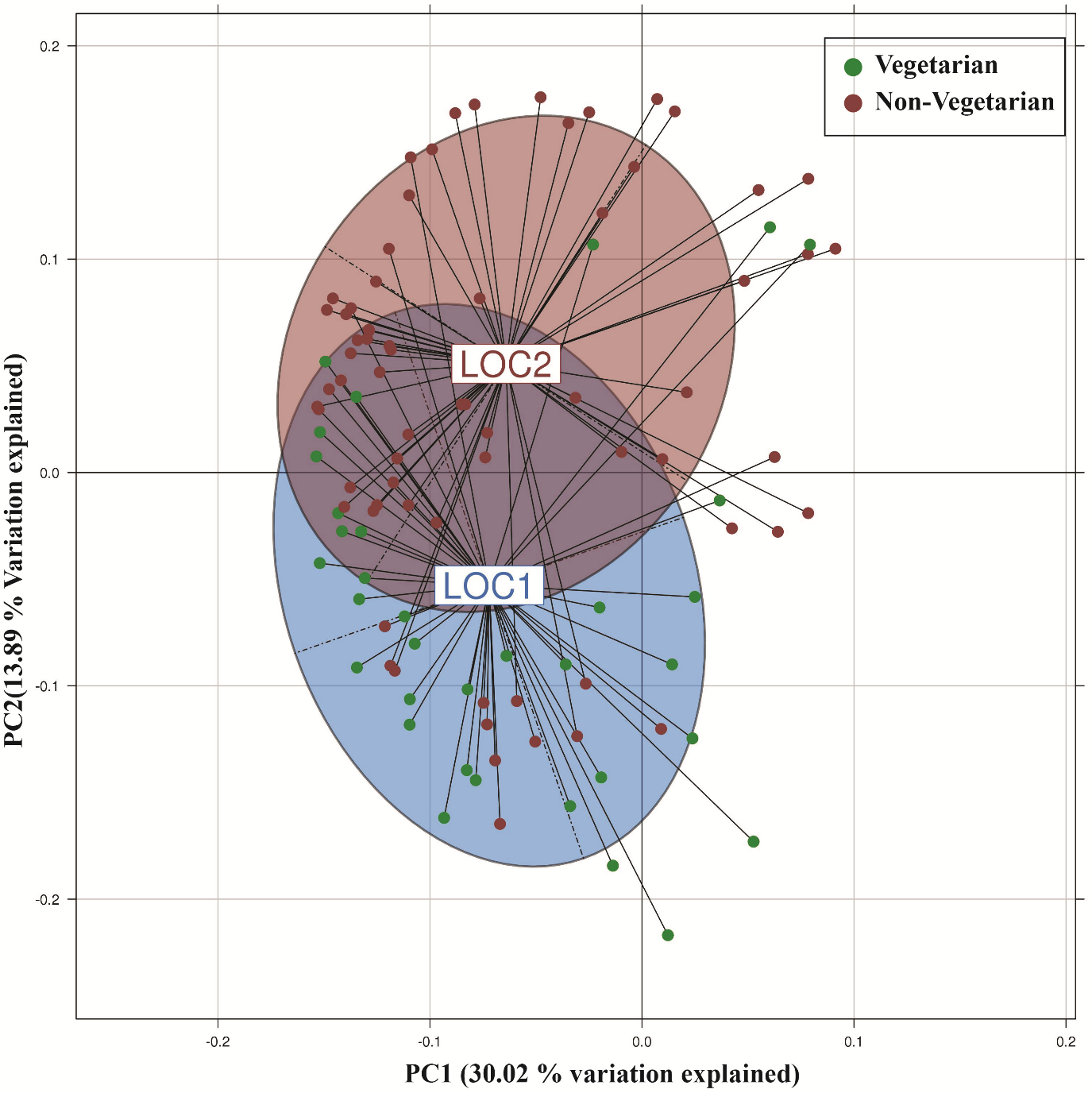
**

**Figure S12. Principal Component Analysis of unweighted UniFrac distances between samples from LOC1 and LOC2.**

PCA analysis of unweighted UniFrac distances of rarefied OTU table (100,000 seqs/sample) from Indian population and their differentiation due to location and diet. Here, the samples are grouped based on their locations (LOC1 and LOC2) and the points show individuals with Non-vegetarian (red) and Vegetarian (green) diet. The top ten principal components tested for correlations with known factors showed location and diet to represent the most significant correlations.

**
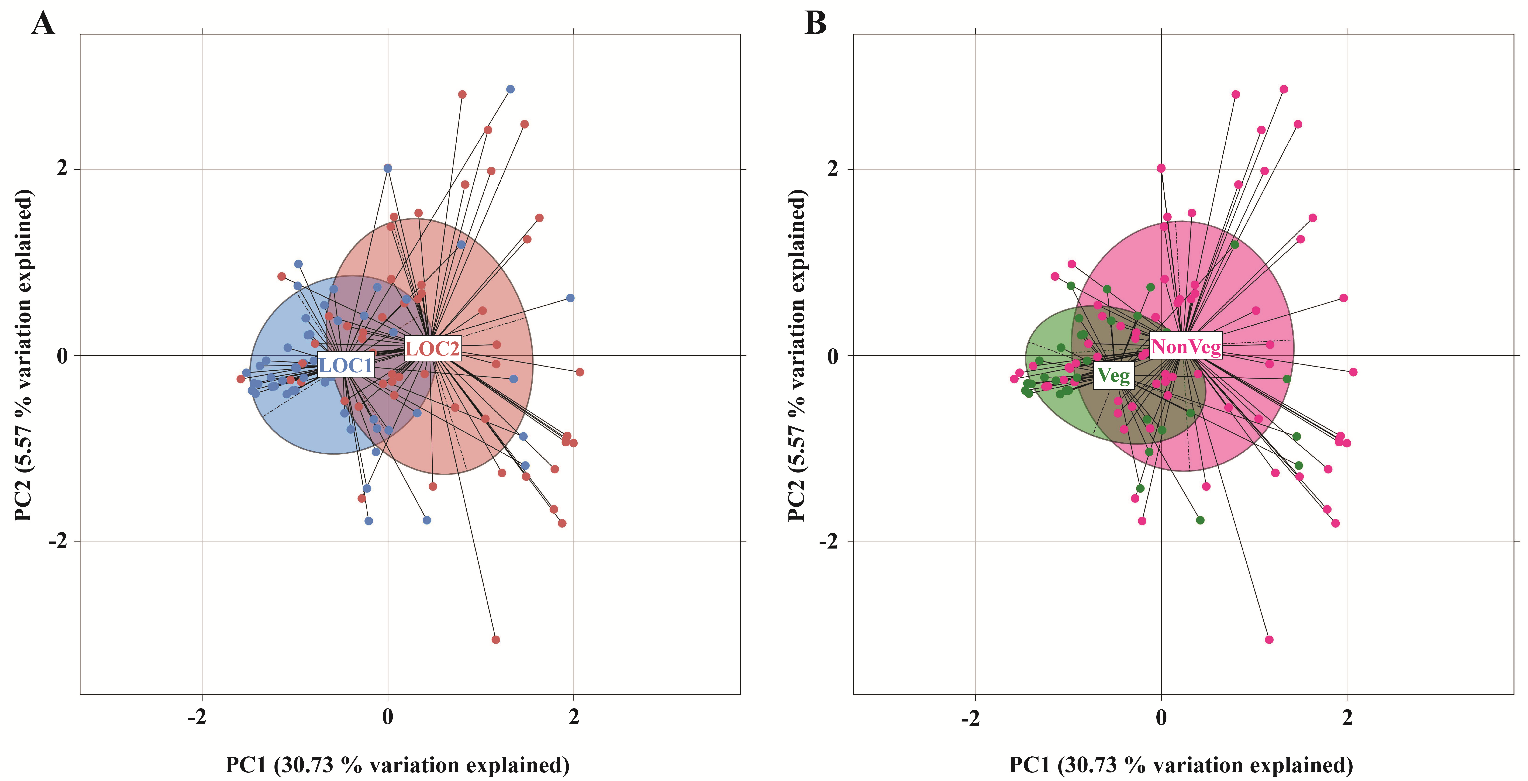
**

**Figure S13. Principal Component analysis of 110 Indian samples using relative abundance of genes and their clustering based on location and diet.**

The ordination of 110 samples performed using relative abundance of genes and their clustering shown using (A) Location and (B) Diet as covariates explaining the maximum variation.

**
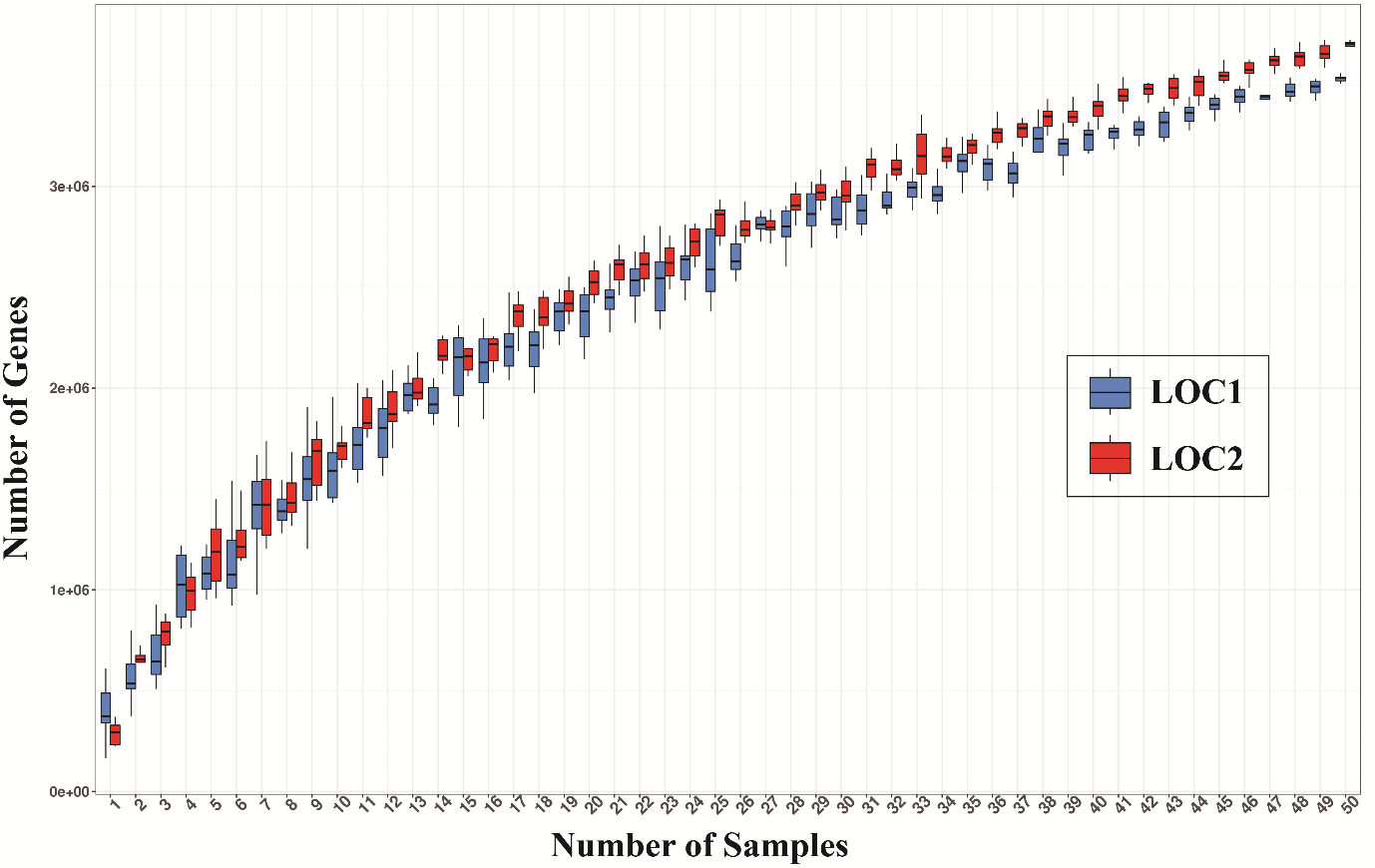
**

**Figure S14. Rarefaction curves of gene count with increasing number of samples in LOC1 and LOC2.** Rarefaction curves showing the gene count with increasing number of samples are shown. Each rarefied curve shows mean gene count calculated from sampling specific number of samples 10 times and their average number of genes from those many samples in LOC1 and LOC2. The red (LOC2) and blue (LOC1) boxes show 75^th^ and 25^th^ percentile (upper and lower quartiles) and the dark line in middle shows median values. The whiskers extending on both sides of the boxes extend 1.5 times IQR.


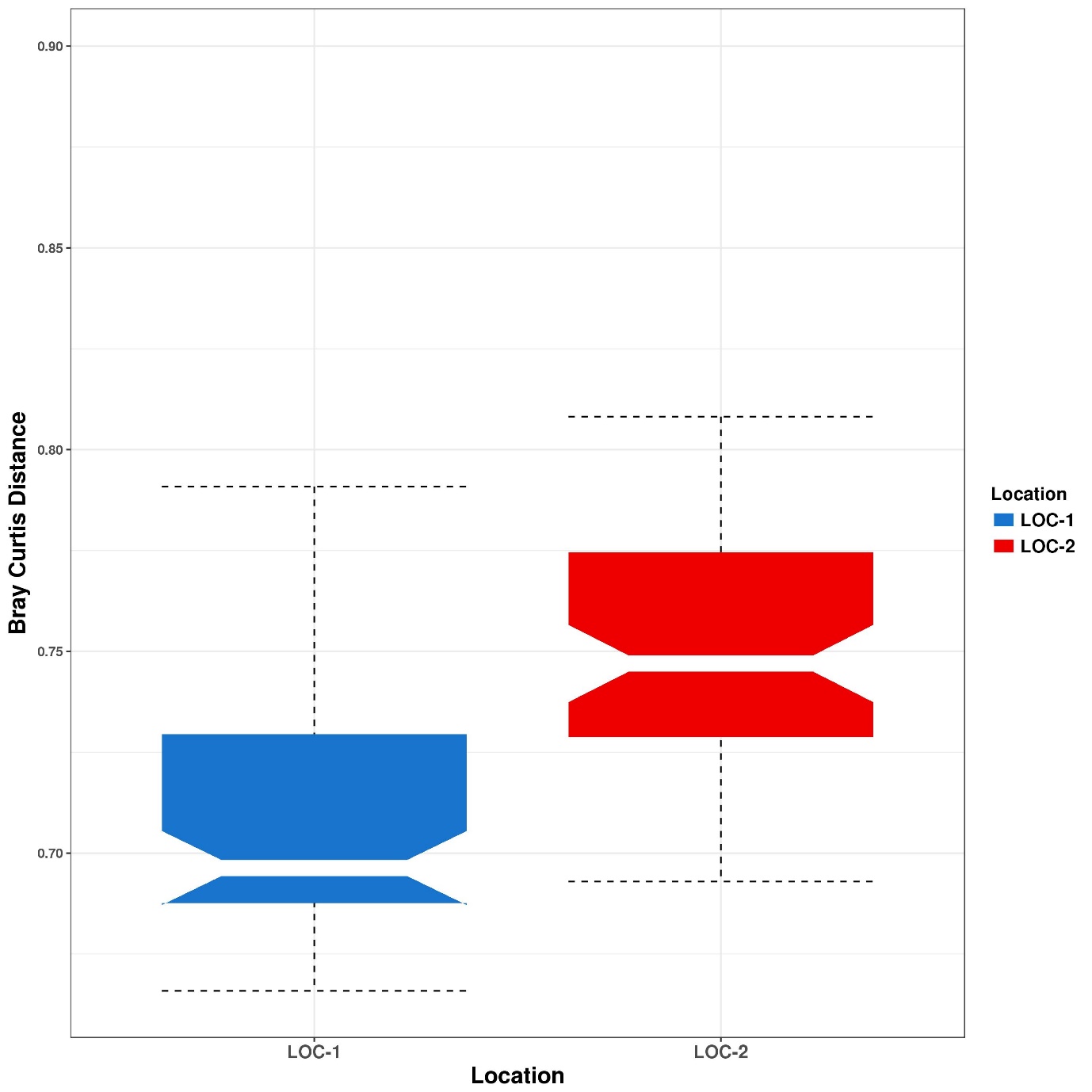


**Figure S15. Inter-sample Bray Curtis distances between LOC1 and LOC2 using gene abundance profiles**

The notched boxplots showing the variation and median values of intersample bray Curtis distances between LOC-1 and LOC-2. The intersample bray custis distances were calculated on variance stabilization based normalized gene abundance tables between samples from LOC-1 with each other and also between samples from LOC2. The boxplots showing the median through gapped notch and the upper and lower quartiles as 75^th^ and 25^th^ percentiles. The whiskers extending 1.5 * IQR (Inter-quartile range) on either side of the boxes shows variation in the data.


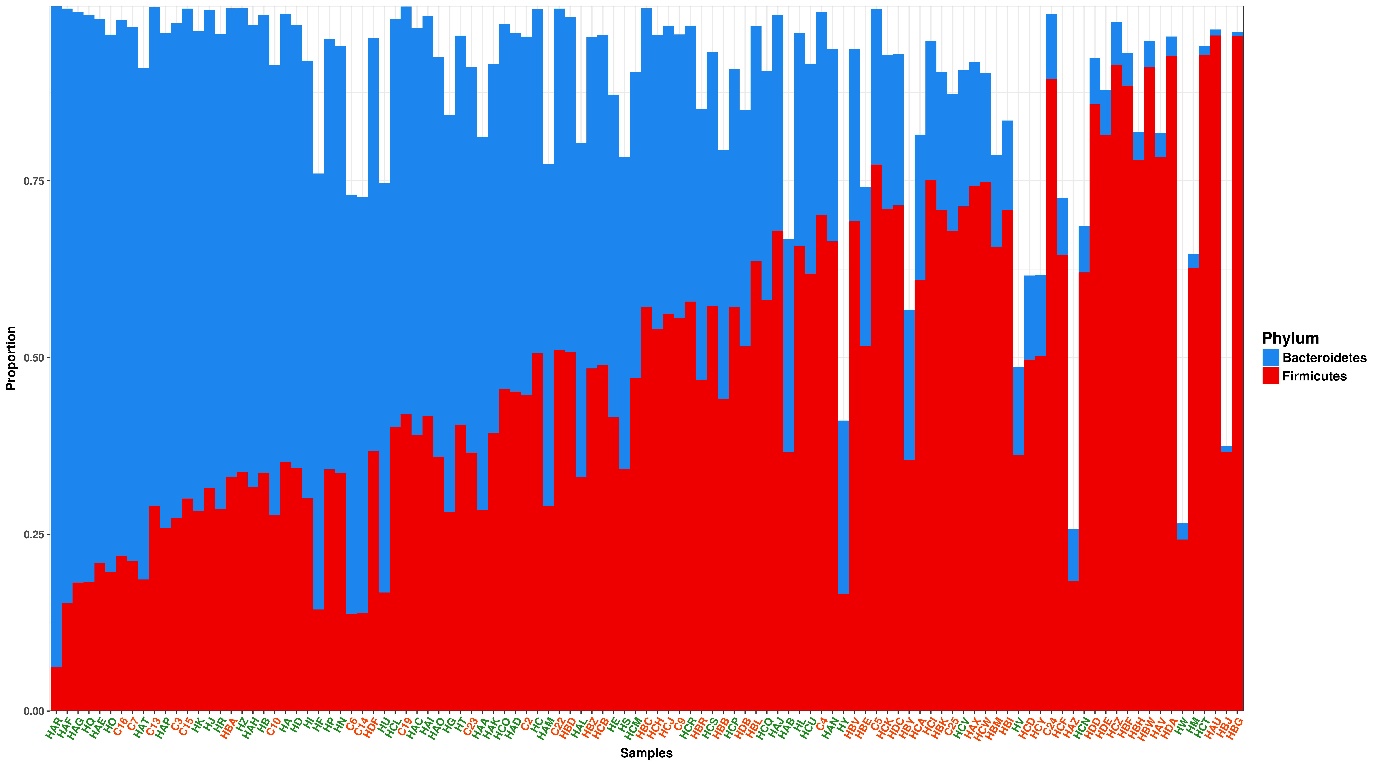


**Figure S16. Bacteroidetes and Firmicutes proportions in Indian Microbiome Samples.** Stacked bar plots showing the relative abundance of Bacteroidetes and Firmicutes in samples from both LOC1 (labelled as green) and LOC2 (labelled as red) in the x-axis.


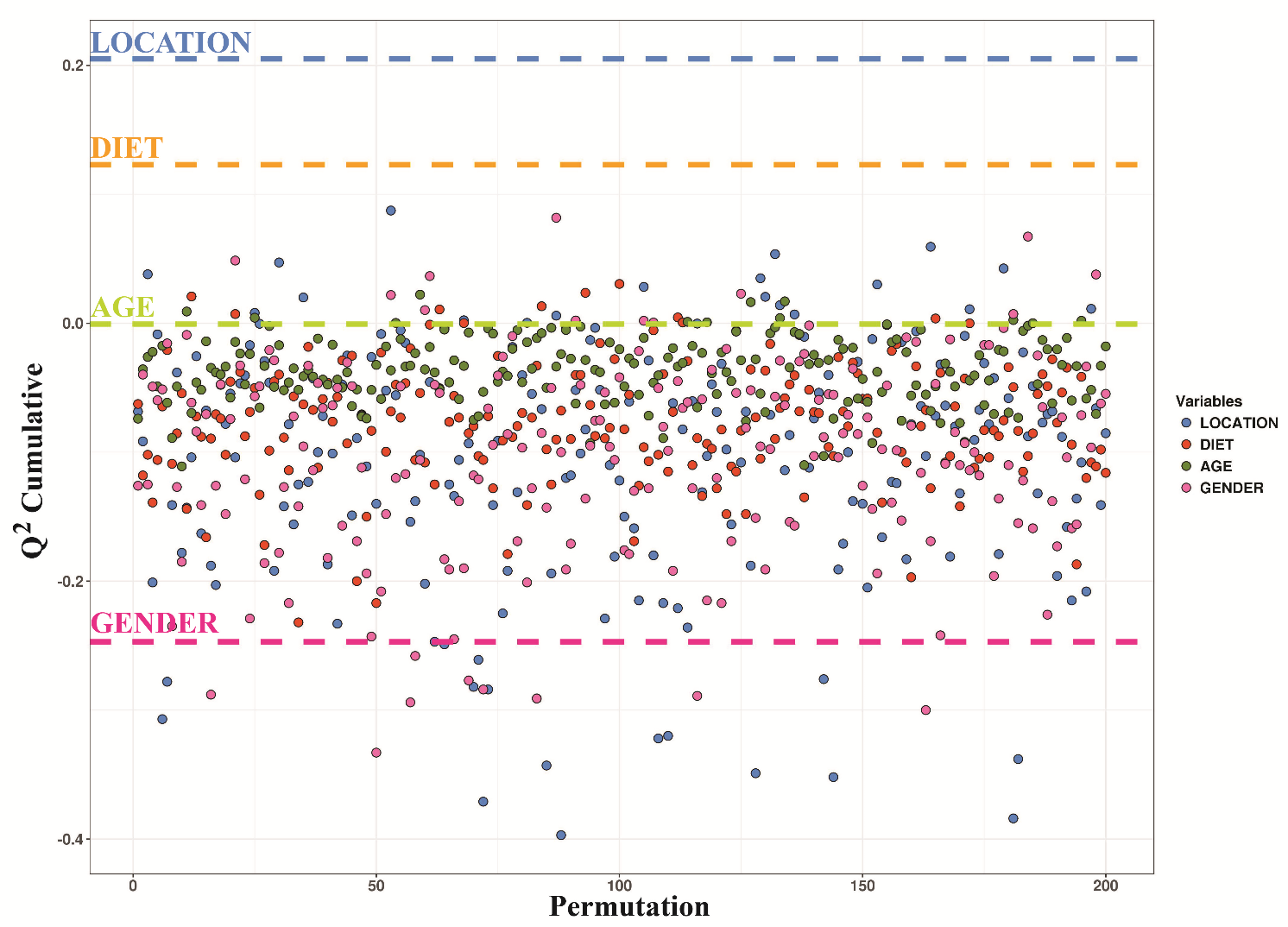


**Figure S17. Validation of OPLS-DA models for different covariates used as classes of separation**

The dotted lines show the Q^2^ values calculated for OPLS-DA models generated using different covariates as classes for separation of samples. The points represent the Q^2^ values calculated from OPLS-DA models generated from samples with randomly permuted labels (n=200). The number of Q^2^ values obtained from models with randomly permuted labels that are above the Q^2^ value from OPLS-DA model with actual label is used validate the model.

**
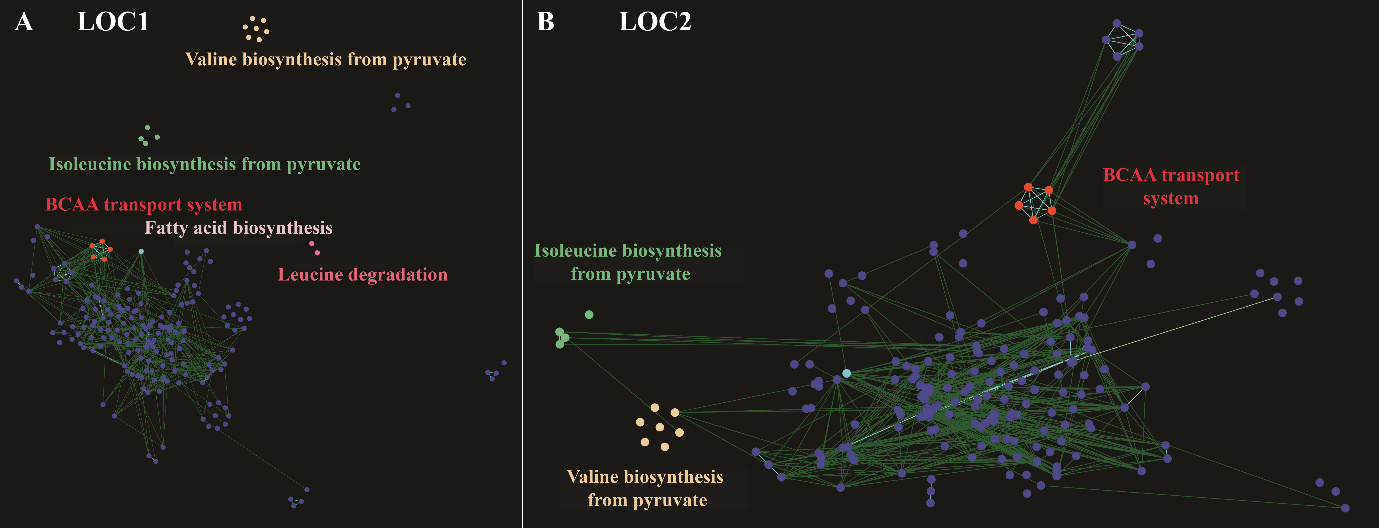
**

**Figure S18.** **Differential correlations reveal differences association of BCAA pathways in LOC1 and LOC2**

Differential correlations between KO modules in **(A)** LOC1 and **(B)** LOC2 which showed significant differences (FDR Adj. P-value <0.05) in Spearman’s correlations are plotted. The KOs within each module are associated with KOs from other modules. The KOs belonging to BCAA metabolism and their correlations with other KOs showed significant (FDR Adj. P-value <0.05) difference between LOC1 and LOC2. The network shows each KO as node and their associations with other KOs as edges. Only significant correlations (Correlation P-value < 0.05) are plotted. The KOs which had positive correlations with other KOs are connected by edges and the network analysis identifies important associations between modules from KO correlations.
