# Supplementary figures and images for: The unique composition of Indian gut microbiome, gene catalogue, and associated fecal metabolome deciphered using multi-omics approaches
Source: Gigascience. 2019 Jan 30;8(3):giz004. doi: 10.1093/gigascience/giz004 (PMC6394208; doi:10.1093/gigascience/giz004)

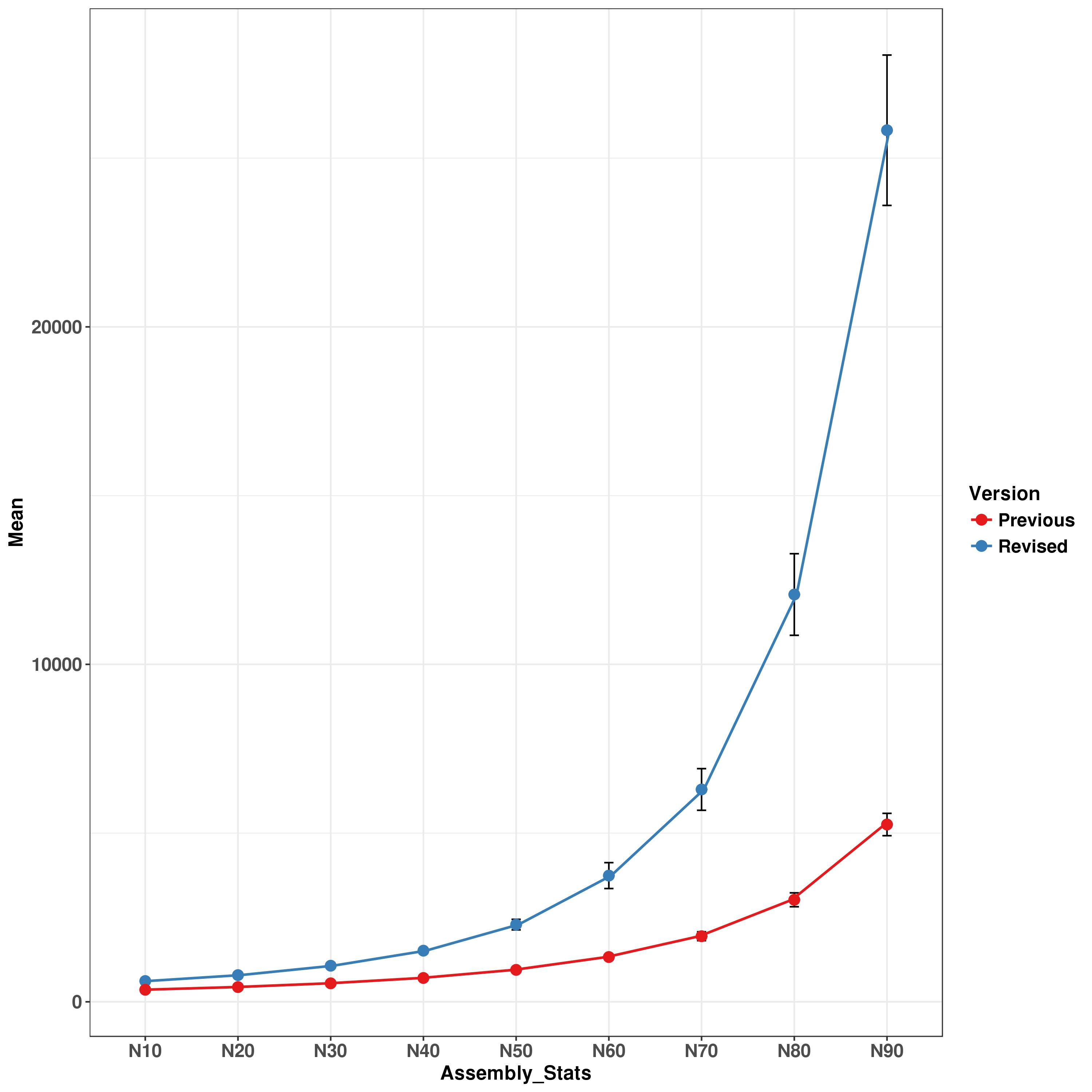

Supplement: Supplemental Files [file giz004_supplemental_files.zip › Supplementary_Figure_1.jpg]
