# Supplementary material for: The unique composition of Indian gut microbiome, gene catalogue, and associated fecal metabolome deciphered using multi-omics approaches
Source: Gigascience. 2019 Jan 30;8(3):giz004. doi: 10.1093/gigascience/giz004 (PMC6394208; doi:10.1093/gigascience/giz004)
Supplement: response_to_reviewer_comments_original_submission.pdf [file giz004_response_to_reviewer_comments_original_submission.pdf]

Author's Response To Reviewer Comments

Close

Replies to Comments -Reviewer 1

The revised manuscript text has been marked in Pink and Orange colours to indicate the changes made as per the suggestions of reviewer 1 and 2, respectively.

--Reviewer #1: The study entitled "Multi-omics analysis reveals Indian gut microbiome variations due to diet and location and its implications on human health" describes an in-depth sequencing and metabolomic analysis of a unique set of samples from two distinct locations in India. The authors correlate bacterial species composition and fecal metabolites in order to draw conclusions about health in the two geographic locations and the link with diet and disease risk. Specifically, the North Central, primarily vegetarian population, consumes a high proportion of high-fat and sugary foods and ranks among the lowest for life-expectancy. This is compared to a Southern location with an omnivorous population with a much higher life expectancy and lower risks of T2D and cardiovascular disease.
The correlation and discussion of specific metabolites and risk factors in the North Indian population versus the Southern population, and the conclusions appears to be supported by the data. The authors concentrate on a limited number of major metabolites, BCAAs and SCFAs, and link these to pathways identified in the bacterial species that are present in the populations. This focused approach is quite effective and the subsequent detailed discussion of P. Copri is very relevant (previous association with rheumatoid arthritis). The importance of bacteria-driven metabolism and its association with vegetarian diets are all interesting points where this study of the Indian population brings news perspectives.
Indeed the uniqueness of the Indian population, an under-sampled population, is a major contribution to the available databases. It is for this reason that I consider the work appropriate for publication with a certain number of minor revisions prior to publication:

Reply: We thank the reviewer for appreciating our work and providing suggestions which really helped in improving the manuscript. We have tried our best to satisfactorily address the comments and have performed all the suggested analysis. Additionally, we have improved the metagenomic assembly of Indian gut microbiome using IDBA-UD assembler (Kuang et al.; GigaScience; 2017: Please see Methods section). The mean N50 values across all samples showed an increase from 946 bp to 2,288 bp, and the total contig size increased from 1.78 Gbp to 3.086 Gbp (Please see Supplementary Figure 1) in the revised assembly. The updated non-redundant gene catalogue for Indian gut microbiome now consists of 1,551,581 genes. The genes from Indian gene catalogue were added to the Integrated Gene Catalogue (IGC) to construct the ‘Updated Integrated Gene Catalogue’ (India+IGC), which now consists of 10,823,291 non-redundant genes. We have updated all the corresponding results as per the revised Updated IGC and the suggestions provided by reviewer.
Reference
Kuang et al.; Connections between the human gut microbiome and gestational diabetes mellitus; GigaScience; 2017; doi 10.1093/gigascience/gix058

General comments:
--Subjects were excluded if there was reported use of antibiotics during the previous month. How was this cutoff determined and was any analysis performed on the cohort to determine if there was any residual effect of antibiotic use (a known issue in India)? This could be as simple as a PCoA plot, using time since last antibiotics exposure as a variable in the 16s diversity analysis.

Reply: We agree with the Reviewer that antibiotic treatment can have residual effects on the gut microbiome and is an important consideration while collecting the samples. A few recent studies have specifically examined these effects, such as the study carried out by Suez et al. demonstrated that a period of 28 days was sufficient for spontaneous recovery of microbiome composition after antibiotic treatment (Please refer Figure 2 of the article [1]). A recent study by Ruixin Liu et al. [2] has also used the same criteria, where the subjects who did not receive any antibiotic treatment for at least one month prior to sample collection were selected (Please refer to Online Methods: ‘Faecal sample collection and DNA extraction’ section of the cited manuscript). Dethlefsen and Relman [3] show that microbiome communities return to their initial state within one week after the end of antibiotic course. However, we agree that the return of microbiome composition to initial state do vary depending on the type of antibiotic used and can be incomplete. We also agree with the Reviewer’s suggestion that a PCoA using time as variable since last antibiotic exposure and estimating its effect would help to identify the effect of treatment on microbiome composition. However, we did not collect this data during the sample collection, and thus could not perform this analysis. Nevertheless, as per the above mentioned studies including the recent ones, we were very careful in recruiting only those volunteers who were not exposed to any antibiotic treatment for over a month.
References
1. Jotham Suez et al; Post-Antibiotic Gut Mucosal Microbiome Reconstitution is Impaired by Probiotics and Improved by Autologous FMT; Cell; 2018; doi:10.1016/j.cell.2018.08.047
2. Ruixin Liu et al; Gut microbiome and serum metabolome alterations in obesity and after weight-loss intervention; Nature Medicine; 2017; doi:10.1038/nm.4358
3. Les Dethlefsen and David Relman; Incomplete recovery and individualised responses of the human distal gut microbiota to repeated antibiotic perturbation; PNAS; 2011; doi:10.1073/pnas.1000087107

--Could the authors please explain their use of Greengenes 13_5? This release dates to 2013. Was SILVA tested?

Reply: We used the Greengenes database because of its wide use in large number of microbiome studies (Yatsunenko et al; Nature; 2011 & Nakayama et al; Sci Rep; 2016) and also in some of our early publications (Maji et al; Environ Microbiol; 2018, Pullikan J et al; Microb Ecol; 2018). We agree with the Reviewer’s suggestion of using ARB SILVA database for taxonomic classification of 16S rRNA gene sequences since the Greengenes database has not been updated after May 2013, which justifies the use of more recently updated SILVA database.
As per Reviewer’s suggestion, we have now repeated the 16S rRNA gene analysis using ARB

SILVA database release 132 (13th December 2017) as reference database for taxonomic annotation. In order to visualize the differences in the results generated from analysis using the two databases, we compared the taxonomies and OTUs generated from the two databases. The Supplementary Table 1 provides details on the percentage of reads assigned at different hierarchical levels using Greengenes and ARB Silva database as reference. There was a marked increase in assignment of OTUs at genus level using ARB SILVA database (95.2%) compared to Greengenes database (54.56%). The increase in the taxonomic annotation was also observed for other population datasets used in the comparison (Supplementary Table 1).
After the reanalysis of 16S rRNA gene data using the annotations from ARB SILVA database, the results have been updated in the revised manuscript in the Results and Figures (please see Figure 1C, Additional File 5: Figure S3, Figure S5 and Figure S10). We observed similar trends with significant improvements in the annotations of OTUs at the genus level.
References
Tanya Yatsunenko et al; Human gut microbiome viewed across age and geography; Nature; 2012; doi:10.1038/nature11053
Jiro Nakayama; Diversity in the gut bacterial community of school-age children in Asia; Nature Scientific Reports; 2015; doi:10.1038/srep08397
Maji A. et al; Gut microbiome contributes to impairment of immunity in pulmonary tuberculosis patients by alteration of butyrate and propionate producers; Environmental Microbiology; 2018; doi:10.1111/1462-2920.14015
Pullikan J. et al; Gut microbial dysbiosis in Indian children with Autism Spectrum Disorders; Microbial Ecology; 2018; doi:10.1007/s00248-018-1176-2

--I am convinced of the utility of the study, despite some of the additional comments below. Therefore, I would request that the raw shotgun metagenomics data also be made available, and not just the assembled contigs as is currently the case. This is extremely important so that future groups can improve on assemblies and annotations as more data is generated from future studies.

Reply: As per the reviewer’s suggestion, we have now released the raw reads data which can be found at NCBI SRA (https://www.ncbi.nlm.nih.gov/sra) with Project ID: PRJNA397112. The assembled contigs, genes and gene catalogue will also be uploaded on the Giga Science ftp server, which can be accessed by any researcher for the future studies.

Specific comments:
--Line 209: "Detection of Enterotypes" The authors use the term 'analysis of enterotypes', referring to Arumugam et al., for the analysis performed in this section and relate the results to those found in the previous study. However the resulting two enterotypes are more accurately, and simply, called clusters, as they are based on two distinct populations in the current study only. This is in contrast to four-country, 22-metagenome analysis performed in Arumugam et al. I would suggest that the terminology be revised. This same type of nomenclature is repeated in line 272: 'metabotype.' I thank that referring to these as clusters is more accurate and more consistent.
It is also present in the discussion (lines 400-401) and methods (699). I would just stress again that two distinct geographical locations which can be statistically separated into two groups, within a single study, does not constitute an enterotype as defined in Arumugam et al. As LOC1

and LOC2 are distinct in this study, factoring this information into clinically relevant models (lines 403-408) does not require a further variable. The analysis and conclusions about the two groups, nevertheless, appear valid.
My suggestion, if the authors wish to use the "enterotype" comparison, would be to explore how this new dataset of 110 individuals fits when combined with that from Arumugam et al. Do the samples still classify into three enterotypes, and what is the distribution across LOC1 and LOC2?

Reply: We agree with the Reviewer's suggestion that the term 'enterotype' should be used when referring to cross national clusters resulting from similarities in microbiome profiles of different populations and their clustering into groups.
We thank the reviewer for the valuable suggestion to compare the Indian samples with that of Arumugam et al., and see if the Indian samples could still be classified into the three enterotypes. Thus, we performed the meta-analysis of 37 samples from the four nations used in Arumugam et al. with our Indian cohort consisting of 110 samples (Please see Figure 3A and Additional File 8). We were able to classify the Indian samples into three enterotypes using genus-level abundance of 110 Indian + 37 samples from four countries (Arumugam et al.). We also identified the distribution of samples from LOC1 and LOC2 in these three enterotypes. We could observe clear differences in representation of samples from India and the other four populations. We could also identify the differences in representation of samples from LOC1 and LOC2 among these enterotypes. We thank the Reviewer for suggesting this analysis, which helped in confirming the previous analysis and results. We have revised the results section 'Line: 246-255' to include the above analysis and have highlighted in pink. We have also revised the terminology from 'enterotypes' to 'clusters' when referring to the clusters using only Indian datasets in all the sections.

--Line 235: 16S Data Analysis
The authors use rarefied reads for downstream analysis. This type of normalization, while useful for calculating UniFrac distances, is no longer accepted as the gold standard for statistical analysis of 16s data. See (McMurdie PJ, Holmes S. Waste not, want not: why rarefying microbiome data is inadmissible. PLoS computational biology. 2014). The authors should explain why they decided to use sub-sampling normalization. How the threshold of 100K was determined?

Reply: We thank the reviewer for this important suggestion on normalizing the 16S rRNA gene counts. Regarding the threshold of 100K, it was a cut-off based on the lowest sequencing depth among all the samples. We agree with the reviewer that the rarefactions method is useful for calculating UniFrac distances, however for comparative analysis it is not the gold standard now, and should be replaced with the methods used in study by McMurdie et al; PLOS Computational biology, as highlighted by the Reviewer. We would like to mention that we did not use rarefaction in any of our statistical analysis or comparisons except for diversity estimations (Alpha and Beta Diversity). For statistical analysis, we used relative abundance of taxa. As per the reviewer's suggestions, we have now revised all the statistical analysis performed using DESeq2 package in R as mentioned in the study (McMurdie et al.) suggested by the Reviewer. The Unifrac analysis has been revised based on OTUs picked using SILVA database (Please see Additional File 5: Figure S11 and Additional File 13).

--The differential analysis performed in relation to clinical data and location (lines 247-255) should be reanalyzed using current normalization methods (e.g. DeSeq2 or edgeR packages exist for R).

Reply: We appreciate and agree with the reviewer’s suggestions on normalization. Earlier, we had calculated relative abundance by normalizing the raw count of each taxon with total number of reads in each sample. However, as per the reviewer’s suggestion we have now re-run all the differential analysis on raw counts at taxonomic level using negative Binomial model based-Wald test in DESeq2. The genera that showed significant difference between Location 1 and Location 2 were plotted (Please see Figure 3B). We also reanalysed the differential species between LOC1 and LOC2 using DESeq2 based normalization on raw abundances of species obtained from mapping of metagenomic reads to the reference genomes (Please see Figure 3C). Further, differential analysis between clusters was also performed using DeSeq2 based normalization on raw counts (Please see Additional File 10). The results and figures have now been updated according to the latest analysis carried out using DESeq2.

--Lines 347-352: The addition of 110 individuals is a major contribution. Yet, I think that the authors would agree, any future metagenomics analysis of the intestinal microbiota, even those focusing on South-Asia populations, would best be accomplished using the IGC + this study's additional database. Analysis would not be performed using this study's catalog alone. Please consider rewording here to accurately present the impact of the study.

Reply: We agree with the reviewer’s suggestion that IGC+ Indian gene catalogue (constructed in this study), referred to as ‘Updated-IGC’, would be more useful as a reference database than the Indian gene catalog alone even when studying the South-Asian populations. Thus, we have now also uploaded the ‘Updated IGC’ at the GigaScience web server. We have also revised the line 421-424 to include these changes.

--Line 561: The authors appear to perform normalization in relation to gene length, probably RPKM. Like 16s analysis, it has been demonstrated that this type of normalization is not the most appropriate for whole genome metagenomics analysis (https://doi.org/10.1186/s12864-016-2386-y). The authors should rerun the analysis to validate that the bacterial species cited in the manuscript remain significant after applying a modern normalization method such as DESeq2 or edgeR. Perhaps other significant species will also be identified.

Reply: We do agree with the Reviewer that the method of normalization can have an impact on the results. As per the reviewer's suggestion, we have now recalculated gene abundance for all the datasets as raw counts instead of normalizing them by gene length, or as proportions. The raw read counts of genes were used for MGWAS analysis and the construction of MGS was performed. The MGS abundance was recalculated, and reanalysed using DESeq2. The P-values obtained were used for further analysis. The differential abundance of MGS between India and other datasets were determined using negative binomial model-based Wald test implemented in DESeq2 for calculating the P-values (Please see Additional File 5: Figure S2, Additional File 6). Moreover, the differential abundance (P-value calculation) of MGS between LOC1 and LOC2 was also determined using DESeq2 based normalization (Please see Additional File 14).

Using the raw abundance, we also re-calculated abundance of EggNOG, KEGG Orthologues (KO) and KEGG Modules and performed differential analysis using NB model based Wald test in DESeq2 (Please see Figure 2C, 2D and Additional File 7, Additional File 12, Additional File 17). We have now revised the manuscript at the above mentioned places to include the revised results.

--Line 603: The reference cited does not describe the canopy-mgs algorithm. The correct reference is Nature Biotechnology volume 32, pages 822-828 (2014); 'Identification and assembly of genomes and genetic elements in complex metagenomic samples without using reference genomes.' This reference also describes MGS (metagenomic species) that the authors refer to (Line 726, and elsewhere in text).

Reply: We thank the Reviewer for pointing out this error. We have now corrected this reference in the manuscript (Line: 650).

Reply to Comments- Reviewer 2

The revised manuscript text has been marked in Pink and Orange colours to indicate the changes made as per the suggestions of reviewer 1 and 2, respectively.

Reviewer #2: # SUMMARY
--In this manuscript Dhakan & Maji et al. report on their multi-omic analyses of 110 healthy individuals from two distinct regions in India. The authors obtained 16S rRNA gene (V3 region) amplicon sequencing data, metagenomic sequencing data, and metabolomic data from volunteers' faecal samples. In addition, metabolomic data from serum samples were obtained. Using the metagenomic sequencing data, the existing Integrated Gene Catalog (IGC) was expanded by adding novel, non-redundant genes derived from the India cohort. This represents an important addition to the IGC, thereby further complementing the global, human gut-derived microbial gene catalog. The authors compared the taxonomic composition (amplicon and metagenomic data) and the functional potential (metagenomic data) of Indian-derived gut samples to samples from earlier studies (China, Denmark, USA) and found the Indian microbiome to be largely distinct. The authors conclude that diet is likely to be a strong factor in this, especially since the eating habits are often strongly conserved according to region. Using the metabolomic data, Dhakan & Maji et al. identified differences in the faecal and serum concentrations according to region.
GENERAL COMMENTS
--Overall, I think that this study nicely complements existing microbiome studies by further expanding gut microbiome characterization to include samples derived from an Indian population and from different diets (plant-based and omnivorous). Moreover, it highlights the importance of complementary omics, here, metabolomics, in the study of host-microbe interactions.

Reply: We thank the reviewer for appreciating our work and providing suggestions which really helped in improving the manuscript. We have tried our best to satisfactorily address the

comments and have performed all the suggested analysis. Additionally, we have improved the metagenomic assembly of Indian gut microbiome using IDBA-UD assembler (Kuang et al.; GigaScience; 2017: Please see Methods section). The mean N50 values across all samples showed an increase from 946 bp to 2,288 bp, and the total contig size increased from 1.78 Gbp to 3.086 Gbp (Please see Supplementary Figure 1) in the revised assembly. The updated non-redundant gene catalogue for Indian gut microbiome now consists of 1,551,581 genes. The genes from Indian gene catalogue were added to the Integrated Gene Catalogue (IGC), to construct the 'Updated Integrated Gene Catalogue' (India+IGC) and now consists of 10,823,291 non-redundant genes. We have updated all the corresponding results as per the revised Updated IGC and the suggestions provided by reviewer.

Reference

Kuang et al.; Connections between the human gut microbiome and gestational diabetes mellitus; GigaScience; 2017; doi 10.1093/gigascience/gix058

--While many of the authors' conclusions are supported by the reported results, I found that some conclusions need to be toned down as there is not sufficient supporting evidence for these conclusions. Please also see my detailed comments.

Reply: We have made our best efforts to address all the comments and have provided below a point-wise reply to the comments and suggestions. We have also revised the Discussion section at several places to tone down the conclusions correlating the impact of microbiome composition on health as suggested by the reviewer.

--The metagenomic sequencing depth in this study is unfortunately not particularly deep, but neither is it shallow. While sequencing depth is always a limiting factor, it is an important factor if the objective is the recovery of novel genetic/genomic information. This needs to be considered when concluding.

Reply: We agree with the reviewer that sequencing depth is a limiting factor in metagenomic studies. In this study, the sequencing depth was not too high (1.5 ± 0.5 Gbp per sample, mean ± standard deviation), compared to the datasets from other microbiome studies (METAHIT: 4.5 Gbp, 100bp reads; Human Microbiome Project: 2.9 Gb, 100bp reads; Qin et al; 2012: 2.61Gbp, 100 bp reads) that were used for comparison with Indian microbiome. However, through a read length of 150bp and a decent paired-end sequencing depth (1.5Gbp) of 110 individuals in this study, we have been able to provide the first insights on the Indian gut microbiome and reveal its unique composition. The increase in sequencing depth certainly would recover more novel genetic information from low abundant microbes which is an important point to consider while making the conclusions. We have now mentioned it in the discussion section and have also considered it while interpreting the results and deriving conclusions (Line: 408-411, 518-520).

References

Qin et a; A human gut microbial gene catalogue established by metagenomic sequencing; Nature; 2010; doi 10.1038/nature08821.

The Human Microbiome Project Consortium; Structure, function and diversity of the healthy human microbiome; Nature; 2012; doi 10.1038/nature11234.

Qin et al; A metagenome-wide association study of gut microbiota in type-2 diabetes; Nature; 2012; doi 10.1038/nature11450.

--Moreover, I found the variation/spread of the samples from the Indian cohort exceptionally large (Fig. 1 B). This might be something the authors could elaborate on.

Reply: We agree with the reviewer that the spread of the samples from the Indian cohort needs to be discussed in the manuscript. The reason for this variation/spread is the higher inter-sample distances between samples from Indian population compared to other populations (Additional File 5: Figure S1). We have now analysed the principal coordinates from PCA in Figure 1B (Please see Additional File 5; Figure S2). The Wilcoxon rank sum test of coordinates at PC1 revealed significant difference between LOC1 and LOC2 coordinates. A plausible reason could to be the dietary differences between LOC2 population (non-vegetarian diet) and LOC1 population (plant-based diet), resulting into significant (FDR Adj. P-value = 0.0013) differences observed in their MGS abundance profiles (Additional File 5: Figure S2). We have now included this analysis and elaborated it in the results (Line: 182-188).

--An experiment which I would have liked to see - I am not saying that it is necessary, though - is an ordination of the 110 samples alone, i.e., not contrasting against samples from other studies but rather within the current study. I would be curious to know if there is substantial separation of samples according to region and/or diet.

Reply: We thank the reviewer for this suggestion and have now performed an ordination of samples based on gene relative abundance table of 110 Indian samples only and observed their separation according to region and diet (Please see Additional File 5: Figure S13). We have also performed polyserial correlation to observe the effect of diet and location on separation of samples using gene abundance (Please see Additional File 13). The location and diet both were observed to be significantly associated (FDR Adj. P< 0.01) with PC1 explaining the maximum variation in the unsupervised clustering of Indian samples (Line: 288-292).

--Finally, I would strongly encourage the authors to be more careful with their conclusions on "the gut microbiome and its functional consequences on human health". The present study did not investigate "non-healthy" individuals from the respective regions. It might very well be that the same or very similar observations would have been made with respect to faecal/serum metabolite levels and correlations to respective microorganisms if "non-healthy" individuals were included

Reply: As suggested by the reviewer, we have revised the discussion and conclusion sections, and have carefully rewritten the interpretations and conclusions related to human health. We have also revised the title of the manuscript as suggested in the later comments.

--The Data Description section should be extended. It should include description of the metabolomic data that was generated as well as of the metadata which was collected (Age, BMI, etc.). Some of this information is provided in the Methods "Study design and subject enrolment" and should be moved to the Data Description instead.

Reply: As per the suggestion, we have now included the description of the metabolomic data,

BMI, age, metadata, study design and subject enrolment in the Data Description section (Line: 109-132). Moreover we have now provided a separate table for data collected for different samples in Additional File 1.

--Instead of reporting "thresholded" p-values (e.g., "P<0.05)"), please report the actual p-values.

Reply: We have replaced the threshold P-values with the actual P-values at most places in the manuscript. However at places such as Line: 317, where multiple species/genes are mentioned we have reported a threshold P-value for considering significant ones.

--I would encourage the authors to include the version and parameters of tools that were used in the Methods.

Reply: We have now included the version and parameters of the tools that were used in the Methods section (Please see Methods section).

--Moreover, it appears that references are occasionally missing, e.g., for the WMW test, FDR-adjustment, Polyserial correlation/biserial correlations, Reporter features algorithm, etc.

Reply: Thanks for pointing it out. We have now added the references for the statistical tests used for the analysis.

--The readability of the manuscript should be further improved, e.g., by involving a professional editing service.

Reply: We have carefully read the manuscript and have made specific efforts to improve the readability. I hope you would find the revised manuscript much improved than the previous version.

My comments below refer to the second row of line numbers, i.e., the one _not_ in typewriter font.
TITLE
--Title: "its implications on human health": It is not clear what the "its" refers to. I would suggest adjusting the title accordingly. Moreover, while it has been shown that diet has an effect on the gut microbiome, I do not know whether "due" is the right wording here. I prefer how the authors phrased it in the abstract, e.g., "showed associations with". I would thus recommend a more careful wording. Moreover, no "non-healthy" individuals were included in the present study, hence making the conclusion of "implications" rather difficult due to lack of supporting evidence (s.a., my general comments)

Reply: We thank the Reviewer for this suggestion. We have revised the title to provide more emphasis on the unique composition of Indian gut microbiome and the functional associations revealed through metabolomics approach. The revised title now reads as “The unique composition of Indian gut microbiome, gene catalogue and associated faecal metabolome

deciphered using multi-omics approaches”. I hope the reviewers would find it more appropriate than the earlier title.

ABSTRACT
--L25: "comprehensively": This could be debated, e.g., at what sequencing depth would one consider to have covered the composition and/or function "comprehensively". Please remove this.

Reply: We have removed the word ‘comprehensive’ from this line. (Line: 25).

--L26: "including 16S rRNA marker gene and shotgun metagenomics": This sounds to me as if the "16S rRNA marker gene" sequencing is also considered "metagenomics", which it is not. I would thus suggest "including 16S rRNA gene amplicon sequencing, metagenomic sequencing, and ...".

Reply: We agree with the reviewer and understand that 16S rRNA marker gene sequencing is not metagenomics. While framing the sentence it appeared as one of the methods for metagenomics, and we thank the reviewer for pointing it out. We have now revised it in the manuscript (Line: 26-27).

--L32: "BCAA": This abbreviation was not introduced before. Same applies to "SCFA in L34". Please adjust accordingly throughout and for all other abbreviations in the manuscript.

Reply: We have now provided the expanded form of all abbreviations at the first instance of their inclusion in the manuscript and have made these changes at all required places (Line: 33, 36, 37).

--L37: "BCAAs were found higher": "higher" in what? I assume in concentration, but this should be clarified in the text.

Reply: Indeed, we were referring to the BCAA concentration, and we have now revised this sentence (Line: 38-40).

--L41: "its functional consequences on human health": I think that this is too strong of a claim here. In particular, this study involved only healthy individuals, hence, while there have been differences observed, these differences may not necessarily have a positive or negative effect, but could be neutral. Put differently, different gut microbiomes may be related to healthy individuals or "non-healthy" individuals might have revealed similar findings.

Reply: We agree with the Reviewer and have revised the sentence (Line: 43-44).

MAIN TEXT
--L63: "constitution": This typically refers to the "the highest laws of a sovereign state, a federated state, a country or other polity." (https://en.wikipedia.org/wiki/Constitution_(disambiguation)). The authors should consider

reformulating this, e.g., by using "condition" or a more appropriate term. Maybe the authors were referring to "composition"? It is not really clear to me, especially with respect to "understanding its variability". It is not just the taxonomic but also the functional composition which has been shown to be of importance. Hence, I would encourage the authors to clarify their point more explicitly here. Finally, this sentence may be misleading as "dysbiosis" is typically used when comparing (at least) one phenotype (e.g., lean) to another (e.g., obese). However, this study is focussed only on one phenotype, i.e., "healthy".

Reply: We agree that the word ‘constitution’ can be replaced with ‘composition’ and have revised this sentence by including all the suggestions made by the reviewer (Lines: 54-55).

--L69: "WGS": This abbreviation was not properly introduced. Please make sure to do so for all abbreviations throughout the manuscript.

Reply: Thank you for this comment. We have now introduced this abbreviation and all other abbreviations in the manuscript at their first usage (Line: 59-60).

--L72: "Branch" -> "Branched".

Reply: We have corrected this word (Line: 62-63).

--L83: I would rephrase "from the major world populations".

Reply: We have rephrased this statement (Line: 74).

--L86: I would rephrase "equally dominated". Typically, "domination" is used when a single entity has a majority stake.

Reply: We have rephrased this word as ‘equal representation’ (Line: 77-78).

--L114: I am not sure if these two locations as well as the total cohort size (n = 110) qualify as being "representative". I would thus suggest to remove the respective wording. Same applies to "comprehensive" , s.a., my respective comment above.

Reply: We agree with the suggestion and have removed the word ‘representative’ and reframed the sentence. (Line: 104-105).

--L115: "16S rRNA sequencing" -> "16S rRNA gene sequencing".

Reply: We have made this change (Line: 105-106).

--L133ff: Was the assembly done on reads from individual samples or on the pooled set of reads? It is not clear as the authors emphasize pooling in the subsequent sentence which reads to me as if this was _not_ done to generate the 1,337,547 contigs. Please clarify.

Reply: We wish to clarify that the assembly was performed on individual samples separately.

The reads were mapped back to the assembled contigs from individual samples and the reads that did not map to the contigs from each sample were pooled from all the samples and a denovo cross assembly was performed using the unmapped reads from all the samples. We have employed a similar strategy for contigs and gene catalogue construction as used in other studies [1]. We have now clearly clarified this point in the revised manuscript (Line: 139-144, 590-592).

References:

Qin et al; A human gut microbial gene catalogue established by metagenomic sequencing; Nature 2011 (see section Metagenomic sequencing of gut microbiomes).

--L139: Please remove "In addition". It sounds as if this is a result from the current paper but it is not.

Reply: We have removed this word and have reframed the sentence. (Line: 146).

--L141: "populations" seems inappropriate here as the HMP and MetaHIT projects both involved multiple populations themselves.

Reply: We agree with the reviewer and have now changed this word to “multiple populations”. (Line: 147- 148)

--L145 + L146: Please specify what the numbers in the brackets with the "plus-minus" mean. Are they representing the standard deviation?

Reply: As correctly pointed out by the reviewer, the ‘plus-minus’ represent standard deviation. We have now added standard deviation in the brackets, for example 69.2% (± 4.01% standard deviation). (Line: 153,155).

--L147f: I am not sure what the authors wanted to say here. Do they mean that reads from _other_ studies were mapped to the original IGC as well as to the updated IGC?

Reply: Here, we had mapped reads from microbiome samples of healthy individuals from three different studies (USA datasets from HMP, Denmark dataset from MetaHIT and Chinese datasets from Qin et al; 2012) on the original IGC and on the updated IGC. We have reframed this statement (Line: 158-162) and the mapping is shown in Fig. 1A. The results have been updated as per the revised gene catalogue.

--L150f: Please rephrase this to reflect that only a _subset_ of the genes of the 110 Indian gut samples in the current study are not represented in other gut microbiome datasets. After all, 718,360 of the 1,479,998 non-redundant genes were added to the original IGC but not the full extent of the current non-redundant genes.

Reply: We thank the Reviewer for this comment. We would like to mention that we aligned the set of non-redundant genes (after removal of redundancy) identified in Indian gut microbiome with the Integrated Gene Catalogue (IGC), and removed the genes sharing ≥90% identity with IGC genes. Thus, the remaining genes from Indian gut microbial gene catalogue which were

unique to the IGC (sharing < 90% identity) were added to generate the updated IGC. As per the revised gene catalogue, 943,395 genes from Indian microbiome samples were added to IGC, thus forming an updated IGC containing only the non-redundant genes from Indian cohort. We have now reframed the sentence (Line: 148-153, 163-164).

--L157: "non-reference" -> "reference-independent".

Reply: We have replaced ‘non-reference’ with ‘reference-independent’ (Line: 171)

--L159: Please remove "higher", it does not seem to fit here.

Reply: We have removed the word ‘higher’ from the position (Line: 175)

--L164: "PCA" stands for "Principal Component Analysis", hence, the second "analysis" in the text is redundant.

Reply: We agree with reviewer and have removed the word ‘analysis’ (Line: 179-180)

--L166: Actually, if the data was projected to PC1, there would be quite some overlap. The separation is actually benefiting from _both_ dimension, PC1 _and_ PC2. I would suggest removing the "at PC1" altogether.

Reply: We agree with the reviewer and have removed ‘at PC1’ from this sentence (Line: 181-182).

--L174: "16S rRNA markers" -> "16S rRNA gene markers".

Reply: We have replaced ‘16S rRNA markers’ with ‘16S rRNA gene markers’ (Line: 198).

--L175f: While, indeed, the amplicon and, to some extent, the metagenomic data suggest members of the Prevotellaceae to be enriched in the present cohort, referring to this family as a marker should be supported by quantitative analyses, e.g., statistical analysis of differences in group means (t-Test or WMW-test) or a classification-based approach (feature selection).

Reply: We thank the Reviewer for this observation and suggesting the need for a statistical analysis to support it. We have now performed a feature selection test using Random Forest analysis (Please see Additional File 5: Figure S4) showing the selection of most important features (mean decrease in accuracy > 0.01; mean relative abundance ≥ 1% in at least one population) and their relative abundance in different populations. The most discriminating features (families) which were able to classify Indian samples from other populations were plotted rank-wise (Additional File 5: Figure S5). The pairwise Wilcoxon rank sum test of important families between India and other populations was performed and represented using box plots (Please see Additional File 5: Figure S6). The analysis has been included in revised manuscript (Line: 199-203).

--L184ff: This paragraph needs to be revised as it currently is hard to read. The sentence in

L193f was especially hard to read and I am still unsure about what "The proportion of essential genes covered by top-ranking nine eggNOG clusters" means: What is the meaning of "nine" in this context when the authors refer to 15,000 to 30,000 eggNOG clusters later.

Reply: We apologize for the typo error. We have removed the word “nine” from this statement. We have also revised this paragraph to make it more readable. Please see the changes made in the paragraph (Line: 215-220).

--L196f: It was not readily clear to me what "alpha diversity (Shannon) calculations using gene abundances" meant and I found the Methods lacking on this point. What gene(s) was/were used ? Moreover, Fig. S4's legend mentions "gene proportions". How does this relate to "gene abundances"? It seems, from the Methods, that rarefaction was used, while the remaining information is scarce on this point. However, this is an important point as the sequencing depth in the current study (mean of 4,545,280 reads/sample) is not particularly deep (cf. Table 1) and, hence, gut microbes' genomes may be covered only partially. In the study by Qin et al . (2010), an order of magnitude more reads per sample ("an average of 62.5 million reads") were produced, albeit at rather short sequencing lengths of 75 bp (compared to 150 bp in the current study).

Reply: We apologise for the lack of clarity in this part. We earlier did not use rarefaction at gene level but the entire gene proportions were used to calculate the diversity. We agree that sequencing depth can have large impact on diversity metrics. We have now used raw gene abundance table which were rarefied at a depth of 1,000,000 seqs/sample for n=30 iterations, and the mean Shannon index were calculated and plotted as box plot (Please see Additional File 5: Figure S9) (Kuang et al.; GigaScience; 2017). We have now included this information in the methods section in revised manuscript (Line: 228-230, 770-772).

--L202: What does "Eigen values, and their scores" mean, i.e., what is a "score" here? Moreover, they are spelled "eigenvalues", i.e., in one word. Please correct throughout.

Reply: We have now revised the statement and also corrected the term ‘eigenvalues’ throughout the text as per the suggestions (Line: 235).

--L203: I am not sure if the authors refer here to "szignificantly" in a statistical sense or not. If so, please include respective quantitive results to support this conclusion.

Reply: As you have rightly mentioned, we were referring to a statistically significant observation, and have now provided the FDR Adjusted P-value in this sentence (Line 236-237).

--L206: How was the odds-ratio computed? In the Methods, the description refers to LOC1 and LOC2, albeit, it seemed, i.e., I was not sure, that a comparison of Indian microbiome vs. "Other" microbiome was intended. If this is the case, the authors should clarify this in the Methods, i.e., that not only was LOC1 compared against LOC2 but also "Indian" vs. "Other" (maybe among other pairwise comparisons).

Reply: The Odds Ratio was computed to obtain the enrichment of species/genes between LOC1 and LOC2 as $OR (k) = [\sum_{s=LOC1} A_{sk} / \sum_{s=LOC1}(\sum_{i \neq k} A_{si})] / [\sum_{s=LOC2} A_{sk} / \sum_{s=LOC2} (\sum_{i \neq k} A_{si})]$, and also for enrichment in Indian microbiome compared to other datasets consisting of USA, Denmark and China referred as “OTHERS” : $OR (k) = ([\sum_{s=INDIA} A_{sk} / \sum_{s=INDIA}(\sum_{i \neq k} A_{si})] / [\sum_{s=OTHERS} A_{sk} / \sum_{s=OTHERS} (\sum_{i \neq k} A_{si})])$. We have now provided the details of comparison performed in the Methods section (Line: 809-812).

--L216ff: I welcome the careful wording chosen by the authors here. It appears that there is no detailed dietary information available which could have been used to further support the authors' hypothesis, but they might want to highlight this as a window of opportunity for future study, i.e., including something like a food-frequency questionaire to be able to quantitatively assess possible links to diet.

Reply: We thank the reviewer for this suggestion. This is an important point and we have now included it in the revised manuscript (Line: 268-270).

--L227: Could the authors please elaborate on how the "Spearman's correlation coefficient" was used in this context? I would have applied Fisher's exact test here.

Reply: As suggested by the Reviewer, we have now used Fisher’s exact test here. Earlier, the Spearman’s correlations were applied to identify the correlation between KO based and Genus based cluster allocation. Using Fisher’s exact test, we found no differences between Genus level and KO level clustering (Fisher’s exact P-value = 0.6843) in the samples assignment (Line: 275). We have provided the file containing details of cluster allocation for each sample (Please see Additional File 11).

--L235: "16S rRNA" -> "16S rRNA gene"

Reply: We have replaced 16S rRNA with 16S rRNA gene at all the places in revised manuscript.

--L236: The term "PCA" has been used previously, so this is not the place to introduce the abbreviation.

Reply: We agree and have now removed this term (Line: 284-285).

--L240: It was not clear to me if "taxonomic and functional diversity" were combined here or not. However, this is important to clarify as taxonomy and function are only partially linked.

Reply: We agree with the Reviewer that taxonomic and functional diversity are only partially linked. We understand that the text could have led to this confusion. We have now revised the text in manuscript and hope that it would read fine now (Line: 292-293).

--L255: Is this analysis based on amplicon or based on metagenomic sequencing data? L247 indicates the former, while MGS/CAGs are defined based on the latter. Please clarify in the text.

Reply: The results mentioned in line number 300-302 were based on amplicon sequencing data analysis using Phylum abundance, whereas the results in lines 305-314 are based on taxonomic species identified from metagenomic sequencing data using reads mapped to reference genomes. The results in line 314-320 are based on the MGS analysis from clustering of gene abundance profiles. We apologize for this confusion. We have now provided this information in the revised manuscript.

--L260: Please list "the two species".

Reply: We apologize for the confusion. We were referring to the two species mentioned in the previous line. We have now revised the sentence to clearly refer to the above-mentioned two species (Line: 320-321).

--L262: Isn't "high fiber-rich" redundant? I.e., either "diet high in fiber" or "fiber-rich diet".

Reply: We agree with the Reviewer and we have now changed this word to fibre-rich diet (Line: 323)

--L274: The conclusion drawn by the authors about the OPLS-DA results is misleading, s.a., https://www.ncbi.nlm.nih.gov/pmc/articles/PMC4990351/. Specifically, the OPLS-DA model integrates the class information with the aim to _increase_ the between-class separation. Hence, the separation observed in Fig. 4C may (partially) be a consequence of the method used and not of actual separation being in the data. An unsupervised method should be used to check for the presence of meaningful separation followed by a supervised method to perform quantitative evaluation, e.g., PERMANOVA, to check how much of the variance is explained by the respective covariates.

Reply: We agree with the reviewer that OPLS-DA model integrates class information (in this case location) and increases the between class separation. As per the reviewer's suggestion, in addition to OPLS-DA, we have now performed PERMANOVA on metabolite abundance table to assess the effect of covariates and identify the ones which explain maximum variation. We have now included the results of PERMANOVA in the manuscript (Please see Table 2). Moreover OPLS-DA models using class information for each of the covariates were used to calculate model Q2 which assesses the quality of the measurement for each of the covariate (Please see Table 3). Since invalid models can still produce higher Q2 values due to over-fitting, the class labels were randomly permuted for n=200 iterations and distribution of Q2 values were produced to assess the reliability of the Q2 values. The reliable model should yield significantly higher Q2 values compared to Q2 values generated from models with randomly permuted labels (Please see Additional File 5: Figure S17). Moreover, an unsupervised clustering of metabolite abundance is already performed (Please see Figure 4A), and its polyserial/biserial correlation with different covariates identified PC1 to be correlated with location, and PC2 with the diet (Line: 340-348).

--L298f: I am not sure if I understood the authors' point right here. "result of its inward transport in microbial cells by the BCAA transporters, thus leading to their accumulation in the colon

lumen": Do the authors' mean "uptake by the bacteria, i.e., transport into the microbial cell"? If so, I would not expect an accumulation in the lumen as such.

Reply: We apologize for this confusion. We meant “faecal samples” here and not ‘colon lumen’. We have revised this text appropriately in the manuscript (Line: 364-365)

--L305: Where do the authors show this comparison (serum vs. faeces)? Fig. 6A compares Valine and Isoleucine in LOC1 samples and LOC2 samples, but not serum vs. faeces.

Reply: We have now modified figure 6A showing the comparison of BCAA levels in feaces vs serum (Please see revised Fig. 6A)

--L328: "the major pathway utilized by this species for BCAA biosynthesis": I am not sure in how much the metagenomic and metabolomic data in this study allow to draw this statement. Metatranscriptomic and metaproteomic data would likely be needed here. I would thus suggest that the authors qualify/nuance this statement.

Reply: We agree with the reviewer. We have revised this text appropriately mentioning the result rather than drawing any conclusion in the manuscript (Line: 391-395).

--L375ff: The average age of the cohort is rather low (mean of 29.72 years). Age, however, is an important factor for rheumatoid arthritis. Hence, "A probable explanation" could be toned down to "One aspect to this could be ...".

Reply: We thank the Reviewer for this suggestion. We have now revised this statement accordingly (Line: 446-448)

--L419: "isoluecine" -> "isoleucine".

Reply: We have corrected this word (Line: 488).

--L439f: The second part of the sentence is redundant with the first part and could be removed, or vice versa.

Reply: We have now removed the redundant part from this sentence (Line 508-510).

--L459 - 460: "which appears promising in reducing the metabolic risk factors originating through the interactions between diet and gut microbes to maintain a healthy gut flora": This reads misleading as the "diet" was binary, i.e., "vegetarian" vs. omnivorous" and such a statement likely requires for more fine-grained and specialized studies than were performed in this work. Please adjust accordingly.

Reply: We agree with the reviewer. We have now revised this statement and have toned down the general interpretations at various places in the Discussion section (Line: 512-514).

--L463ff: This entire paragraph reads redundant with the remainder of the Discussion and

should thus be removed or substantially shortened.

Reply: We agree with the reviewer. We have now substantially shortened and revised this paragraph in the manuscript (Line: 515-520).

--L599: "non-reference" -> "reference-independent".

Reply: We have corrected this word (Line: 647).

--L610: Could the authors please, in analogy to their HMP+NCBI results, report how many of the remaining genes aligned to UNIREF?

Reply: In total, out of 10,839,539 genes present in the Updated gene catalogue, 2,773,591 genes were taxonomically annotated using NCBI + HMP reference genomes at nucleotide level. The remaining 8,049,540 genes were aligned against UNIREF database, and a total of 4,553,299 genes (56.56%) could be assigned with a taxonomic annotation. We have now mentioned this information in Methods section (Line: 656-660).

--L611f: This sentence should be rephrased.

Reply: We have now rephrased this sentence (Line: 660-662)

--L706f: How was this assessed and where can the interested reader find the results for this statement?

Reply: We have provided results of CHI index and prediction strength in Additional File 9 with the values. The information about these metrics is provided in Methods section (Line: 754-759).

--L709ff: It is not clear how the "Between class analysis" was peformed. The authors should provide the respective details, e.g., which test, implementation etc.

Reply: Between Class Analysis was performed to support the clustering and to identify the drivers of these clusters. The between class analysis is a type of principal component analysis with instrumental variables. As in this case, ‘Location’ is a variable for the separation between LOC1 and LOC2 within India, and “population” for separation between India and other datasets (USA, Denmark and China). It is a supervised projection of data where the distance between predefined classes (example clusters/location) is maximised. We have provided a clear explanation in the manuscript (Line: 761-767)

--L720: Does "geography" refer to "location" (LOC1 or LOC2) here?

Reply: As correctly pointed out by the reviewer, we meant the two locations (LOC1 and LOC2), and have changed the word ‘geography’ with ‘location’ throughout the manuscript (Line: 775)

--L732: Why was the negative correlation not considered?

Reply: We wish to mention that in this analysis, the objective was to observe the positive association and link them in a network plot. Hence, the negative correlations were not considered. Moreover, plotting negative correlations was not possible in the plot using igraph package in R.

METHODS

--L485: Do you mean the respective table in "Additional_file_1.doc"? Not sure whether this is under the control of the authors, but it should be checked in the proof that the information is consistently named and can be readily found.

Reply: We apologize for this error. We have now changed the name ‘Supplementary Table’ to ‘Additional File 1’ in the revised manuscript. We hope that it could now be easily found.

--L507: "16S rRNA" -> "16S rRNA gene"

Reply: We have corrected this word at all places in the manuscript.

--L534: "phylogenetic distances between reads": Not sure, but did the authors mean "phylogenetic distances between the samples" here?

Reply: The phylogenetic distances were used to calculate Unifrac distances between the samples. The reads used here are the representative sequences from each OTU. Thus, the phylogenetic distances were calculated between each OTU using the representative sequences from OTUs. Using these phylogenetic distances, we calculated Unifrac distances between samples. We have now revised this sentence in manuscript (Line: 578-580, 772-774).

--L539f: How were host-origin reads identified? Which tool, version, and parameters?

Reply: Human reads were identified and removed from each sample using 18mer matches parameter in Best Match Tagger (BMTagger) version 3.101 (http://casbioinfo.cas.unt.edu/sop/mediawiki/index.php/Bmtagger). We have now mentioned this information in methods section (Line: 584-586).

--L561ff: This is probably for the formal proofs, but I would strongly encourage to properly format here as it seems that, e.g, "bi" is supposed to read "b subscript i".

Reply: Thanks for bringing it to our notice. We have now formatted the formula (Line: 610)

--L1037ff: Please check whether "<" and "> are used correctly here." Typically "p < 0.05 is " considered significant and _not_ "P-value>0.05".

Reply: The ‘>’ and ‘<’ are correctly used in Figures 2c, 2d and S3. We used $P > 0.05$ to show the non-significant dots plotted in ‘Red’ colour. The significant ones are shown in ‘Blue’ colour. We have now mentioned it in the figure legend (Line: 1112-1113).

TABLES
--I do not know whether the information provided in Table 2 necessitates a separate table. I leave this up to the authors to decide and to potentially discus this with the journal.

Reply: We have now removed this table from the manuscript and included PERMANOVA table as Table 2, which was also suggested by the reviewer in an earlier comment. Also, we have now provided Table 3 showing validation of OPLSDA models for each of covariate by generating a distribution of Q2 values from random permutation (n=200) of labels and evaluating the number of Q2 above the model Q2 for each covariate.

FIGURES
--5: "Logs-Odd Ratio" -> "Log-Odds Ratio"

Reply: Thanks for pointing out this typo. We have corrected it in Figure 5.

--S6: The labels on the x-axis and y-axis were not readable. Please adjust accordingly. Moreover, I am not sure in how much the "clouds" add value here. They are not further discussed in the text and, hence, could be omitted for clarity.

Reply: The font-size of labels has been increased and we hope that it would be easily readable now. The clouds show the density of the unique KOs in the two groups. It has now been mentioned in the legends of this figure. The blue cloud represents the local density estimated from the coordinates of orthologous groups (KO).

LEGENDS
--Throughout: Please verify correct use of "16S rRNA" and "16S rRNA gene".

Reply: We have now changed 16S rRNA to 16S rRNA gene at all places throughout the manuscript.

--L1015: "MWAS": Shouldn't this be "MGWAS"?

Reply: Thank you for pointing this type. We have corrected it in the figure legend and also at all places in the manuscript.

--L1027: What does "Eigen values and their scores" mean, i.e., what is a "score" here?

Reply: The word ‘score’ has been removed, and ‘Eigen value’ have been replaced with ‘eigenvalue’ at all places in manuscript.

--L1092ff: This reads more like a discussion/conclusion and I would thus suggest to remove this from the figure legend.

Reply: The figure legend of Figure 7 has been revised as per the suggestion (Line: 1162-1164).

Close
