# Supplementary material for: The unique composition of Indian gut microbiome, gene catalogue, and associated fecal metabolome deciphered using multi-omics approaches
Source: Gigascience. 2019 Jan 30;8(3):giz004. doi: 10.1093/gigascience/giz004 (PMC6394208; doi:10.1093/gigascience/giz004)
Supplement: response_to_reviewer_comments_revision_1.pdf [file giz004_response_to_reviewer_comments_revision_1.pdf]

Author's Response To Reviewer Comments

Close

--Reviewer #1

--I commend the authors on their openness and responsiveness to the comments from both reviewers. The additional analyses performed and the clarifications in the manuscript have resulted in a much-improved draft. The availability of both the raw amplicon and WGS data in NCBI's Sequence Read Archive is a great service to the scientific community and should ensure open access to this data and its inclusion in future studies. The origin of the samples and the sequencing data is well-documented and cohort sample collection and storage appears to adhere to ethical and technical standards.

--More specifically, the updated manuscript addresses and corrects all points that I raised in the first review. These include a major reanalysis of 16S data with an updated database (SILVA December 2017 versus GreenGenes 2013) and an implementation of statistical analysis with normalization performed using DESeq2. They clarify their use of downsized data for Unifrac analysis. Further, the authors now combine their data and run downstream analysis using an "Updated-IGC." This clearly aids their analysis and broadens the appeal of the manuscript as a whole. The 9% of additional genes appears to be unique to the Indian cohort. The authors also performed the suggested enterotype comparisons with the data from Arumugam et al.

--Based on this new version of the manuscript, my recommendation is the manuscript can be accepted without further scientific revision. The authors should, nevertheless, have a careful review of the text to address remaining grammatical errors and awkward phrasing. A few, non-exhaustive, examples are given:

The changes suggested by reviewers have been highlighted (orange coloured) in the manuscript.

Reply: We thank the reviewer for appreciating our efforts and recommending the manuscript for publication. We have carefully read the manuscript for any grammatical errors and have also made all the suggested changes in the manuscript text.

--Line 114: Change "All the recruited individuals" to "Recruited individuals"

Reply: As per reviewer’s suggestion, we have made this change (Line: 114).

--Line 216: Should be "relative abundances"

Reply: We have corrected these words (Line: 217).

--Line 365: "its inward transport in microbial cells by the BCAA transporters" would be better as "its uptake by microbes via BCAA transporters"

Reply: We agree with the rephrasing and have revised this sentence as suggested (Line: 369).

--Line 408: Change "Though, the sequencing depth in the study was not too high..." to "Although sequencing depth was modest….longer paired-ends reads, from the cohort of 110 individuals appears sufficient to provide the first insights on the Indian gut microbiome"

Reply: We have revised the sentence as per the suggestions from both reviewers (Line: 412-415).

--Line 446: "One aspect to this could" could be better written as "One potential explanation could be…"

Reply: We have revised this sentence as per the suggestion (Line: 450)

--Line 486: "has known health benefits..." might be better as "has been reported to be beneficial by preventing…."

Reply: We have revised this sentence as per the reviewer's suggestion (Line: 490).

--Line 505: "are emerging, which results in the increased…." is better as "are emerging, with results showing increased….

Reply: We have revised this sentence as per the reviewer's suggestion (Line: 509).

In addition, we have also carefully checked the manuscript for any grammatical or phrasing errors and hope that the revised manuscript is much better in reading.

Reviewer #2

--The authors have reasonably addressed the comments I raised in the original submission. Only one general comment and a few minor comments remain, which should all be readily addressable by the authors.

General comments:
--L286-291: It would be good to test whether the location and diet are correlated and to which extent. In fact, given the information from the authors, I would expect them to be correlated. Hence, the observed results (Fig. S12) are to be expected and this should be qualified. If no such test is performed, I would recommend to at least reemphasize the (strong) influence of location on the diet of the studied Indian populations. This is also important with respect to the results in L333-335.

The changes suggested by reviewers have been highlighted (orange coloured) in the manuscript

Reply: We thank the reviewer for the suggestion. We have now performed a correlation of

location and diet across all samples and observed a high correlation (ρ= 0.708; FDR Adj. P-value = 2*10-16). We have included these results at both places in the revised manuscript (Line: 293-294, 337-338).

Minor comments:
--Throughout: Frequently, "the"/"a" is missing, e.g., L158 "analysis of microbiome", L159 "reads from other three datasets", L163 "This shows that the addition of subset".

Reply: As suggested by the Reviewer, we have added the/a in the manuscript. We have also carefully checked and corrected the manuscript for any such errors.

--L149-150 - "and unique to IGC": This reads as if the 943,395 genes are unique to the IGC, but aren't his unique to the newly constructed Indian microbial gene catalogue?

Reply: We agree with the reviewer that these 943,395 genes identified from Indian gut microbiome are unique to Indian microbial gene catalogue and not present in IGC. We have rephrased this sentence for clarity (Line 149-150).

--L161 - "did not show a significant (P< 0.01)": Not sure if the signficance level (alpha = 0.01) is meant here or if the p-value was "< 0.01". In the latter case, it woud be considered significant at alpha = 0.01. Please clarify and verify throughout.

Reply: We apologize for this confusion. We have now provided the exact P-values for HMP, MetaHIT and China datasets, and the P-values were not significant for all the three datasets as found using the student's t-test, whereas it was significant for Indian dataset. The P-values are mentioned at all places in the manuscript where the results were significant (Line 155-156, 161-162, 166).

--L212-214: Species names should be italicized.

Reply: We have made this correction (Line: 213-215).

--L270: "be" is missing -> "needs to be collected".

Reply: We have corrected this sentence (Line: 271).

--L275: The text suggests a "significance", yet the p-value is listed as 0.6841. Please clarify.

Reply: We apologize for this confusion. We have removed the word "significance" and have rephrased the sentence for clarity. Here, we observed a high concordance in allocation of samples to clusters using both taxonomic (genus abundance) and functional (KEGG abundance) information. Using Fisher's exact test as suggested by reviewer 2 during the earlier revision, no significant difference was observed in cluster allocation (P-value =0.6841) thus showing similarity in clustering of samples using taxonomic and functional information. We have also provided this information of cluster allocation in Additional File 11. (Line: 274-277).

--Supplements: Fig S11 still contains a reference to "enterotypes" which, as suggested by Reviewer 1 (and I agree) should be generally avoided, unless in combination with the non-Indian populations. Please check this throughout.

Reply: We agree with the reviewer and we have replaced the word ‘enterotypes’ with “clusters” in Additional File 5.

--L304-305: This is not a necessity for the revision, but rather a question out of curiosity: Was an association with age tested here, in addition to BMI?

Reply: We had examined the association of multiple covariates including age and BMI, with taxonomic and functional data. We have provided the details of these associations in Additional File 13 and Additional File 15, which were also provided with the earlier submitted manuscript.

--L317 + L319: What do "19 MGS/CAG" and "67 MGS/CAG" refer to here? Are these the numbers of MGSs/CAGs that were annotated to likely be P. copri populations, i.e., multiple strains/sub-species of P. copri were identified? Please clarify this.

Reply: Here, we were referring to the total 19 MGS/CAGs found enriched in LOC1, and 67 MGS/CAGs found enriched in LOC2. We have reframed this sentence for clarity (Line: 317-322).

--L339: Did Cluster-2 show *no* association with location, i.e, was a mixture of samples from LOC1 and LOC2?
Reply: Cluster-2 did show an association with location. Out of a total of 36 samples assigned to Cluster-2 it included 13 samples from LOC-1 and 23 samples from LOC-2. We have mentioned this in the manuscript (Line: 343-344).

--Legend Fig.S17: "OPLD-DA" -> "OPLS-DA "

Reply: We have corrected this word in the legend of Fig. S17 (Additional File 5).

--Fig.S18: Panel A is rather small and the fonts are hard to read. Please increase the size of the panel.

Reply: We have now increased the font size of Panel A in Figure S18.

--L409-411: I welcome the qualification of the sequencing depth here. Nevertheless, the argument of 2x150bp sequencing is misleading here. Read-length clearly plays a role, so does the overall sequencing depth. While 2x150bp is commonly used currently, and hence the current study is up-to-date, I would suggest the authors to rephrase this slightly. My suggestion would be: "... deviation), the inclusion of 110 individuals from two distinct geographic locations as well as the identification of Indian gut microbiome-specific genes provide a first insight into the Indian gut microbiome and are thus considered important additions to the field."

Reply: We thank the reviewer for this suggestion, and have revised this text as per the suggestion (Line: 412-415).

--L411-413: This sentence reads contradictory in itself. If there is a high diversity, how can (only) two locations be considered representative? I would suggest to rephrase this.

Reply: We have removed the word ‘representative’ and have rephrased this sentence (Line: 415-417).

--L431: It is not readily clear what "Its" refers to here. I assume it is "Prevotella", yet this should be clarified.

Reply: Yes, the word ‘its’ was referring to Prevotella. We have reframed this statement for more clarity (Line: 435).

--L439: Please consider removing "driver" unless you can show a causation rather than the association which was presented in the results.

Reply: As per the suggestion, we have removed the word ‘driver’ from the sentence (Line: 443).

--L442: "bacteria" -> "bacterium"

Reply: We have corrected this word (Line: 446).

--L470-471: The "statistically sound" is not readily clear here. Please consider removing this as I do not find it relevant in this context.

Reply: As per the reviewer’s suggestion, we have revised this sentence and have removed the phrase ‘statistically sound’ (Line: 474).

--L500: "Firmicute" -> "Firmicutes"

Reply: We have corrected this word (Line: 504).

--L515: Please remove "populations", it does not fit in here.

Reply: We have removed this word (Line: 519).

--L578: Please check correct capitalization.

Reply: We thank the reviewer for pointing it out. We have corrected this word to ‘UniFrac’ and checked it throughout the manuscript (Line: 582).

--L582: Please be consistent in the numbers: "mean = 1.36 Gb" vs. "1.5" (L408).

Reply: Thanks for pointing out this typo, we have corrected this number in line number 412.

--L585: Consider removing "bacterial" unless there was some enrichment step for bacterial DNA.

Reply: We thank the reviewer for pointing it out. We have removed the word “bacterial” from this sentence (Line: 589).

Close
