# Supplementary material for: The unique composition of Indian gut microbiome, gene catalogue, and associated fecal metabolome deciphered using multi-omics approaches
Source: Gigascience. 2019 Jan 30;8(3):giz004. doi: 10.1093/gigascience/giz004 (PMC6394208; doi:10.1093/gigascience/giz004)
Supplement: reviewer_1_report_original_submission -- Sean P Kenedy, Ph.D — 7/31/2018 Reviewed [file giz004_reviewer_1_report_original_submission.pdf]

Reviewer Report

Title: The unique composition of Indian gut microbiome, gene catalogue and associated faecal metabolome deciphered using multi-omics approaches

Version: Original Submission **Date:** 7/31/2018

Reviewer name: Sean P Kenedy, Ph.D

Reviewer Comments to Author:

The study entitled "Multi-omics analysis reveals Indian gut microbiome variations due to diet and location and its implications on human health" describes an in-depth sequencing and metabolomic analysis of a unique set of samples from two distinct locations in India. The authors correlate bacterial species composition and fecal metabolites in order to draw conclusions about health in the two geographic locations and the link with diet and disease risk. Specifically, the North Central, primarily vegetarian population, consumes a high proportion of high-fat and sugary foods and ranks among the lowest for life-expectancy. This is compared to a Southern location with an omnivorous population with a much higher life expectancy and lower risks of T2D and cardiovascular disease.
The correlation and discussion of specific metabolites and risk factors in the North Indian population versus the Southern population, and the conclusions appears to be supported by the data. The authors concentrate on a limited number of major metabolites, BCAAs and SCFAs, and link these to pathways identified in the bacterial species that are present in the populations. This focused approach is quite effective and the subsequent detailed discussion of P. Copri is very relevant (previous association with rheumatoid arthritis). The importance of bacteria-driven metabolism and its association with vegetarian diets are all interesting points where this study of the Indian population brings news perspectives. Indeed the uniqueness of the Indian population, an under-sampled population, is a major contribution to the available databases. It is for this reason that I consider the work appropriate for publication with a certain number of minor revisions prior to publication:
General comments:
-Subjects were excluded if there was reported use of antibiotics during the previous month. How was this cutoff determined and was any analysis performed on the cohort to determine if there was any residual effect of antibiotic use (a known issue in India)? This could be as simple as a PCoA plot, using time since last antibiotics exposure as a variable in the 16s diversity analysis.
-Could the authors please explain their use of Greengenes 13_5? This release dates to 2013. Was SILVA tested?
-I am convinced of the utility of the study, despite some of the additional comments below. Therefore, I would request that the raw shotgun metagenomics data also be made available, and not just the assembled contigs as is currently the case. This is extremely important so that future groups can improve on assemblies and annotations as more data is generated from future studies.
Specific comments:
Line 209: "Detection of Enterotypes"
The authors use the term 'analysis of enterotypes', referring to Arumugam et al., for the analysis

performed in this section and relate the results to those found in the previous study. However the resulting two enterotypes are more accurately, and simply, called clusters, as they are based on two distinct populations in the current study only. This is in contrast to four-country, 22-metagenome analysis performed in Arumugam et al. I would suggest that the terminology be revised.
This same type of nomenclature is repeated in line 272: 'metabotype.' I thank that referring to these as clusters is more accurate and more consistent.
It is also present in the discussion (lines 400-401) and methods (699). I would just stress again that two distinct geographical locations which can be statistically separated into two groups, within a single study, does not constitute an enterotype as defined in Arumugam et al. As LOC1 and LOC2 are distinct in this study, factoring this information into clinically relevant models (lines 403-408) does not require a further variable. The analysis and conclusions about the two groups, nevertheless, appear valid.
My suggestion, if the authors wish to use the "enterotype" comparison, would be to explore how this new dataset of 110 individuals fits when combined with that from Arumugam et al. Do the samples still classify into three enterotypes, and what is the distribution across LOC1 and LOC2?
Line 235: 16S Data Analysis
The authors use rarefied reads for downstream analysis. This type of normalization, while useful for calculating UniFrac distances, is no longer accepted as the gold standard for statistical analysis of 16s data. See (McMurdie PJ, Holmes S. Waste not, want not: why rarefying microbiome data is inadmissible. PLoS computational biology. 2014). The authors should explain why they decided to use sub-sampling normalization. How the threshold of 100K was determined?
The differential analysis performed in relation to clinical data and location (lines 247-255) should be reanalyzed using current normalization methods (e.g. DeSeq2 or edgeR packages exist for R).
Lines 347-352: The addition of 110 individuals is a major contribution. Yet, I think that the authors would agree, any future metagenomics analysis of the intestinal microbiota, even those focusing on South-Asia populations, would best be accomplished using the IGC + this study's additional database. Analysis would not be performed using this study's catalog alone. Please consider rewording here to accurately present the impact of the study.
Line 561: The authors appear to perform normalization in relation to gene length, probably RPKM. Like 16s analysis, it has been demonstrated that this type of normalization is not the most appropriate for whole genome metagenomics analysis (https://doi.org/10.1186/s12864-016-2386-y). The authors should rerun the analysis to validate that the bacterial species cited in the manuscript remain significant after applying a modern normalization method such as DESeq2 or edgeR. Perhaps other significant species will also be identified.
Line 603: The reference cited does not describe the canopy-mgs algorithm. The correct reference is Nature Biotechnology volume 32, pages 822-828 (2014); 'Identification and assembly of genomes and genetic elements in complex metagenomic samples without using reference genomes.' This reference also describes MGS (metagenomic species) that the authors refer to (Line 726, and elsewhere in text).

Methods

Are the methods appropriate to the aims of the study, are they well described, and are necessary controls included? Choose an item.

Conclusions

Are the conclusions adequately supported by the data shown? Choose an item.

Reporting Standards

Does the manuscript adhere to the journal's guidelines on minimum standards of reporting? Choose an item.

Choose an item.

Statistics

Are you able to assess all statistics in the manuscript, including the appropriateness of statistical tests used? Choose an item.

Quality of Written English

Please indicate the quality of language in the manuscript: Choose an item.

Declaration of Competing Interests

Please complete a declaration of competing interests, considering the following questions:

- Have you in the past five years received reimbursements, fees, funding, or salary from an organisation that may in any way gain or lose financially from the publication of this manuscript, either now or in the future?
- Do you hold any stocks or shares in an organisation that may in any way gain or lose financially from the publication of this manuscript, either now or in the future?
- Do you hold or are you currently applying for any patents relating to the content of the manuscript?
- Have you received reimbursements, fees, funding, or salary from an organization that holds or has applied for patents relating to the content of the manuscript?
- Do you have any other financial competing interests?
- Do you have any non-financial competing interests in relation to this paper?

If you can answer no to all of the above, write 'I declare that I have no competing interests' below. If your reply is yes to any, please give details below.

I declare that I have no competing interests.

I agree to the open peer review policy of the journal. I understand that my name will be included on my report to the authors and, if the manuscript is accepted for publication, my named report including any attachments I upload will be posted on the website along with the authors' responses. I agree for my

report to be made available under an Open Access Creative Commons CC-BY license (http://creativecommons.org/licenses/by/4.0/). I understand that any comments which I do not wish to be included in my named report can be included as confidential comments to the editors, which will not be published.

I agree to the open peer review policy of the journal

To further support our reviewers, we have joined with Publons, where you can gain additional credit to further highlight your hard work (see: https://publons.com/journal/530/gigascience). On publication of this paper, your review will be automatically added to Publons, you can then choose whether or not to claim your Publons credit. I understand this statement.

Yes Choose an item.
