# Supplementary material for: The unique composition of Indian gut microbiome, gene catalogue, and associated fecal metabolome deciphered using multi-omics approaches
Source: Gigascience. 2019 Jan 30;8(3):giz004. doi: 10.1093/gigascience/giz004 (PMC6394208; doi:10.1093/gigascience/giz004)
Supplement: reviewer_1_report_revision_1 -- Sean P Kenedy, Ph.D — 11/20/2018 Reviewed [file giz004_reviewer_1_report_revision_1.pdf]

Reviewer Report

Title: The unique composition of Indian gut microbiome, gene catalogue and associated faecal metabolome deciphered using multi-omics approaches

Version: Revision 1 **Date:** 11/20/2018

Reviewer name: Sean P Kenedy, Ph.D

Reviewer Comments to Author:

I commend the authors on their openness and responsiveness to the comments from both reviewers. The additional analyses performed and the clarifications in the manuscript have resulted in a much-improved draft. The availability of both the raw amplicon and WGS data in NCBI's Sequence Read Archive is a great service to the scientific community and should ensure open access to this data and its inclusion in future studies. The origin of the samples and the sequencing data is well-documented and cohort sample collection and storage appears to adhere to ethical and technical standards.
More specifically, the updated manuscript addresses and corrects all points that I raised in the first review. These include a major reanalysis of 16S data with an updated database (SILVA December 2017 versus GreenGenes 2013) and an implementation of statistical analysis with normalization performed using DESeq2. They clarify their use of downsized data for Unifrac analysis. Further, the authors now combine their data and run downstream analysis using an "Updated-IGC." This clearly aids their analysis and broadens the appeal of the manuscript as a whole. The 9% of additional genes appears to be unique to the Indian cohort. The authors also performed the suggested enterotype comparisons with the data from Arumugam et al.
Based on this new version of the manuscript, my recommendation is the manuscript can be accepted without further scientific revision. The authors should, nevertheless, have a careful review of the text to address remaining grammatical errors and awkward phrasing. A few, non-exhaustive, examples are given:
Line 114 : Change "All the recruited individuals" to "Recruited individuals"
Line 216 : Should be "relative abundances"
Line 365 : "its inward transport in microbial cells by the BCAA transporters" would be better as "its uptake by microbes via BCAA transporters"
Line 408 : Change "Though, the sequencing depth in the study was not too high..." to "Although sequencing depth was modest….longer paired-ends reads, from the cohort of 110 individuals appears sufficient to provide the first insights on the Indian gut microbiome"
Line 446 : "One aspect to this could" could be better written as "One potential explanation could be…"
Line 486 : "has known health benefits..." might be better as "has been reported to be beneficial by preventing…."
Line 505 : "are emerging, which results in the increased…." is better as "are emerging, with results showing increased….

Methods

Are the methods appropriate to the aims of the study, are they well described, and are necessary controls included? Choose an item.

Conclusions

Are the conclusions adequately supported by the data shown? Choose an item.

Reporting Standards

Does the manuscript adhere to the journal's guidelines on minimum standards of reporting? Choose an item.

Choose an item.

Statistics

Are you able to assess all statistics in the manuscript, including the appropriateness of statistical tests used? Choose an item.

Quality of Written English

Please indicate the quality of language in the manuscript: Choose an item.

Declaration of Competing Interests

Please complete a declaration of competing interests, considering the following questions:

- Have you in the past five years received reimbursements, fees, funding, or salary from an organisation that may in any way gain or lose financially from the publication of this manuscript, either now or in the future?
- Do you hold any stocks or shares in an organisation that may in any way gain or lose financially from the publication of this manuscript, either now or in the future?
- Do you hold or are you currently applying for any patents relating to the content of the manuscript?
- Have you received reimbursements, fees, funding, or salary from an organization that holds or has applied for patents relating to the content of the manuscript?
- Do you have any other financial competing interests?
- Do you have any non-financial competing interests in relation to this paper?

If you can answer no to all of the above, write 'I declare that I have no competing interests' below. If your reply is yes to any, please give details below.

I declare that I have no competing interests

I agree to the open peer review policy of the journal. I understand that my name will be included on my report to the authors and, if the manuscript is accepted for publication, my named report including any attachments I upload will be posted on the website along with the authors' responses. I agree for my report to be made available under an Open Access Creative Commons CC-BY license (http://creativecommons.org/licenses/by/4.0/). I understand that any comments which I do not wish to be included in my named report can be included as confidential comments to the editors, which will not be published.

I agree to the open peer review policy of the journal

To further support our reviewers, we have joined with Publons, where you can gain additional credit to further highlight your hard work (see: https://publons.com/journal/530/gigascience). On publication of this paper, your review will be automatically added to Publons, you can then choose whether or not to claim your Publons credit. I understand this statement.

Yes Choose an item.
