# Supplementary material for: The unique composition of Indian gut microbiome, gene catalogue, and associated fecal metabolome deciphered using multi-omics approaches
Source: Gigascience. 2019 Jan 30;8(3):giz004. doi: 10.1093/gigascience/giz004 (PMC6394208; doi:10.1093/gigascience/giz004)
Supplement: reviewer_2_report_original_submission -- CÃ©dric Laczny — 8/14/2018 Reviewed [file giz004_reviewer_2_report_original_submission.pdf]

Reviewer Report

Title: The unique composition of Indian gut microbiome, gene catalogue and associated faecal metabolome deciphered using multi-omics approaches

Version: Original Submission **Date:** 8/14/2018

Reviewer name: CÃ©dric Laczny

Reviewer Comments to Author:

SUMMARY
In this manuscript Dhakan & Maji et al. report on their multi-omic analyses of 110 healthy individuals from two distinct regions in India. The authors obtained 16S rRNA gene (V3 region) amplicon sequencing data, metagenomic sequencing data, and metabolomic data from volunteers' faecal samples. In addition, metabolomic data from serum samples were obtained. Using the metagenomic sequencing data, the existing Integrated Gene Catalog (IGC) was expanded by adding novel, non-redundant genes derived from the India cohort. This represents an important addition to the IGC, thereby further complementing the global, human gut-derived microbial gene catalog. The authors compared the taxonomic composition (amplicon and metagenomic data) and the functional potential (metagenomic data) of Indian-derived gut samples to samples from earlier studies (China, Denmark, USA) and found the Indian microbiome to be largely distinct. The authors conclude that diet is likely to be a strong factor in this, especially since the eating habits are often strongly conserved according to region. Using the metabolomic data, Dhakan & Maji et al. identified differences in the faecal and serum concentrations according to region.
GENERAL COMMENTS
Overall, I think that this study nicely complements existing microbiome studies by further expanding gut microbiome characterization to include samples derived from an Indian population and from different diets (plant-based and omnivorous). Moreover, it highlights the importance of complementary omics, here, metabolomics, in the study of host-microbe interactions.
While many of the authors' conclusions are supported by the reported results, I found that some conclusions need to be toned down as there is not sufficient supporting evidence for these conclusions. Please also see my detailed comments. The metagenomic sequencing depth in this study is unfortunately not particularly deep, but neither is it shallow. While sequencing depth is always a limiting factor, it is an important factor if the objective is the recovery of novel genetic/genomic information. This needs to be considered when concluding. Moreover, I found the variation/spread of the samples from the Indian cohort exceptionally large (Fig. 1 B). This might be something the authors could elaborate on.
An experiment which I would have liked to see - I am not saying that it is necessary, though - is an ordination of the 110 samples alone, i.e., not contrasting against samples from other studies but rather within the current study. I would be curious to know if there is substantial separation of samples according to region and/or diet.
Finally, I would strongly encourage the authors to be more careful with their conclusions on "the gut

microbiome and
its functional consequences on human health". The present study did not investigate "non-healthy" individuals from the respective regions. It might very well be that the same or very similar observations would have been made with respect to faecal/serum metabolite levels and correlations to respective microorganisms if "non-healthy" individuals were included
The Data Description section should be extended. It should include description of the metabolomic data that was generated as well as of the metadata which was collected (Age, BMI, etc.). Some of this information is provided in the Methods "Study design and subject enrolment" and should be moved to the Data Description instead.
Instead of reporting "thresholded" p-values (e.g., "P<0.05)"), please report the actual p-values.
I would encourage the authors to include the version and parameters of tools that were used in the Methods.
Moreover, it appears that references are occasionally missing, e.g., for the WMW test, FDR-adjustment, Polyserial correlation/biserial correlations, Reporter features algorithm, etc.
The readability of the manuscript should be further improved, e.g., by involving a professional editing service.
My comments below refer to the second row of line numbers, i.e., the one _not_ in typewriter font.
TITLE
Title: "its implications on human health": It is not clear what the "its" refers to. I would suggest adjusting the title accordingly. Moreover, while it has been shown that diet has an effect on the gut microbiome, I do not know whether "due" is the right wording here. I prefer how the authors phrased it in the abstract, e.g., "showed associations with". I would thus recommend a more careful wording. Moreover, no "non-healthy" individuals were included in the present study, hence making the conclusion of "implications" rather difficult due to lack of supporting evidence (s.a., my general comments)
ABSTRACT
L25: "comprehensively": This could be debated, e.g., at what sequencing depth would one consider to have covered the composition and/or function "comprehensively". Please remove this.
L26: "including 16S rRNA marker gene and shotgun metagenomics": This sounds to me as if the "16S rRNA marker gene" sequencing is also considered "metagenomics", which it is not. I would thus suggest "including 16S rRNA gene amplicon sequencing, metagenomic sequencing, and ...".
L32: "BCAA": This abbreviation was not introduced before. Same applies to "SCFA in L34". Please adjust accordingly throughout and for all other abbreviations in the manuscript.
L37: "BCAAs were found higher": "higher" in what? I assume in concentration, but this should be clarified in the text.
L41: "its functional consequences on human health": I think that this is too strong of a claim here. In particular, this study involved only healthy individuals, hence, while there have been differences observed, these differences may not necessarily have a positive or negative effect, but could be neutral. Put differently, different gut microbiomes may be related to healthy individuals or "non-healthy" individuals might have revealed similar findings.
MAIN TEXT
L63: "constitution": This typically refers to the "the highest laws of a sovereign state, a federated state, a country or other polity." (https://en.wikipedia.org/wiki/Constitution_(disambiguation)). The authors

should consider reformulating this, e.g., by using "condition" or a more appropriate term. Maybe the authors were referring to "composition"? It is not really clear to me, especially with respect to "understanding its variability". It is not just the taxonomic but also the functional composition which has been shown to be of importance. Hence, I would encourage the authors to clarify their point more explicitly here. Finally, this sentence may be misleading as "dysbiosis" is typically used when comparing (at least) one phenotype (e.g., lean) to another (e.g., obese). However, this study is focussed only on one phenotype, i.e., "healthy".

L69: "WGS": This abbreviation was not properly introduced. Please make sure to do so for all abbreviations throughout the manuscript.

L72: "Branch" -> "Branched".

L83: I would rephrase "from the major world populations".

L86: I would rephrase "equally dominated". Typically, "domination" is used when a single entity has a majority stake.

L114: I am not sure if these two locations as well as the total cohort size (n = 110) qualify as being "representative". I would thus suggest to remove the respective wording. Same applies to "comprehensive" , s.a., my respective comment above.

L115: "16S rRNA sequencing" -> "16S rRNA gene sequencing".

L133ff: Was the assembly done on reads from individual samples or on the pooled set of reads? It is not clear as the authors emphasize pooling in the subsequent sentence which reads to me as if this was _not_ done to generate the 1,337,547 contigs. Please clarify.

L139: Please remove "In addition". It sounds as if this is a result from the current paper but it is not.

L141: "populations" seems inappropriate here as the HMP and MetaHIT projects both involved multiple populations themselves.

L145 + L146: Please specify what the numbers in the brackets with the "plus-minus" mean. Are they representing the standard deviation?

L147f: I am not sure what the authors wanted to say here. Do they mean that reads from _other_ studies were mapped to the original IGC as well as to the updated IGC?

L150f: Please rephrase this to reflect that only a _subset_ of the genes of the 110 Indian gut samples in the current study are not represented in other gut microbiome datasets. After all, 718,360 of the 1,479,998 non-redundant genes were added to the original IGC but not the full extent of the current non-redundant genes.

L157: "non-reference" -> "reference-independent".

L159: Please remove "higher", it does not seem to fit here.

L164: "PCA" stands for "Principal Component Analysis", hence, the second "analysis" in the text is redundant.

L166: Actually, if the data was projected to PC1, there would be quite some overlap. The separation is actually benefiting from _both_ dimension, PC1 _and_ PC2. I would suggest removing the "at PC1" altogether.

L174: "16S rRNA markers" -> "16S rRNA gene markers".

L175f: While, indeed, the amplicon and, to some extent, the metagenomic data suggest members of the Prevotellaceae to be enriched in the present cohort, referring to this family as a marker should be supported by quantitative analyses, e.g., statistical analysis of differences in group means (t-Test or

WMW-test) or a classification-based approach (feature selection).

L184ff: This paragraph needs to be revised as it currently is hard to read. The sentence in L193f was especially hard to read and I am still unsure about what "The proportion of essential genes covered by top-ranking nine eggNOG clusters" means: What is the meaning of "nine" in this context when the authors refer to 15,000 to 30,000 eggNOG clusters later.

L196f: It was not readily clear to me what "alpha diversity (Shannon) calculations using gene abundances" meant and I found the Methods lacking on this point. What gene(s) was/were used ? Moreover, Fig. S4's legend mentions "gene proportions". How does this relate to "gene abundances"? It seems, from the Methods, that rarefaction was used, while the remaining information is scarce on this point. However, this is an important point as the sequencing depth in the current study (mean of 4,545,280 reads/sample) is not particularly deep (cf. Table 1) and, hence, gut microbes' genomes may be covered only partially. In the study by Qin et al . (2010), an order of magnitude more reads per sample ("an average of 62.5 million reads") were produced, albeit at rather short sequencing lengths of 75 bp (compared to 150 bp in the current study).

L202: What does "Eigen values, and their scores" mean, i.e., what is a "score" here? Moreover, they are spelled "eigenvalues", i.e., in one word. Please correct throughout.

L203: I am not sure if the authors refer here to "significantly" in a statistical sense or not. If so, please include respective quantitive results to support this conclusion.

L206: How was the odds-ratio computed? In the Methods, the description refers to LOC1 and LOC2, albeit, it seemed, i.e., I was not sure, that a comparison of Indian microbiome vs. "Other" microbiome was intended. If this is the case, the authors should clarify this in the Methods, i.e., that not only was LOC1 compared against LOC2 but also "Indian" vs. "Other" (maybe among other pairwise comparisons).

L216ff: I welcome the careful wording chosen by the authors here. It appears that there is no detailed dietary information available which could have been used to further support the authors' hypothesis, but they might want to highlight this as a window of opportunity for future study, i.e., including something like a food-frequency questionaire to be able to quantitatively assess possible links to diet.

L227: Could the authors please elaborate on how the "Spearman's correlation coefficient" was used in this context? I would have applied Fisher's exact test here.

L235: "16S rRNA" -> "16S rRNA gene"

L236: The term "PCA" has been used previously, so this is not the place to introduce the abbreviation.

L240: It was not clear to me if "taxonomic and functional diversity" were combined here or not. However, this is important to clarify as taxonomy and function are only partially linked.

L255: Is this analysis based on amplicon or based on metagenomic sequencing data? L247 indicates the former, while MGS/CAGs are defined based on the latter. Please clarify in the text.

L260: Please list "the two species".

L262: Isn't "high fiber-rich" redundant? I.e., either "diet high in fiber" or "fiber-rich diet".

L274: The conclusion drawn by the authors about the OPLS-DA results is misleading, s.a., https://www.ncbi.nlm.nih.gov/pmc/articles/PMC4990351/. Specifically, the OPLS-DA model integrates the class information with the aim to _increase_ the between-class separation. Hence, the separation observed in Fig. 4C may (partially) be a consequence of the method used and not of actual separation being in the data. An unsupervised method should be used to check for the presence of meaningful separation followed by a supervised method to perform quantitative evaluation, e.g., PERMANOVA, to

check how much of the variance is explained by the respective covariates.
L298f: I am not sure if I understood the authors' point right here. "result of its inward transport in microbial cells by the BCAA transporters, thus leading to their accumulation in the colon lumen": Do the authors' mean "uptake by the bacteria, i.e., transport into the microbial cell"? If so, I would not expect an accumulation in the lumen as such.
L305: Where do the authors show this comparison (serum vs. faeces)? Fig. 6A compares Valine and Isoleucine in LOC1 samples and LOC2 samples, but not serum vs. faeces.
L328: "the major pathway utilized by this species for BCAA biosynthesis": I am not sure in how much the metagenomic and metabolomic data in this study allow to draw this statement. Metatranscriptomic and metaproteomic data would likely be needed here. I would thus suggest that the authors qualify/nuance this statement.
L375ff: The average age of the cohort is rather low (mean of 29.72 years). Age, however, is an important factor for rheumatoid arthritis. Hence, "A probable explanation" could be toned down to "One aspect to this could be ...".
L419: "isoluecine" -> "isoleucine".
L439f: The second part of the sentence is redundant with the first part and could be removed, or vice versa.
L459 - 460: "which appears promising in reducing the metabolic risk factors originating through the interactions between diet and gut microbes to maintain a healthy gut flora": This reads misleading as the "diet" was binary, i.e., "vegetarian" vs. omnivorous" and such a statement likely requires for more fine-grained and specialized studies than were performed in this work. Please adjust accordingly.
L463ff: This entire paragraph reads redundant with the remainder of the Discussion and should thus be removed or substantially shortened.
L599: "non-reference" -> "reference-independent".
L610: Could the authors please, in analogy to their HMP+NCBI results, report how many of the remaining genes aligned to UNIREF?
L611f: This sentence should be rephrased.
L706f: How was this assessed and where can the interested reader find the results for this statement?
L709ff: It is not clear how the "Between class analysis" was peformed. The authors should provide the respective details, e.g., which test, implementation etc.
L720: Does "geography" refer to "location" (LOC1 or LOC2) here?
L732: Why was the negative correlation not considered?
METHODS
L485: Do you mean the respective table in "Additional_file_1.doc"? Not sure whether this is under the control of the authors, but it should be checked in the proof that the information is consistently named and can be readily found.
L507: "16S rRNA" -> "16S rRNA gene"
L534: "phylogenetic distances between reads": Not sure, but did the authors mean "phylogenetic distances between the samples" here?
L539f: How were host-origin reads identified? Which tool, version, and parameters?
L561ff: This is probably for the formal proofs, but I would strongly encourage to properly format here as it seems that, e.g, "bi" is supposed to read "b subscript i".

L1037ff: Please check whether "<" and "> are used correctly here." Typically "p < 0.05 is " considered significant and _not_ "P-value>0.05".
TABLES
I do not know whether the information provided in Table 2 necessitates a separate table. I leave this up to the authors to decide and to potentially discus this with the journal.
FIGURES
5: "Logs-Odd Ratio" -> "Log-Odds Ratio"
S6: The labels on the x-axis and y-axis were not readable. Please adjust accordingly. Moreover, I am not sure in how much the "clouds" add value here. They are not further discussed in the text and, hence, could be omitted for clarity.
LEGENDS
Throughout: Please verify correct use of "16S rRNA" and "16S rRNA gene".
L1015: "MWAS": Shouldn't this be "MGWAS"?
L1027: What does "Eigen values and their scores" mean, i.e., what is a "score" here?
L1092ff: This reads more like a discussion/conclusion and I would thus suggest to remove this from the figure legend.

Methods

Are the methods appropriate to the aims of the study, are they well described, and are necessary controls included? Choose an item.

Conclusions

Are the conclusions adequately supported by the data shown? Choose an item.

Reporting Standards

Does the manuscript adhere to the journal's guidelines on [minimum standards of reporting?] Choose an item.

Choose an item.

Statistics

Are you able to assess all statistics in the manuscript, including the appropriateness of statistical tests used? Choose an item.

Quality of Written English

Please indicate the quality of language in the manuscript: Choose an item.

Declaration of Competing Interests

Please complete a declaration of competing interests, considering the following questions:

- Have you in the past five years received reimbursements, fees, funding, or salary from an organisation that may in any way gain or lose financially from the publication of this manuscript, either now or in the future?
- Do you hold any stocks or shares in an organisation that may in any way gain or lose financially from the publication of this manuscript, either now or in the future?
- Do you hold or are you currently applying for any patents relating to the content of the manuscript?
- Have you received reimbursements, fees, funding, or salary from an organization that holds or has applied for patents relating to the content of the manuscript?
- Do you have any other financial competing interests?
- Do you have any non-financial competing interests in relation to this paper?

If you can answer no to all of the above, write 'I declare that I have no competing interests' below. If your reply is yes to any, please give details below.

I declare that I have no competing interests.

I agree to the open peer review policy of the journal. I understand that my name will be included on my report to the authors and, if the manuscript is accepted for publication, my named report including any attachments I upload will be posted on the website along with the authors' responses. I agree for my report to be made available under an Open Access Creative Commons CC-BY license (http://creativecommons.org/licenses/by/4.0/). I understand that any comments which I do not wish to be included in my named report can be included as confidential comments to the editors, which will not be published.

I agree to the open peer review policy of the journal

To further support our reviewers, we have joined with Publons, where you can gain additional credit to further highlight your hard work (see: https://publons.com/journal/530/gigascience). On publication of this paper, your review will be automatically added to Publons, you can then choose whether or not to claim your Publons credit. I understand this statement.

Yes Choose an item.
