# Supplementary material for: The unique composition of Indian gut microbiome, gene catalogue, and associated fecal metabolome deciphered using multi-omics approaches
Source: Gigascience. 2019 Jan 30;8(3):giz004. doi: 10.1093/gigascience/giz004 (PMC6394208; doi:10.1093/gigascience/giz004)
Supplement: reviewer_2_report_revision_1 -- CÃ©dric Laczny — 11/21/2018 Reviewed [file giz004_reviewer_2_report_revision_1.pdf]

Reviewer Report

Title: The unique composition of Indian gut microbiome, gene catalogue and associated faecal metabolome deciphered using multi-omics approaches

Version: Revision 1 **Date:** 11/21/2018

Reviewer name: CÃ©dric Laczny

Reviewer Comments to Author:

The authors have reasonably addressed the comments I raised in the original submission.
Only one general comment and a few minor comments remain, which should all be readily addressable by the authors.
General comments:
L286-291: It would be good to test whether the location and diet are correlated and to which extent. In fact, given the information from the authors, I would expect them to be correlated. Hence, the observed results (Fig. S12) are to be expected and this should be qualified. If no such test is performed, I would recommend to at least reemphasize the (strong) influence of location on the diet of the studied Indian populations. This is also important with respect to the results in L333-335.
Minor comments:
Throughout: Frequently, "the"/"a" is missing, e.g., L158 "analysis of microbiome", L159 "reads from other three datasets", L163 "This shows that the addition of subset".
L149-150 - "and unique to IGC" : This reads as if the 943,395 genes are unique to the IGC, but aren't his unique to the newly constructed Indian microbial gene catalogue?
L161 - "did not show a significant (P< 0.01)": Not sure if the signficance level (alpha = 0.01) is meant here or if the p-value was "< 0.01". In the latter case, it woud be considered significant at alpha = 0.01. Please clarify and verify throughout.
L212-214: Species names should be italicized.
L270: "be" is missing -> "needs to be collected".
L275: The text suggests a "significance", yet the p-value is listed as 0.6841. Please clarify.
Supplements: Fig S11 still contains a reference to "enterotypes" which, as suggested by Reviewer 1 (and I agree) should be generally avoided, unless in combination with the non-Indian populations. Please check this throughout.
L304-305: This is not a necessity for the revision, but rather a question out of curiosity: Was an association with age tested here, in addition to BMI?
L317 + L319: What do "19 MGS/CAG" and "67 MGS/CAG" refer to here? Are these the numbers of MGSs/CAGs that were annotated to likely be P. copri populations, i.e., multiple strains/sub-species of P. copri were identified? Please clarify this.
L339: Did Cluster-2 show *no* association with location, i.e, was a mixture of samples from LOC1 and LOC2?
Legend Fig.S17: "OPLD-DA" -> "OPLS-DA "
Fig.S18: Panel A is rather small and the fonts are hard to read. Please increase the size of the panel.

L409-411: I welcome the qualification of the sequencing depth here. Nevertheless, the argument of 2x150bp sequencing is misleading here. Read-length clearly plays a role, so does the overall sequencing depth. While 2x150bp is commonly used currently, and hence the current study is up-to-date, I would suggest the authors to rephrase this slightly. My suggestion would be: "... deviation), the inclusion of 110 individuals from two distinct geographic locations as well as the identification of Indian gut microbiome-specific genes provide a first insight into the Indian gut microbiome and are thus considered important additions to the field."
L411-413: This sentence reads contradictory in itself. If there is a high diversity, how can (only) two locations be considered representative? I would suggest to rephrase this.
L431: It is not readily clear what "Its" refers to here. I assume it is "Prevotella", yet this should be clarified.
L439: Please consider removing "driver" unless you can show a causation rather than the association which was presented in the results.
L442: "bacteria" -> "bacterium"
L470-471: The "statistically sound" is not readily clear here. Please consider removing this as I do not find it relevant in this context.
L500: "Firmicute" -> "Firmicutes"
L515: Please remove "populations", it does not fit in here.
L578: Please check correct capitalization.
L582: Please be consistent in the numbers: "mean = 1.36 Gb" vs. "1.5" (L408).
L585: Consider removing "bacterial" unless there was some enrichment step for bacterial DNA.

Methods

Are the methods appropriate to the aims of the study, are they well described, and are necessary controls included? Choose an item.

Conclusions

Are the conclusions adequately supported by the data shown? Choose an item.

Reporting Standards

Does the manuscript adhere to the journal's guidelines on minimum standards of reporting? Choose an item.

Choose an item.

Statistics

Are you able to assess all statistics in the manuscript, including the appropriateness of statistical tests used? Choose an item.

Quality of Written English

Please indicate the quality of language in the manuscript: Choose an item.

Declaration of Competing Interests

Please complete a declaration of competing interests, considering the following questions:

- Have you in the past five years received reimbursements, fees, funding, or salary from an organisation that may in any way gain or lose financially from the publication of this manuscript, either now or in the future?
- Do you hold any stocks or shares in an organisation that may in any way gain or lose financially from the publication of this manuscript, either now or in the future?
- Do you hold or are you currently applying for any patents relating to the content of the manuscript?
- Have you received reimbursements, fees, funding, or salary from an organization that holds or has applied for patents relating to the content of the manuscript?
- Do you have any other financial competing interests?
- Do you have any non-financial competing interests in relation to this paper?

If you can answer no to all of the above, write 'I declare that I have no competing interests' below. If your reply is yes to any, please give details below.

I declare that I have no competing interests

I agree to the open peer review policy of the journal. I understand that my name will be included on my report to the authors and, if the manuscript is accepted for publication, my named report including any attachments I upload will be posted on the website along with the authors' responses. I agree for my report to be made available under an Open Access Creative Commons CC-BY license (http://creativecommons.org/licenses/by/4.0/). I understand that any comments which I do not wish to be included in my named report can be included as confidential comments to the editors, which will not be published.

I agree to the open peer review policy of the journal

To further support our reviewers, we have joined with Publons, where you can gain additional credit to further highlight your hard work (see: https://publons.com/journal/530/gigascience). On publication of this paper, your review will be automatically added to Publons, you can then choose whether or not to claim your Publons credit. I understand this statement.

Yes Choose an item.
