# Supplementary material for: The unique composition of Indian gut microbiome, gene catalogue, and associated fecal metabolome deciphered using multi-omics approaches
Source: Gigascience. 2019 Jan 30;8(3):giz004. doi: 10.1093/gigascience/giz004 (PMC6394208; doi:10.1093/gigascience/giz004)
Supplement: Supplemental Files [file giz004_supplemental_files.zip › Additional_file_1.doc]

**Additional File 1: Supplementary data**

**Study population:**

The study cohort consisted of 110 healthy individuals including infants, adults and aged individuals from two different locations across India i.e., Bhopal (LOC1, n=53) and Kerala (LOC2, n=57), which are separated by ~2,000 kilometers. LOC1 is dominated by vegetarians, whereas the population of LOC2 has dietary habits mostly consisting of rice, seafoods and red meat. The sample were collected from three regions in LOC2 including Kanhangad, Neeleswar and Trichur, which were upto ~250 kilometers apart from each other.

Background map taken from http://gurudeva.weebly.com/maps.html

**Diet description:**

In LOC1, the individuals are mostly vegetarian with diet including *poha* (a rice-based snack), rice, *roti* (a handmade wheat bread), *dal* (split pulses, in particular lentils), vegetables, a variety of dairy products (milk, curd, clarified butter, etc.) and trans-fat food (including oil fried snacks, sandwiches, etc.). Vegetable oil, hydrogenated oil, butter and *Vanaspati* oil are used for the preparation of most of the dishes in LOC1.

The staple food for LOC2 is rice and mostly the meals consist of rice as the chief ingredient. including *idli* (made with rice and *udad* dal/split black gram), *puttu* (steamed rice powder) and other dishes made from rice. LOC2 diet includes a wide variety of non-vegetarian food such as chicken, beef, pork and mutton and majority of the population is omnivorous. The diet also includes a variety of sea food in which mackerel and sardine are the prominent fishes, apart from other river fishes and backwater fishes. The ingredients of curry mostly include coconut (chopped or grated), coconut milk, coconut oil, chilies, curry leaf, mustard seeds and tamarind.

**Table 1: Metadata Information**

| **Sample** | **Age(Years)** | **Age Group** | **Gender** | **Diet** | **Location** | **BMI** |
| --- | --- | --- | --- | --- | --- | --- |
| HA | 26 | Adult | M | Omnivorous | LOC1 | 22.72 |
| HAA | 27 | Adult | F | Omnivorous | LOC1 | 21.5 |
| HAB | 28 | Adult | F | Vegetarian | LOC1 | 19.28 |
| HAC | 21 | Young | F | Vegetarian | LOC1 | 28.12 |
| HAD | 27 | Adult | F | Vegetarian | LOC1 | 23.87 |
| HAE | 21 | Young | F | Vegetarian | LOC1 | 19.22 |
| HAF | 21 | Young | F | Vegetarian | LOC1 | 28.84 |
| HAG | 23 | Young | F | Vegetarian | LOC1 | 22.3 |
| HAH | 25 | Young | F | Vegetarian | LOC1 | 21.26 |
| HAI | 24 | Young | M | Vegetarian | LOC1 | 26.03 |
| HAJ | 22 | Young | F | Vegetarian | LOC1 | 23.23 |
| HAK | 29 | Adult | F | Vegetarian | LOC1 | 22.03 |
| HAL | 24 | Young | F | Vegetarian | LOC1 | 23.25 |
| HAM | 23 | Young | F | Vegetarian | LOC1 | 19.22 |
| HAN | 26 | Adult | F | Vegetarian | LOC1 | 22.03 |
| HAO | 26 | Adult | F | Vegetarian | LOC1 | 20.82 |
| HAP | 25 | Young | M | Vegetarian | LOC1 | 21.71 |
| HAR | 28 | Adult | M | Vegetarian | LOC1 | 25.51 |
| HAT | 26 | Adult | M | Vegetarian | LOC1 | 22.65 |
| HB | 23 | Young | M | Omnivorous | LOC1 | 22.2 |
| HC | 23 | Young | M | Vegetarian | LOC1 | 23.52 |
| HCL | 64 | Aged | F | Omnivorous | LOC1 | 36.4 |
| HCM | 62 | Aged | F | Omnivorous | LOC1 | 22.9 |
| HCN | 57 | Aged | M | Omnivorous | LOC1 | 17.9 |
| HCO | 57 | Aged | F | Omnivorous | LOC1 | 20.8 |
| HCP | 71 | Aged | M | Omnivorous | LOC1 | 19.2 |
| HCQ | 52 | Aged | F | Omnivorous | LOC1 | 23 |
| HCR | 62 | Aged | M | Omnivorous | LOC1 | 20.8 |
| HCS | 52 | Aged | F | Omnivorous | LOC1 | 17.9 |
| HCT | 58 | Aged | M | Omnivorous | LOC1 | 19.2 |
| HCU | 61 | Aged | M | Omnivorous | LOC1 | 19.5 |
| HCV | 58 | Aged | F | Omnivorous | LOC1 | 19.9 |
| HD | 29 | Adult | M | Vegetarian | LOC1 | 20.71 |
| HE | 22 | Young | M | Vegetarian | LOC1 | 22.23 |
| HF | 30 | Adult | M | Vegetarian | LOC1 | 24.38 |
| HG | 30 | Adult | M | Omnivorous | LOC1 | 22.47 |
| HI | 2.4 | Toddler | F | Vegetarian | LOC1 | 11.12 |
| HJ | 23 | Young | M | Vegetarian | LOC1 | 27.88 |
| HK | 26 | Adult | F | Vegetarian | LOC1 | 22.3 |
| HL | 29 | Adult | M | Vegetarian | LOC1 | 23.4 |
| HM | 30 | Adult | M | Omnivorous | LOC1 | 23.38 |
| HN | 28 | Adult | M | Omnivorous | LOC1 | 22.58 |
| HO | 26 | Adult | M | Vegetarian | LOC1 | 23.78 |
| HP | 30 | Adult | M | Vegetarian | LOC1 | 19.36 |
| HQ | 24 | Young | F | Omnivorous | LOC1 | 23.14 |
| HR | 26 | Adult | M | Vegetarian | LOC1 | 22.22 |
| HS | 25 | Young | M | Vegetarian | LOC1 | 18.3 |
| HT | 25 | Young | F | Vegetarian | LOC1 | 22.6 |
| HU | 28 | Adult | F | Vegetarian | LOC1 | 21.3 |
| HV | 6 Months | Toddler | F | Vegetarian | LOC1 | NA |
| HW | 6 Months | Toddler | F | Vegetarian | LOC1 | NA |
| HY | 8 Months | Toddler | F | Vegetarian | LOC1 | NA |
| HZ | 29 | Adult | F | Vegetarian | LOC1 | 28.04 |
| C10 | 7 | Children | F | Omnivorous | LOC2 | 12.45 |
| C13 | 11 | Children | M | Omnivorous | LOC2 | 12.92 |
| C14 | 12 | Children | M | Omnivorous | LOC2 | 12.85 |
| C15 | 11 | Children | F | Omnivorous | LOC2 | 12.6 |
| C16 | 16 | Children | M | Omnivorous | LOC2 | 13.25 |
| C19 | 9 | Children | F | Omnivorous | LOC2 | 12.5 |
| C2 | 12 | Toddler | F | Omnivorous | LOC2 | 13.26 |
| C22 | 4 | Children | M | Omnivorous | LOC2 | 11.8 |
| C23 | 6 | Toddler | M | Omnivorous | LOC2 | 12.1 |
| C24 | 5 | Children | F | Omnivorous | LOC2 | 11.56 |
| C25 | 7 | Children | F | Omnivorous | LOC2 | 11.2 |
| C3 | 12 | Children | M | Omnivorous | LOC2 | 12.86 |
| C4 | 10 | Children | F | Omnivorous | LOC2 | 13.2 |
| C5 | 6 | Children | M | Omnivorous | LOC2 | 12.65 |
| C6 | 9 | Toddler | M | Omnivorous | LOC2 | 12.8 |
| C7 | 5 | Toddler | M | Omnivorous | LOC2 | 11.65 |
| C9 | 3.5 | Children | M | Omnivorous | LOC2 | 11.2 |
| C18 | 10 | Children | M | Omnivorous | LOC2 | 12.8 |
| HAD | 50 | Aged | M | Omnivorous | LOC2 | 22.5 |
| HAU | 30 | Adult | M | Omnivorous | LOC2 | 27.84 |
| HAV | 24 | Young | M | Omnivorous | LOC2 | 33.8 |
| HAX | 25 | Young | M | Omnivorous | LOC2 | 16.04 |
| HAZ | 23 | Young | M | Omnivorous | LOC2 | 21.13 |
| HBA | 23 | Young | M | Omnivorous | LOC2 | 25.1 |
| HBB | 23 | Young | M | Omnivorous | LOC2 | 28.82 |
| HBC | 19 | Young | M | Omnivorous | LOC2 | 25.9 |
| HBD | 20 | Young | M | Omnivorous | LOC2 | 17.61 |
| HBE | 30 | Adult | F | Omnivorous | LOC2 | 23.3 |
| HBF | 30 | Adult | F | Omnivorous | LOC2 | 17.08 |
| HBG | 29 | Adult | F | Omnivorous | LOC2 | 26.63 |
| HBH | 26 | Adult | F | Omnivorous | LOC2 | 23.92 |
| HBI | 28 | Adult | F | Omnivorous | LOC2 | 24.32 |
| HBJ | 27 | Adult | F | Omnivorous | LOC2 | 20.61 |
| HBK | 21 | Young | F | Omnivorous | LOC2 | 21.5 |
| HBL | 30 | Adult | F | Omnivorous | LOC2 | 16.56 |
| HBM | 23 | Young | F | Omnivorous | LOC2 | 17.74 |
| HBR | 50 | Aged | F | Omnivorous | LOC2 | 27.1 |
| HBV | 55 | Aged | F | Omnivorous | LOC2 | 24.97 |
| HBW | 55 | Aged | F | Omnivorous | LOC2 | 25.88 |
| HBY | 60 | Aged | F | Omnivorous | LOC2 | 26.14 |
| HBZ | 50 | Aged | F | Omnivorous | LOC2 | 25.47 |
| HCA | 50 | Aged | F | Omnivorous | LOC2 | 32.04 |
| HCB | 50 | Aged | F | Omnivorous | LOC2 | 21.1 |
| HCD | 56 | Aged | M | Omnivorous | LOC2 | 24.38 |
| HCF | 58 | Aged | M | Omnivorous | LOC2 | 25.51 |
| HCH | 53 | Aged | M | Omnivorous | LOC2 | 25.86 |
| HCI | 53 | Aged | M | Omnivorous | LOC2 | 23.38 |
| HCJ | 60 | Aged | M | Omnivorous | LOC2 | 22.91 |
| HCK | 53 | Aged | M | Omnivorous | LOC2 | 23.83 |
| HCW | 21 | Young | M | Omnivorous | LOC2 | 22.18 |
| HCY | 53 | Aged | F | Omnivorous | LOC2 | 26.8 |
| HCZ | 25 | Young | M | Omnivorous | LOC2 | 20.61 |
| HDB | 50 | Aged | F | Omnivorous | LOC2 | 25.1 |
| HDC | 27 | Adult | F | Omnivorous | LOC2 | 20.42 |
| HDD | 30 | Adult | M | Omnivorous | LOC2 | 22.33 |
| HDE | 30 | Adult | F | Omnivorous | LOC2 | 22.43 |
| HDF | 58 | Aged | F | Omnivorous | LOC2 | 25.7 |

**Details of primers used in the study for 16S rRNA gene sequencing**

The amplification of bacterial 16S rRNA gene V3 region was performed using Illumina Nextera XT adapter-ligated eubacterial V3 region-specific primers, 341F and 534R, with seven different base modifications. Nucleotide bases were introduced in different numbers to increase the overall sequence diversity of the samples, thus improving the quality of the sequenced data. Details of each primer and the corresponding sample IDs are provided below.

**Table 2: Primer details**

| 341F-ADA_OB | TCGTCGGCAGCGTCAGATGTGTATAAGAGACAGCCTACGGGAGGCAGCAG |
| --- | --- |
| 534R-ADA_OB | GTCTCGTGGGCTCGGAGATGTGTATAAGAGACAGATTACCGCGGCTGCTGGC |
| 341F_ADA_1B | TCGTCGGCAGCGTCAGATGTGTATAAGAGACAGTCCTACGGGAGGCAGCAG |
| 534R_ADA_1B | GTCTCGTGGGCTCGGAGATGTGTATAAGAGACAGCATTACCGCGGCTGCTGGC |
| 341F_ADA_2B | TCGTCGGCAGCGTCAGATGTGTATAAGAGACAGCTCCTACGGGAGGCAGCAG |
| 534R_ADA_2B | GTCTCGTGGGCTCGGAGATGTGTATAAGAGACAGCTATTACCGCGGCTGCTGGC |
| 341F_ADA_3B | TCGTCGGCAGCGTCAGATGTGTATAAGAGACAGCATCCTACGGGAGGCAGCAG |
| 534R_ADA_3B | GTCTCGTGGGCTCGGAGATGTGTATAAGAGACAGACTATTACCGCGGCTGCTGGC |
| 341F_ADA_4B | TCGTCGGCAGCGTCAGATGTGTATAAGAGACAGTCATCCTACGGGAGGCAGCAG |
| 534R_ADA_4B | GTCTCGTGGGCTCGGAGATGTGTATAAGAGACAGCTATATTACCGCGGCTGCTGGC |
| 341F_ADA_5B | TCGTCGGCAGCGTCAGATGTGTATAAGAGACAGCTACTCCTACGGGAGGCAGCAG |
| 534R_ADA_5B | GTCTCGTGGGCTCGGAGATGTGTATAAGAGACAGCATCTATTACCGCGGCTGCTGGC |
| 341F_ ADA_REV | GTCTCGTGGGCTCGGAGATGTGTATAAGAGACAGCCTACGGGAGGCAGCAG |
| 534R_ ADA_REV | TCGTCGGCAGCGTCAGATGTGTATAAGAGACAGATTACCGCGGCTGCTGGC |

**Table 3: Sample detail with corresponding primers used to amplify 16s rRNA gene V3 region**

| **Sample ID** | **Primer used** | **Sample ID** | **Primer used** | **Sample ID** | **Primer used** | **Sample ID** | **Primer used** | **Sample ID** | **Primer used** |
| --- | --- | --- | --- | --- | --- | --- | --- | --- | --- |
| HA | 1B | HS | 2B | HAJ | 3B | HAU | 4B | HBH | 5B |
| HB | 1B | HK | 2B | HAK | 3B | HAX | 4B | HBI | 5B |
| HC | 1B | HQ | 2B | HAL | 3B | HAZ | 4B | HBJ | 5B |
| HD | 1B | HT | 2B | HAM | 3B | HBA | 4B | HBK | 5B |
| HE | 1B | HU | 2B | HAN | 3B | HBB | 4B | HBL | 5B |
| HF | 1B | HZ | 2B | HAO | 3B | HBC | 4B | HBM | 5B |
| HG | 1B | HAA | 2B | HAI | 3B | HBD | 4B | HDE | 5B |
| HJ | 1B | HAB | 2B | HAP | 3B | HDD | 4B | HDC | 5B |
| HL | 1B | HAC | 2B | HAR | 3B | HCZ | 4B | HCT | 5B |
| HM | 1B | HAD | 2B | HAT | 3B | HCW | 4B | HCR | 5B |
| HN | 1B | HAE | 2B | HI | 3B | HAV | 4B | HCP | 5B |
| HO | 1B | HAF | 2B | HV | 3B | HBE | 4B | HCN | 5B |
| HP | 1B | HAG | 2B | HY | 3B | HBF | 4B | HCU | 5B |
| HR | 1B | HAH | 2B | HW | 3B | HBG | 4B | HAD | 5B |
| HCK | REV | HCD | REV | HCM | REV | HDB | OB | HBY | OB |
| HCJ | REV | HCS | REV | HCL | REV | HCY | OB | HBV | OB |
| HCI | REV | HCQ | REV | HCV | REV | HCB | OB | HBW | OB |
| HCH | REV | HCO | REV | HDF | REV | HCA | OB |  |  |
| HCF | REV | HBR | REV | C15 | 1B | HBZ | OB |  |  |
| C2 | 1B | C7 | 1B | C16 | 1B | C23 | 1B |  |  |
| C3 | 1B | C9 | 1B | C18 | 1B | C24 | REV |  |  |
| C4 | 1B | C10 | 1B | C19 | 1B |  |  |  |  |
| C5 | 1B | C13 | 1B | C22 | 1B |  |  |  |  |
| C6 | 1B | C14 | 1B | C25 | REV |  |  |  |  |

**Table 4: Information on samples used for sequencing and metabolomics analysis**

| **Sample** | **WGS sequencing** | **16Sr rRNA gene amplicon sequencing** | **Faecal metabolomics** | **Serum Metabolomics** |
| --- | --- | --- | --- | --- |
| **HA** | Yes | Yes | Yes | No |
| **HAA** | Yes | Yes | Yes | Yes |
| **HAB** | Yes | Yes | Yes | Yes |
| **HAC** | Yes | Yes | Yes | Yes |
| **HAD** | Yes | Yes | Yes | Yes |
| **HAE** | Yes | Yes | Yes | Yes |
| **HAF** | Yes | Yes | Yes | Yes |
| **HAG** | Yes | Yes | Yes | Yes |
| **HAH** | Yes | Yes | Yes | Yes |
| **HAI** | Yes | Yes | Yes | Yes |
| **HAJ** | Yes | Yes | Yes | No |
| **HAK** | Yes | Yes | Yes | No |
| **HAL** | Yes | Yes | Yes | No |
| **HAM** | Yes | Yes | Yes | No |
| **HAN** | Yes | Yes | Yes | No |
| **HAO** | Yes | Yes | Yes | No |
| **HAP** | Yes | Yes | Yes | Yes |
| **HAR** | Yes | Yes | Yes | Yes |
| **HAT** | Yes | Yes | Yes | Yes |
| **HB** | Yes | Yes | Yes | Yes |
| **HD** | Yes | Yes | Yes | Yes |
| **HE** | Yes | Yes | Yes | Yes |
| **HF** | Yes | Yes | Yes | Yes |
| **HG** | Yes | Yes | Yes | No |
| **HI** | Yes | Yes | Yes | No |
| **HJ** | Yes | Yes | Yes | No |
| **HK** | Yes | Yes | Yes | No |
| **HL** | Yes | Yes | Yes | No |
| **HM** | Yes | Yes | Yes | No |
| **HN** | Yes | Yes | Yes | No |
| **HO** | Yes | Yes | Yes | No |
| **HP** | Yes | Yes | Yes | No |
| **HQ** | Yes | Yes | Yes | No |
| **HR** | Yes | Yes | Yes | No |
| **HS** | Yes | Yes | Yes | No |
| **HT** | Yes | Yes | Yes | No |
| **HU** | Yes | Yes | Yes | No |
| **HV** | Yes | Yes | Yes | No |
| **HW** | Yes | Yes | Yes | No |
| **HY** | Yes | Yes | Yes | No |
| **HZ** | Yes | Yes | Yes | No |
| **C10** | Yes | Yes | Yes | No |
| **C13** | Yes | Yes | Yes | No |
| **C14** | Yes | Yes | Yes | No |
| **C15** | Yes | Yes | Yes | No |
| **C16** | Yes | Yes | Yes | No |
| **C18** | Yes | No | Yes | No |
| **C19** | Yes | Yes | Yes | No |
| **C2** | Yes | Yes | Yes | No |
| **C3** | Yes | Yes | Yes | No |
| **C4** | Yes | Yes | Yes | No |
| **C5** | Yes | Yes | Yes | No |
| **C6** | Yes | Yes | Yes | No |
| **C7** | Yes | Yes | Yes | No |
| **C9** | Yes | Yes | Yes | No |
| **HAD** | Yes | Yes | Yes | No |
| **HAU** | Yes | Yes | Yes | No |
| **HAV** | Yes | Yes | Yes | Yes |
| **HAX** | Yes | Yes | Yes | Yes |
| **HAZ** | Yes | Yes | Yes | Yes |
| **HBA** | Yes | Yes | Yes | Yes |
| **HBB** | Yes | Yes | Yes | Yes |
| **HBC** | Yes | Yes | Yes | Yes |
| **HBD** | Yes | Yes | Yes | Yes |
| **HBE** | Yes | Yes | Yes | Yes |
| **HBF** | Yes | Yes | Yes | Yes |
| **HBG** | Yes | Yes | Yes | Yes |
| **HBH** | Yes | Yes | Yes | Yes |
| **HBI** | Yes | Yes | Yes | Yes |
| **HBJ** | Yes | Yes | Yes | Yes |
| **HBK** | Yes | Yes | Yes | Yes |
| **HBL** | Yes | Yes | Yes | Yes |
| **HBM** | Yes | Yes | Yes | Yes |
| **HBR** | Yes | Yes | Yes | No |
| **HBV** | Yes | Yes | Yes | No |
| **HBW** | Yes | Yes | Yes | No |
| **HBY** | Yes | Yes | Yes | No |
| **HBZ** | Yes | Yes | Yes | No |
| **HCA** | Yes | Yes | No | No |
| **HCB** | Yes | Yes | No | No |
| **HCD** | Yes | Yes | Yes | No |
| **HCF** | Yes | Yes | Yes | No |
| **HCH** | Yes | Yes | Yes | No |
| **HCI** | Yes | Yes | Yes | No |
| **HCJ** | Yes | Yes | Yes | No |
| **HCK** | Yes | Yes | Yes | No |
| **HCW** | Yes | Yes | Yes | Yes |
| **HCY** | Yes | Yes | Yes | No |
| **HCZ** | Yes | Yes | Yes | No |
| **HDB** | Yes | Yes | Yes | No |
| **HDC** | Yes | Yes | Yes | No |
| **HDD** | Yes | Yes | Yes | No |
| **HDE** | Yes | Yes | Yes | No |
| **HDF** | Yes | Yes | Yes | No |
| **HCL** | Yes | Yes | Yes | No |
| **HCM** | Yes | Yes | Yes | No |
| **HCN** | Yes | Yes | Yes | No |
| **HCO** | Yes | Yes | Yes | No |
| **HCP** | Yes | Yes | Yes | No |
| **HCQ** | Yes | Yes | Yes | No |
| **HCR** | Yes | Yes | Yes | No |
| **HCS** | Yes | Yes | Yes | No |
| **HCT** | Yes | Yes | Yes | No |
| **HCU** | Yes | Yes | Yes | No |
| **HCV** | Yes | Yes | Yes | No |
| **C22** | Yes | Yes | Yes | No |
| **C23** | Yes | Yes | Yes | No |
| **C24** | Yes | Yes | Yes | No |
| **C25** | Yes | Yes | Yes | No |
| **HC** | Yes | Yes | Yes | No |
